# Transcriptomic characterization of Lonrf1 at the single-cell level under pathophysiological conditions

**DOI:** 10.1093/jb/mvad021

**Published:** 2023-03-08

**Authors:** Dan Li, Teh-Wei Wang, Sae Aratani, Satotaka Omori, Maho Tamatani, Yoshikazu Johmura, Makoto Nakanishi

**Affiliations:** Division of Cancer Cell Biology, Institute of Medical Science, University of Tokyo, 4-6-1 Shirokane-dai, Minato-ku, Tokyo 108-8639, Japan; Division of Cancer Cell Biology, Institute of Medical Science, University of Tokyo, 4-6-1 Shirokane-dai, Minato-ku, Tokyo 108-8639, Japan; Division of Cancer Cell Biology, Institute of Medical Science, University of Tokyo, 4-6-1 Shirokane-dai, Minato-ku, Tokyo 108-8639, Japan; Department of Endocrinology, Metabolism, and Nephrology, Graduate School of Medicine, Nippon Medical School, 1-1-5 Sendagi, Bunkyo-ku, Tokyo 113-8603, Japan; Division of Cancer Cell Biology, Institute of Medical Science, University of Tokyo, 4-6-1 Shirokane-dai, Minato-ku, Tokyo 108-8639, Japan; Division of Cancer Cell Biology, Institute of Medical Science, University of Tokyo, 4-6-1 Shirokane-dai, Minato-ku, Tokyo 108-8639, Japan; Division of Cancer and Senescence Biology, Cancer Research Institute, Institute for Frontier Science Initiative, Kanazawa University, Kakuma-machi, Kanazawa 920-1192, Japan; Division of Cancer Cell Biology, Institute of Medical Science, University of Tokyo, 4-6-1 Shirokane-dai, Minato-ku, Tokyo 108-8639, Japan

**Keywords:** regulation gene, proteolytic enzyme, mapping gene, expression gene, ageing diseases

## Abstract

The LONRF family of proteins consists of three isozymes, LONRF1–3, which harbors RING (really interesting new gene) domain and Lon substrate binding domain. We have recently identified LONRF2 as a protein quality control ubiquitin ligase that acts predominantly in neurons. LONRF2 selectively ubiquitylates misfolded or damaged proteins for degradation. LONRF2^−/−^ mice exhibit late-onset neurological deficits. However, the physiological implications of other LONRF isozymes remain unclear. Here, we analysed Lonrf1 expression and transcriptomics at the single-cell level under normal and pathological conditions. We found that Lonrf1 was ubiquitously expressed in different tissues. Its expression in LSEC and Kupffer cells increased with age in the liver. Lonrf1^high^ Kupffer cells showed activation of regulatory pathways of peptidase activity. In normal and NASH (nonalcoholic steatohepatitis) liver, Lonrf1^high^ LSECs showed activation of NF-kB and p53 pathways and suppression of IFNa, IFNg and proteasome signalling independent of p16 expression. During wound healing, Lonrf1^high^/p16^low^ fibroblasts showed activation of cell growth and suppression of TGFb and BMP (bone morphogenetic protein) signalling, whereas Lonrf1^high^/p16^high^ fibroblasts showed activation of WNT (wingless and Int-1) signalling. These results suggest that although Lonrf1 does not seem to be associated with senescence induction and phenotypes, LONRF1 may play a key role in linking oxidative damage responses and tissue remodelling during wound healing in different modes in senescent and nonsenescent cells.

## Graphical Abstract

**Graphical Abstract:**
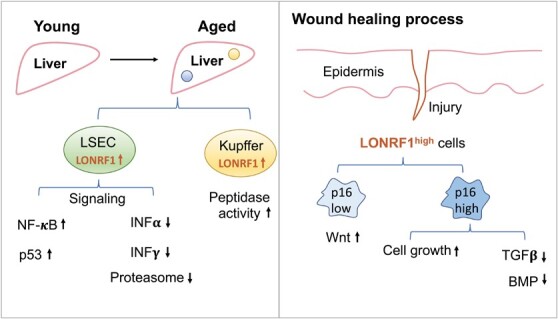

Many age-related disorders are causally linked to protein misfolding. In addition, certain environmental stressors can induce misfolding of mature proteins. To prevent this, all cells have evolved systems of protein quality control (PQC) systems that include translation control, molecular chaperone activity and proteolytic degradation by either the proteasome or via autophagy *(**1**,**2**)*.

Lon is a homo-oligomeric ATP (adenosine triphosphate)-stimulated protease conserved in prokaryotes and eukaryotic mitochondria and peroxisomes. Lon mediates the degradation of abnormal and damaged proteins and is essential for cellular homeostasis *(**3**)*. In prokaryotes, mutations in Lon greatly reduce the turnover of abnormal proteins, suggesting a role as a PQC protease *(**4**)*. Lon belongs to the AAA+ family of proteases, which consists of the N-terminal LON domain, the ATPase domain, the substrate sensor and discriminator domain and the C-terminal proteolytic active site *(**5**)*. The N-terminal LON domain is involved in protein substrate binding and the subunit oligomerization *(**6**)*. In eukaryotes, ATP-dependent proteolytic activity has been found to exist in the mitochondrial matrix and implicated in the mitochondrial Lon protease, LonP1 *(**7**)*. LonP1 degrades folded substrate proteins but prefers unfolded substrates, suggesting that it is a functional and structural homologue of the bacterial Lon protease *(**8**)*. However, LON family proteases that degrade protein substrates in the nucleus and cytoplasm have not yet been identified in eukaryotes.

Recently, we have identified LONRF2 as a PQC ubiquitin E3 ligase. Lonrf2 expression is strongly induced in senescent cells where protein aggregates accumulate. LONRF2 selectively binds and ubiquitylates misfolded or damaged proteins. Under unperturbed conditions, Lonrf2 is predominantly expressed in neurons. Importantly, loss of Lonrf2 causes late-onset neurological deficits, as evidenced by a reduction in the number of ChAT-positive neurons and accumulation of p-TDP43 aggregates in neurons in the spinal cord, cerebral cortex and cerebellum *(**9**)*. However, the pathophysiological context of other LONRF family proteins remains unknown. In this study, we analysed the expression of Lonrf1 and the transcriptomic features of Lonrf1-expressing cells at the single-cell level. We found that Lonrf1, in contrast to Lonrf2, is ubiquitously expressed in various tissues and cell types and is likely to promote tissue remodelling during the wound healing process.

## Materials and Methods

### ProteoStat staining

Cells on CELLview™ Sterile Glass Bottom Dishes (Greiner Bio-One) were fixed in 4% paraformaldehyde for 10 min at room temperature, washed with PBS (phosphate buffered saline), and permeabilized with 0.2% TritonX-100 in PBS for 5 min. Samples were then stained with 1/2000X diluted PROTEOSTAT® Aggresome Detection Reagent (Enzo Life Sciences) according to the manufacturer’s protocol, and nuclei were counterstained with Hoechst 33342 (1 mg/ml) (Dojindo) for 30 min at room temperature in the dark. After extensive washing with PBS, images were acquired by BZ-9000 (KEYENCE). As a positive control, the proteasome inhibitor MG132 (Sigma-Aldrich) was used at a concentration of 5 mM for 12 h.

### Quantitative- polymerase chain reaction (qPCR)

qPCR was performed as previously described *(**9**)*. An MTC Mouse Panel (Takara) was used for cDNA from mouse tissues. Real-time PCR amplifications were performed in 96-well optical reaction plates using Thunderbird SYBR qPCR Master Mix (TOYOBO) from QuantStudio3 (Applied Biosystems). Relative expression values of each gene was determined by normalization to Gapdh expression for each sample. Primer sequences were as follows:

Mouse Lonrf1-forward primer: CTCTCAAAATCTACAGAGCGGAG.

Mouse Lonrf1-reverse primer: AAGCTGAAAGAGAACTGCGTT.

Mouse Gapdh-forward primer: CATCACTGCCACCCAGAAGACTG.

Mouse Gapdh-reverse primer: ATGCCAGTGAGCTTCCCGTTCAG.

**Fig. 1:**
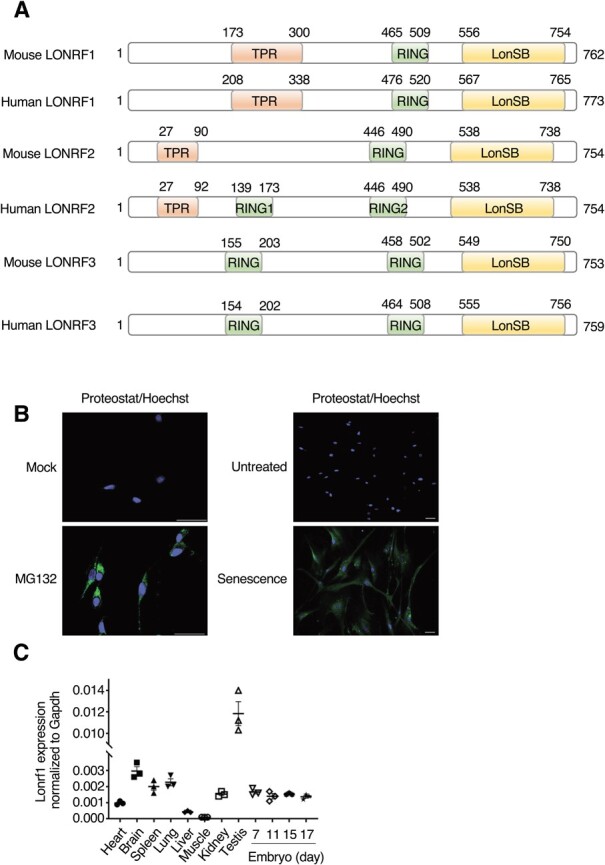
**Lonrf1 is ubiquitously expressed in mouse tissues.** (A) Structure of LONRF proteins from *Mus musculus* (mouse) and *Homo sapiens* (human). Domain structures are indicated. TPR is a protein–protein interaction domain, the RING finger is a domain that binds ubiquitination enzymes and their substrates and functions as a ligase, and the LonSB is associated with the turnover of misfolded or denatured proteins. (B) ProteoStat staining of HCA2 cells treated with (MG132) or without (mock) MG132 (5 mM) for 12 h. Representative images (scale bar: 50 mm) and quantifications (%) are shown (left panels). ProteoStat staining of HCA2 cells treated with (Senescence) or without (Untreated) doxorubicin for 24 h and then with BI2536 for 48 h. (right panels). Green: ProteoStat, blue: Hoechst. (C) qPCR analysis with the indicated primers using a panel of RNAs from mouse tissues. The relative expression of Lonrf1 to Gapdh is shown. Data are presented as mean ± SEM of independent experiments. *n* = 3

### Wound healing experiments

All animals were handled in accordance with the Guidelines for Animal Experiments of the Institute of Medical Science, the University of Tokyo and the Institutional Laboratory Animal Care. Six-week-old p16-Cre^ERT2^-tdTomato mice were given a single dose of analgesic on the day before surgery. Mice were anesthetized, hairs on the lower back were removed with a shaver and two holes were made in the skin approximately 2 cm from the tail using a 6-mm biopsy punch (KAI). Injured mice were kept in separate cages, one per mouse. Tamoxifen (TAM) (80 mg/kg BW, Sigma-Aldrich) was administrated from day 1 to day 5 after injury. Mice were sacrificed 7 d after surgery. For fluorescence cytochemistry, mouse skin tissues were fixed with 4% paraformaldehyde phosphate buffer solution overnight at 4°C, then replaced with 30% sucrose, embedded by OCT(optimal cutting temperature), and subjected to sectioning. After drying the sections with a hair dryer for 15 min, the sections were washed with PBS for 5 min. The tissues were incubated with Hoechst 33342 solution (Thermo) for 60 min under light shade and then washed twice for 5 min with PBS. Sections were analysed and photographed using an IN Cell Analyzer 2500HS (Cytiva). For single-cell RNA sequencing, skin tissue was incubated in DMEM containing 1.75 mg/ml collagenase IV (Gibco, 9001–12-1) and 12.5 kU DNase (Sigma, D4263) at 37°C for 90 min. Tom^high^ and Tom^low^ cells were isolated using FACS Aria II (BD Biosciences).

**Fig. 2:**
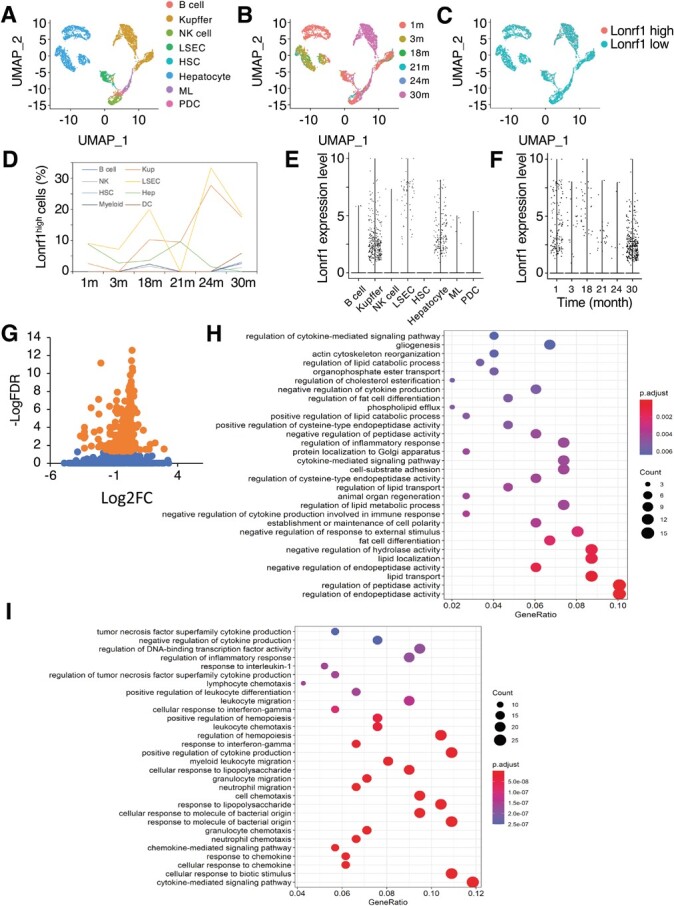
**scRNA analysis of Lonrf1 in liver from the Tabula Muris dataset.** (A) UMAP visualization showing cell types in the Tabula Muris liver scRNA dataset containing 6,480 transcriptomes, including B cell, Kupffer cell, NK cell, liver sinusoidal endothelial cell (LSEC), HSC, hepatocyte, ML and PDC. (B) UMAP visualization showing the age of the mouse sample. (C) UMAP visualization showing the distribution of Lonrf1^high^ and Lonrf1^low^ cells in scRNA dataset. The threshold of Lonrf1-normalized UMI count used to determine high and low was 0.2. (D) Line graph showing the age-dependent changes in the percentage of Lonrf1^high^ cells in each cell type. (E) Violin plot showing the Lonrf1 expression in each cell type. The expression level is represented by normalized UMI counts. (F) Violin plot showing the Lonrf1 expression in each age of the samples in all cell types. (G) Volcano plot showing the DEGs between Lonrf1^high^ and Lonrf1^low^ Kupffer cells in all the time points of mice. DEGs were identified by FDR < 0.05. Orange dots: significantly up-regulated or down-regulated DEGs. (H) Dot plot showing the top 30 enriched GO terms of up-regulated DEGs in Lonrf1^high^ vs. Lonrf1^low^ Kupffer cells. GO terms were identified with adjusted *P*-value<0.05 through B-H method. (I) Dot plot showing the top 30 enriched GO terms of downregulated DEGs in Lonrf1^high^ compared with Lonrf1^low^ Kupffer cells.

### Single cell transcriptome analysis

For the Tabula Muris dataset, the h5ad files were downloaded from https://tabula-muris.ds.czbiohub.org/ and fully processed with normalization, clustering and cell type identification. The scRNA datasets containing whole cells and Tom^high^ of normal liver and nonalcoholic steatohepatitis (NASH) liver from 7-mo-old p16-Tom mice were obtained from GEO (GSE155182) *(**10**)*. The datasets of whole cells and Tom^high^ cells from identical mice were directly combined using the merge function in the Seurat package (v4.0.2) *(**11**)*. A total of 7,689 and 11,476 transcriptomes were obtained from normal liver and NASH liver, respectively. The doublets and cells with low sequencing quality or containing high levels of mitochondrial gene content were excluded from further analysis (thresholds: 800 < genes < 6,000, unique molecular identifier (UMI) counts < 16,000, mitochondrial gene ratio < 10%). The average UMI counts were 7,295.8 and 7,765.3 for the normal and NASH liver datasets, respectively. The transcriptomes were further normalized by log1p function and clustered with Leiden method. The clusters with cell number <10 were removed, and the cell types of each cluster were determined by marker genes. There were 5,199 and 8,508 cells from normal liver and NASH liver, respectively, included in further analysis.

**Fig. 3:**
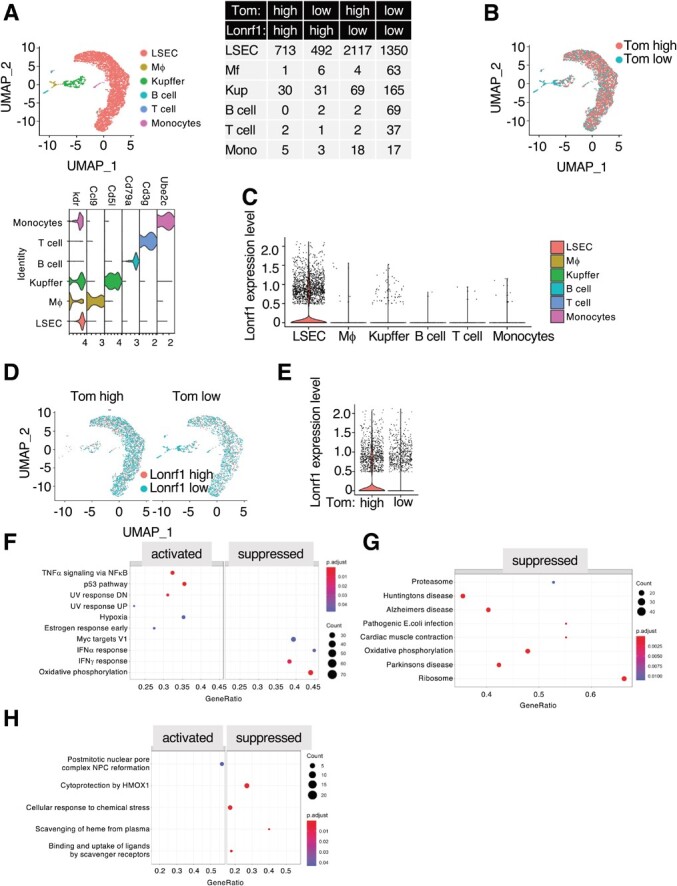
**scRNA analysis of Lonrf1 in the liver of 7-month-old p16-Tom mice.** (A) UMAP visualization (upper-left panel) showing cell types in liver from 7-month-old p16-Tom mice dataset containing 5199 transcriptomes, including LSEC, macrophage (Mf), Kupffer cell, B cell, T cell, and monocyte. Violin plot showing the expression levels of cell type markers in each cell type (lower-left panel). The number of cells in each group is shown in the table (right panel). The thresholds for Lonrf1 and Tom expression levels were 0.2. (B) UMAP visualization showing the of distribution of Tom^high^ and Tom^low^ cells. (C) Violin plot showing the Lonrf1 expression in each cell type. The expression level is represented by normalized UMI counts. (D) UMAP visualization showing the distribution of Lonrf1^high^ and Lonrf1^low^ cells in Tom^high^ (left panel) and Tom^low^ cells (right panel), respectively. (E) Violin plot showing the Lonrf1 expression in Tom^high^ and Tom^low^ cells in all cell types. (F) Dot plot showing the enriched GSEA terms from the Hallmark database in Lonrf1^high^/Tom^low^ compared with Lonrf1^low^/Tom^low^ LSECs. GSEA terms were identified with adjusted *P* < 0.05 by B-H method. (G) Dot plot showing the enriched GSEA terms from KEGG database in Lonrf1^high^/Tom^low^ compared with Lonrf1^low^/Tom^low^ LSECs. (H) Dot plot showing the enriched GSEA terms from Reactome database in Lonrf1^high^/Tom^high^ compared with Lonrf1^low^/Tom^high^ LSECs.

**Fig. 4:**
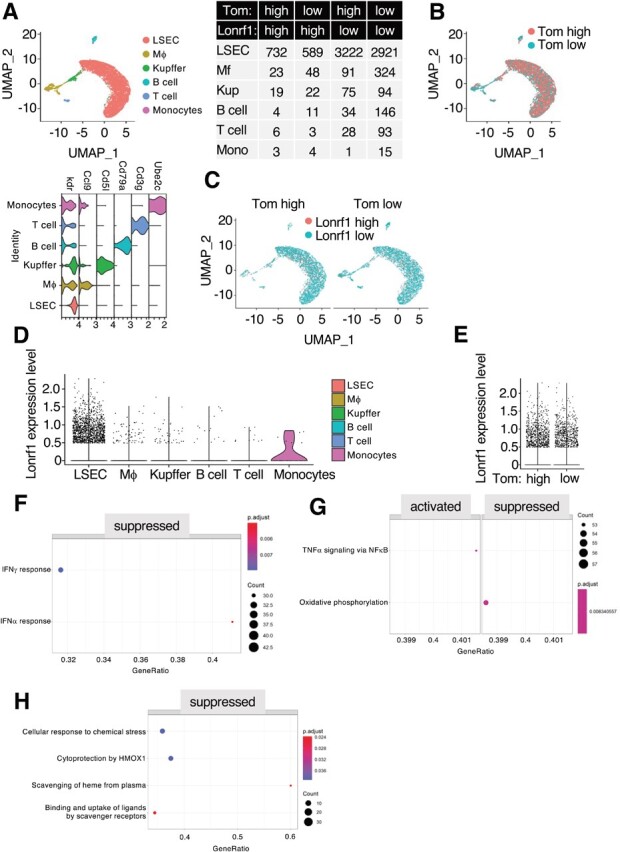
**scRNA analysis of Lonrf1 in NASH liver of 7-mo-old p16-Tom mice.** (A) UMAP visualization (upper-left panel) showing cell types in NASH liver from 7-mo-old p16-Tom mice dataset containing 8,508 transcriptomes. Violin plot showing the expression levels of cell-type markers in each cell type (lower-left panel). The number of cells in each group is shown in the table (right panel). The thresholds for Lonrf1 and Tom expression levels were 0.2. (B) UMAP visualization showing the distribution of Tom^high^ and Tom^low^ cells. (C) UMAP visualization showing the distribution of Lonrf1^high^ and Lonrf1^low^ cells in Tom^high^ (left panel) and Tom^low^ cells (right panel), respectively. (D) Violin plot showing the Lonrf1 expression in each cell type. The expression level is represented by normalized UMI counts. (E) Violin plot showing the Lonrf1 expression in Tom^high^ and Tom^low^ cells in all cell types. (F) Dot plot showing the enriched GSEA terms from the Hallmark database in Lonrf1^high^/Tom^low^ compared with Lonrf1^low^/Tom^low^ LSECs. GSEA terms were identified with adjusted *P* < 0.05 by B-H method. (G) Dot plot showing the enriched GSEA terms from KEGG database in Lonrf1^high^/Tom^high^ compared with Lonrf1^low^/Tom^high^ LSECs. (H) Dot plot showing the enriched GSEA terms from Reactome database in Lonrf1^high^/Tom^high^ compared with Lonrf1^low^/Tom^high^ LSECs.

For the wound healing sample from 7-wk-old p16-Tom mice, the wound area of the skin was digested with 1.75 mg/ml collagenase IV (Gibco) and 12.5 kU/ml DNase I (Sigma) for 90 min in 37°C water bath. The cell suspension was washed twice with PBS, and then red blood cells were lysed with RBS lysis buffer (Thermo). Suspended cells were incubated with mouse FcR-blocking reagent (Miltenyi Biotec) and anti-CD45 for 30 min on ice. DAPI (4′,6-diamidino-2-phenylindole) staining was performed to label the dead cells, and then live CD45-negative cells were sorted by FACS Aria II (BD Bioscience). The whole cells and Tom^high^ cells were collected, and the single-cell RNA libraries were prepared using 10X Genomics Chromium Single Cell 30 Reagent Kit v3.1 (10X Genomics). The libraries were sequenced using the DNBSEQ-G400RS (MGI Tech). For sequence alignment, UMIs and barcodes were recognized, and transcripts were mapped to an mm10 mouse reference genome using a Cell Ranger package (ver. 3.0.2). The whole-cell and Tom^high^ datasets were merged using the merge function in the Seurat package (v4.0.2) *(**11**)*. A total of 11,413 transcriptomes were obtained from dermal tissues. The doublets and cells with low sequencing quality or containing high levels of mitochondrial gene content were excluded from further analysis (thresholds: 2,000 < genes < 8,000, UMI counts < 50,000, mitochondrial gene ratio < 10%). The average UMI count was then 19,907.9. The transcriptomes were further normalized by log1p function and clustered by Leiden method. The clusters with cell number <20 were removed, and the cell types of each cluster were decided by marker genes. There were 5,201 cells involved in further analysis. For all datasets, the threshold of Lonrf1 and Tom-normalized expression levels was 0.2. DEGs (differentially expressed genes) were identified by FindMarkers function with Wilcoxon rank sum test in Seurat package, and the significance was defined as FDR < 0.05. Gene ontology (GO) and gene set enrichment analysis (GSEA) were performed using clusterProfiler (v3.18.1) *(**12**)* and fgsea packages (v1.16.0) *(**13**)*, respectively. All significantly enriched terms were identified with adjusted *P* < 0.05. All GSEA terms were obtained from MSigDB.

**Fig. 5:**
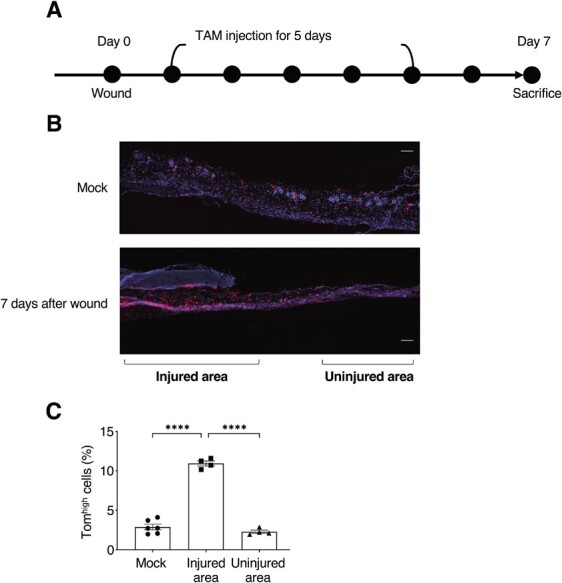
**Tom** ^**high**^ **cells specifically increased in the injured area after wounding.** (A) Experimental schedule of analysis during wound healing process. (B) Representative images of skin section 7 d after wounding or without wounding (mock). Injured and uninjured areas are indicated. Scale bar: 500 mm. (C) The proportion of Tom^high^ cells was determined by calculating Tom-positive cells/Hoechst-positive cells in mock, injured, and uninjured areas. Data are presented as mean ± SEM of independent experiments (*n* = 6, 4, 4). One-way ANOVA followed by Tukey HSD test was performed. ^****^P < 0.0001

### Statistical analysis

One-way analysis of variance (ANOVA) followed by Tukey HSD (honestly significant difference) test was performed to analyse the number of Tom^high^ cells from wound healing samples. We confirm that the data supporting the findings of this study are available within the article and its supplementary materials.

## Results and Discussion

### Lonrf1 is ubiquitously expressed in various tissues

In mammals, the LONRF family of proteins consists of three isozymes (LONRF1–3) containing RING domains and a LON substrate-binding domain (LonSB) (Figure 1A). Human LONRF2 contains two RING domains, whereas mouse LONRF2 contains only one RING domain. Recently, we found that LONRF2 selectively ubiquitylates misfolded proteins for proteasome-dependent degradation and maintains proteostasis. During senescence induction, when protein aggregates accumulate (Figure 1B), Lonrf2 expression significantly increased whereas Lonrf1 expression decreased, suggesting that the reduction of LONRF1 expression may be involved in the accumulation of protein aggregates in senescent cells *(**9**)*. Under unperturbed conditions, LONRF2 is predominantly expressed in the mouse brain. LONRF2^−/−^ mice show late-onset neurological deficits *(**9**)*. However, the physiological context of other LONRF isozymes remains unclear. To address this issue, we first determined the tissue expression patterns of Lonrf1. qPCR using mouse tissue cDNA revealed that Lonrf1 is ubiquitously expressed, with the highest expression in the testis (Figure 1C). These results suggest that LONRF1 may maintain protein homeostasis in many cell types and tissues, in contrast to the selective role of LONRF2 in neurons.

**Fig. 6:**
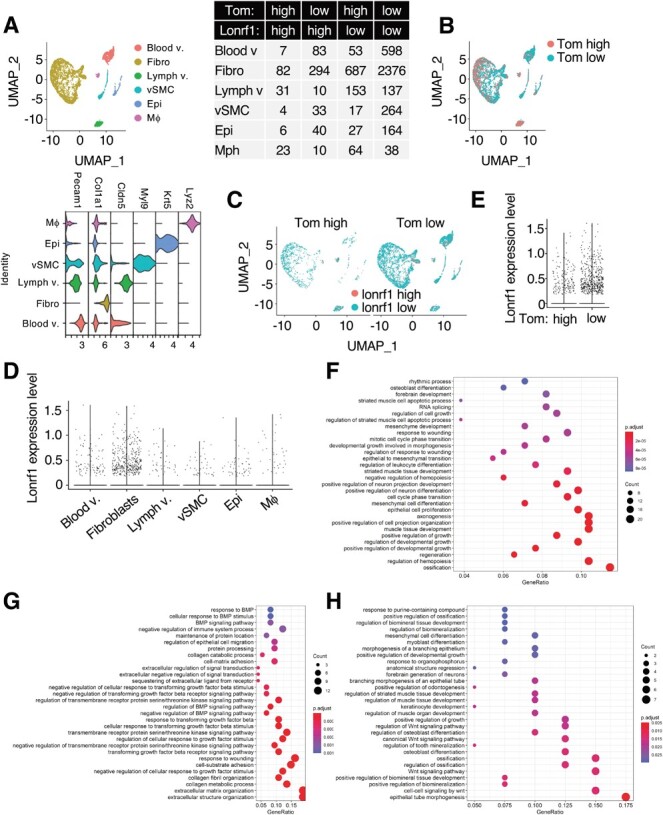
**scRNA analysis of Lonrf1 in the skin of p16-Tom mice during wound healing process.** (A) UMAP visualization (upper-left panel) showing cell types in 7-wk-old skin from p16-Tom mice dataset containing 5,201 transcriptomes, including blood vessel endothelial cell (Blood v.), fibroblast (Fibro), lymph vessel endothelial cell (Lymph v.), vascular smooth muscle cell (vSMC), epithelial cell (Epi), and macrophage (Mf). Violin plot showing the expression levels of cell-type markers in each cell type (lower-left panel). The number of cells in each group is shown in the table (right panel). The thresholds for Lonrf1 and Tom expression levels were 0.2. (B) UMAP visualization showing the distribution Tom^high^ and Tom^low^ cells. (C) UMAP visualization showing the distribution of Lonrf1^high^ and Lonrf1^low^ cells in Tom^high^ (left panel) and Tom^low^ cells (right panel), respectively. (D) Violin plot showing the Lonrf1 expression in each cell type. The expression level is represented by normalized UMI counts. (E) Violin plot showing the Lonrf1 expression in Tom^high^ and Tom^low^ cells in all cell types. (F) Dot plot showing the top 30 enriched GO terms of upregulated DEGs in Lonrf1^high^/Tom^low^ compared with Lonrf1^low^/Tom^low^ fibroblasts. GO terms were identified with adjusted *P* < 0.05 by B-H method. (G) Dotplot showing the top 30 enriched GO terms of downregulated DEGs in Lonrf1^high^/Tom^low^ compared with Lonrf1^low^/Tom^low^ fibroblasts. (H) Dot plot showing the top 30 enriched GO terms of upregulated DEGs in Lonrf1^high^/Tom^high^ compared with Lonrf1^low^/Tom^high^ fibroblasts.

### scRNA-seq analysis of Lonrf1 during ageing

Because LONRF1 may function differently from LONRF2 in senescent cells, we first investigated the role of LONRF1 in the ageing process. Among many external environmental stimuli, oxidative stress is one of the major factors in ageing that disrupts protein homeostasis by promoting misfolding and aggregate formation of various proteins *(**14**)*. The liver is the center of metabolism in the body and produces a large amount of reactive oxidative species (ROS) during its various metabolic processes *(**15**)*. Therefore, the liver has the most advanced antioxidant mechanism, and molecules involved in protein homeostasis are likely to play an important role in maintaining proper liver function. To determine which cell types express Lonrf1 in the liver and how the expression patterns change during ageing, we analysed Lonrf1 expression in its transcriptomics from the liver using a single-cell transcriptome dataset, Tabula Muris *(**16**)*. After unsupervised graph-based clustering, eight cell types including B cells, Kupffer cells, natural killer cell (NK cells), hepatic sinusoidal endothelial cells (LSEC), hepatic stellate cells (HSCs), hepatocytes, myeloid leukocytes (ML) and plasmacytoid dendritic cells (PDC) displayed on UMAP (uniform manifold approximation and projection) were identified based on the pattern fitness between the top 100 cluster rank genes and a cell-type gene list of a reported scRNA-seq dataset (Figure 2A and 2B). Lonrf1 is mainly expressed in Kupffer cells, LSEC and hepatocytes (Figure 2C-2E). Interestingly, the number of Lonrf1 high-expressing cells (Lonrf1^high^) tended to increase with age in Kupffer cells and LSEC, but not hepatocytes, suggesting that LONRF1 may play a role in ageing in these nonparenchymal cells (Figure 2D and 2F). To further infer the function of LONRF1, we identified the DEGs between Lonrf1^high^ and Lonrf1^low^ Kupffer cells at all ages (230 upregulated and 163 downregulated DEGs) (Figure 2G and Supplementary Table S1). We also attempted to identify DEGs between Lonrf1^high^ and Lonrf1^low^ LSECs, but were unable to do so, possibly due to the small number of cells. GO analysis revealed the significant enrichment of terms related to the regulation of various peptidase activities in Lonrf1^high^ Kupffer cells (Figure 2H). Kupffer cells are tissue-resident macrophages in the liver, and their primary role is to phagocytose insoluble macromolecules, immune complexes and other foreign substances in the circulating blood, and higher peptidase activity is critical for efficient phagocytosis *(**17**)*. Further experiments will be important to investigate the role of LONRF1 in the phagocytic capacity of Kupffer cells. Furthermore, GO analysis of Lonrf1^high^ Kupffer cells significantly enriched for terms related to lipid metabolism such as fat differentiation and lipid transport. It has been reported that peroxisome proliferator-activated receptor delta (PPARd) and peroxisome proliferator-activated receptor gamma (PPARg), two nuclear receptor family members important for the regulation of lipid metabolism, shift the lipid-induced macrophage/Kupffer cell polarization from an inflammatory phenotype to an anti-inflammatory phenotype by increasing the expression of various antioxidant genes and decreasing the synthesis of proinflammatory mediators *(18–20)*. Consistent with these reports, inflammation-related terms such as cytokine-mediated signalling pathway and chemotaxis were significantly enriched in the GO analysis of Lonrf1^low^ Kupffer cells (Figure 2I). Further experiments are required to address whether PPARd and PPARg directly regulate the transcription of LONRF1 in Kupffer cells and whether LORF1 plays a role in the antioxidant responses and anti-inflammatory responses mediated by these transcriptional regulators.

### scRNA-seq analysis of Lonrf1 in normal and NASH livers

We then investigated whether LONRF1 might be involved in a specific pathogenesis. Nonalcoholic fatty liver disease (NAFLD) is a growing chronic liver disease worldwide. In particular, patients with NAFLD develop NASH, which has a poor prognosis and often leads to oxidative stress, liver fibrosis and, eventually, cirrhosis and hepatocellular carcinoma *(**21**)*. Cellular senescence is induced by various factors, including oxidative stress, and is characterized by the expression of cell cycle inhibitors such as p16 and the secretion of bioactive molecules, the so-called SASP *(**22**)*. Recently, we have shown that senescent cells accumulate in the liver of NASH-induced mice and that elimination of senescent cells by genetic or pharmacological approaches ameliorates the pathogenesis of NASH *(**10**,**23**,**24**)*. To infer the role of LONRF1 in cellular senescence, we examined scRNA-seq datasets of LSECs, a rich set of cell types, in normal and NASH livers of the p16-Cre^ERT2^-tdTomato (p16-Tom) mouse model *(**10**)* (Figure 3A-3C). tdTomato-high (Tom^high^) and tdTomato-low (Tom^low^) expressing LSECs were distinguished as Lonrf1^high^ and Lonrf1^low^ based on the presence of LONRF1 transcript, and it was found that there are comparable numbers of Lonrf1^high^ cells in Tom^high^ and Tom^low^ LSECs, consistent with a decrease in Lonrf1 expression during cellular senescence *in vitro (**9**)*. DEGs in Lonrf1^high^ and Lonrf1^low^ LSECs were identified separately in Tom^high^ and Tom^low^ cells (Figure 3D and Supplementary Tables S2 and S3). Recently, we reported that senescent cells heterogeneously express PD-L1, and GO analysis of PD-L1^+^ senescent cells showed the enrichment of terms related to cytokine responses and inflammatory signalling as compared with PD-L1^−^ senescent cells using the identical datasets for LSECs *(**24**)*. We found that the transcriptomes of Tom^high^ and Tom^low^ LSECs from normal liver were not perturbed by Lonrf1 expression levels (only 4 DEGs in Tom^low^, 16 DEGs in Tom^high^ and 0 enriched terms in GO analysis) (Figure 3E). These results suggest that LONRF1 plays a minimal role in senescence phenotypes including SASP. GSEA of Hallmark and KEGG gene sets revealed activation of NF-kB and p53 signalling, and the suppression of IFNa, IFNg, and proteasome signalling, and pathways related to neuronal degenerative disorders such as Huntington’s, Alzheimer’s, and Parkinson’s diseases in Lonrf1^high^/Tom^low^ cells (Figure 3F and 3G). Reactome gene sets revealed the suppression of cytoprotection by HMOX1 in Lonrf1^high^/Tom^high^ LSECs from normal liver (Figure 3H). HMOX1 is the gene encoding the primary antioxidant enzyme involved in heme group degradation regulated by the KAEP1-NRF2 system under oxidative stress *(**25**)*. These results suggest that LONRF1 may play a beneficial role in the defense against oxidative stress. In NASH liver, we also observed similar transcriptomes and ratios between Lonrf1^high^ and Lonrf1^low^ cells (Figure 4A-4E, and Supplementary Tables S4 and S5), suggesting that Lonrf1 expression may not be related to NASH pathogenesis. GSEA analysis also revealed the suppression of IFNa and IFNg in Lonrf1^high^/Tom^low^ cells (Figure 4F). In addition, activation of the NF-kB pathway and suppression of HMOX1 were also detected in Lonrf1^high^/Tom^high^ cells (Figure 4G and 4H).

### scRNA-seq analysis of Lonrf1 during wound healing process

Because senescent cells have been proposed to have both deleterious and beneficial effects under physiological conditions, we expected that LONRF1 might be involved in the beneficial effects of senescent cells. Therefore, we analysed Lonrf1 expression and transcriptomics during the wound healing process where senescent cells have tissue regenerative activity *(**26**)*.

Six-week-old male p16-Tom mice were wounded with a biopsy punch, injected daily with TAM for the next 5 d, and sacrificed 7 d after wounding (Figure 5A). As reported previously, the number of p16^high^ cells increased significantly in the injured but not in the uninjured area (Figure 5B and 5C). Two different cell sorting approaches were used to perform scRNA-seq of dermal cells. To assess the size of the Tom^high^ population in each cell type, unseparated cells were collected directly. However, because the *in vivo* conditions resulted in an extremely low proportion of senescent cells, Tom^high^ cells were also sorted to enrich the cell numbers for further analysis.

Based on the well-established marker genes and unsupervised clustering, dermal cells were classified into seven cell types including blood vessel endothelial cells (Blood v.), fibroblasts (Fibro), lymph vessel endothelial cells (Lymph v.), vascular smooth muscle cells (vSMC), epithelial cells (Epi) and macrophages (Mf) (Figure 6A). Tom^high^ and Tom^low^ were distinguished by fluorescence using FACS analysis (Figure 6B). Among these cell types, Lonrf1-expressing cells were abundant in fibroblasts (Figure 6C and 6D). Again, the Lonrf1 expression and population of Lonrf1^high^ cells was comparable between Tom^high^ (Lonrf1^high^: 13.2%) and Tom^low^ cells (Lonrf1^high^: 11.6%) (Figure 6A and 6E), suggesting that Lonrf1 may not be involved in senescence induction during the wound healing process. GO analysis in Lonrf1^high^/Tom^low^ fibroblasts revealed activation of cell growth-related terms and suppression of TGFb, BMP signalling and extracellular matrix organization (Figure 6F and 6G and Supplementary Table S6 and S7). GO analysis in Lonrf1^high^/Tom^high^ fibroblasts revealed activation of WNT signalling and epithelial development-related terms (Figure 6H). These results suggest that Lonrf1 may be involved in the proliferation and differentiation of myofibroblasts, which have been reported to be activated by oxidative stress *(**27**)*. In addition, activation of WNT signalling in Lonrf1^high^/Tom^high^ cells may promote the regeneration of epithelial cells such as keratinocytes *(*28–30*)*.

In conclusion, Lonrf1 expression does not seem to be associated with senescence induction and phenotypes in the different cell types analysed. However, the transcriptomic approach reveals that Lonrf1^low^ cells show higher oxidative stress responses, suggesting a benefit role of Lonrf1 in antioxidant and tissue remodelling. Further functional analysis using Lonrf1-deficient mice will be required to draw clear conclusions in this regard.

## Supplementary Material

Web_Material_mvad021
