# Supplementary material for: Transcriptomic characterization of Lonrf1 at the single-cell level under pathophysiological conditions
Source: J Biochem. 2023 Mar 8;173(6):459–69. doi: 10.1093/jb/mvad021 (PMC10226518; doi:10.1093/jb/mvad021)
Supplement: Web_Material_mvad021 [file web_material_mvad021.zip › Supplementary Table S1.pdf]

Supplementary Table S1

DEG LonFR1+vsLonRF1- in Kupffer from Tabula Muris liver

	p_val	avg_log2F(	pct.1	pct.2	p_val_adj
Thap6	1.29E-06	2.947162	0.148	0.069	0.025647
Loxl3	2.80E-07	2.248169	0.267	0.145	0.005563
Fnip2	4.01E-07	1.869377	0.312	0.181	0.007967
Rnf144b	1.41E-06	1.790711	0.379	0.235	0.028043
Zfp667	1.46E-06	1.501014	0.206	0.108	0.02907
Cd274	1.08E-08	1.326048	0.609	0.404	0.000214
Fbxo25	1.71E-06	1.074318	0.188	0.096	0.033913
Zfp703	2.63E-09	0.910605	0.779	0.576	5.23E-05
Niacr1	1.24E-10	0.833684	0.679	0.481	2.46E-06
Csnk2a2	5.42E-09	0.786762	0.173	0.071	0.000108
Csprs	8.95E-07	0.743838	0.636	0.447	0.017777
Kcnj8	1.28E-06	0.670663	0.133	0.06	0.025332
Arc	2.84E-07	0.668279	0.315	0.183	0.005642
Parp11	4.15E-07	0.663727	0.345	0.201	0.00825
R74862	4.30E-11	0.61953	0.709	0.486	8.53E-07
Tnfaip3	4.77E-13	0.59495	0.785	0.533	9.47E-09
Atg2a	3.09E-08	0.578226	0.427	0.262	0.000613
AI606473	1.82E-12	0.577589	0.648	0.418	3.61E-08
Fam168a	3.65E-07	0.542573	0.385	0.234	0.007245
Tnfaip2	2.05E-07	0.518393	0.918	0.797	0.004071
Gpr132	6.83E-09	0.500122	0.327	0.18	0.000136
Fam105b	2.63E-09	0.49309	0.788	0.575	5.23E-05
Cxcl2	2.84E-15	0.49235	0.858	0.628	5.63E-11
Ifit1	3.50E-07	0.47584	0.433	0.279	0.00696
Cxcl13	5.19E-13	0.475723	0.912	0.736	1.03E-08
Fem1b	1.40E-06	0.473833	0.424	0.268	0.02787
Mon2	1.73E-06	0.469035	0.318	0.189	0.034329
Phf15	3.05E-07	0.465711	0.412	0.248	0.006057
Tiparp	1.26E-15	0.46087	0.788	0.539	2.50E-11
Gdf15	1.40E-17	0.45397	0.861	0.619	2.79E-13
Nlrc4	4.85E-07	0.453779	0.288	0.164	0.009632
Bcl7a	1.42E-06	0.447852	0.191	0.099	0.028272
Map3k8	1.07E-06	0.446359	0.761	0.583	0.021344
Gbp4	6.56E-08	0.445287	0.458	0.287	0.001302
Mmp14	4.83E-07	0.442502	0.43	0.281	0.009601
4933426M	5.91E-08	0.431887	0.77	0.561	0.001173
Atp1b3	2.22E-08	0.428739	0.961	0.852	0.000441
Cd83	9.48E-13	0.422903	0.888	0.684	1.88E-08
Tob2	2.04E-10	0.42179	0.806	0.604	4.05E-06
Basp1	2.20E-11	0.415762	0.933	0.8	4.37E-07

Plek	1.01E-10	0.414355	0.915	0.724	2.01E-06
Ccl2	9.55E-13	0.410066	0.876	0.697	1.90E-08
Fosb	1.96E-16	0.407177	0.797	0.557	3.89E-12
Il10	2.84E-08	0.405694	0.482	0.318	0.000565
Myo10	1.79E-08	0.402221	0.561	0.369	0.000355
Mthfd2	1.95E-07	0.390652	0.548	0.377	0.003864
Mxd1	4.28E-14	0.385046	0.679	0.432	8.51E-10
Tmem140	1.42E-06	0.378251	0.642	0.451	0.028273
Cdkn1a	6.26E-13	0.378217	0.797	0.572	1.24E-08
Gse1	5.22E-14	0.376316	0.373	0.175	1.04E-09
Tbc1d13	8.16E-08	0.374326	0.452	0.274	0.00162
Plk2	5.31E-07	0.369088	0.412	0.28	0.010537
Bhlhe40	1.34E-13	0.366581	0.694	0.448	2.66E-09
Rilpl2	2.36E-10	0.361609	0.897	0.72	4.68E-06
Nfkbiz	2.90E-12	0.356776	0.848	0.642	5.76E-08
Ppp1r15a	1.07E-11	0.355429	0.821	0.597	2.13E-07
Pparg	1.75E-06	0.351624	0.397	0.252	0.034819
Lhx8	2.29E-06	0.351541	0.261	0.148	0.045462
Pf4	2.75E-09	0.349704	0.924	0.825	5.47E-05
Dclre1c	1.53E-06	0.347742	0.464	0.308	0.030442
Cox4i2	2.12E-06	0.347526	0.339	0.214	0.042021
Trem2	1.34E-08	0.337622	0.921	0.743	0.000267
Egr1	9.99E-09	0.336028	0.639	0.476	0.000198
Ptms	6.08E-12	0.332236	0.933	0.797	1.21E-07
Syne1	5.03E-09	0.330211	0.427	0.257	9.99E-05
Ccnd2	9.99E-13	0.328018	0.809	0.579	1.98E-08
Ccl7	1.82E-10	0.324307	0.824	0.635	3.61E-06
Med20	1.27E-07	0.323782	0.252	0.13	0.002515
2310047M	1.13E-06	0.320209	0.594	0.398	0.022438
Ddit3	3.01E-09	0.319665	0.752	0.532	5.97E-05
Lgals1	4.05E-12	0.318044	0.858	0.678	8.05E-08
Ccl4	9.72E-14	0.316509	0.912	0.688	1.93E-09
Cd207	2.90E-07	0.315229	0.912	0.769	0.005761
Pim3	7.79E-12	0.314074	0.764	0.537	1.55E-07
Hilpda	8.08E-15	0.313559	0.909	0.699	1.60E-10
2410006H	8.25E-10	0.312095	0.909	0.749	1.64E-05
Junb	1.51E-11	0.309411	0.979	0.835	3.00E-07
Cd93	2.40E-06	0.303785	0.215	0.119	0.047662
Fam98c	1.14E-07	0.302033	0.464	0.292	0.002257
Capg	1.59E-07	0.301351	0.912	0.791	0.003148
Cxcl10	5.11E-10	0.297195	0.718	0.511	1.02E-05
Tnrc6b	8.77E-11	0.289972	0.561	0.344	1.74E-06
Mt1	8.56E-13	0.287869	0.961	0.895	1.70E-08

B4galt5	3.81E-09	0.287799	0.585	0.388	7.56E-05
Pmaip1	4.68E-09	0.286961	0.8	0.577	9.30E-05
Csrnp1	1.94E-12	0.286337	0.748	0.504	3.85E-08
Il2rg	3.08E-09	0.283539	0.87	0.643	6.12E-05
Zfp36	2.38E-11	0.282928	0.958	0.845	4.73E-07
Odc1	2.08E-08	0.282488	0.745	0.571	0.000413
Metrnl	4.79E-08	0.281715	0.936	0.802	0.000951
C130026I2	1.64E-08	0.281261	0.824	0.61	0.000325
Klf4	6.29E-13	0.280489	0.742	0.521	1.25E-08
Sp100	3.73E-08	0.280071	0.691	0.479	0.000741
Ier3	4.21E-13	0.27965	0.867	0.644	8.35E-09
Nr4a1	2.53E-10	0.277328	0.585	0.377	5.02E-06
Trim25	6.81E-09	0.276298	0.891	0.702	0.000135
Gins4	4.63E-08	0.273882	0.576	0.369	0.00092
Sorl1	1.88E-08	0.27186	0.158	0.066	0.000374
LOC54526:	2.10E-11	0.271847	0.533	0.322	4.17E-07
Jund	7.01E-10	0.2711	0.973	0.909	1.39E-05
Fos	2.99E-10	0.27107	0.958	0.859	5.95E-06
Per1	3.52E-08	0.26861	0.6	0.404	0.000698
Slc25a28	1.48E-07	0.267143	0.512	0.34	0.002933
Gadd45b	1.56E-10	0.261019	0.764	0.525	3.11E-06
Eng	9.02E-07	0.257395	0.406	0.259	0.017911
Id3	1.51E-11	0.256188	0.997	0.946	3.00E-07
Ezh2	4.65E-08	0.255908	0.418	0.258	0.000924
Ctdnep1	8.16E-10	0.25469	0.773	0.544	1.62E-05
Dgat1	1.96E-09	0.254593	0.4	0.228	3.89E-05
Atf4	9.26E-10	0.253776	0.867	0.709	1.84E-05
Ccl9	3.53E-07	0.253367	0.83	0.652	0.007003
H2-Eb1	2.18E-07	0.252963	0.979	0.913	0.004332
Mt2	6.07E-10	0.25281	0.776	0.579	1.20E-05
Socs3	1.44E-10	0.252791	0.558	0.366	2.85E-06
Ccnl1	4.83E-09	0.25169	0.827	0.651	9.59E-05
Id2	2.60E-08	0.251569	0.979	0.898	0.000516
Herpud1	1.60E-10	0.249392	0.882	0.663	3.17E-06
Ccl8	5.57E-10	0.249299	0.788	0.58	1.11E-05
Nfkbia	2.41E-08	0.248561	0.982	0.899	0.00048
Rpl37	2.27E-09	0.244651	1	1	4.50E-05
Ccl12	1.40E-07	0.244106	0.655	0.454	0.00279
Clic4	5.65E-09	0.24392	0.803	0.603	0.000112
Atf3	1.66E-13	0.243772	0.848	0.617	3.29E-09
1500012F(	6.44E-07	0.242844	0.833	0.665	0.012789
Gas6	1.46E-09	0.241442	0.821	0.596	2.90E-05
H2-Ab1	2.78E-07	0.236138	0.994	0.95	0.00552

Cd36	2.84E-08	0.235265	0.924	0.758	0.000563
Efhd2	5.28E-07	0.234797	0.948	0.864	0.010487
1500032L2	3.88E-07	0.233949	0.97	0.927	0.007706
Pim1	3.84E-09	0.231867	0.83	0.632	7.63E-05
Ifrd1	3.47E-10	0.231849	0.864	0.644	6.90E-06
Rps14	5.75E-12	0.23093	1	1	1.14E-07
H2-Aa	1.56E-06	0.227641	0.991	0.944	0.031051
Eps8	3.28E-07	0.224255	0.3	0.17	0.006514
Btg2	6.74E-08	0.221131	0.952	0.816	0.001338
Fosl2	3.00E-09	0.220616	0.77	0.549	5.96E-05
Il1b	6.30E-11	0.2202	0.961	0.811	1.25E-06
Vps4a	1.29E-06	0.220148	0.567	0.387	0.025585
Fundc2	4.95E-07	0.216282	0.852	0.67	0.00983
Cwc25	1.24E-09	0.21562	0.388	0.214	2.46E-05
Srgn	6.16E-08	0.213398	0.976	0.905	0.001224
Stat3	1.56E-07	0.2089	0.758	0.546	0.0031
Zc3h12a	6.53E-10	0.205833	0.6	0.388	1.30E-05
Clec7a	6.39E-10	0.205727	0.958	0.864	1.27E-05
Tmsb10	6.30E-08	0.205657	0.961	0.884	0.001251
Eif4ebp1	1.79E-06	0.205062	0.909	0.791	0.03549
Cd74	1.43E-06	0.20488	1	0.987	0.02837
Slc15a2	2.52E-10	0.204286	0.488	0.298	5.00E-06
Cacna1f	2.08E-06	0.203735	0.752	0.558	0.041407
Ccnt1	1.68E-09	0.201614	0.324	0.176	3.34E-05
Sat1	5.62E-07	0.201528	0.994	0.988	0.011152
Cd82	2.07E-08	0.201097	0.761	0.541	0.000412
Irf8	1.69E-07	0.197969	0.952	0.833	0.003351
Adamts1	3.00E-09	0.19683	0.215	0.098	5.96E-05
Pcbp2	1.38E-06	0.196767	0.988	0.919	0.027357
Sdc4	3.03E-08	0.196686	0.727	0.54	0.000602
Itgam	4.88E-09	0.192854	0.73	0.51	9.70E-05
Tgif1	1.64E-07	0.192704	0.876	0.692	0.003261
Mpst	1.17E-06	0.191951	0.467	0.307	0.023181
Rpl32	1.77E-10	0.191225	0.997	1	3.52E-06
Vim	7.57E-07	0.189311	0.882	0.738	0.015035
Gda	1.25E-06	0.183427	0.367	0.237	0.024753
Hpgds	2.42E-07	0.183075	0.797	0.596	0.00481
Fxyd2	2.54E-07	0.181429	0.715	0.533	0.005038
Chmp4b	7.00E-08	0.177498	0.985	0.906	0.00139
Il21r	1.61E-06	0.177173	0.327	0.199	0.031961
H3f3b	2.78E-07	0.17512	1	0.991	0.005517
Maff	2.41E-10	0.174986	0.688	0.448	4.79E-06
Tspo	1.84E-06	0.174866	0.952	0.815	0.036505

Nt5dc3	8.88E-07	0.173326	0.206	0.105	0.017635
Dctn1	8.83E-11	0.173018	0.745	0.484	1.75E-06
Tspan32	1.76E-07	0.172636	0.579	0.382	0.003503
Apold1	4.08E-09	0.172619	0.218	0.102	8.10E-05
Man1a	4.89E-07	0.172591	0.306	0.178	0.009712
Slc43a3	4.00E-13	0.171098	0.388	0.194	7.94E-09
Sertad1	3.02E-08	0.170514	0.667	0.457	0.000599
Arhgef7	8.74E-08	0.16666	0.385	0.23	0.001736
Orc6	1.92E-06	0.166432	0.339	0.207	0.038057
Ets2	9.74E-10	0.164002	0.861	0.641	1.93E-05
Rpl10	5.07E-07	0.16365	1	0.999	0.010076
Pde4b	1.41E-08	0.163592	0.73	0.505	0.000281
Stmn1	1.28E-06	0.163396	0.506	0.351	0.02536
Kdm6b	2.22E-08	0.163372	0.767	0.552	0.00044
Slc2a1	1.62E-09	0.161989	0.485	0.292	3.22E-05
Rps4x	7.57E-08	0.161801	1	1	0.001504
Rnase6	8.28E-08	0.160127	0.73	0.525	0.001645
Csf2rb	2.32E-07	0.158406	0.873	0.713	0.004599
Ier5	1.81E-06	0.155892	0.912	0.708	0.035867
Hmgb2	8.89E-07	0.153921	0.87	0.692	0.017654
Procr	7.48E-07	0.152838	0.382	0.24	0.014865
Rnf19b	3.33E-07	0.152761	0.739	0.54	0.006615
Igbp1	1.51E-06	0.152514	0.833	0.661	0.030033
Atp6v0a1	3.72E-09	0.151677	0.603	0.419	7.38E-05
Cldn5	9.59E-07	0.150355	0.215	0.113	0.019038
Fcgr2b	1.31E-10	0.149792	0.77	0.557	2.60E-06
Uba52	6.98E-07	0.148042	0.997	1	0.013853
D6Wsu116	1.94E-07	0.147668	0.642	0.433	0.003855
Smc1a	3.37E-07	0.146805	0.733	0.526	0.006696
Rpl18a	2.27E-07	0.145614	1	1	0.004511
Ralgds	2.01E-07	0.142449	0.327	0.191	0.003988
Pfkfb3	9.92E-08	0.142103	0.379	0.231	0.001971
Avpi1	5.01E-11	0.140038	0.521	0.303	9.96E-07
Cytip	7.29E-08	0.138764	0.491	0.317	0.001448
Clec4n	1.95E-07	0.134498	0.958	0.874	0.003866
Actn4	1.01E-06	0.134271	0.682	0.484	0.020066
Cenpa	5.52E-09	0.134202	0.403	0.237	0.00011
Pfkp	2.23E-09	0.133954	0.458	0.274	4.42E-05
Sbno2	7.89E-08	0.12697	0.473	0.303	0.001568
Klf3	7.28E-07	0.126573	0.658	0.475	0.01446
Klf10	4.48E-08	0.124054	0.742	0.526	0.000891
Ankrd11	1.35E-08	0.121082	0.682	0.47	0.000267
Cxcr4	4.22E-11	0.119758	0.567	0.357	8.37E-07

Mapkapk3	2.16E-10	0.119541	0.4	0.221	4.29E-06
Esam	1.91E-06	0.117949	0.176	0.089	0.038027
Arl8b	1.40E-06	0.11757	0.858	0.642	0.027709
Dtnbp1	3.81E-07	0.114726	0.724	0.546	0.007563
2300009A(	2.01E-06	0.114614	0.645	0.452	0.039866
Coq10b	4.42E-08	0.111145	0.679	0.45	0.000878
Ryr1	3.73E-07	0.11096	0.721	0.531	0.007402
Mxi1	3.13E-08	0.110764	0.442	0.265	0.000623
Siah1a	1.13E-06	0.110261	0.245	0.133	0.022402
Il1rn	1.84E-06	0.109828	0.645	0.488	0.036513
Arhgap31	1.64E-08	0.108188	0.503	0.314	0.000326
Kars	2.80E-07	0.107952	0.621	0.414	0.005551
Ccdc109b	6.42E-07	0.106449	0.391	0.245	0.012753
Napsa	1.66E-06	0.10578	0.415	0.27	0.03293
Tbc1d10a	7.58E-08	0.105128	0.603	0.401	0.001504
Nrp2	4.02E-09	0.105075	0.355	0.197	7.98E-05
Slc11a2	8.97E-08	0.101942	0.548	0.355	0.001782
Appl2	2.86E-07	0.10106	0.355	0.21	0.00569
Stk4	2.31E-06	-0.10004	0.515	0.336	0.04597
Ankrd9	1.33E-08	-0.10124	0.136	0.053	0.000264
Tfeb	2.02E-06	-0.10273	0.4	0.254	0.040211
Npr1	7.49E-07	-0.12046	0.27	0.147	0.014866
Amz1	6.13E-12	-0.12423	0.545	0.321	1.22E-07
Chid1	8.06E-07	-0.12452	0.524	0.351	0.016006
Ahcyl2	2.83E-08	-0.12744	0.521	0.323	0.000561
Ptpn12	9.12E-07	-0.12867	0.527	0.344	0.018115
Ccl3	1.13E-10	-0.13232	0.855	0.638	2.25E-06
Fbxw11	5.18E-07	-0.13507	0.445	0.275	0.010295
Parvg	1.17E-07	-0.13692	0.612	0.408	0.002327
Pip5k1a	1.33E-06	-0.14715	0.215	0.111	0.026418
Bicd2	2.94E-09	-0.15071	0.336	0.177	5.84E-05
Zmiz1	9.91E-07	-0.15103	0.409	0.255	0.019681
Wwp2	9.09E-07	-0.15506	0.482	0.315	0.018044
Esm1	3.73E-08	-0.15536	0.155	0.066	0.00074
Slc28a2	1.07E-06	-0.16161	0.491	0.319	0.021344
Abhd8	1.13E-08	-0.16174	0.245	0.12	0.000225
Gbf1	2.37E-07	-0.16567	0.352	0.203	0.004715
Osbp	1.52E-07	-0.17294	0.297	0.161	0.003022
Yipf6	3.09E-09	-0.17492	0.536	0.333	6.13E-05
Mink1	1.03E-06	-0.17944	0.409	0.257	0.020461
Mul1	1.30E-06	-0.18175	0.27	0.152	0.02585
Chchd6	3.18E-07	-0.18306	0.397	0.251	0.006318
Nucb2	9.49E-07	-0.1833	0.673	0.472	0.018842

Laptm4b	2.36E-06	-0.18717	0.376	0.235	0.046863
Glg1	7.74E-07	-0.18806	0.552	0.361	0.015363
Pelo	2.03E-06	-0.18967	0.33	0.197	0.040308
Slc25a19	9.41E-09	-0.19116	0.379	0.215	0.000187
Nde1	1.83E-06	-0.19476	0.327	0.196	0.03636
Brd3	1.62E-06	-0.19584	0.388	0.238	0.032184
Zfp516	5.59E-07	-0.19664	0.367	0.214	0.011092
Zc3h18	4.12E-09	-0.19691	0.412	0.235	8.18E-05
Ahsg	7.85E-07	-0.21286	0.303	0.394	0.015595
C1qb	6.09E-09	-0.21479	0.997	0.993	0.000121
Agfg1	2.33E-06	-0.21692	0.397	0.248	0.046345
Skp2	5.07E-10	-0.21888	0.648	0.431	1.01E-05
Arfgef1	4.15E-07	-0.21925	0.445	0.273	0.008239
Tgfb3	2.15E-09	-0.23142	0.4	0.233	4.27E-05
Ms4a7	1.92E-07	-0.23545	0.882	0.707	0.003822
Vipar	1.23E-08	-0.2401	0.479	0.29	0.000244
Myo18a	1.75E-06	-0.24171	0.261	0.143	0.034703
Egr2	3.24E-07	-0.2476	0.339	0.202	0.006426
Zfp275	8.26E-07	-0.24852	0.145	0.065	0.016408
Gak	2.16E-08	-0.25463	0.555	0.348	0.000429
Pfn1	6.99E-07	-0.25718	1	1	0.013884
Lgmn	4.63E-08	-0.26598	0.994	0.992	0.00092
Tfe3	9.68E-07	-0.26633	0.655	0.454	0.019219
Tada3	7.37E-07	-0.26791	0.476	0.309	0.014633
Slc16a10	6.78E-07	-0.26799	0.579	0.384	0.013461
Arl5b	1.81E-10	-0.27786	0.276	0.132	3.60E-06
Anapc10	2.45E-06	-0.2807	0.521	0.345	0.048635
Atp8b4	4.45E-07	-0.28084	0.224	0.115	0.008847
Serpinb6b	4.43E-08	-0.2857	0.63	0.438	0.000879
Ap1s2	5.94E-08	-0.29101	0.512	0.326	0.001179
Mkln1	1.42E-06	-0.29122	0.458	0.285	0.028129
Rhoa	9.78E-07	-0.29532	0.997	0.973	0.019425
Snhg4	1.62E-06	-0.29769	0.306	0.178	0.032235
Plau	9.53E-08	-0.29877	0.179	0.083	0.001893
Arhgap29	5.74E-08	-0.30892	0.145	0.059	0.001141
Mmd	7.56E-07	-0.31801	0.603	0.41	0.015004
Jdp2	1.02E-09	-0.31806	0.524	0.327	2.02E-05
Zbtb38	6.42E-09	-0.32727	0.148	0.057	0.000128
Fem1c	5.14E-07	-0.33009	0.367	0.22	0.010212
Fbrs	9.94E-07	-0.33092	0.382	0.236	0.01974
3110062M	1.75E-08	-0.33441	0.445	0.264	0.000348
Larp4	5.44E-08	-0.33489	0.521	0.312	0.00108
Rbm33	6.91E-08	-0.33952	0.297	0.158	0.001371

Hist1h4i	1.21E-06	-0.3412	0.591	0.404	0.023973
Plxnd1	2.51E-07	-0.35796	0.418	0.264	0.004985
1600002K(	2.34E-06	-0.35815	0.37	0.223	0.046382
Tpra1	1.34E-06	-0.36776	0.752	0.543	0.02671
1700003F1	1.88E-06	-0.37661	0.485	0.31	0.037284
Zfp688	2.44E-07	-0.38188	0.221	0.113	0.004847
Pbx2	1.52E-06	-0.38199	0.418	0.263	0.030091
Slc37a2	5.26E-07	-0.38435	0.639	0.451	0.010449
Ncor2	2.23E-06	-0.39114	0.367	0.223	0.044338
Etv6	5.95E-08	-0.41079	0.467	0.283	0.001181
Cdc42	4.48E-11	-0.41329	0.994	0.983	8.90E-07
Nlrp3	6.28E-08	-0.41476	0.627	0.456	0.001247
Fbxw4	5.37E-08	-0.42296	0.43	0.262	0.001066
Ogg1	4.68E-07	-0.42463	0.57	0.381	0.009299
Apol9a	1.21E-06	-0.43057	0.345	0.206	0.023956
Mefv	6.32E-09	-0.44533	0.345	0.189	0.000125
Tfec	1.82E-07	-0.45773	0.667	0.451	0.003619
Vps26b	2.13E-06	-0.46599	0.309	0.178	0.04225
6330407A(	1.39E-06	-0.47348	0.739	0.543	0.027558
Fam102b	6.03E-08	-0.48368	0.355	0.197	0.001198
Csf2rb2	1.29E-07	-0.4886	0.57	0.38	0.002572
Stim1	1.85E-07	-0.49051	0.267	0.143	0.00368
Spata2	4.66E-07	-0.49832	0.273	0.149	0.009247
Tnip1	1.09E-06	-0.52282	0.445	0.285	0.021588
Pole3	1.11E-06	-0.53993	0.421	0.261	0.022019
Camta2	2.92E-08	-0.54177	0.467	0.284	0.000581
Sbf1	6.94E-07	-0.55092	0.348	0.209	0.013789
Abl2	1.46E-06	-0.56746	0.373	0.231	0.028959
Aspa	3.88E-07	-0.59861	0.33	0.192	0.007715
1700112E(	4.83E-09	-0.60338	0.373	0.207	9.60E-05
Eml3	1.59E-06	-0.6182	0.267	0.147	0.031636
Slc40a1	1.97E-07	-0.63295	0.93	0.925	0.00391
Cd4	6.55E-11	-0.64337	0.542	0.338	1.30E-06
Nbeal2	5.24E-07	-0.65582	0.182	0.087	0.010401
Rhod	1.86E-06	-0.67474	0.112	0.046	0.036937
Zfyve16	1.96E-06	-0.68176	0.227	0.122	0.038859
Slc26a11	1.54E-10	-0.70995	0.282	0.133	3.06E-06
Ckb	4.10E-07	-0.71129	0.897	0.9	0.008148
Rffl	2.03E-06	-0.71484	0.285	0.159	0.040382
Gm7609	6.60E-13	-0.72184	0.597	0.351	1.31E-08
Ints10	2.23E-06	-0.72905	0.282	0.161	0.044267
Ttc39b	8.19E-07	-0.74457	0.436	0.266	0.016274
Tnni2	1.61E-07	-0.75467	0.276	0.152	0.003195

Fndc7	2.88E-11	-0.76355	0.176	0.065	5.73E-07
Abcc1	9.34E-07	-0.78527	0.439	0.279	0.018546
Cd5l	5.54E-12	-0.78703	0.994	0.985	1.10E-07
Wiz	5.83E-07	-0.80481	0.182	0.088	0.011579
Heatr7a	3.97E-08	-0.80699	0.388	0.223	0.000788
Trmt6	2.23E-06	-0.80718	0.436	0.269	0.044347
Smcr8	1.14E-06	-0.81112	0.242	0.131	0.022603
Serpinb9	6.55E-08	-0.83362	0.591	0.397	0.001301
Phka2	7.87E-11	-0.84506	0.364	0.186	1.56E-06
Abca2	1.96E-06	-0.86266	0.239	0.129	0.038849
Tslp	1.35E-09	-0.87128	0.536	0.333	2.67E-05
Aldob	2.38E-07	-0.9604	0.076	0.185	0.004723
Gla	2.45E-06	-0.97482	0.585	0.411	0.04875
Gdap10	3.81E-08	-0.99167	0.615	0.423	0.000757
Hoxb7	3.15E-07	-0.99975	0.427	0.269	0.006263
Bmp2	4.76E-09	-1.00895	0.333	0.183	9.45E-05
2310035K2	4.03E-07	-1.10096	0.482	0.3	0.008012
9030625A(	3.25E-07	-1.12133	0.615	0.438	0.006449
Slc36a1	1.78E-06	-1.14037	0.345	0.207	0.03531
C1qtnf1	1.61E-06	-1.18605	0.142	0.065	0.031993
Fkbp1b	1.15E-06	-1.1938	0.212	0.109	0.022747
Abcc9	6.76E-09	-1.20545	0.482	0.294	0.000134
Apaf1	2.18E-06	-1.20874	0.355	0.211	0.043259
Gramd1b	1.95E-06	-1.2713	0.203	0.105	0.0388
Oas2	2.17E-08	-1.27186	0.464	0.285	0.000432
Akr1b8	3.01E-08	-1.28902	0.376	0.221	0.000598
Marco	5.43E-07	-1.28988	0.085	0.199	0.010793
Tnf	5.26E-09	-1.29788	0.509	0.328	0.000104
Sh3tc1	2.47E-11	-1.31609	0.324	0.16	4.90E-07
Rpl3l	3.21E-07	-1.33718	0.267	0.146	0.006371
Mettl21d	1.52E-06	-1.37841	0.303	0.175	0.030201
Vps39	4.04E-07	-1.39237	0.227	0.117	0.00803
Ighmbp2	5.65E-07	-1.58939	0.127	0.053	0.011229
Atad2	1.56E-06	-1.61939	0.452	0.289	0.030945
Gab2	1.02E-06	-1.70306	0.433	0.268	0.02017
Gm216	6.76E-07	-1.72139	0.303	0.173	0.01343
Ttr	3.56E-16	-1.99673	0.458	0.608	7.08E-12
Fam149a	4.37E-07	-2.02719	0.215	0.107	0.008683
Mup3	1.09E-08	-2.10137	0.158	0.291	0.000217
Apoa2	1.80E-10	-2.1516	0.573	0.646	3.57E-06
Apoa1	7.76E-07	-2.17625	0.582	0.614	0.015416
Alb	4.02E-12	-2.19141	0.776	0.807	7.99E-08
D6Mm5e	1.09E-07	-2.20789	0.203	0.1	0.002168

Vtn	1.70E-08	-2.53197	0.13	0.258	0.000337
Hpx	6.29E-07	-2.74912	0.442	0.52	0.012496
Serpina3k	3.53E-11	-2.77169	0.2	0.355	7.01E-07
Apoh	5.50E-08	-3.24603	0.112	0.234	0.001092
Serpina1c	1.67E-08	-3.28858	0.282	0.39	0.000332
Fgb	5.26E-09	-3.29495	0.17	0.309	0.000104
Rbl1	1.08E-06	-3.30601	0.167	0.079	0.021353
B3gnt3	7.82E-08	-3.3161	0.145	0.059	0.001553
Fabp1	4.97E-08	-3.61411	0.345	0.438	0.000986
Lonrf1	0	7.404947	1	0	0
Hif1an	0.00712	3.318273	0.115	0.071	1
Itgax	2.46E-05	3.246179	0.197	0.112	0.48796
Vaultrc5	0.010324	2.939187	0.155	0.104	1
Rft1	0.000425	2.840462	0.164	0.096	1
Gga3	0.018215	2.788185	0.173	0.12	1
Vti1a	0.294865	2.579762	0.145	0.119	1
C430049B(	0.001822	2.369176	0.115	0.066	1
Taf5	5.24E-05	2.120992	0.127	0.063	1
Sh3bp1	0.025141	2.057206	0.115	0.076	1
Pdgfb	0.000754	2.053756	0.273	0.18	1
Ppp6r2	8.10E-06	1.996349	0.118	0.053	0.160863
Polr3a	0.00054	1.972612	0.106	0.054	1
Aifm2	0.165172	1.968915	0.112	0.085	1
Foxk2	0.016825	1.95598	0.182	0.125	1
Zfp653	0.000401	1.924046	0.1	0.05	1
Pip4k2b	0.009721	1.842576	0.2	0.135	1
Chst7	0.000297	1.763336	0.152	0.086	1
Pygo2	0.071193	1.757881	0.124	0.09	1
4931440P2	0.009438	1.754147	0.167	0.113	1
Mbd1	1.61E-05	1.744146	0.333	0.216	0.320158
Pkdcc	0.07749	1.673264	0.103	0.073	1
Rasd1	3.36E-05	1.66693	0.112	0.053	0.667762
Nfkbil1	9.17E-06	1.652149	0.542	0.37	0.18214
Dennd1b	0.005845	1.641353	0.333	0.239	1
Slc39a13	0.013345	1.633097	0.236	0.166	1
Elac1	0.014012	1.544833	0.155	0.105	1
Parp8	0.0001	1.529902	0.227	0.135	1
Rasa4	0.000162	1.523401	0.442	0.307	1
Gm12191	0.056198	1.522591	0.118	0.082	1
Zfp598	0.001362	1.494387	0.267	0.177	1
Fastkd2	0.116808	1.473159	0.109	0.081	1
Mon1b	0.015241	1.451699	0.155	0.108	1
Gbp5	0.010467	1.449098	0.267	0.196	1

Tmem170l	0.014079	1.388153	0.306	0.221	1
Sestd1	0.000974	1.356328	0.191	0.121	1
Tbc1d24	0.063099	1.351681	0.109	0.077	1
Tet2	0.003251	1.336658	0.118	0.069	1
Wwox	0.002286	1.305899	0.112	0.064	1
1500010J0	0.027563	1.282799	0.233	0.17	1
Capn5	2.63E-05	1.260422	0.148	0.076	0.523171
Rnf219	8.29E-05	1.258733	0.118	0.057	1
Pstk	0.066672	1.219992	0.176	0.134	1
Trim3	0.012088	1.188267	0.188	0.128	1
Fam160b1	0.000224	1.184802	0.2	0.118	1
I830012O1	5.56E-05	1.177334	0.324	0.209	1
Atm	0.003663	1.176099	0.173	0.112	1
Aph1b	0.193963	1.170534	0.148	0.119	1
Hgsnat	7.82E-05	1.159701	0.852	0.7	1
Lemd3	0.000981	1.139812	0.106	0.057	1
Dyrk2	0.000203	1.135704	0.315	0.211	1
Nedd1	0.003038	1.130831	0.188	0.121	1
Ctu2	0.003949	1.125154	0.258	0.179	1
Setd2	0.000294	1.124677	0.355	0.239	1
Agk	0.03567	1.108573	0.121	0.083	1
Pdxp	0.107922	1.08652	0.124	0.091	1
Cenpv	0.143916	1.06701	0.248	0.195	1
Erc1	8.30E-06	1.055109	0.227	0.128	0.164747
Mavs	0.037151	1.025312	0.406	0.313	1
Slc30a6	0.032118	0.998995	0.106	0.07	1
Fgfr1op	0.053274	0.960007	0.1	0.068	1
Sfmbt1	0.004994	0.947549	0.306	0.213	1
2310015A1	0.015101	0.920742	0.136	0.09	1
Cep170	0.015005	0.918601	0.306	0.226	1
Nln	0.015793	0.911666	0.161	0.11	1
Ppp1r8	0.002246	0.884208	0.233	0.157	1
Lhfpl2	8.65E-06	0.883636	0.103	0.044	0.171777
Plekhm2	0.000204	0.883026	0.588	0.435	1
2310003H0	0.17766	0.878356	0.103	0.078	1
Arf2	4.63E-05	0.873233	0.512	0.358	0.919303
Gorasp1	0.001892	0.868994	0.1	0.054	1
Ckm	0.000142	0.853081	0.121	0.061	1
Ptdss2	0.019709	0.848091	0.173	0.123	1
Zfp84	0.510902	0.843077	0.103	0.088	1
Zfp865	0.000794	0.827797	0.161	0.096	1
Nek9	0.001441	0.825322	0.452	0.326	1
Pank3	0.043352	0.819418	0.261	0.194	1

Myo9a	0.104431	0.808332	0.673	0.533	1
Marveld1	0.000152	0.799688	0.276	0.175	1
Plekhg4	0.000171	0.79854	0.13	0.07	1
Lcmt2	0.007594	0.795421	0.1	0.059	1
Dtx4	0.022811	0.794246	0.106	0.069	1
B3galtl	0.130789	0.792845	0.191	0.149	1
Mppe1	0.005871	0.791686	0.112	0.068	1
Miip	0.005783	0.788905	0.2	0.132	1
Asb3	0.022462	0.787075	0.106	0.069	1
Zfp335	0.008068	0.776309	0.106	0.065	1
Clec4e	0.003257	0.773616	0.218	0.151	1
Fam199x	0.009872	0.772432	0.115	0.072	1
B4galt6	0.002578	0.77243	0.77	0.608	1
Lnpep	0.024464	0.770905	0.152	0.106	1
Cxcl11	0.000946	0.742743	0.115	0.064	1
Zswim4	0.000217	0.737617	0.218	0.134	1
Lpcat4	0.224634	0.736273	0.133	0.105	1
Ly9	0.013809	0.73305	0.63	0.484	1
Prpf39	0.008585	0.731713	0.173	0.117	1
4930502E1	0.007289	0.731538	0.106	0.065	1
Mocs1	0.002187	0.730714	0.188	0.123	1
Irak3	3.24E-05	0.730445	0.264	0.159	0.644068
Ophn1	0.023812	0.712413	0.158	0.11	1
Cd200r4	0.104369	0.709418	0.121	0.091	1
Vps33a	0.000112	0.704435	0.361	0.237	1
Slc2a6	0.010618	0.695163	0.285	0.206	1
Rev1	3.44E-05	0.690943	0.139	0.07	0.683378
Sowahc	0.0001	0.675479	0.533	0.382	1
Dos	0.001116	0.666211	0.1	0.053	1
Abca1	0.018819	0.659194	0.733	0.579	1
Pvrl4	0.195299	0.652674	0.236	0.194	1
Eif2c3	0.002648	0.651081	0.158	0.101	1
Kdm3b	0.000451	0.650935	0.333	0.223	1
Slc26a2	8.53E-06	0.642827	0.291	0.176	0.169473
Pot1b	0.128241	0.638163	0.294	0.237	1
Ccdc122	0.009335	0.637334	0.179	0.121	1
Nr2f2	0.002931	0.636169	0.133	0.081	1
Tesk1	0.001585	0.629076	0.394	0.279	1
Nit1	1.19E-05	0.628556	0.236	0.135	0.23604
Brpf1	0.002502	0.621476	0.388	0.277	1
Khsrp	0.013353	0.615514	0.258	0.182	1
Asb2	2.64E-05	0.612399	0.485	0.332	0.524818
1700026L0	0.029892	0.605124	0.13	0.09	1

Gprc5c	0.036673	0.602891	0.179	0.131	1
Stat1	0.001816	0.595423	0.852	0.698	1
Ldb2	9.58E-05	0.594149	0.106	0.05	1
Zbtb17	0.000158	0.590833	0.179	0.104	1
2410002F2	0.005335	0.589065	0.194	0.128	1
Rnd1	0.000393	0.587785	0.291	0.196	1
Pik3c2a	0.598919	0.581827	0.285	0.253	1
Gfm2	0.000706	0.580512	0.152	0.088	1
Aoah	5.56E-05	0.571407	0.77	0.56	1
Ing5	0.218055	0.564042	0.139	0.11	1
Slc16a3	0.000119	0.562259	0.224	0.135	1
Orai2	0.003626	0.558845	0.233	0.158	1
Dctn4	0.000234	0.556427	0.324	0.208	1
Phlpp1	0.040041	0.552781	0.106	0.071	1
Aff1	8.00E-05	0.551325	0.285	0.177	1
Atxn2	8.31E-06	0.545714	0.294	0.174	0.165038
5430437J1	3.04E-06	0.544002	0.385	0.246	0.060312
Tmem206	0.012121	0.536954	0.427	0.33	1
Nsmaf	0.000205	0.535989	0.267	0.168	1
Sgsh	0.194952	0.535248	0.212	0.172	1
4933421O	0.005227	0.53459	0.158	0.102	1
Zfp91	1.34E-05	0.53112	0.512	0.36	0.26609
Stxbp1	0.000632	0.523802	0.221	0.14	1
Kdm4a	0.00035	0.523525	0.276	0.178	1
Clec4g	0.019599	0.521937	0.27	0.206	1
Plxdc1	2.20E-05	0.52108	0.164	0.087	0.437452
Lancl2	0.0114	0.520247	0.148	0.1	1
Adcy3	0.00105	0.517247	0.212	0.137	1
Narfl	0.030446	0.514169	0.212	0.16	1
Nup107	0.000735	0.513339	0.103	0.053	1
Tpcn2	0.00147	0.511033	0.309	0.209	1
Pds5b	0.084858	0.510998	0.164	0.123	1
Itpr3	2.32E-05	0.509501	0.236	0.136	0.459949
Ocstamp	3.68E-06	0.508086	0.218	0.122	0.073013
Mmp12	9.33E-05	0.496187	0.164	0.091	1
Ncoa2	0.000432	0.493714	0.239	0.153	1
Ceacam16	3.95E-05	0.485915	0.164	0.087	0.784239
Stx17	0.055101	0.479059	0.179	0.132	1
Lmbr1l	0.004082	0.477526	0.148	0.093	1
Mpv17	0.003921	0.471826	0.394	0.282	1
Psph	0.081559	0.470398	0.109	0.078	1
Rars	0.000409	0.460099	0.452	0.325	1
Thra	0.020445	0.456736	0.127	0.085	1

Crnkl1	0.00044	0.455282	0.279	0.181	1
Hars2	0.154035	0.453324	0.136	0.107	1
Ttc7	5.62E-06	0.450392	0.409	0.264	0.111595
Ptgs2	0.000271	0.44486	0.23	0.146	1
Gabpa	0.419656	0.442892	0.182	0.156	1
D14Ertd66	0.043093	0.432995	0.582	0.454	1
Kif20b	6.41E-05	0.429075	0.112	0.053	1
Irf2bp2	1.59E-05	0.428966	0.921	0.776	0.316553
C230096C1	0.137774	0.424675	0.239	0.191	1
Ly6g6e	0.008046	0.422552	0.209	0.145	1
Ccdc109a	0.086825	0.421715	0.124	0.09	1
Scrib	4.22E-06	0.421446	0.291	0.171	0.083732
Mx2	0.004354	0.421263	0.188	0.124	1
0610007P2	0.139807	0.417829	0.252	0.196	1
Phf2	0.005239	0.415726	0.194	0.128	1
Narf	0.000489	0.413817	0.173	0.103	1
Gtf3c2	0.046691	0.410668	0.258	0.196	1
Renbp	0.00169	0.410577	0.779	0.599	1
E030030I0	0.032783	0.409808	0.167	0.12	1
Ell2	0.023097	0.409242	0.303	0.234	1
Man2a2	0.002983	0.40463	0.288	0.205	1
Oasl1	3.22E-06	0.403599	0.779	0.612	0.063864
B4galt3	0.002223	0.401582	0.252	0.167	1
Pomt1	0.001214	0.398106	0.118	0.066	1
Prune2	0.000212	0.396505	0.345	0.227	1
Pgpep1	4.46E-06	0.393463	0.424	0.269	0.08861
Rc3h1	0.004109	0.393079	0.312	0.222	1
Ankfy1	0.000149	0.39306	0.521	0.36	1
Rbm4b	0.008391	0.389435	0.112	0.069	1
Mvk	0.001782	0.387542	0.139	0.084	1
Slk	0.002858	0.38245	0.306	0.212	1
Gpr107	5.07E-05	0.378588	0.367	0.245	1
Trim24	0.000427	0.377722	0.23	0.145	1
Letm2	0.000152	0.375892	0.121	0.062	1
Pi4k2b	0.022674	0.374454	0.133	0.09	1
Rxrb	9.80E-06	0.372655	0.515	0.353	0.194565
Sft2d3	0.000509	0.367856	0.212	0.133	1
Acsl4	0.01762	0.365506	0.567	0.446	1
Abca3	0.008079	0.364802	0.239	0.166	1
Uba7	0.061488	0.362965	0.264	0.201	1
Ankrd37	0.00012	0.360971	0.191	0.113	1
H2-M2	0.000393	0.356934	0.215	0.138	1
Clec10a	2.66E-05	0.35169	0.706	0.524	0.528998

Dbt	0.044468	0.350869	0.115	0.08	1
Rhoq	0.01978	0.349925	0.379	0.284	1
Med13	0.001586	0.347378	0.248	0.168	1
Gpd2	0.007455	0.34652	0.315	0.232	1
Thrb	0.030735	0.345542	0.164	0.117	1
Hira	0.118052	0.344942	0.109	0.081	1
Atg10	0.015752	0.343463	0.27	0.198	1
Spata24	0.000495	0.339541	0.158	0.093	1
Relb	3.04E-05	0.335187	0.533	0.373	0.602997
2810403A(	0.001361	0.33288	0.264	0.176	1
Zfp821	0.001425	0.332514	0.206	0.13	1
Itprip	0.000553	0.332405	0.182	0.111	1
Clcn7	0.004058	0.331255	0.418	0.311	1
Cd1d1	0.050302	0.330878	0.615	0.501	1
Dpp7	0.079229	0.325962	0.345	0.271	1
Abca7	0.026164	0.32447	0.133	0.092	1
Cry1	0.002336	0.324201	0.1	0.055	1
Gyk	5.83E-05	0.319818	0.352	0.226	1
Fam20c	5.34E-05	0.319408	0.236	0.14	1
Mpp5	0.000557	0.318395	0.121	0.065	1
Cd209g	2.89E-05	0.317963	0.261	0.16	0.57486
Rrm2b	0.000939	0.317467	0.194	0.121	1
Arid5a	9.64E-06	0.317446	0.324	0.203	0.191371
Coasy	0.010814	0.314703	0.276	0.195	1
Lemd2	0.000217	0.314142	0.412	0.281	1
Nsdhl	1.56E-05	0.311819	0.273	0.162	0.309499
Rps19-ps3	0.00034	0.310279	0.155	0.087	1
Pvrl2	0.001398	0.309075	0.267	0.177	1
Ano6	0.000125	0.307177	0.921	0.765	1
Magohb	1.46E-05	0.30521	0.633	0.457	0.29085
1110018G(	0.109162	0.304722	0.167	0.129	1
Rnf150	3.49E-05	0.301838	0.258	0.157	0.69397
Rpap2	0.084239	0.300556	0.127	0.094	1
Abr	0.004205	0.297404	0.385	0.281	1
Zdhhc14	0.009908	0.296682	0.385	0.282	1
Trim34a	0.00247	0.293607	0.282	0.19	1
Nusap1	0.001188	0.292349	0.115	0.065	1
9130206I2	0.000158	0.291847	0.1	0.048	1
Zmym4	0.000724	0.291548	0.17	0.102	1
Supt5h	1.11E-05	0.290507	0.527	0.368	0.220298
Rab22a	0.009856	0.290161	0.382	0.28	1
Cdca3	9.06E-06	0.289017	0.224	0.128	0.179939
Nampt	0.001043	0.286883	0.9	0.782	1

Otud6b	0.061349	0.285375	0.215	0.16	1
Kctd20	0.087955	0.284014	0.106	0.077	1
2700078E1	0.012359	0.283827	0.342	0.257	1
Lactb2	0.002542	0.283483	0.161	0.099	1
Nagk	0.000437	0.282484	0.855	0.657	1
Ints3	4.15E-06	0.279525	0.242	0.135	0.082497
Ptpre	3.19E-06	0.27448	0.479	0.313	0.063416
Cyp4b1	1.00E-05	0.273724	0.179	0.094	0.199049
Pisd-ps3	0.000356	0.272243	0.748	0.584	1
Tapt1	0.00363	0.272194	0.112	0.065	1
Fryl	0.01974	0.271982	0.161	0.111	1
Atp5sl	0.001098	0.270977	0.164	0.1	1
Hist1h1c	4.05E-06	0.270314	0.824	0.639	0.080522
Rbm47	0.017564	0.270212	0.548	0.412	1
Slamf7	3.19E-06	0.26753	0.509	0.346	0.063309
Itga5	5.69E-05	0.26572	0.47	0.326	1
Necap1	7.35E-05	0.265199	0.352	0.235	1
Klhl24	0.017369	0.263904	0.255	0.184	1
Micu1	0.020518	0.262743	0.336	0.254	1
Mdc1	0.076676	0.261864	0.127	0.093	1
Slx1b	0.216473	0.261577	0.4	0.333	1
Pyroxd1	0.035839	0.258633	0.118	0.082	1
Ppox	0.032494	0.258538	0.188	0.138	1
Smad6	0.000687	0.25762	0.415	0.297	1
Dusp16	0.000457	0.257154	0.2	0.123	1
Tagap1	0.038671	0.255211	0.106	0.07	1
Dab2	0.000172	0.254344	0.888	0.73	1
Ipo4	0.039006	0.253251	0.221	0.166	1
Pla2g12a	0.004852	0.252563	0.355	0.258	1
Slc22a23	0.000131	0.250983	0.23	0.14	1
Tmem167l	0.032153	0.250887	0.209	0.155	1
Fabp4	0.000126	0.249149	0.333	0.235	1
Pusl1	0.435715	0.247371	0.127	0.108	1
Rasip1	0.000168	0.245926	0.148	0.082	1
Gpr182	0.000353	0.24565	0.203	0.126	1
Stard3	0.002067	0.243413	0.615	0.476	1
1700086O	0.485321	0.242813	0.1	0.086	1
Itpk1	0.260435	0.242472	0.273	0.231	1
Tpx2	0.000206	0.24207	0.161	0.092	1
C1galt1c1	0.224417	0.241755	0.336	0.282	1
Slc35a5	0.056733	0.241356	0.221	0.17	1
Fscn1	3.71E-05	0.24135	0.152	0.079	0.736792
Stk40	0.016392	0.241123	0.333	0.252	1

Isg15	3.49E-05	0.239643	0.891	0.768	0.693778
Ube2c	0.000131	0.238562	0.215	0.134	1
Htt	0.000136	0.234155	0.255	0.159	1
Supt6h	0.00473	0.233763	0.376	0.272	1
Ccnb2	4.56E-05	0.233402	0.176	0.098	0.905743
Plbd1	0.000222	0.2311	0.942	0.869	1
Irf1	2.03E-05	0.229885	0.803	0.621	0.40285
Arhgef2	6.22E-06	0.229668	0.679	0.492	0.123577
Gigyf1	3.46E-06	0.228768	0.327	0.198	0.068754
Rhbdd2	0.379062	0.228504	0.112	0.093	1
C530028O	0.000152	0.227459	0.224	0.139	1
Rapgef6	0.000505	0.226501	0.533	0.404	1
Mga	0.000796	0.226074	0.282	0.189	1
Plcb2	0.005882	0.225328	0.224	0.155	1
Fgl2	3.58E-05	0.224934	0.864	0.683	0.71021
Nfkb1	2.53E-05	0.224258	0.615	0.434	0.50198
Zdhhc8	0.12721	0.22423	0.115	0.087	1
Kat5	0.060472	0.223479	0.248	0.189	1
Sirpa	0.000245	0.220878	0.967	0.89	1
Plbd2	2.50E-05	0.219987	0.867	0.672	0.495754
Chpf	0.029773	0.219693	0.176	0.127	1
Nkap	2.77E-05	0.219218	0.452	0.301	0.549368
G730013B	0.001035	0.219086	0.258	0.173	1
Clec5a	0.000424	0.217334	0.439	0.328	1
Cybb	4.06E-05	0.216328	0.982	0.94	0.805403
Phf20l1	9.66E-05	0.216218	0.524	0.38	1
Hk2	0.0001	0.21596	0.855	0.706	1
Syk	0.025056	0.215269	0.773	0.635	1
Aak1	1.81E-05	0.214989	0.336	0.21	0.359579
Sirpb1b	0.019039	0.214027	0.418	0.327	1
Tmem135	0.12393	0.213156	0.285	0.23	1
Gsk3a	1.38E-05	0.212974	0.455	0.299	0.274598
Btnl2	7.12E-06	0.212592	0.139	0.066	0.141399
Map4k4	2.87E-06	0.212568	0.336	0.202	0.056965
Mcm5	5.98E-06	0.210478	0.436	0.281	0.118779
Spic	2.89E-06	0.21008	0.63	0.463	0.057492
Naf1	0.028185	0.209794	0.185	0.132	1
Pip4k2c	0.000216	0.207689	0.282	0.178	1
Hs6st1	3.90E-05	0.20683	0.576	0.393	0.773636
Slc25a33	0.00087	0.206447	0.739	0.586	1
Ndrg3	0.000161	0.206183	0.3	0.195	1
Blnk	0.000912	0.205629	0.27	0.181	1
Ip6k2	3.35E-06	0.205496	0.155	0.074	0.066567

Cd40	1.13E-05	0.205468	0.621	0.444	0.225121
2310035C2	0.128186	0.204581	0.176	0.137	1
Slc16a6	0.000144	0.203697	0.227	0.138	1
Ube2s	5.30E-06	0.20231	0.876	0.697	0.105173
Mfng	0.000149	0.199825	0.17	0.098	1
Icam1	1.27E-05	0.198792	0.748	0.585	0.251867
1110014N1	0.000214	0.197158	0.327	0.216	1
Ccr3	0.000151	0.196679	0.606	0.457	1
Btrc	0.000357	0.196479	0.185	0.109	1
Ly6a	3.33E-05	0.195406	0.927	0.82	0.662311
2610306M	0.002974	0.194205	0.13	0.078	1
F8a	0.001928	0.193703	0.191	0.121	1
Efcab4a	0.020685	0.193576	0.27	0.203	1
2310061C1	0.000214	0.191058	0.582	0.433	1
Rabgef1	2.46E-05	0.190619	0.43	0.29	0.487695
Cstb	1.03E-05	0.189046	0.985	0.96	0.204445
Pfdn5	1.39E-05	0.188936	1	0.962	0.276548
Xcr1	1.78E-05	0.188495	0.418	0.285	0.353833
Pramef8	0.18135	0.187037	0.127	0.1	1
Rhob	1.59E-05	0.186769	0.894	0.759	0.315482
Grina	3.41E-05	0.186243	0.915	0.79	0.67721
Pgk1	0.005309	0.185627	0.915	0.792	1
AW549877	0.000217	0.185128	0.3	0.2	1
Zfand5	1.04E-05	0.184472	0.915	0.741	0.207304
C5ar1	0.024626	0.184079	0.788	0.65	1
Tmem176b	7.44E-06	0.18391	0.964	0.897	0.147669
Mtch1	0.000691	0.182556	0.83	0.666	1
Flna	0.000188	0.181828	0.858	0.71	1
Prdx4	1.29E-05	0.181597	0.812	0.655	0.255334
2610507B1	0.003734	0.180737	0.642	0.5	1
Fyb	5.31E-06	0.180733	0.961	0.884	0.105489
Wdfy3	0.009272	0.180076	0.648	0.5	1
Marcksl1	0.000452	0.17989	0.97	0.929	1
Chd4	5.88E-06	0.178836	0.724	0.539	0.11671
Crem	3.60E-06	0.178096	0.624	0.438	0.071511
Trp53i13	0.131753	0.177245	0.133	0.104	1
Birc5	4.85E-05	0.174123	0.239	0.147	0.962862
Gm12942	0.011465	0.17281	0.379	0.277	1
Plcg2	0.001083	0.172564	0.582	0.426	1
Rgs2	0.008999	0.172393	0.939	0.841	1
Ldb1	0.003827	0.172268	0.294	0.211	1
Cnot7	0.026146	0.171081	0.17	0.12	1
Aftph	0.113847	0.170755	0.455	0.369	1

Edem1	0.000587	0.170441	0.703	0.532	1
Cand1	1.30E-05	0.170216	0.376	0.241	0.258787
Socs6	4.86E-05	0.169726	0.261	0.156	0.964905
Fgfr1op2	2.85E-06	0.169376	0.818	0.639	0.056585
Slc4a5	0.002058	0.168866	0.206	0.136	1
Slc46a3	0.083755	0.168729	0.252	0.193	1
2310001H	0.000659	0.167806	0.355	0.246	1
Egfl7	0.001116	0.166835	0.273	0.191	1
Tmem120l	0.001763	0.166565	0.133	0.078	1
Sepp1	0.000169	0.16534	1	0.997	1
H2-DMb1	6.30E-05	0.164912	0.97	0.877	1
Serpinb6a	6.65E-05	0.16449	0.985	0.933	1
Arfip1	0.000661	0.164459	0.294	0.198	1
Hs1bp3	0.00468	0.164325	0.182	0.12	1
Slc25a5	0.000148	0.163498	0.994	0.979	1
Rbm3	0.000374	0.163021	0.961	0.91	1
Aup1	4.46E-05	0.162998	0.918	0.76	0.886674
Bri3	0.000269	0.162488	0.982	0.922	1
Nfe2l2	3.98E-06	0.162416	0.918	0.755	0.079004
Gas5	2.71E-05	0.162301	0.936	0.814	0.538029
Nrp1	0.000241	0.162147	0.848	0.693	1
C4b	2.86E-06	0.161999	0.924	0.744	0.056887
Zfp944	0.321158	0.161742	0.1	0.081	1
Oit3	0.000196	0.161455	0.17	0.098	1
Eif3h	0.000112	0.161439	0.958	0.878	1
Gm13139	0.076323	0.160326	0.109	0.078	1
Wdr76	0.005574	0.15966	0.103	0.06	1
Ccdc82	0.038162	0.158763	0.139	0.098	1
Fxyd5	2.32E-05	0.158696	0.961	0.872	0.461416
Ubr5	3.12E-05	0.158306	0.552	0.385	0.619474
B3gnt2	0.000153	0.157967	0.455	0.325	1
Zfp212	0.00096	0.157725	0.127	0.072	1
Clint1	0.000218	0.157167	0.606	0.431	1
Vamp8	6.54E-06	0.157123	0.985	0.977	0.129866
Scd2	0.114752	0.156613	0.136	0.103	1
Ckap4	0.000194	0.156173	0.597	0.441	1
Pabpc1	7.52E-06	0.155121	0.994	0.965	0.149268
Rnase4	2.89E-06	0.154539	0.676	0.486	0.057423
Tm2d2	2.04E-05	0.154321	0.879	0.716	0.405161
Dtx3	0.000415	0.154242	0.121	0.064	1
Cenpm	5.37E-06	0.154236	0.197	0.105	0.106611
Vegfb	0.000199	0.153933	0.218	0.132	1
Mfsd6	0.001572	0.152741	0.127	0.075	1

Parp1	0.002533	0.151951	0.367	0.26	1
Gatc	0.062941	0.15132	0.248	0.191	1
Ndufa1	0.003351	0.151293	0.952	0.886	1
Nbeal1	0.001904	0.150557	0.233	0.157	1
Srek1	0.008032	0.150379	0.309	0.225	1
Grpel2	0.000272	0.150375	0.288	0.19	1
Zfp706	1.53E-05	0.150351	0.952	0.873	0.304081
Slc12a2	0.000254	0.149878	0.303	0.202	1
Grap	3.25E-06	0.148666	0.582	0.4	0.064607
H2afj	6.44E-05	0.147162	0.976	0.962	1
Syap1	0.000289	0.147157	0.661	0.506	1
Cmtm8	0.000111	0.146197	0.339	0.229	1
Psat1	0.002021	0.145481	0.124	0.073	1
Eef2	1.75E-05	0.145289	0.997	0.98	0.347699
Hdac10	0.005508	0.145015	0.23	0.163	1
Dnase1l3	0.000137	0.144739	0.7	0.549	1
Slc7a5	9.27E-06	0.14467	0.115	0.052	0.184106
Midn	9.39E-06	0.144477	0.645	0.487	0.186526
Snx18	0.000156	0.144296	0.715	0.532	1
Map4k5	9.99E-06	0.143905	0.152	0.076	0.198341
2310021P1	9.81E-05	0.143753	0.318	0.21	1
Atp6v1b2	0.007151	0.14332	0.891	0.775	1
Tmem176a	0.000147	0.143144	0.9	0.781	1
Socs1	0.007933	0.142119	0.145	0.095	1
Rassf1	2.14E-05	0.141991	0.627	0.464	0.425766
Tle4	0.001882	0.141968	0.5	0.354	1
Atox1	0.001894	0.141728	0.997	0.981	1
St13	0.001322	0.141396	0.924	0.817	1
Clec12a	0.000986	0.141344	0.97	0.912	1
Cox7a2l	0.001632	0.141271	0.976	0.895	1
Cenpe	2.01E-05	0.140946	0.167	0.089	0.398943
BC024582	0.000154	0.140877	0.282	0.179	1
Gnai2	1.94E-05	0.139605	0.994	0.965	0.385884
2810417H1	0.000238	0.139584	0.23	0.147	1
Naa35	8.16E-05	0.138993	0.297	0.193	1
Ndufb11	0.001256	0.138313	0.903	0.823	1
Slc25a37	9.35E-06	0.138224	0.224	0.126	0.185766
Gja1	0.000228	0.138138	0.164	0.093	1
Jun	9.45E-06	0.137939	0.864	0.717	0.187607
Csf3r	2.54E-06	0.137385	0.524	0.358	0.050496
Ubr2	3.07E-05	0.136809	0.418	0.276	0.609074
Rpl8	3.39E-05	0.136669	0.997	0.998	0.673069
2210013O	2.74E-06	0.136661	0.752	0.579	0.054357

Zbtb7b	0.002752	0.136482	0.47	0.34	1
Rbm39	0.001233	0.136463	0.942	0.827	1
Sec11c	0.000255	0.136292	0.918	0.795	1
Ppp1r9a	0.001894	0.136042	0.358	0.251	1
Nars	6.84E-05	0.135752	0.894	0.756	1
Oxct1	9.21E-06	0.135627	0.797	0.615	0.182838
Sntb2	0.000292	0.135014	0.245	0.157	1
Acyp2	0.001693	0.135014	0.155	0.095	1
Tmem184c	0.005744	0.134756	0.145	0.093	1
Aprt	0.007983	0.134756	0.93	0.845	1
Pcbp1	0.000146	0.134662	0.942	0.865	1
Taz	4.13E-05	0.134277	0.542	0.378	0.819465
Selk	2.26E-05	0.134078	0.979	0.925	0.448583
Mgat4b	6.14E-06	0.133205	0.488	0.327	0.121897
Morc2a	4.90E-05	0.133083	0.155	0.082	0.97215
Sos1	0.003887	0.132495	0.17	0.109	1
Ap2b1	2.54E-06	0.131996	0.612	0.42	0.050421
Gripap1	1.05E-05	0.131808	0.597	0.433	0.208914
Cask	0.005931	0.131651	0.209	0.142	1
Arhgap8	0.048116	0.131382	0.17	0.128	1
Cdkn2d	5.24E-06	0.131132	0.261	0.153	0.104025
Gcnt2	6.87E-05	0.130666	0.267	0.166	1
Cdca8	0.000163	0.130344	0.194	0.115	1
Prkx	6.15E-06	0.130094	0.412	0.276	0.122082
Car2	7.19E-06	0.129797	0.355	0.227	0.142832
Klf6	1.01E-05	0.129569	0.924	0.785	0.201026
Nrg4	1.51E-05	0.128653	0.185	0.099	0.300728
Rexo1	0.001826	0.128366	0.285	0.192	1
Rnf44	0.010128	0.127931	0.342	0.257	1
Tagln2	0.00043	0.127717	0.879	0.759	1
Reep5	0.000297	0.127232	0.979	0.918	1
Nfkbie	1.33E-05	0.126811	0.57	0.412	0.264198
H2afz	0.000654	0.126354	0.982	0.95	1
E130311K1	0.096446	0.126323	0.115	0.084	1
Clec4a1	0.000153	0.126079	0.955	0.825	1
Zc3hav1	0.000328	0.126031	0.482	0.35	1
Ivd	0.031777	0.125828	0.139	0.098	1
Raph1	0.000126	0.125664	0.345	0.227	1
Txnip	0.0709	0.125303	0.885	0.771	1
Slc8a1	0.00013	0.123859	0.458	0.315	1
1190002F1	2.96E-06	0.123603	0.236	0.134	0.05871
Mmp13	2.28E-05	0.123107	0.379	0.254	0.453589
Gtf2e2	0.020106	0.122783	0.415	0.32	1

Npc2	0.000696	0.122365	0.997	0.997	1
Mcl1	0.004287	0.122311	0.97	0.87	1
Vrk1	3.06E-05	0.12185	0.318	0.2	0.608043
Bag3	2.88E-05	0.121686	0.415	0.276	0.572035
Atrx	7.20E-06	0.121682	0.748	0.56	0.143032
Ift27	6.36E-06	0.121663	0.482	0.318	0.126375
Vamp5	1.90E-05	0.121064	0.836	0.652	0.377929
Glt25d1	3.64E-05	0.120791	0.927	0.789	0.722568
Ms4a6b	8.83E-05	0.120186	0.83	0.658	1
Dusp1	0.000174	0.120183	0.985	0.908	1
Dock7	0.00777	0.119384	0.248	0.179	1
Rpl17	0.000184	0.119127	1	1	1
Mid1ip1	2.28E-05	0.11849	0.694	0.524	0.452363
Arl4a	4.71E-05	0.118305	0.352	0.235	0.936362
Brd2	0.000113	0.118129	0.8	0.632	1
Tpm1	9.44E-05	0.118105	0.391	0.267	1
Fam134b	8.51E-05	0.117874	0.279	0.174	1
Akap2	0.000149	0.117856	0.139	0.075	1
Zdhhc21	0.025417	0.116894	0.239	0.173	1
Ncf4	0.000606	0.116884	0.897	0.762	1
Idh3g	0.002788	0.116844	0.848	0.701	1
Sec31a	0.001188	0.116785	0.488	0.356	1
Clec4a2	0.000191	0.116462	0.918	0.822	1
Apc	0.000252	0.116402	0.479	0.346	1
Icam2	0.000502	0.115898	0.585	0.449	1
Itgb1	0.013446	0.115474	0.906	0.825	1
Cox6c	0.003364	0.115257	0.991	0.981	1
Kctd2	0.053281	0.115179	0.224	0.17	1
Ier2	2.19E-05	0.114799	0.903	0.719	0.434336
Snx10	0.001684	0.114484	0.764	0.588	1
Fam63a	0.000132	0.113982	0.324	0.208	1
Tgm2	0.014342	0.11374	0.964	0.899	1
Atp6v0d1	0.000822	0.113674	0.912	0.786	1
Rpl36a	8.90E-05	0.113467	0.997	0.995	1
1810058I2	0.000376	0.113452	0.918	0.747	1
Klf2	0.000375	0.112866	0.964	0.867	1
Dnajb1	4.82E-05	0.112305	0.715	0.534	0.957118
Fnip1	1.81E-05	0.112283	0.545	0.384	0.358679
Lmo2	9.54E-05	0.112039	0.812	0.654	1
Rpl13	2.64E-05	0.11189	1	1	0.523709
B630005N	3.15E-05	0.111823	0.521	0.354	0.626489
Pnrc1	0.000395	0.111406	0.948	0.783	1
Samsn1	4.28E-05	0.111234	0.6	0.44	0.850595

Casp4	0.000125	0.111223	0.433	0.306	1
Dync1h1	2.98E-05	0.111208	0.624	0.441	0.590907
Hebp1	0.066712	0.111107	0.809	0.67	1
Igfbp4	0.000729	0.110463	0.645	0.509	1
Grb2	0.000117	0.110211	0.961	0.868	1
Calm1	0.000132	0.110176	1	0.995	1
Fabp5	0.000142	0.110125	0.473	0.343	1
Derl1	0.003152	0.109635	0.93	0.825	1
Hist1h2bc	0.00103	0.109496	0.912	0.787	1
Ehd4	0.000119	0.109016	0.861	0.683	1
Tgoln1	0.000488	0.108766	0.733	0.596	1
Atp6v1f	0.000173	0.108665	0.994	0.981	1
Poldip3	1.64E-05	0.108203	0.645	0.476	0.326073
Cbr2	0.000578	0.107149	0.161	0.096	1
Nedd9	0.006768	0.106828	0.215	0.147	1
Zfp36l2	0.000129	0.105791	0.855	0.713	1
Ptrh2	0.572858	0.105646	0.212	0.188	1
Gpr116	4.34E-05	0.1053	0.17	0.093	0.862171
Cd55	3.30E-05	0.105103	0.609	0.451	0.654585
Serbp1	0.000942	0.104933	0.97	0.895	1
Rps20	1.89E-05	0.104629	0.997	0.998	0.375706
Nhp2l1	0.001425	0.104594	0.952	0.832	1
Ninj1	0.020306	0.104425	0.985	0.968	1
Cebpb	0.001559	0.104365	0.988	0.966	1
Myom1	0.008597	0.104178	0.1	0.061	1
Zyx	0.000539	0.104089	0.861	0.712	1
Slc38a2	0.00038	0.103683	0.618	0.472	1
Txndc12	0.007979	0.103669	0.464	0.354	1
Gimap6	8.02E-05	0.103488	0.224	0.135	1
Gm2a	0.051016	0.103064	0.942	0.868	1
Lsm11	0.100326	0.102684	0.1	0.072	1
AI413582	0.000135	0.102569	0.852	0.665	1
Diap1	8.20E-05	0.102388	0.339	0.225	1
Psap	0.028887	0.102189	0.997	0.996	1
Zfand6	0.000193	0.101936	0.761	0.586	1
Timp3	2.01E-05	0.10186	0.182	0.099	0.398611
Grasp	0.000888	0.101535	0.221	0.143	1
Kdr	2.23E-05	0.100805	0.139	0.069	0.442722
Tomm20	0.000516	0.100752	0.945	0.873	1
Sp1	0.000214	0.100628	0.361	0.235	1
Igfbp7	0.290005	0.100428	0.409	0.373	1
D8Ertd738	0.003584	0.100351	0.976	0.93	1
Plxdc2	5.16E-05	0.100346	0.273	0.169	1

Srsf7	0.188591	-0.10009	0.773	0.654	1
Iscu	0.33426	-0.10011	0.909	0.777	1
Tspan9	1.72E-05	-0.10013	0.385	0.251	0.341256
Nol8	0.012169	-0.10019	0.227	0.158	1
Fnta	0.002536	-0.10031	0.485	0.355	1
Galnt6	1.99E-05	-0.10031	0.497	0.344	0.394231
Adipor1	0.288333	-0.10032	0.936	0.827	1
Fam76b	0.076454	-0.10041	0.285	0.226	1
Hp1bp3	0.00213	-0.10051	0.555	0.417	1
Spint2	0.01453	-0.10052	0.133	0.087	1
Arl8a	0.00294	-0.10056	0.773	0.623	1
Etfa	0.012595	-0.10071	0.752	0.574	1
2010320M	0.000166	-0.10078	0.336	0.222	1
Pin1	0.079797	-0.10085	0.624	0.519	1
Hoxb6	1.31E-05	-0.10094	0.109	0.048	0.260688
Sp140	0.047605	-0.10095	0.764	0.604	1
Arhgap15	0.016436	-0.10103	0.418	0.31	1
Psmd12	0.001323	-0.10105	0.673	0.494	1
Tpp2	0.000656	-0.10106	0.503	0.358	1
Rnf7	0.485775	-0.1012	0.867	0.78	1
Hbp1	0.005273	-0.10125	0.47	0.359	1
Rab43	2.59E-05	-0.1013	0.779	0.588	0.514569
Snx4	0.037878	-0.10131	0.361	0.274	1
Lsm5	0.234631	-0.1014	0.773	0.649	1
Nfat5	0.012134	-0.10144	0.367	0.275	1
Suclg2	0.00238	-0.10157	0.545	0.4	1
Ccdc107	0.002584	-0.10162	0.658	0.52	1
Cyp4f18	0.000129	-0.10162	0.658	0.485	1
Bphl	0.008116	-0.10174	0.194	0.131	1
Utp14a	0.002799	-0.10178	0.533	0.397	1
Atp5d	0.961975	-0.10181	0.958	0.924	1
Plekha2	0.005954	-0.10193	0.642	0.488	1
Hnrnpab	0.327071	-0.10194	0.912	0.818	1
Tyms	0.015343	-0.10217	0.248	0.182	1
Ppp2r2d	0.002824	-0.1022	0.339	0.241	1
Tmem111	0.003643	-0.10236	0.706	0.529	1
Cndp2	0.636743	-0.10255	0.927	0.834	1
Mat2b	0.002861	-0.10266	0.715	0.555	1
Arf6	0.596021	-0.10267	0.845	0.738	1
Ypel3	0.131066	-0.10271	0.682	0.567	1
Rell1	0.000127	-0.10278	0.43	0.299	1
Snrnp70	0.161622	-0.10286	0.712	0.563	1
Pea15a	0.012197	-0.10301	0.83	0.669	1

Tifa	0.108805	-0.10326	0.561	0.453	1
Anxa6	0.106946	-0.10343	0.473	0.386	1
Eif5a	0.43031	-0.10349	0.979	0.96	1
Tssc4	5.69E-06	-0.10355	0.43	0.275	0.113067
Sumo2	0.663461	-0.10358	0.964	0.91	1
Ewsr1	0.807331	-0.10377	0.827	0.733	1
H2-M3	0.238471	-0.10393	0.779	0.642	1
Ndufb2	0.727544	-0.10393	0.903	0.813	1
Ubxn1	0.071955	-0.10412	0.933	0.791	1
Cdkn2c	7.67E-05	-0.10424	0.282	0.175	1
Cd84	0.02394	-0.10438	0.906	0.774	1
Osgep	0.001439	-0.10438	0.691	0.539	1
Nfu1	0.014437	-0.1044	0.648	0.531	1
9930021J0	0.000647	-0.10443	0.245	0.16	1
Pafah1b3	0.014858	-0.10461	0.394	0.307	1
Trem1	0.014402	-0.10487	0.148	0.102	1
Bnip3l	0.551148	-0.10487	0.733	0.614	1
1700061G	0.003011	-0.10497	0.1	0.056	1
Fech	0.083165	-0.10501	0.476	0.363	1
Nadk	0.13817	-0.10502	0.827	0.669	1
2810474O	9.74E-05	-0.10508	0.552	0.404	1
Rab18	0.029742	-0.10514	0.776	0.627	1
Myd88	0.014679	-0.10538	0.561	0.438	1
Myg1	0.000685	-0.10539	0.436	0.318	1
Sdha	0.073608	-0.10574	0.745	0.602	1
Prrc2c	0.002572	-0.10582	0.664	0.501	1
Apoa1bp	0.000701	-0.10592	0.755	0.559	1
Coro1b	0.789863	-0.10597	0.945	0.86	1
Nme1	0.567576	-0.106	0.906	0.816	1
Ext1	0.001686	-0.10609	0.373	0.265	1
Gars	0.001099	-0.10611	0.667	0.495	1
Zbtb44	0.012301	-0.10611	0.164	0.111	1
Slc15a4	1.63E-05	-0.10628	0.476	0.328	0.32319
Bzw1	0.042527	-0.10632	0.612	0.489	1
Tmem120a	0.000156	-0.10648	0.47	0.334	1
Pfdn1	0.587367	-0.10664	0.858	0.735	1
2900064A1	0.014179	-0.10667	0.818	0.668	1
Itpr2	0.00133	-0.10671	0.191	0.118	1
Pprc1	0.033944	-0.1068	0.167	0.119	1
0610010K1	0.069135	-0.10685	0.791	0.683	1
Aurkaip1	0.47535	-0.1069	0.873	0.776	1
Gcap14	0.004004	-0.10692	0.197	0.127	1
Txndc17	0.494751	-0.10705	0.945	0.892	1

Bsdc1	0.001812	-0.10708	0.245	0.162	1
Mid1	0.702174	-0.10714	0.1	0.105	1
Pik3r1	0.019351	-0.10722	0.585	0.462	1
Tm9sf1	0.002131	-0.10723	0.53	0.383	1
Ahsa1	0.582579	-0.1073	0.861	0.748	1
Gadd45gip	0.066179	-0.10741	0.724	0.603	1
Maea	0.000734	-0.10757	0.57	0.41	1
Tmem14c	0.243214	-0.10769	0.97	0.929	1
Ggh	0.076934	-0.10798	0.606	0.483	1
Fam76a	0.000618	-0.10799	0.521	0.377	1
Rtn4	0.457631	-0.10802	0.924	0.881	1
Gpcpd1	0.000115	-0.10808	0.627	0.453	1
0610007C2	0.079267	-0.10809	0.767	0.642	1
Tbca	0.855782	-0.1084	0.955	0.928	1
Alkbh5	0.000437	-0.10852	0.506	0.364	1
Anp32b	0.286436	-0.10855	0.924	0.864	1
Bloc1s1	0.800434	-0.10855	0.897	0.817	1
Hnrpdl	0.025041	-0.10867	0.682	0.548	1
Fam122a	0.000263	-0.10873	0.37	0.249	1
Gfra2	0.581316	-0.10879	0.197	0.175	1
0610011F0	0.437361	-0.10879	0.136	0.148	1
Pnrc2	0.020119	-0.10879	0.558	0.434	1
Gnb1	0.475063	-0.10885	0.964	0.891	1
Iffo2	0.032324	-0.10887	0.173	0.124	1
Sgsm2	3.96E-05	-0.10891	0.139	0.072	0.785798
Arrdc1	3.05E-05	-0.10894	0.439	0.296	0.604963
Arl4c	0.00792	-0.10917	0.755	0.605	1
Naga	0.000167	-0.10917	0.606	0.445	1
Nlk	0.000374	-0.10921	0.185	0.11	1
Pja1	0.110291	-0.10925	0.27	0.218	1
Ing3	0.000177	-0.10929	0.239	0.148	1
Ppp1cb	0.010499	-0.1095	0.648	0.507	1
Ccdc12	0.801544	-0.10958	0.961	0.936	1
Sltm	0.003869	-0.10966	0.545	0.414	1
Sephs2	0.010742	-0.10973	0.43	0.325	1
Puf60	0.221203	-0.10982	0.691	0.583	1
Fyttd1	3.74E-05	-0.10983	0.385	0.249	0.743091
Hibadh	0.012768	-0.10997	0.63	0.486	1
Slc4a2	0.298709	-0.11001	0.152	0.127	1
Gclc	0.001829	-0.11005	0.391	0.286	1
Eif4ebp2	0.011516	-0.11018	0.2	0.139	1
Pppde2	0.001772	-0.11042	0.439	0.318	1
Rassf4	0.239927	-0.11053	0.861	0.737	1

Rad50	0.000194	-0.1106	0.37	0.247	1
Spr	0.008523	-0.11082	0.724	0.579	1
Uqcrq	0.631543	-0.11084	0.982	0.959	1
Vmp1	0.192687	-0.11094	0.824	0.706	1
Slfn5	0.085024	-0.11096	0.776	0.647	1
Nedd8	0.458082	-0.11103	0.988	0.93	1
Cox5a	0.111309	-0.11127	1	0.99	1
Gga1	0.000419	-0.11144	0.739	0.561	1
Lilrb4	0.803905	-0.1115	0.903	0.831	1
Gdi2	0.729978	-0.11158	0.97	0.929	1
Papss1	0.000233	-0.1116	0.37	0.248	1
Fndc3a	0.000621	-0.11161	0.597	0.445	1
Fig4	0.087251	-0.11171	0.139	0.103	1
Itsn2	0.004553	-0.11182	0.403	0.296	1
Gnb2	0.069854	-0.11204	0.991	0.96	1
Ywhab	0.589902	-0.11212	0.942	0.876	1
Chic2	0.034602	-0.11219	0.709	0.555	1
Hsp90b1	0.092096	-0.1123	0.994	0.958	1
Dut	0.051508	-0.11235	0.355	0.27	1
Ndufa5	0.426585	-0.11252	0.9	0.794	1
Impdh2	0.207619	-0.11258	0.591	0.481	1
Trp53inp2	2.57E-05	-0.1128	0.345	0.222	0.509834
Gsdmd	0.002227	-0.11292	0.7	0.537	1
Arsb	0.010919	-0.11309	0.121	0.077	1
Fip1l1	0.000712	-0.11314	0.394	0.279	1
Snhg5	0.163237	-0.11315	0.233	0.187	1
Mif4gd	0.013675	-0.11321	0.57	0.436	1
Mrps33	0.560185	-0.11335	0.903	0.808	1
Flcn	1.77E-05	-0.11345	0.424	0.278	0.351612
Polr1c	0.000337	-0.11349	0.476	0.334	1
Mrpl32	0.003828	-0.11356	0.627	0.474	1
Csf1r	0.731081	-0.11365	0.991	0.978	1
Map1lc3a	0.133756	-0.11378	0.491	0.397	1
Csnk2b	0.304019	-0.1138	0.797	0.67	1
Tcf3	0.031275	-0.11405	0.503	0.394	1
Smek2	0.016442	-0.11416	0.418	0.318	1
Arf1	0.560495	-0.11416	0.979	0.925	1
Pdpk1	0.001982	-0.11417	0.315	0.219	1
Prkcsh	0.129818	-0.11419	0.706	0.587	1
Xrcc1	4.23E-05	-0.11432	0.315	0.197	0.840264
Cald1	0.61968	-0.11451	0.118	0.106	1
Supt4h1	0.828239	-0.11462	0.867	0.756	1
Rbm14	3.44E-05	-0.11473	0.285	0.173	0.683438

Rexo2	0.977307	-0.11476	0.9	0.837	1
C3	0.352468	-0.1148	0.73	0.623	1
Baiap2	0.032671	-0.11483	0.182	0.13	1
D4Wsu53e	0.921611	-0.11484	0.939	0.842	1
Mll3	0.000352	-0.11513	0.385	0.259	1
Lbr	0.003268	-0.1152	0.461	0.337	1
Dedd2	0.007872	-0.11526	0.224	0.157	1
Arglu1	0.11544	-0.11548	0.703	0.584	1
Ankrd44	4.70E-06	-0.11549	0.267	0.152	0.093331
Rpn2	0.761665	-0.11574	0.876	0.797	1
Rps21	0.53812	-0.11594	1	0.993	1
Pdcd6	0.801986	-0.11599	0.852	0.717	1
Mbd2	4.62E-06	-0.1161	0.682	0.486	0.091789
Fam46c	0.000296	-0.11617	0.303	0.205	1
Rpl5	0.056561	-0.11622	0.991	0.981	1
Ntan1	0.012524	-0.11623	0.642	0.496	1
Minos1	0.643948	-0.1165	0.967	0.929	1
Fxc1	0.41468	-0.11654	0.927	0.817	1
Ms4a4c	0.002366	-0.11663	0.585	0.436	1
Surf4	0.066378	-0.11666	0.87	0.722	1
1700019E1	6.31E-06	-0.11673	0.288	0.172	0.125383
Lypla1	0.05058	-0.11675	0.388	0.3	1
Hook3	0.005056	-0.1168	0.424	0.32	1
Rbm10	0.000159	-0.11683	0.279	0.18	1
Amfr	0.003634	-0.1169	0.715	0.547	1
Cyth4	0.027832	-0.11706	0.894	0.739	1
Zfand2b	0.002629	-0.11718	0.373	0.267	1
5830417I1	0.063161	-0.11725	0.124	0.089	1
Smc2	0.004385	-0.1174	0.318	0.23	1
Rbfox3	0.000166	-0.11755	0.276	0.179	1
Uqcrh	0.98778	-0.11755	0.979	0.957	1
Clk3	0.000108	-0.11763	0.497	0.341	1
Stk24	0.002392	-0.11776	0.648	0.47	1
Top1	0.533267	-0.11776	0.927	0.811	1
Neurl2	1.62E-05	-0.11788	0.203	0.113	0.321222
Pabpc4	0.001679	-0.11821	0.197	0.125	1
Dleu2	0.00127	-0.11826	0.645	0.498	1
Gmcl1	0.004943	-0.11827	0.161	0.103	1
Cdk8	0.00767	-0.11858	0.194	0.131	1
Nme2	0.159564	-0.11876	0.982	0.982	1
Ap2m1	0.963686	-0.11877	0.924	0.855	1
Cyth1	5.64E-06	-0.11891	0.573	0.381	0.112019
Mbnl2	0.000847	-0.11903	0.679	0.496	1

Csnk1a1	0.913263	-0.11928	0.748	0.652	1
Mrpl28	0.304212	-0.11929	0.727	0.609	1
Ifit3	0.029374	-0.11944	0.658	0.55	1
Tgfbr2	0.00764	-0.11957	0.627	0.487	1
Mycbp2	0.003016	-0.11966	0.785	0.642	1
Paics	0.037159	-0.11967	0.53	0.41	1
Cmklr1	0.02319	-0.1198	0.564	0.436	1
Tapbp	0.773173	-0.11982	0.927	0.864	1
Ibtk	0.01771	-0.1199	0.139	0.093	1
Syncrip	0.076239	-0.11993	0.467	0.369	1
Yipf5	0.028993	-0.12032	0.491	0.387	1
Arhgef6	1.71E-05	-0.12052	0.455	0.303	0.339652
Eif4a3	0.018137	-0.12064	0.588	0.458	1
Tgfb1	0.474472	-0.1207	0.979	0.917	1
Sash3	0.000311	-0.12079	0.488	0.333	1
Tbcb	0.286566	-0.1208	0.864	0.743	1
Mkrn2	0.025797	-0.12087	0.218	0.159	1
Man1a2	0.017639	-0.12103	0.318	0.232	1
Raf1	0.000608	-0.12116	0.539	0.388	1
Tcof1	0.001241	-0.12117	0.6	0.446	1
Serpinb8	0.00031	-0.12118	0.273	0.176	1
Uvrag	0.013071	-0.12126	0.445	0.342	1
AI462493	0.146771	-0.12146	0.727	0.607	1
Vrk3	1.84E-05	-0.12206	0.421	0.27	0.364992
Gsto1	0.778699	-0.12212	0.742	0.66	1
Cdca4	0.0056	-0.12218	0.173	0.113	1
Upf2	0.140576	-0.12224	0.227	0.182	1
Gtf2i	0.003576	-0.12233	0.309	0.213	1
Abcf2	0.033716	-0.12237	0.361	0.27	1
Ahcyl1	0.000152	-0.12249	0.391	0.27	1
Hspe1	0.836218	-0.12263	0.961	0.936	1
Arhgap9	0.041133	-0.12275	0.682	0.547	1
Ccnl2	0.081746	-0.12283	0.394	0.305	1
Pgm2	7.46E-05	-0.12285	0.355	0.233	1
Ppm1b	0.046952	-0.12316	0.258	0.193	1
Cd300lf	1.08E-05	-0.12318	0.718	0.517	0.214549
Mrpl50	0.003112	-0.12321	0.315	0.218	1
Kras	0.032916	-0.12332	0.824	0.671	1
Triobp	0.000555	-0.12352	0.467	0.324	1
Ppat	0.145182	-0.12356	0.112	0.085	1
Mdp1	0.001212	-0.12362	0.418	0.296	1
Lamtor3	0.015035	-0.12385	0.791	0.61	1
Cd44	0.663253	-0.12388	0.939	0.849	1

Tor2a	4.18E-06	-0.12399	0.558	0.368	0.082968
Edf1	0.573599	-0.12434	0.967	0.936	1
Cltc	0.910628	-0.12444	0.873	0.79	1
Lrrc33	0.155021	-0.12448	0.867	0.722	1
Asna1	0.031423	-0.12454	0.727	0.578	1
Cds2	0.020454	-0.1246	0.276	0.201	1
Ccdc125	0.019573	-0.12482	0.112	0.072	1
Dnm2	0.000372	-0.12529	0.748	0.567	1
2310003F1	0.669937	-0.12537	0.906	0.811	1
Csnk1g3	2.99E-05	-0.12547	0.452	0.299	0.594692
Gm6654	5.06E-05	-0.12553	0.43	0.281	1
Dynll1	0.062728	-0.12569	0.967	0.955	1
Ifi27l1	0.245313	-0.12575	0.712	0.597	1
Efr3a	0.000459	-0.12592	0.594	0.43	1
Ubap2l	0.019549	-0.12596	0.694	0.55	1
Vezf1	0.002117	-0.12597	0.279	0.192	1
Myo1c	0.00353	-0.12613	0.518	0.388	1
Zbtb7a	0.006867	-0.12617	0.685	0.521	1
Brp44	0.865765	-0.12639	0.876	0.78	1
Sgk1	0.509997	-0.1265	0.752	0.648	1
Gtf2h5	0.128118	-0.12651	0.785	0.666	1
Akap13	0.098118	-0.12651	0.57	0.459	1
Mettl7a1	0.003177	-0.12657	0.342	0.242	1
Adrm1	0.286504	-0.1266	0.715	0.608	1
Plac8	0.099401	-0.12665	0.876	0.831	1
Drg1	0.055802	-0.12686	0.57	0.451	1
H2-Ke6	0.002096	-0.12693	0.43	0.315	1
BC031181	0.894593	-0.12703	0.897	0.775	1
Mapk1	0.143138	-0.12721	0.567	0.462	1
Uqcrc2	0.032078	-0.12762	0.715	0.569	1
Tnfaip8l2	0.37355	-0.12767	0.776	0.676	1
Dstn	0.584335	-0.12779	0.706	0.602	1
Rbm22	0.039143	-0.12796	0.421	0.324	1
Ifngr1	0.355038	-0.12804	0.933	0.848	1
Mrps24	0.716597	-0.12812	0.909	0.807	1
Irf2	0.215065	-0.12828	0.752	0.608	1
Loh12cr1	4.33E-05	-0.12829	0.312	0.196	0.859432
Rnf14	0.047718	-0.12835	0.37	0.286	1
Urod	0.537831	-0.12851	0.191	0.166	1
AA960436	0.000428	-0.12853	0.364	0.252	1
Ctsd	0.070486	-0.12869	0.994	0.996	1
Cox16	0.098156	-0.12879	0.658	0.525	1
Ncoa3	0.233801	-0.12881	0.527	0.427	1

Dapk1	0.008583	-0.12883	0.494	0.376	1
E130102H:	0.000158	-0.1289	0.145	0.078	1
Tmbim1	0.001419	-0.12892	0.403	0.291	1
Ddx39	0.054854	-0.12913	0.406	0.321	1
Hnrnpc	0.623693	-0.12926	0.858	0.737	1
Lass5	0.005296	-0.12933	0.491	0.371	1
Kdsr	0.241409	-0.12971	0.167	0.136	1
Rcc2	5.87E-06	-0.12983	0.6	0.407	0.116522
Eif1ax	0.012789	-0.12985	0.658	0.508	1
Mapre1	0.0442	-0.12989	0.824	0.655	1
Wbp4	0.009077	-0.12989	0.639	0.506	1
Bbx	0.000655	-0.13006	0.6	0.441	1
Xrn2	0.024741	-0.13008	0.694	0.544	1
Ddx6	0.001543	-0.13012	0.648	0.492	1
Mospd3	0.158765	-0.13014	0.452	0.355	1
Actr1a	0.00063	-0.13019	0.567	0.409	1
1500003O	0.007579	-0.13022	0.524	0.399	1
Cpd	0.001102	-0.13028	0.27	0.179	1
Ndufb10	0.808377	-0.13037	0.894	0.808	1
Rnf6	0.000189	-0.13038	0.361	0.24	1
Prdx3	0.063901	-0.13048	0.485	0.386	1
Atf7ip	0.007732	-0.13059	0.2	0.139	1
Dhx15	0.001046	-0.13065	0.561	0.41	1
Rab4b	0.009877	-0.13066	0.727	0.561	1
Dazap1	0.072151	-0.13066	0.588	0.472	1
Prrg2	6.83E-05	-0.13072	0.276	0.17	1
Stap2	0.000657	-0.13088	0.188	0.115	1
Sept7	0.114834	-0.1309	0.77	0.633	1
Gkap1	0.003504	-0.13095	0.364	0.26	1
Dgcr6	0.117048	-0.13129	0.418	0.34	1
Tmem219	0.029567	-0.13131	0.855	0.704	1
Sf3b3	0.000563	-0.13139	0.445	0.312	1
Oat	0.015556	-0.13158	0.636	0.486	1
Cux1	0.003156	-0.13181	0.364	0.26	1
Ptpn2	2.20E-05	-0.13184	0.552	0.381	0.435961
Itfg2	0.031081	-0.13188	0.1	0.065	1
Med30	0.012193	-0.13188	0.606	0.462	1
Bag1	0.994736	-0.132	0.891	0.824	1
Usp2	0.000226	-0.13202	0.248	0.158	1
Zmpste24	0.057962	-0.13211	0.336	0.265	1
F630111L1	0.038142	-0.13214	0.103	0.069	1
Fam3b	4.26E-06	-0.13215	0.221	0.12	0.084569
Ost4	0.656612	-0.13218	0.952	0.915	1

Eif4enif1	0.000508	-0.13219	0.248	0.157	1
Top2b	0.000932	-0.13264	0.436	0.307	1
Max	0.055582	-0.13269	0.794	0.641	1
Vsig4	0.145824	-0.13285	0.997	0.985	1
Ssbp4	0.489597	-0.13287	0.882	0.764	1
Cfh	0.37012	-0.13289	0.655	0.551	1
Ankrd46	0.002816	-0.1329	0.197	0.13	1
Rbfa	0.039128	-0.13301	0.852	0.707	1
G6pdx	0.003505	-0.13303	0.739	0.591	1
Bin3	0.00892	-0.1331	0.73	0.579	1
Dlc1	0.000352	-0.13335	0.209	0.128	1
Psmc6	0.004916	-0.13336	0.624	0.474	1
Ndufs2	0.571182	-0.13347	0.761	0.671	1
Sfrs18	0.109002	-0.13392	0.612	0.503	1
Hnrnph1	0.08858	-0.13397	0.427	0.333	1
Lypla2	0.53722	-0.13402	0.497	0.432	1
Tbk1	0.000343	-0.1344	0.436	0.298	1
D17Wsu92	0.002402	-0.13441	0.639	0.48	1
Fam43a	0.080826	-0.13471	0.285	0.217	1
Luc7l3	0.050232	-0.13508	0.63	0.511	1
Cox7a2	0.524645	-0.13516	0.982	0.954	1
Tnfrsf1b	0.02453	-0.13523	0.664	0.542	1
Stra13	0.151389	-0.13534	0.87	0.737	1
Spsb3	0.005157	-0.13538	0.288	0.206	1
2210016F1	0.00048	-0.13563	0.809	0.632	1
Anxa5	0.131288	-0.13571	0.958	0.902	1
Laptm4a	0.045009	-0.13581	0.985	0.948	1
Fmr1	0.009561	-0.13583	0.37	0.272	1
Tardbp	0.002046	-0.13594	0.624	0.481	1
Pmm1	9.06E-06	-0.13604	0.406	0.256	0.180017
Tomm7	0.510118	-0.13623	0.964	0.917	1
Rap1b	0.072094	-0.13639	0.991	0.962	1
Alad	0.032723	-0.13641	0.303	0.227	1
Fads1	0.000296	-0.13646	0.479	0.348	1
Bri3bp	0.000271	-0.13661	0.145	0.079	1
Cenpi	0.000108	-0.13665	0.115	0.057	1
Ptp4a2	0.315046	-0.13671	0.948	0.915	1
Eif4h	0.447895	-0.13683	0.845	0.707	1
Uqcrc1	0.701206	-0.13686	0.827	0.719	1
Arl2	0.000256	-0.13688	0.491	0.351	1
Hdac5	0.000156	-0.13694	0.373	0.255	1
Kpna2	0.343805	-0.13707	0.37	0.309	1
Csf2ra	0.461548	-0.13709	0.876	0.764	1

Krtcap2	0.52586	-0.13722	0.933	0.895	1
Slc35e4	0.124106	-0.13729	0.115	0.086	1
Ndufs4	0.39011	-0.13736	0.83	0.705	1
Tial1	0.000878	-0.13748	0.57	0.417	1
Glipr2	0.036778	-0.13755	0.17	0.121	1
Psme1	0.347313	-0.13764	0.982	0.95	1
Gcnt1	0.042269	-0.13768	0.17	0.123	1
C1qa	0.00024	-0.1377	0.997	0.993	1
Mdh1	0.816923	-0.13771	0.879	0.778	1
Sh3kbp1	0.002247	-0.13777	0.645	0.479	1
Ndufa9	0.364281	-0.1378	0.755	0.621	1
Adprh	0.001293	-0.13783	0.7	0.546	1
Slc25a17	0.002803	-0.13784	0.364	0.262	1
Polr2f	0.546228	-0.13788	0.906	0.816	1
Fkbp8	0.659315	-0.13791	0.921	0.804	1
Arpc1a	0.628423	-0.13793	0.748	0.634	1
Dscr3	0.000775	-0.13813	0.545	0.407	1
Nol7	0.08673	-0.13813	0.658	0.542	1
Npm3	0.010039	-0.13813	0.442	0.335	1
Cd97	0.674794	-0.13818	0.942	0.866	1
Creb5	0.032837	-0.13824	0.179	0.129	1
Psmb7	0.494802	-0.13824	0.794	0.688	1
Pkn1	0.000126	-0.13835	0.585	0.419	1
Tef	0.003591	-0.13847	0.2	0.132	1
Csnk1g2	2.79E-06	-0.13854	0.509	0.341	0.055473
Atxn2l	0.014358	-0.13882	0.488	0.368	1
Ubxn6	0.013513	-0.1389	0.636	0.498	1
Pcna	0.012106	-0.13918	0.8	0.642	1
Lrrfip2	0.000646	-0.13926	0.424	0.298	1
Sectm1a	0.002439	-0.13932	0.385	0.277	1
Zranb2	0.002196	-0.13934	0.588	0.44	1
Psmd1	0.051314	-0.13937	0.688	0.553	1
Rpp21	0.003611	-0.13937	0.752	0.595	1
Lrch3	0.073483	-0.13938	0.209	0.159	1
Mrpl30	0.060376	-0.13945	0.827	0.691	1
Serhl	5.14E-05	-0.13947	0.494	0.345	1
Dip2b	0.004679	-0.13947	0.521	0.399	1
Sft2d1	0.392341	-0.13947	0.873	0.772	1
Crip1	0.054569	-0.13964	0.958	0.937	1
2610029G:	0.000874	-0.13989	0.555	0.404	1
Crot	0.501866	-0.1399	0.158	0.136	1
Vps26a	0.037211	-0.1399	0.676	0.533	1
1700123O	0.010084	-0.13992	0.497	0.38	1

Sf3a1	0.043432	-0.13996	0.255	0.192	1
Snrpb2	0.057851	-0.13998	0.833	0.691	1
Taf13	0.009723	-0.14001	0.345	0.254	1
Dus3l	0.473066	-0.14012	0.158	0.138	1
Nrd1	0.003893	-0.1403	0.515	0.387	1
Srsf5	0.743472	-0.14034	0.924	0.81	1
Susd3	0.029188	-0.14036	0.333	0.256	1
Srpr	5.05E-05	-0.1406	0.658	0.459	1
Mrps17	0.001723	-0.14082	0.712	0.536	1
Tbc1d17	0.000237	-0.14091	0.355	0.236	1
Atpif1	0.284686	-0.14094	0.982	0.957	1
Irf5	0.298679	-0.14114	0.918	0.791	1
Ifitm1	0.102298	-0.14119	0.161	0.122	1
Ndufa7	0.445766	-0.14121	0.964	0.923	1
Pin4	0.059282	-0.14122	0.836	0.684	1
Cox17	0.856969	-0.14139	0.936	0.886	1
Calu	0.008116	-0.14155	0.606	0.464	1
Daxx	0.001077	-0.1416	0.573	0.423	1
Usp22	0.000247	-0.14181	0.245	0.154	1
Zmynd11	0.005458	-0.14183	0.5	0.373	1
Cox7c	0.19795	-0.14191	0.994	0.989	1
Tmem85	0.000729	-0.14201	0.667	0.501	1
Smurf2	0.003119	-0.14229	0.273	0.189	1
Erdr1	1.31E-05	-0.14231	0.6	0.42	0.261115
Nutf2	0.005893	-0.14235	0.118	0.072	1
Fundc1	0.000131	-0.14238	0.742	0.57	1
Cd53	0.026882	-0.14247	0.976	0.93	1
Wdr33	0.000249	-0.14248	0.603	0.427	1
Arpc2	0.002229	-0.1425	1	0.994	1
Srp14	0.747628	-0.14251	0.936	0.878	1
Arpc4	0.278167	-0.14253	0.985	0.93	1
Calr	0.016351	-0.14256	0.991	0.981	1
Fbxl5	0.035555	-0.14263	0.4	0.312	1
Myst3	1.12E-05	-0.14269	0.433	0.287	0.221677
Rab35	0.00725	-0.14275	0.436	0.331	1
Yrdc	0.008632	-0.14299	0.503	0.388	1
Vps25	0.006211	-0.14309	0.609	0.47	1
Rsl1d1	0.00302	-0.14321	0.585	0.423	1
Trappc5	0.000436	-0.14325	0.636	0.474	1
Crbn	0.003288	-0.14326	0.376	0.275	1
U2af2	0.129072	-0.14328	0.733	0.583	1
Klhl21	0.028504	-0.14369	0.118	0.079	1
Psmb3	0.516166	-0.14383	0.918	0.849	1

Pdcd11	6.25E-05	-0.14394	0.382	0.251	1
Ppm1g	0.003784	-0.14395	0.661	0.507	1
Ctbp1	0.116056	-0.14429	0.779	0.64	1
Pnpla2	0.049246	-0.14438	0.658	0.521	1
Cxx1b	0.002724	-0.14452	0.37	0.261	1
Cct8	0.718836	-0.14468	0.848	0.748	1
Pum1	0.001463	-0.14475	0.373	0.262	1
Ttc33	6.34E-05	-0.14498	0.327	0.212	1
Als2cr4	0.017542	-0.14502	0.17	0.116	1
Cnp	0.068175	-0.14504	0.552	0.445	1
Abtb1	0.002501	-0.14543	0.33	0.236	1
Tet3	7.54E-05	-0.14551	0.479	0.334	1
Nemf	0.062762	-0.14561	0.388	0.301	1
Khdrbs1	0.473493	-0.14567	0.742	0.636	1
Ssbp1	0.00817	-0.14567	0.694	0.531	1
Rnf187	0.044744	-0.14584	0.839	0.681	1
Usp4	0.015094	-0.14585	0.397	0.294	1
Klf16	3.38E-05	-0.14588	0.191	0.106	0.670966
Terf1	0.01204	-0.14603	0.215	0.153	1
Faf1	5.71E-05	-0.14626	0.436	0.296	1
Spin1	0.027834	-0.1465	0.403	0.308	1
Prpf40a	0.015335	-0.14658	0.615	0.477	1
Srrm2	0.506929	-0.14662	0.93	0.833	1
Cklf	0.204173	-0.14666	0.703	0.586	1
Pmf1	0.03608	-0.14667	0.821	0.717	1
Spryd3	1.53E-05	-0.14681	0.361	0.232	0.303875
Luc7l	0.004692	-0.14692	0.361	0.269	1
Ccdc88a	0.012692	-0.14693	0.415	0.327	1
Slc15a3	0.024244	-0.14698	0.958	0.874	1
Snrpf	0.560944	-0.14705	0.879	0.816	1
Tcp1	0.663005	-0.14725	0.788	0.663	1
Rfc2	0.002428	-0.14746	0.452	0.333	1
Pcmtd1	0.000131	-0.14749	0.494	0.342	1
Comt	0.745012	-0.14755	0.952	0.866	1
Vdac1	0.94099	-0.14756	0.806	0.691	1
Taldo1	0.048571	-0.14761	0.979	0.951	1
Spcs1	0.619979	-0.14768	0.93	0.868	1
Taf1d	0.002213	-0.14768	0.448	0.32	1
Rwdd4a	0.001941	-0.1477	0.394	0.284	1
Ralbp1	0.255319	-0.14772	0.773	0.653	1
Cul3	0.003291	-0.14787	0.327	0.233	1
Capza2	0.387246	-0.14816	0.958	0.908	1
1810030N[illegible]	0.000961	-0.14822	0.273	0.181	1

Cnksr3	0.000325	-0.14829	0.145	0.081	1
Vps35	0.125892	-0.14854	0.791	0.644	1
Rpl31-ps12	0.722622	-0.14858	0.9	0.81	1
Med21	0.052691	-0.14861	0.627	0.507	1
Dnajb11	0.180665	-0.14865	0.821	0.684	1
Fkbp2	0.226421	-0.14872	0.918	0.867	1
Incenp	0.000321	-0.14896	0.342	0.234	1
Plxna1	3.24E-06	-0.14907	0.473	0.316	0.064306
Ostf1	0.493067	-0.14932	0.976	0.93	1
Dtymk	0.008231	-0.14951	0.509	0.389	1
Tmem30a	0.008288	-0.1498	0.712	0.538	1
Rp2h	5.48E-05	-0.14982	0.621	0.437	1
Igtp	0.006102	-0.14982	0.515	0.39	1
Ctnnbip1	0.396842	-0.15032	0.639	0.531	1
Srsf3	0.962144	-0.15034	0.885	0.808	1
Lztfl1	0.040622	-0.15036	0.164	0.117	1
Txn1	0.052074	-0.15041	0.997	0.994	1
Tubb4b	0.035343	-0.15042	0.476	0.361	1
Ncor1	0.026727	-0.15053	0.761	0.579	1
Capzb	0.026914	-0.15075	0.988	0.967	1
Nudt16l1	0.209801	-0.15085	0.309	0.249	1
Gpbp1	0.046426	-0.1509	0.485	0.375	1
Bub3	0.091599	-0.15113	0.67	0.535	1
Rrp7a	0.000105	-0.15117	0.697	0.498	1
Ppib	0.476319	-0.15117	0.942	0.905	1
Siva1	0.045157	-0.15117	0.624	0.51	1
Cmtm7	0.31947	-0.1513	0.955	0.902	1
Cbfb	0.000488	-0.15151	0.739	0.532	1
Ncdn	0.038752	-0.15155	0.121	0.084	1
Ddx54	0.007403	-0.1517	0.512	0.38	1
Plekhg2	0.075331	-0.15178	0.106	0.075	1
Lsm2	0.472759	-0.1519	0.539	0.457	1
Ift46	0.002466	-0.15194	0.215	0.143	1
Gbp7	0.00074	-0.15195	0.655	0.486	1
Txndc5	0.040047	-0.15211	0.536	0.404	1
Eprs	0.040309	-0.15222	0.506	0.404	1
Rab21	5.35E-06	-0.15223	0.239	0.136	0.106169
Ube2g1	0.002335	-0.15229	0.467	0.327	1
Dnaja1	0.440276	-0.15231	0.936	0.852	1
Nfia	0.085697	-0.15235	0.252	0.193	1
Rbm18	0.055052	-0.15245	0.215	0.164	1
Psmd13	0.587448	-0.15246	0.639	0.544	1
Fus	0.624635	-0.15291	0.721	0.615	1

Mpnd	0.005715	-0.15318	0.448	0.342	1
Fis1	0.195478	-0.15322	0.912	0.812	1
Dynlrb1	0.7217	-0.15329	0.912	0.846	1
Rexo4	0.030316	-0.15332	0.333	0.254	1
Snapin	0.006857	-0.15343	0.485	0.357	1
Plekha1	0.059108	-0.15344	0.258	0.207	1
Blvra	0.009672	-0.15344	0.879	0.755	1
Cd38	0.194486	-0.15352	0.927	0.831	1
Mocs2	0.009875	-0.15375	0.645	0.483	1
Denr	0.088985	-0.15378	0.776	0.667	1
Rlf	6.02E-05	-0.15381	0.185	0.104	1
Bach2	0.186652	-0.15389	0.106	0.081	1
Parp14	0.001316	-0.15407	0.694	0.517	1
Cast	0.02942	-0.15427	0.212	0.155	1
Fas	0.50081	-0.15449	0.136	0.118	1
Nsa2	0.565296	-0.15469	0.782	0.678	1
Blcap	0.000261	-0.15484	0.285	0.185	1
1810026B(	0.008574	-0.15492	0.503	0.372	1
Mbd3	0.141341	-0.15493	0.773	0.635	1
Bok	3.40E-05	-0.15495	0.148	0.077	0.675307
Wls	0.061127	-0.1552	0.345	0.272	1
Ccni	0.116284	-0.15526	0.703	0.572	1
Rheb	0.66046	-0.15528	0.873	0.763	1
Mdh2	0.408108	-0.15531	0.879	0.789	1
Tceb3	0.062716	-0.15535	0.533	0.432	1
Ndufa11	0.903855	-0.15538	0.882	0.759	1
AI662270	0.070012	-0.15539	0.839	0.678	1
Jtb	0.456562	-0.15553	0.785	0.656	1
Eif3g	0.592012	-0.15565	0.852	0.74	1
Fam63b	0.000621	-0.15573	0.582	0.413	1
Pkig	0.615079	-0.15576	0.855	0.74	1
Slc12a4	0.003088	-0.15576	0.164	0.105	1
Acadm	0.051897	-0.15578	0.339	0.263	1
Thap3	0.008975	-0.15591	0.345	0.252	1
Ech1	0.090317	-0.15599	0.709	0.557	1
Zfp330	0.006148	-0.15603	0.552	0.413	1
Rnf34	0.011322	-0.15606	0.327	0.24	1
Eif2ak1	0.000204	-0.15609	0.336	0.225	1
Dhrs3	0.234093	-0.1561	0.718	0.61	1
Bms1	3.85E-06	-0.15618	0.348	0.216	0.076535
Slco3a1	4.41E-05	-0.1562	0.364	0.241	0.876048
Pcnp	0.000794	-0.1562	0.648	0.479	1
St3gal1	5.68E-05	-0.15622	0.615	0.43	1

Cdc42se2	0.005817	-0.15624	0.73	0.565	1
Ascc1	0.016801	-0.15635	0.309	0.23	1
Trim44	0.014088	-0.15637	0.339	0.251	1
Atf5	0.000258	-0.15637	0.376	0.249	1
Tle3	0.003915	-0.15648	0.242	0.166	1
Chrac1	0.033262	-0.15649	0.797	0.657	1
Pwwp2b	4.78E-06	-0.15649	0.373	0.234	0.094979
Snrnp27	0.009456	-0.15679	0.77	0.601	1
Mrp63	0.256281	-0.15684	0.818	0.702	1
Nktr	0.002814	-0.15685	0.518	0.383	1
Cnih4	0.068211	-0.15688	0.782	0.655	1
Map2k1	6.92E-05	-0.15688	0.606	0.432	1
Smap2	0.026685	-0.15693	0.682	0.529	1
Prrc2b	0.005253	-0.15725	0.248	0.174	1
Bcas2	0.541948	-0.15732	0.748	0.617	1
Tm7sf3	0.039133	-0.15735	0.309	0.234	1
Mia3	0.013029	-0.15758	0.409	0.312	1
Mff	0.263687	-0.15765	0.652	0.547	1
Msra	0.037151	-0.15774	0.245	0.184	1
Ube2l6	0.236817	-0.15787	0.715	0.598	1
Gm9320	0.021135	-0.158	0.594	0.474	1
Mnda	0.032833	-0.15802	0.776	0.612	1
Pdlim2	0.002914	-0.15826	0.464	0.331	1
Surf1	0.002642	-0.15826	0.509	0.379	1
Ncf2	0.199763	-0.15833	0.833	0.702	1
Srsf9	0.15101	-0.15843	0.873	0.738	1
Polr2j	0.142474	-0.1585	0.8	0.67	1
Anp32e	0.052214	-0.15851	0.521	0.412	1
Obfc2b	0.000192	-0.15853	0.642	0.477	1
Crip2	0.9459	-0.15856	0.479	0.422	1
Parp4	0.050599	-0.15861	0.2	0.152	1
Atg7	0.023199	-0.15868	0.409	0.314	1
Prps1	0.008376	-0.15869	0.273	0.198	1
Arrdc4	0.001593	-0.15873	0.415	0.305	1
Slc35c2	0.014004	-0.15876	0.639	0.497	1
Apoe	3.83E-05	-0.1588	1	1	0.760722
Eea1	0.001498	-0.15893	0.539	0.393	1
Sri	0.869601	-0.15907	0.867	0.741	1
Ptplb	0.00214	-0.15909	0.448	0.328	1
Abhd5	0.037184	-0.15922	0.273	0.205	1
Arrb2	0.6102	-0.15932	0.927	0.843	1
Serinc1	0.377635	-0.1594	0.761	0.638	1
Arid2	8.00E-05	-0.15945	0.294	0.181	1

U2af1	0.698354	-0.15947	0.727	0.63	1
4833420G	0.037634	-0.15964	0.218	0.166	1
Mrpl23	0.490986	-0.15968	0.93	0.829	1
Trmt112	0.478795	-0.15969	0.87	0.758	1
Vps37a	5.29E-06	-0.15975	0.467	0.31	0.105047
Rsu1	0.159706	-0.15975	0.688	0.563	1
Cybasc3	0.600687	-0.15979	0.112	0.099	1
Ubac2	0.002073	-0.15982	0.552	0.409	1
Tab2	0.015578	-0.15988	0.648	0.504	1
Cdc123	0.008177	-0.1599	0.561	0.432	1
Klra2	0.03962	-0.16001	0.848	0.7	1
Elovl5	0.22459	-0.16007	0.427	0.351	1
Psmd2	0.004547	-0.16015	0.709	0.564	1
Eri3	0.000695	-0.16021	0.518	0.387	1
Taok3	0.009632	-0.16055	0.585	0.454	1
Txndc9	0.056445	-0.16059	0.615	0.488	1
Gatad2a	0.000173	-0.16061	0.6	0.434	1
Nmd3	0.005521	-0.16066	0.358	0.265	1
Cdc5l	0.018932	-0.16067	0.506	0.39	1
Krcc1	0.143552	-0.1607	0.788	0.658	1
Itgb1bp1	0.007676	-0.16085	0.582	0.447	1
Abcg2	0.307362	-0.16095	0.252	0.207	1
Rpl36al	0.110461	-0.16117	0.988	0.991	1
Cnpy2	0.707576	-0.16129	0.818	0.711	1
Aip	0.160508	-0.16142	0.606	0.488	1
Cdk2	0.018509	-0.16147	0.242	0.175	1
Clns1a	0.00322	-0.16153	0.567	0.426	1
Clec4a3	0.784152	-0.16162	0.93	0.821	1
Atl2	0.002856	-0.16167	0.152	0.094	1
Guk1	0.008758	-0.16173	0.755	0.582	1
Ywhae	0.395061	-0.16173	0.961	0.885	1
Neu1	0.151097	-0.16173	0.836	0.698	1
Arhgap5	0.002938	-0.16192	0.273	0.189	1
Arih1	9.45E-05	-0.16194	0.439	0.298	1
Tbc1d15	7.62E-06	-0.16204	0.364	0.228	0.15143
Utp11l	0.00212	-0.16217	0.561	0.418	1
Nfya	6.52E-06	-0.16219	0.37	0.231	0.129545
Iah1	0.042082	-0.16235	0.509	0.389	1
Brd4	0.000157	-0.16236	0.594	0.415	1
Samd4b	0.001031	-0.1625	0.464	0.324	1
Rbms1	0.017588	-0.16252	0.788	0.619	1
9130401M	2.71E-06	-0.16258	0.679	0.473	0.053762
Mlec	0.006727	-0.16271	0.515	0.384	1

Snrpd2	0.929673	-0.16299	0.87	0.806	1
Tspan7	0.001634	-0.16305	0.155	0.093	1
4931406P1	0.000819	-0.16333	0.206	0.128	1
Ebi3	0.001974	-0.16336	0.782	0.622	1
Ccdc72	0.3834	-0.16349	0.982	0.96	1
Rnf138	0.002083	-0.16356	0.548	0.405	1
2310022A1	9.23E-05	-0.16361	0.361	0.241	1
Cwc15	0.738018	-0.16363	0.894	0.81	1
Pebp1	0.900545	-0.16365	0.891	0.822	1
5430435G1	1.24E-05	-0.16368	0.703	0.526	0.245542
1110001J0	0.015473	-0.16369	0.664	0.508	1
Epb4.1	0.000122	-0.16373	0.33	0.213	1
Zfp644	0.125678	-0.16381	0.152	0.117	1
Anapc1	0.02407	-0.16384	0.239	0.176	1
4732418C0	5.54E-05	-0.16385	0.376	0.246	1
Man2b1	0.634867	-0.16397	0.973	0.925	1
Gpx3	0.154618	-0.16397	0.358	0.351	1
Uhrf1bp1l	0.000285	-0.16398	0.245	0.154	1
Dnajc15	0.781705	-0.16409	0.921	0.837	1
Aldh2	0.006291	-0.16418	0.979	0.945	1
Apobec1	0.000308	-0.16421	0.912	0.769	1
Bnip2	0.599753	-0.16426	0.764	0.653	1
Baz2a	0.002027	-0.16429	0.427	0.303	1
Ifi27l2a	0.014066	-0.16457	0.988	0.981	1
Cd164	0.039968	-0.16457	0.948	0.89	1
Cdc37	0.947365	-0.16457	0.897	0.792	1
Mrpl24	0.248333	-0.1646	0.655	0.531	1
Smu1	0.032233	-0.16462	0.448	0.342	1
Nap1l4	0.378101	-0.16463	0.47	0.399	1
H2afy	0.645101	-0.16465	0.815	0.718	1
Reps1	6.81E-05	-0.16491	0.255	0.158	1
Vcp	0.536393	-0.16494	0.876	0.8	1
Acin1	0.058635	-0.16507	0.615	0.488	1
C1qc	5.53E-05	-0.1651	0.997	0.993	1
Sfpi1	0.01321	-0.16521	0.979	0.959	1
Cbx1	0.005131	-0.16529	0.33	0.243	1
Snn	0.068631	-0.1653	0.145	0.107	1
St8sia4	0.000728	-0.16535	0.158	0.092	1
Preb	0.124777	-0.16539	0.539	0.447	1
Ccdc6	0.017742	-0.16542	0.221	0.16	1
Ywhah	0.515513	-0.16546	0.939	0.872	1
Ciao1	0.000106	-0.16551	0.345	0.227	1
Cct4	0.792863	-0.16554	0.836	0.751	1

Abhd16a	0.020378	-0.16557	0.542	0.419	1
BC004004	0.042093	-0.16559	0.779	0.633	1
Epha2	5.07E-05	-0.16565	0.173	0.094	1
Cnot2	0.011566	-0.16567	0.391	0.293	1
Tmem59	0.11645	-0.16568	0.942	0.875	1
Gpr180	1.09E-05	-0.16577	0.339	0.214	0.216486
Brms1	2.62E-05	-0.16584	0.57	0.391	0.519496
Setd3	0.004134	-0.16597	0.509	0.371	1
Pdcd4	0.081818	-0.166	0.239	0.181	1
Tomm70a	0.020955	-0.16603	0.436	0.327	1
Vdac2	0.105452	-0.16606	0.936	0.886	1
Dbi	0.291527	-0.16609	0.964	0.912	1
Stub1	0.254028	-0.16614	0.818	0.665	1
Higd2a	0.387558	-0.16645	0.852	0.729	1
Arpc5	0.042824	-0.16648	0.985	0.944	1
Htra2	0.134658	-0.16658	0.512	0.434	1
Actr2	0.78343	-0.16659	0.918	0.824	1
Fam32a	0.95137	-0.16667	0.839	0.731	1
Cul1	0.15312	-0.16668	0.652	0.56	1
Apbb1ip	0.159707	-0.16688	0.8	0.662	1
Rer1	0.521103	-0.167	0.9	0.802	1
Arf3	0.011463	-0.16701	0.861	0.732	1
Slmo2	0.010297	-0.16709	0.703	0.545	1
Dpm3	0.240443	-0.16711	0.885	0.758	1
Bzw2	0.039117	-0.1672	0.176	0.128	1
Sec61g	0.249852	-0.1678	0.976	0.972	1
Ly86	0.067188	-0.16792	0.985	0.948	1
2510039O	1.41E-05	-0.16796	0.603	0.421	0.279566
Cxx1a	0.013983	-0.16798	0.255	0.184	1
Psmb1	0.074452	-0.16808	0.93	0.862	1
Ndufb7	0.217016	-0.16819	0.958	0.9	1
Set	0.970944	-0.16825	0.894	0.803	1
Ap1s1	0.017405	-0.16827	0.752	0.606	1
Rbm5	0.000367	-0.16847	0.403	0.275	1
Znhit6	0.002465	-0.16848	0.215	0.141	1
Mrpl48	0.004341	-0.16879	0.597	0.448	1
Lrrc25	0.201753	-0.16884	0.945	0.878	1
Hcfc1r1	0.501626	-0.16885	0.836	0.692	1
Zc3h15	0.020876	-0.169	0.585	0.458	1
5031425E2	0.005142	-0.16919	0.303	0.214	1
Rnaseh2a	0.003318	-0.16923	0.448	0.335	1
Notch2	0.081396	-0.16926	0.306	0.24	1
Rabgap1	0.01245	-0.1693	0.167	0.114	1

Zfp451	0.003263	-0.16937	0.115	0.067	1
Plekhg6	0.062963	-0.16938	0.127	0.093	1
Tmem93	0.537183	-0.16947	0.839	0.706	1
Elavl1	0.158827	-0.16959	0.706	0.584	1
1300014I0	0.044254	-0.16966	0.448	0.338	1
Tmem88	2.18E-05	-0.1697	0.236	0.141	0.433189
Myl6	0.047801	-0.16976	1	0.99	1
Tmem147	0.978618	-0.16984	0.827	0.717	1
Gng5	0.027275	-0.16985	0.997	0.993	1
Ptbp1	0.135527	-0.16994	0.712	0.604	1
Metap2	0.732688	-0.16995	0.894	0.805	1
Rtp4	0.633074	-0.16997	0.897	0.829	1
Psmc2	0.094109	-0.17031	0.712	0.565	1
Pfas	0.036644	-0.17032	0.103	0.068	1
Nfyc	0.001248	-0.17066	0.33	0.231	1
Fiz1	0.358102	-0.17071	0.173	0.145	1
Eif4g2	0.792052	-0.17075	0.888	0.803	1
Ankrd17	0.001322	-0.17094	0.458	0.332	1
Ccdc34	3.03E-05	-0.17133	0.436	0.289	0.602022
Huwe1	0.006251	-0.17138	0.479	0.361	1
Ttc14	0.035234	-0.17145	0.5	0.393	1
Psmd11	0.414296	-0.17168	0.57	0.48	1
Fermt3	0.144709	-0.17183	0.861	0.729	1
Usp8	0.060429	-0.172	0.588	0.454	1
Tapbpl	0.001931	-0.17202	0.424	0.311	1
Hdac3	0.011044	-0.17203	0.452	0.342	1
0910001L(	0.856746	-0.17218	0.864	0.775	1
Rraga	0.068649	-0.17223	0.452	0.361	1
Pdcd10	0.19662	-0.17235	0.755	0.612	1
Isoc2b	0.137083	-0.17235	0.203	0.163	1
Kbtbd2	0.064223	-0.17251	0.33	0.251	1
0610037P(	0.001379	-0.17251	0.288	0.197	1
Fcho2	0.004424	-0.17265	0.442	0.326	1
Camta1	0.000274	-0.17268	0.588	0.418	1
Gpsm3	0.25985	-0.17281	0.918	0.835	1
Twf2	0.013564	-0.17287	0.73	0.563	1
Cmpk1	0.874555	-0.17295	0.906	0.799	1
Rps15a-ps(	0.500441	-0.173	0.864	0.763	1
Nans	0.001067	-0.17327	0.688	0.527	1
Srsf6	0.767591	-0.17337	0.755	0.634	1
Bptf	0.00904	-0.17355	0.476	0.36	1
Ndufa8	0.767871	-0.17361	0.897	0.798	1
Dhodh	2.13E-05	-0.1737	0.212	0.12	0.422754

0610031J0	0.560573	-0.17386	0.833	0.701	1
Rab6a	0.150331	-0.17387	0.555	0.433	1
Ethe1	0.410673	-0.17416	0.321	0.274	1
Gtf2a2	0.166174	-0.17419	0.715	0.591	1
Kitl	0.009594	-0.17428	0.173	0.115	1
Pttg1ip	0.167893	-0.1743	0.597	0.482	1
4931406C(	0.052063	-0.17432	0.367	0.287	1
Abhd12	0.457271	-0.17435	0.952	0.83	1
Usp1	0.014637	-0.17463	0.409	0.308	1
Rfc4	0.019776	-0.17466	0.179	0.124	1
Ddx41	6.05E-06	-0.17471	0.439	0.287	0.120209
Nfix	0.00025	-0.17479	0.212	0.13	1
Psmd10	0.001533	-0.17481	0.679	0.52	1
Dnajb9	0.174924	-0.175	0.461	0.371	1
Sod2	0.340844	-0.17516	0.861	0.788	1
Raly	0.683923	-0.17522	0.897	0.75	1
Polr2g	0.073913	-0.17525	0.824	0.687	1
Tm2d3	0.008313	-0.17526	0.491	0.374	1
Pdap1	0.066973	-0.1755	0.773	0.612	1
Kank3	0.00201	-0.17606	0.109	0.06	1
Rod1	0.292132	-0.17611	0.83	0.708	1
Cstf3	0.007576	-0.17631	0.291	0.206	1
4930420K1	0.017313	-0.17636	0.47	0.361	1
Stim2	0.000979	-0.17644	0.321	0.221	1
Prkrir	0.001386	-0.17654	0.336	0.23	1
Smad1	0.009877	-0.17661	0.536	0.409	1
1110004F1	0.350779	-0.17664	0.676	0.583	1
Cript	0.088425	-0.17666	0.773	0.64	1
Eif5b	0.887519	-0.17675	0.952	0.868	1
Phb	0.971958	-0.1769	0.794	0.707	1
Prkar1a	0.278601	-0.17693	0.755	0.637	1
Anapc13	0.468411	-0.17695	0.809	0.719	1
Prorsd1	0.279974	-0.17698	0.436	0.367	1
1810046J1	0.478264	-0.17702	0.845	0.766	1
Dnajc9	0.018161	-0.17703	0.382	0.287	1
Upp1	0.011891	-0.17708	0.1	0.061	1
Trim47	0.001494	-0.1771	0.53	0.396	1
Phyhd1	0.023053	-0.17715	0.215	0.158	1
Ticam1	3.44E-06	-0.17716	0.233	0.129	0.06829
Sema4a	0.027443	-0.17721	0.109	0.071	1
Tor1aip1	0.211526	-0.17724	0.803	0.683	1
0610009D(	0.890751	-0.17724	0.888	0.769	1
Copa	0.125073	-0.1773	0.633	0.512	1

Ttll12	0.010132	-0.17737	0.191	0.131	1
Tmco1	0.867265	-0.17743	0.855	0.719	1
Cenpq	0.001073	-0.17766	0.158	0.096	1
Glyr1	0.000545	-0.17771	0.512	0.36	1
Trnau1ap	0.042966	-0.17779	0.724	0.595	1
Actr3	0.004788	-0.1778	0.985	0.97	1
Bcat2	0.000104	-0.17796	0.594	0.426	1
Ptpn1	0.633921	-0.17797	0.945	0.813	1
Nfib	7.13E-05	-0.178	0.161	0.086	1
Sh3glb2	8.78E-05	-0.17801	0.4	0.263	1
Yipf1	0.002518	-0.17808	0.597	0.453	1
Eif3a	0.579791	-0.17829	0.842	0.737	1
Snhg9	0.386067	-0.17833	0.242	0.208	1
Ttc1	0.002668	-0.17849	0.479	0.357	1
Stard7	0.006546	-0.17852	0.37	0.264	1
Itpa	0.00626	-0.17866	0.342	0.251	1
Hyal2	0.12601	-0.17868	0.245	0.199	1
Psma2	0.02935	-0.17898	0.918	0.865	1
Usmg5	0.13418	-0.1791	0.933	0.894	1
Drap1	0.767396	-0.17919	0.876	0.788	1
C030046I0	0.005757	-0.17943	0.682	0.505	1
Sema6d	0.229211	-0.17946	0.685	0.568	1
Lyar	0.009622	-0.17947	0.521	0.396	1
Rbbp4	0.162778	-0.17947	0.658	0.542	1
Nab1	0.00535	-0.17948	0.476	0.352	1
Pgrmc1	0.560001	-0.17954	0.561	0.47	1
Polr2b	0.32121	-0.17974	0.161	0.135	1
Dok3	0.002114	-0.17977	0.506	0.361	1
Lta4h	0.322947	-0.17986	0.236	0.2	1
Psmb4	0.231873	-0.1801	0.876	0.793	1
Kank2	0.001558	-0.18011	0.112	0.062	1
Uap1	0.83631	-0.18021	0.364	0.317	1
Ttc3	0.181159	-0.18023	0.227	0.185	1
Psmb10	0.671557	-0.18049	0.933	0.863	1
Hes1	0.049684	-0.1806	0.427	0.334	1
Ppp6c	0.001635	-0.18068	0.367	0.257	1
Tmed7	0.069369	-0.18075	0.821	0.668	1
Gramd1a	0.001519	-0.18079	0.448	0.327	1
Hnrnph2	0.12489	-0.18083	0.7	0.556	1
Prcp	0.257464	-0.18084	0.782	0.652	1
Snf8	0.013078	-0.18089	0.842	0.683	1
Neurl3	1.32E-05	-0.18095	0.806	0.604	0.261894
Sec23a	0.025188	-0.18096	0.139	0.095	1

Pafah1b1	0.748125	-0.18109	0.755	0.646	1
Lrp1	0.585784	-0.18115	0.903	0.793	1
Phactr2	0.001089	-0.18119	0.382	0.265	1
Srrt	3.89E-05	-0.18126	0.412	0.266	0.772568
Map2k7	1.00E-05	-0.18127	0.382	0.246	0.199465
Cox6b1	0.009251	-0.18128	0.985	0.983	1
Stxbp2	0.157277	-0.18138	0.718	0.611	1
Pgd	0.334135	-0.18168	0.8	0.668	1
Cdk2ap1	1.42E-05	-0.18172	0.439	0.286	0.281119
Srp9	0.624285	-0.18214	0.942	0.86	1
Srp72	0.012158	-0.18215	0.639	0.493	1
Ip6k1	0.005409	-0.18222	0.3	0.212	1
Flot2	3.93E-05	-0.18223	0.336	0.211	0.781116
Lrp10	0.012167	-0.18233	0.742	0.575	1
F11r	0.000606	-0.18233	0.397	0.269	1
Snx6	0.308962	-0.18241	0.745	0.621	1
Nras	0.004309	-0.18251	0.621	0.476	1
Mrpl54	0.757019	-0.18256	0.836	0.73	1
Ccdc56	0.51131	-0.18269	0.83	0.735	1
Sema6a	4.72E-05	-0.1827	0.115	0.054	0.938166
Dusp7	0.024932	-0.18288	0.321	0.239	1
Uba2	0.002972	-0.18297	0.539	0.394	1
Ubl5	0.112393	-0.18316	0.982	0.956	1
Gnl3	0.01289	-0.18317	0.558	0.428	1
Ncaph2	0.00243	-0.18329	0.691	0.528	1
Ankhd1	9.64E-05	-0.18332	0.473	0.317	1
Foxp4	0.002534	-0.18372	0.118	0.069	1
Eif2b2	0.000296	-0.18375	0.452	0.31	1
1200011I1	0.011171	-0.18377	0.279	0.206	1
Poldip2	0.002114	-0.18402	0.503	0.377	1
Cxxc1	0.027014	-0.18405	0.333	0.257	1
Pam16	0.00543	-0.1841	0.624	0.474	1
Gm6251	8.48E-05	-0.18414	0.382	0.255	1
Acbd6	0.00503	-0.18418	0.585	0.439	1
Gm2382	0.000442	-0.18442	0.603	0.438	1
Magoh	0.070193	-0.18447	0.63	0.497	1
Ndufv1	0.219241	-0.18456	0.555	0.446	1
Fxr2	0.008336	-0.18458	0.436	0.322	1
Sf3b5	0.54082	-0.1846	0.924	0.833	1
Unc93b1	0.012521	-0.18469	0.994	0.96	1
Llph	0.618556	-0.18472	0.812	0.737	1
Pttg1	0.024243	-0.18477	0.794	0.641	1
C1qbp	0.134519	-0.18492	0.77	0.65	1

Dgkd	0.010148	-0.1851	0.173	0.118	1
Naa20	0.019453	-0.18524	0.667	0.52	1
Acy3	0.051249	-0.18525	0.121	0.085	1
Zmat2	0.063362	-0.18525	0.77	0.643	1
Runx1	0.013956	-0.18535	0.4	0.299	1
Yaf2	0.000528	-0.18542	0.536	0.385	1
Rchy1	0.011314	-0.18542	0.433	0.324	1
Commd4	0.017525	-0.18544	0.721	0.581	1
Dhrs7	0.17272	-0.18559	0.436	0.354	1
Ddt	0.00806	-0.18565	0.685	0.535	1
Gps2	6.91E-05	-0.18565	0.542	0.37	1
Zcchc7	0.003146	-0.18573	0.306	0.218	1
Luzp1	0.004186	-0.18579	0.588	0.437	1
Rest	0.012021	-0.1858	0.276	0.199	1
Ccdc32	0.001274	-0.1858	0.261	0.174	1
Acaa2	0.126922	-0.18582	0.585	0.476	1
Bloc1s2	0.000286	-0.18603	0.473	0.326	1
Hnrnpa3	0.496388	-0.18617	0.918	0.853	1
Rock2	0.001444	-0.18621	0.533	0.39	1
Uba1	0.161564	-0.18622	0.77	0.624	1
Tsr2	0.000719	-0.18626	0.245	0.158	1
Ythdc1	0.00875	-0.18633	0.47	0.363	1
Sdhaf1	0.004791	-0.18639	0.424	0.312	1
Lin7c	0.007865	-0.18647	0.288	0.209	1
Ubxn2a	3.42E-05	-0.18651	0.321	0.201	0.679413
Atp5g1	0.106076	-0.18653	0.955	0.924	1
Vps16	0.0189	-0.18666	0.206	0.148	1
Atp5f1	0.048673	-0.18688	0.948	0.884	1
Lpcat3	0.009057	-0.18693	0.591	0.455	1
Nr2c2	0.000907	-0.18704	0.209	0.131	1
Nop58	0.041612	-0.18709	0.421	0.317	1
Dnase2a	0.194984	-0.18714	0.861	0.723	1
Prpf3	0.021303	-0.18722	0.152	0.105	1
Slc25a25	0.144467	-0.18723	0.188	0.146	1
Cmtm4	5.33E-06	-0.18732	0.342	0.212	0.105795
Sfpq	0.787521	-0.18735	0.733	0.643	1
Nfatc2	2.06E-05	-0.1874	0.176	0.095	0.40923
Tceb1	0.891451	-0.18756	0.903	0.807	1
Med10	0.278771	-0.1878	0.679	0.569	1
Golga7	0.093588	-0.18784	0.779	0.619	1
Ctnnbl1	0.005358	-0.18798	0.333	0.245	1
Stx7	0.669122	-0.18799	0.827	0.718	1
Immt	0.074006	-0.18803	0.712	0.55	1

Yipf3	0.065407	-0.18811	0.627	0.5	1
Dnajc1	0.000321	-0.18814	0.497	0.355	1
Card11	9.22E-06	-0.18823	0.206	0.113	0.183088
Chmp1a	0.248953	-0.18826	0.709	0.591	1
Ctbp2	5.57E-05	-0.18833	0.452	0.306	1
Aimp1	0.011537	-0.1885	0.679	0.526	1
Nt5c	0.26584	-0.1885	0.661	0.533	1
Ube2v1	0.842539	-0.18862	0.836	0.733	1
Elovl1	0.279703	-0.18866	0.8	0.664	1
Rdx	0.236693	-0.18887	0.673	0.552	1
Eif4a1	0.004232	-0.18895	0.982	0.947	1
Suclg1	0.543897	-0.18902	0.724	0.609	1
Bag5	0.00604	-0.18915	0.364	0.263	1
Abcf1	0.010635	-0.18931	0.673	0.517	1
Crcp	0.016381	-0.18943	0.355	0.264	1
Irf7	0.336956	-0.1895	0.973	0.956	1
Spast	0.3792	-0.18952	0.23	0.194	1
H2-T23	0.168182	-0.18966	0.921	0.834	1
Txlng	0.000291	-0.18978	0.203	0.124	1
2810408M	0.076356	-0.1898	0.155	0.115	1
Ppp1r18	0.998126	-0.18985	0.821	0.707	1
Nfatc1	0.000644	-0.19023	0.367	0.247	1
Crtc3	0.063237	-0.19026	0.233	0.181	1
Stk17b	0.490037	-0.19031	0.885	0.775	1
G3bp1	0.482734	-0.19033	0.812	0.667	1
LOC10003	0.001087	-0.19036	0.618	0.456	1
Klhl6	0.003963	-0.19037	0.318	0.225	1
Epn1	0.476198	-0.19039	0.921	0.808	1
Sae1	0.001732	-0.19043	0.436	0.315	1
Cdk16	3.31E-05	-0.19055	0.548	0.372	0.658193
Mki67	0.000444	-0.19058	0.267	0.178	1
Pgp	0.000147	-0.19089	0.552	0.382	1
Eif2s1	0.084653	-0.19091	0.727	0.583	1
Trappc3	0.022988	-0.19095	0.682	0.543	1
Cdk5rap3	0.008482	-0.19104	0.427	0.32	1
Tmc6	0.001962	-0.19105	0.503	0.362	1
Stk16	0.035632	-0.19106	0.379	0.297	1
Ywhag	0.028155	-0.19122	0.758	0.614	1
Camk2d	0.001363	-0.19128	0.588	0.419	1
Cd86	0.021037	-0.19129	0.864	0.689	1
Psmc5	0.605095	-0.19143	0.715	0.603	1
Pmvk	0.000251	-0.19148	0.373	0.249	1
Btf3	0.016844	-0.19151	0.976	0.966	1

4933421E1	0.028358	-0.19153	0.224	0.166	1
Usp48	0.005378	-0.19172	0.403	0.298	1
Fbxo18	0.012277	-0.19183	0.324	0.237	1
Ppp1r12c	2.95E-06	-0.1919	0.67	0.47	0.058665
Wbp1	0.00154	-0.19193	0.303	0.209	1
Ict1	0.004812	-0.19204	0.564	0.412	1
Ifi47	0.093135	-0.19205	0.667	0.53	1
Ndfip2	0.032073	-0.19221	0.515	0.403	1
Naa50	0.404629	-0.19259	0.788	0.692	1
Dcakd	0.004746	-0.19264	0.267	0.184	1
Dus1l	0.011261	-0.19267	0.327	0.239	1
Ccdc124	0.262949	-0.19269	0.836	0.7	1
Eif3m	0.433555	-0.19292	0.812	0.7	1
H47	0.886023	-0.19297	0.879	0.759	1
Txndc15	0.035978	-0.19299	0.667	0.514	1
Scaf1	0.003051	-0.19307	0.361	0.255	1
B230312A1	0.066353	-0.19319	0.239	0.184	1
Ska1	0.001337	-0.19325	0.115	0.065	1
Tmem55b	0.017108	-0.19331	0.776	0.614	1
Psmb6	0.13361	-0.19344	0.909	0.85	1
Tctex1d2	0.005996	-0.19351	0.27	0.193	1
Leprel2	0.442454	-0.19362	0.136	0.117	1
Phldb1	0.000222	-0.19408	0.1	0.048	1
Arl13b	0.007325	-0.19409	0.155	0.099	1
Btbd9	9.74E-05	-0.19417	0.382	0.253	1
Strn3	0.006521	-0.19421	0.533	0.394	1
Selplg	0.440701	-0.19445	0.697	0.566	1
1700056E2	0.000102	-0.1947	0.23	0.14	1
Prosc	3.73E-05	-0.19487	0.467	0.31	0.739971
March2	0.041325	-0.19487	0.818	0.652	1
Gm1821	0.600041	-0.19489	0.827	0.724	1
Fbxo28	0.014153	-0.19521	0.188	0.131	1
Fnbp4	0.028825	-0.19539	0.242	0.18	1
Slc31a1	0.042521	-0.19557	0.533	0.412	1
Timm17a	0.01263	-0.19563	0.67	0.517	1
Psma1	0.373761	-0.19568	0.782	0.735	1
Csgalnact2	0.014169	-0.1958	0.179	0.121	1
8430410A1	0.000137	-0.19596	0.427	0.291	1
Hcst	0.97729	-0.19596	0.733	0.624	1
3110001D0	0.893213	-0.19598	0.882	0.788	1
Mrpl17	0.484265	-0.19601	0.839	0.71	1
Klhdc2	0.001297	-0.1962	0.376	0.26	1
Fkbp3	0.016703	-0.19627	0.652	0.508	1

Rbm42	0.06329	-0.19635	0.658	0.524	1
6430527G	0.43441	-0.19635	0.182	0.157	1
Usp25	0.13077	-0.19645	0.724	0.592	1
Sdc2	0.0559	-0.19645	0.206	0.153	1
Snrpc	0.124185	-0.19646	0.739	0.604	1
Phip	0.014461	-0.19649	0.333	0.244	1
Cdc26	0.00943	-0.19652	0.636	0.505	1
Nr3c1	0.530714	-0.19664	0.697	0.594	1
Uggt1	0.053362	-0.19677	0.3	0.233	1
Nrm	0.00472	-0.19684	0.315	0.227	1
Rbm6	0.002706	-0.19715	0.327	0.232	1
Gna11	0.065486	-0.19721	0.248	0.19	1
Rnf126	8.86E-05	-0.19731	0.47	0.32	1
Fam108a	0.096317	-0.19744	0.767	0.627	1
Tecpr1	1.01E-05	-0.19746	0.167	0.085	0.200084
Prr14	0.007422	-0.19757	0.221	0.154	1
Rfk	0.002793	-0.19783	0.433	0.311	1
Mrpl19	9.52E-05	-0.19787	0.403	0.263	1
Tm6sf1	0.034869	-0.19807	0.785	0.622	1
Hnrnpm	0.176893	-0.19809	0.855	0.694	1
Ube2r2	0.033674	-0.19814	0.733	0.581	1
Nr1d2	0.125793	-0.19822	0.158	0.121	1
Lgals9	0.393629	-0.19823	0.876	0.785	1
Sept11	0.000291	-0.19825	0.227	0.14	1
Sp2	5.84E-05	-0.19826	0.182	0.1	1
Pts	0.039234	-0.19828	0.603	0.48	1
Dnajc8	0.238371	-0.19853	0.827	0.683	1
Fam173a	0.255778	-0.19861	0.706	0.596	1
Aspscr1	0.044487	-0.19871	0.382	0.301	1
Ptprb	0.032778	-0.19877	0.164	0.117	1
Gpx4	0.160943	-0.19879	0.973	0.943	1
Dph3	0.351526	-0.19893	0.676	0.561	1
Npepps	0.006267	-0.19896	0.345	0.249	1
Fam162a	0.126579	-0.19925	0.736	0.622	1
Tpm3	0.00603	-0.19927	0.979	0.961	1
Spata6	0.04013	-0.19928	0.261	0.195	1
Gstt3	0.001028	-0.19941	0.112	0.061	1
Appbp2	0.004728	-0.19957	0.212	0.143	1
Man2a1	0.010115	-0.19962	0.548	0.411	1
Pdha1	0.00572	-0.1997	0.561	0.406	1
Notch1	3.92E-05	-0.19972	0.339	0.215	0.777921
Wdr83	0.004618	-0.19995	0.233	0.158	1
Polb	0.0514	-0.20003	0.542	0.442	1

Thrap3	0.014997	-0.20034	0.564	0.417	1
Prmt1	0.341581	-0.20035	0.573	0.487	1
Ptpn11	2.84E-05	-0.20041	0.482	0.325	0.564422
Rab5a	0.029643	-0.2005	0.655	0.523	1
Galk2	0.001431	-0.20052	0.442	0.319	1
Git1	0.00087	-0.20057	0.276	0.185	1
Bcl2l11	0.020389	-0.20064	0.248	0.178	1
Cbr1	0.081078	-0.20065	0.512	0.415	1
Cct2	0.400338	-0.20069	0.782	0.66	1
Baz1b	0.016858	-0.20083	0.573	0.443	1
Rnf139	7.42E-05	-0.20093	0.409	0.282	1
Hpcal1	0.493152	-0.20108	0.939	0.86	1
Hnrnpd	0.243069	-0.20119	0.685	0.562	1
Cmas	0.142077	-0.20122	0.697	0.565	1
Tnfsf12	0.001384	-0.20127	0.37	0.259	1
Cln5	0.041635	-0.20128	0.382	0.295	1
Syvn1	0.002187	-0.20184	0.276	0.185	1
Abi1	0.483617	-0.20191	0.736	0.609	1
Mpg	0.018656	-0.20194	0.361	0.276	1
Xpc	1.87E-05	-0.20195	0.215	0.121	0.370881
Fam126b	0.009688	-0.20201	0.118	0.074	1
Rad23b	0.032222	-0.20215	0.676	0.561	1
2310039H(	6.79E-06	-0.2022	0.597	0.412	0.134846
Irak2	0.000506	-0.20232	0.5	0.357	1
Ift20	0.017761	-0.20239	0.633	0.495	1
Ndufv3	0.20387	-0.20251	0.921	0.858	1
Glt8d1	0.005146	-0.20273	0.267	0.19	1
Tceb2	0.197091	-0.20279	0.973	0.969	1
Cops8	0.161105	-0.20281	0.53	0.44	1
Folr1	0.005885	-0.20294	0.252	0.175	1
Thop1	0.002422	-0.20301	0.185	0.12	1
Slc24a6	0.011202	-0.20313	0.709	0.581	1
Ap3m2	0.002603	-0.20318	0.152	0.093	1
1110005A(	0.031887	-0.20328	0.467	0.347	1
Snx21	0.000449	-0.20341	0.194	0.118	1
Zcchc17	0.00772	-0.20344	0.561	0.422	1
Esco1	0.07273	-0.20349	0.245	0.189	1
Lxn	0.000534	-0.20361	0.339	0.231	1
Anxa3	0.627916	-0.20384	0.827	0.748	1
Mrps16	0.750343	-0.20393	0.742	0.64	1
Irs2	0.003557	-0.20394	0.133	0.081	1
Echs1	0.045182	-0.20399	0.63	0.478	1
Scp2	0.021141	-0.204	0.973	0.937	1

Tmed9	0.34799	-0.20401	0.933	0.827	1
Sc4mol	0.000976	-0.20411	0.385	0.272	1
Uqcrb	0.033064	-0.20416	0.979	0.95	1
Slc50a1	0.311181	-0.20427	0.606	0.511	1
Csrp2	0.064172	-0.2044	0.158	0.115	1
Irgm1	0.006583	-0.20441	0.627	0.488	1
Ece1	0.479895	-0.20444	0.197	0.171	1
Psmg2	0.000172	-0.20447	0.485	0.324	1
Mrps21	0.381224	-0.20464	0.933	0.84	1
Get4	0.000343	-0.20466	0.464	0.333	1
Tmem2	0.000336	-0.2047	0.264	0.17	1
Fam89b	0.379948	-0.20473	0.788	0.679	1
Glb1	0.000391	-0.20475	0.606	0.448	1
Ikzf1	0.026575	-0.20493	0.558	0.432	1
Camkk2	0.080013	-0.205	0.267	0.205	1
G6pc3	7.54E-05	-0.20503	0.394	0.263	1
Klhdc3	0.081075	-0.20506	0.342	0.27	1
Ctnnb1	0.127405	-0.20523	0.63	0.515	1
Myh9	0.028386	-0.20543	0.682	0.544	1
1110008P1	0.009512	-0.20546	0.485	0.361	1
Sbno1	0.001451	-0.20547	0.53	0.377	1
Ogt	0.002583	-0.20554	0.491	0.367	1
Zfp295	0.021996	-0.20558	0.158	0.109	1
Commd7	0.049144	-0.20564	0.685	0.55	1
Mtch2	0.382747	-0.20578	0.733	0.628	1
Arid3a	0.024967	-0.20585	0.121	0.081	1
Snw1	0.018477	-0.20605	0.652	0.502	1
Ube2f	0.04823	-0.20614	0.767	0.62	1
Hspa14	0.002023	-0.20624	0.448	0.332	1
Ppp2r5e	0.01067	-0.20627	0.282	0.201	1
Cdkn1b	0.007228	-0.20644	0.415	0.308	1
Eif3b	0.055043	-0.20657	0.688	0.542	1
Zmiz2	0.000126	-0.20678	0.406	0.27	1
Mdfic	0.018025	-0.20679	0.506	0.405	1
Ivns1abp	0.197511	-0.20681	0.588	0.495	1
Fam3c	0.043996	-0.20695	0.718	0.571	1
Chchd10	0.016514	-0.20727	0.109	0.155	1
Myo1f	0.006965	-0.20728	0.676	0.528	1
Ndufa10	0.024284	-0.20737	0.603	0.457	1
Nup35	0.001632	-0.20765	0.164	0.102	1
Kdelc2	0.00021	-0.20778	0.236	0.144	1
Pofut2	0.020748	-0.20791	0.6	0.48	1
Slc24a5	0.018001	-0.20812	0.1	0.063	1

Inpp5a	0.003393	-0.20816	0.127	0.076	1
Zfp148	0.003155	-0.20821	0.439	0.318	1
2310003C2	0.097857	-0.20823	0.288	0.228	1
Akr7a5	0.074226	-0.20825	0.418	0.331	1
Dvl1	0.000101	-0.2083	0.158	0.085	1
AI848100	0.000117	-0.20839	0.203	0.118	1
Atp5h	0.030734	-0.20848	0.988	0.965	1
Pskh1	0.001741	-0.20878	0.182	0.115	1
Rere	0.022701	-0.209	0.427	0.33	1
Cbx4	0.000244	-0.2094	0.306	0.198	1
Gtf2f2	0.001761	-0.20947	0.403	0.288	1
Fam108c	0.000188	-0.20954	0.539	0.381	1
Mad2l1bp	0.000134	-0.20956	0.203	0.12	1
Clptm1	0.021997	-0.20963	0.755	0.578	1
Mrpl18	0.216201	-0.20985	0.745	0.613	1
Arhgef12	0.001178	-0.20987	0.4	0.285	1
Sike1	0.000305	-0.20992	0.358	0.241	1
Wsb2	0.008158	-0.20992	0.464	0.347	1
Ncaph	5.05E-05	-0.20993	0.13	0.066	1
Ssr2	0.656373	-0.21027	0.815	0.693	1
Fzr1	0.000322	-0.21036	0.367	0.242	1
Eif4g3	0.001102	-0.21042	0.603	0.444	1
Plekho1	0.024473	-0.21044	0.833	0.682	1
Sepw1	0.020063	-0.21074	0.976	0.956	1
Zfp560	0.000768	-0.21076	0.279	0.183	1
Lzts2	0.000488	-0.21078	0.152	0.086	1
Mta3	0.00502	-0.21085	0.606	0.476	1
Cdc34	0.035122	-0.21113	0.624	0.494	1
E130012A1	1.03E-05	-0.21152	0.206	0.116	0.204139
Sla	0.019452	-0.21165	0.697	0.539	1
Srsf1	0.103876	-0.21172	0.524	0.413	1
Ccl6	0.001021	-0.21176	0.982	0.975	1
Fam111a	0.011276	-0.21198	0.73	0.592	1
Arhgap1	3.64E-05	-0.212	0.391	0.253	0.723548
Cib1	0.420127	-0.21201	0.785	0.644	1
Tmem165	0.006738	-0.21209	0.518	0.393	1
Slc25a46	0.000259	-0.21213	0.43	0.297	1
Dpysl2	0.127794	-0.21221	0.485	0.391	1
Vps37c	0.083381	-0.21224	0.142	0.107	1
Atxn7	2.97E-06	-0.21237	0.382	0.236	0.058978
Sin3b	0.233162	-0.21246	0.77	0.633	1
Mgst1	0.244697	-0.21266	0.579	0.545	1
Cd48	0.990763	-0.21276	0.891	0.782	1

Icosl	0.183973	-0.21277	0.185	0.15	1
Acox1	0.105664	-0.21312	0.27	0.212	1
Mvp	0.060236	-0.21314	0.591	0.477	1
Bola3	0.050753	-0.21317	0.688	0.545	1
Sypl	0.831769	-0.21347	0.609	0.531	1
Slc35e1	0.000296	-0.21353	0.33	0.223	1
l7Rn6	0.008415	-0.21354	0.633	0.493	1
Lig1	0.004778	-0.21376	0.418	0.312	1
Timm8b	0.402614	-0.21384	0.833	0.75	1
Sec14l1	0.356564	-0.21395	0.648	0.552	1
Rbpms	0.039276	-0.21408	0.136	0.095	1
Styx	0.010487	-0.21411	0.221	0.154	1
2810428I1	0.451658	-0.21412	0.855	0.732	1
Wtap	0.320373	-0.21413	0.612	0.506	1
Prr3	0.000334	-0.21419	0.185	0.109	1
Ppm1m	0.013467	-0.21443	0.327	0.24	1
Snx15	0.000155	-0.2145	0.467	0.318	1
Pafah1b2	0.002515	-0.21451	0.57	0.418	1
Tor1a	0.001324	-0.21455	0.788	0.602	1
Gnai3	0.144477	-0.21456	0.433	0.344	1
Dpm1	0.013882	-0.21469	0.648	0.494	1
Rap1a	0.071473	-0.21481	0.933	0.9	1
Tomm5	0.799445	-0.21487	0.791	0.713	1
Rbms2	0.000174	-0.21487	0.494	0.342	1
Naaa	0.031682	-0.21523	0.47	0.371	1
Smarcd2	0.037096	-0.21537	0.53	0.406	1
D730005E1	0.002879	-0.21553	0.118	0.07	1
Tcea1	0.124569	-0.21556	0.706	0.571	1
Eml4	0.153258	-0.21574	0.264	0.212	1
Fam133b	0.005131	-0.21582	0.352	0.256	1
Riok3	0.107005	-0.21588	0.694	0.554	1
Qars	0.059456	-0.21588	0.415	0.326	1
Dnajc19	0.085408	-0.21601	0.785	0.663	1
Psmc4	0.545914	-0.21614	0.706	0.597	1
Adk	0.051483	-0.21625	0.282	0.216	1
Cir1	0.007691	-0.21658	0.4	0.297	1
Sorbs3	0.012249	-0.21659	0.106	0.066	1
Clec4f	3.27E-06	-0.21663	0.994	0.988	0.064852
1600012F0	0.071017	-0.21668	0.197	0.15	1
Fam100b	0.00178	-0.21686	0.47	0.34	1
9130011J1	0.056728	-0.21689	0.727	0.576	1
Pex13	0.000143	-0.21734	0.564	0.403	1
Gmds	0.000356	-0.21742	0.294	0.196	1

Il10rb	0.481662	-0.21751	0.912	0.821	1
Jagn1	0.000387	-0.21755	0.588	0.419	1
Fam69a	0.040762	-0.21756	0.379	0.294	1
Canx	0.038645	-0.21756	0.948	0.895	1
Fubp1	0.008927	-0.21764	0.579	0.438	1
Timm23	0.80503	-0.21778	0.903	0.802	1
Prdm2	0.112617	-0.21779	0.279	0.224	1
Cbx5	0.000318	-0.2178	0.37	0.246	1
2310044H1	0.764572	-0.2178	0.836	0.692	1
Rasgrp4	0.002836	-0.21782	0.191	0.124	1
Ube3a	0.006992	-0.21783	0.536	0.394	1
Trappc4	0.070987	-0.21789	0.77	0.631	1
Dlgap4	0.025089	-0.21794	0.561	0.426	1
Hist3h2a	0.006113	-0.21796	0.691	0.564	1
Acsl3	0.013384	-0.21797	0.145	0.097	1
Limd2	0.671217	-0.21801	0.691	0.59	1
Vps72	1.30E-05	-0.21807	0.497	0.318	0.258158
Pcgf5	0.004718	-0.21815	0.227	0.156	1
Xpnpep1	0.012486	-0.21822	0.333	0.244	1
Gsk3b	0.50658	-0.21834	0.661	0.545	1
Tkt	0.079344	-0.21845	0.915	0.865	1
Dnajb4	0.41623	-0.21856	0.106	0.088	1
Lamtor2	0.312749	-0.21892	0.948	0.894	1
Adh5	0.071906	-0.21895	0.527	0.432	1
Coq2	0.259727	-0.21911	0.497	0.406	1
Sys1	0.273181	-0.21919	0.779	0.661	1
Epm2aip1	0.154093	-0.2192	0.1	0.075	1
Mob4	0.329865	-0.21933	0.515	0.43	1
Nr1h2	0.012988	-0.21938	0.618	0.475	1
Lrrc8c	9.68E-05	-0.21949	0.27	0.165	1
Papola	0.085309	-0.21957	0.842	0.681	1
Zfp326	0.000644	-0.21975	0.376	0.263	1
Sept9	0.037374	-0.21976	0.633	0.507	1
Ube2e1	0.218151	-0.21985	0.752	0.638	1
2010106G0	0.052458	-0.21996	0.715	0.573	1
Cuta	0.595191	-0.21999	0.915	0.825	1
Prkca	0.249701	-0.22005	0.167	0.134	1
Rps27l	0.021153	-0.22012	0.964	0.96	1
Celf2	0.948182	-0.22036	0.818	0.718	1
Pom121	0.002629	-0.22072	0.267	0.178	1
Tbc1d12	3.92E-05	-0.22078	0.106	0.048	0.778628
Sox4	0.008784	-0.22081	0.106	0.065	1
Mak16	0.0161	-0.22091	0.412	0.305	1

Pcif1	0.025338	-0.22135	0.394	0.296	1
Sdf2	0.06647	-0.22147	0.761	0.61	1
Ppt1	0.556774	-0.22151	0.745	0.643	1
Vasp	0.008196	-0.22174	0.948	0.893	1
Auh	0.121502	-0.22193	0.188	0.143	1
Mea1	0.079044	-0.222	0.7	0.569	1
Tigd2	0.001439	-0.22204	0.567	0.429	1
Plekhj1	0.956059	-0.2221	0.848	0.737	1
Psma6	0.112567	-0.2222	0.933	0.907	1
Caprin1	0.045638	-0.22225	0.809	0.649	1
Oxr1	0.009866	-0.22258	0.385	0.29	1
Cd151	0.058965	-0.22268	0.461	0.358	1
Mapre3	4.88E-06	-0.22269	0.291	0.173	0.096827
Ergic3	0.744086	-0.22271	0.836	0.711	1
Lmbrd1	0.010479	-0.22286	0.685	0.526	1
Nop10	0.413541	-0.22307	0.982	0.934	1
Nop56	0.141664	-0.22315	0.53	0.425	1
Diablo	0.11543	-0.22318	0.415	0.325	1
Vbp1	0.022426	-0.22327	0.515	0.402	1
Uqcr11	0.022072	-0.22344	0.979	0.965	1
Sec63	4.41E-06	-0.22379	0.439	0.288	0.0876
Isyna1	0.029546	-0.22392	0.652	0.511	1
Pkd1	0.047585	-0.22406	0.145	0.105	1
Psmd14	0.020095	-0.22414	0.67	0.515	1
Ubqln1	0.307817	-0.22417	0.552	0.46	1
Mgrn1	0.000113	-0.22423	0.588	0.433	1
Flnb	0.063238	-0.2244	0.118	0.083	1
Iws1	0.076258	-0.22441	0.303	0.236	1
Mfap1a	0.020794	-0.22442	0.327	0.248	1
Ndufv2	0.526659	-0.22461	0.852	0.775	1
Igsf6	0.341488	-0.22477	0.724	0.601	1
Rin2	0.016556	-0.22486	0.539	0.412	1
Mpdu1	0.011229	-0.22487	0.576	0.451	1
Dhfr	0.011108	-0.22496	0.176	0.122	1
Cobra1	0.06861	-0.22513	0.324	0.249	1
Myeov2	0.222429	-0.22538	0.97	0.934	1
Polr3gl	0.000681	-0.22538	0.476	0.335	1
Fdx1	0.009921	-0.2255	0.285	0.2	1
March6	0.000592	-0.22561	0.485	0.349	1
Tnfsf10	7.17E-05	-0.22563	0.155	0.084	1
Leprot	0.654682	-0.22573	0.897	0.792	1
Slc35a4	0.012969	-0.22583	0.521	0.394	1
Ktn1	0.011347	-0.22593	0.203	0.139	1

1600014C1	0.000981	-0.22595	0.403	0.289	1
Mzt2	0.029147	-0.22625	0.2	0.145	1
2900010M	0.274233	-0.2263	0.888	0.817	1
Gt(ROSA)2	0.036035	-0.22639	0.606	0.473	1
Wdr4	0.002847	-0.2265	0.155	0.094	1
Entpd1	0.917631	-0.22661	0.755	0.655	1
1810022K0	0.176393	-0.22679	0.6	0.486	1
Med31	0.019528	-0.22696	0.252	0.18	1
Ggta1	3.69E-05	-0.227	0.391	0.256	0.732583
Rab2a	0.112791	-0.22702	0.885	0.816	1
Ubr4	1.74E-05	-0.22709	0.576	0.394	0.346259
Tmem106l	0.001352	-0.22713	0.397	0.282	1
Abcb7	0.000816	-0.22713	0.212	0.134	1
Ywhaq	0.928105	-0.22729	0.83	0.711	1
Stk19	0.001794	-0.22754	0.533	0.405	1
Samhd1	0.750094	-0.22765	0.912	0.801	1
Ndufa12	0.324339	-0.22766	0.867	0.785	1
Slain2	0.003268	-0.22773	0.294	0.202	1
Gatad1	0.01688	-0.22793	0.539	0.428	1
Rwdd1	0.723617	-0.22802	0.83	0.723	1
Dctn3	0.495431	-0.22802	0.861	0.74	1
Rftn1	0.003399	-0.22805	0.4	0.283	1
Cpne3	0.1278	-0.22811	0.5	0.408	1
Psmb5	0.171092	-0.22816	0.855	0.766	1
Cd2ap	0.935245	-0.22839	0.639	0.555	1
Scpep1	0.137206	-0.22888	0.591	0.475	1
Tmem60	0.036626	-0.22901	0.461	0.374	1
1200016B1	7.11E-05	-0.22902	0.276	0.171	1
Yars	0.002607	-0.22927	0.227	0.153	1
Fchsd2	0.33318	-0.2297	0.112	0.093	1
Pih1d1	3.52E-05	-0.22972	0.564	0.386	0.698774
Terf2ip	0.007887	-0.22976	0.152	0.1	1
2310045N0	0.001332	-0.22981	0.406	0.283	1
Smad3	0.026352	-0.22982	0.121	0.082	1
Ufc1	0.881198	-0.22982	0.794	0.683	1
E2f4	0.296395	-0.22994	0.552	0.455	1
Usp47	0.000293	-0.22996	0.324	0.212	1
Acvrl1	0.67707	-0.23016	0.791	0.656	1
Gmnn	0.003435	-0.23042	0.545	0.413	1
Ctdsp1	0.06227	-0.23043	0.694	0.539	1
Gle1	0.001665	-0.23081	0.242	0.16	1
Adss	0.037311	-0.23089	0.348	0.265	1
Mmgt2	0.025655	-0.23111	0.148	0.104	1

Ap2a2	0.385297	-0.23113	0.736	0.614	1
Cep57	0.001278	-0.2313	0.258	0.174	1
Rab1b	0.051806	-0.23134	0.627	0.517	1
Bud31	0.916139	-0.23149	0.752	0.645	1
Slc9a3r2	0.855991	-0.23151	0.139	0.129	1
BC005561	0.003502	-0.23156	0.267	0.181	1
Gm15421	0.096273	-0.23161	0.67	0.533	1
Rictor	0.000361	-0.23177	0.191	0.113	1
Rogdi	0.026302	-0.23187	0.345	0.267	1
Pnpo	0.445845	-0.23203	0.212	0.184	1
Tsnax	0.004569	-0.23204	0.509	0.376	1
Ptpmt1	0.006368	-0.23204	0.748	0.578	1
Cdk2ap2	0.136551	-0.23232	0.915	0.85	1
Pomp	0.006656	-0.23236	0.979	0.953	1
Cables2	0.009381	-0.23246	0.103	0.062	1
Dpf2	0.000126	-0.23248	0.403	0.271	1
Churc1	0.464405	-0.2325	0.721	0.598	1
Socs2	0.187452	-0.2325	0.161	0.127	1
Josd2	0.021311	-0.23264	0.573	0.44	1
Kctd10	0.138316	-0.23274	0.242	0.191	1
Ppie	0.000637	-0.23275	0.336	0.226	1
Pdcd5	0.714898	-0.23286	0.882	0.789	1
Acot13	0.371988	-0.23289	0.642	0.536	1
Lpxn	0.019881	-0.23294	0.67	0.509	1
Casp8	0.197594	-0.23299	0.764	0.637	1
Ilf2	0.013224	-0.233	0.342	0.252	1
Snrpa1	0.048507	-0.23307	0.458	0.353	1
Ifi35	0.072549	-0.23309	0.745	0.606	1
Pnkd	0.004177	-0.23312	0.633	0.477	1
Chmp6	0.003319	-0.23316	0.497	0.36	1
Zbtb8os	0.000509	-0.23342	0.442	0.313	1
Wbp2	0.149726	-0.23343	0.636	0.513	1
Mrps23	0.078576	-0.23351	0.573	0.449	1
Znrd1	0.113376	-0.23372	0.621	0.503	1
Commd3	0.436982	-0.23392	0.821	0.7	1
Scn1b	0.001827	-0.23394	0.691	0.557	1
Commd1	0.234505	-0.23405	0.803	0.668	1
Jmjd1c	0.000393	-0.23416	0.461	0.318	1
Zfp318	0.004275	-0.2343	0.288	0.2	1
Uchl3	0.004746	-0.23431	0.606	0.46	1
Gsr	0.959258	-0.23433	0.536	0.469	1
Mrps18a	0.340351	-0.23445	0.676	0.578	1
Acsl5	0.160467	-0.23447	0.833	0.702	1

Cited2	0.516937	-0.23456	0.615	0.519	1
Samm50	0.132481	-0.23464	0.806	0.659	1
Srsf2	0.07018	-0.23467	0.858	0.799	1
Mrpl53	0.066487	-0.23485	0.573	0.461	1
Lsm14a	0.009167	-0.23486	0.542	0.41	1
1300018I1	2.55E-05	-0.23491	0.255	0.152	0.506802
Phax	0.049135	-0.23492	0.548	0.443	1
Gtf2b	0.012161	-0.23503	0.424	0.318	1
2410127L1	0.005078	-0.23511	0.324	0.23	1
Evl	0.447555	-0.23521	0.891	0.752	1
Adpgk	0.193189	-0.23521	0.579	0.467	1
Polr2m	0.527816	-0.23525	0.521	0.422	1
Zfp397	0.587077	-0.23536	0.115	0.102	1
Stx4a	0.424117	-0.23546	0.8	0.673	1
Pa2g4	0.572075	-0.23546	0.715	0.609	1
Gpx1	3.94E-05	-0.2355	1	0.997	0.781572
Cd300a	0.45692	-0.23551	0.952	0.873	1
Atf2	0.002916	-0.23552	0.261	0.176	1
Insig1	0.023812	-0.23558	0.464	0.369	1
Hsd17b11	0.000178	-0.23564	0.718	0.515	1
Wars	0.000196	-0.23584	0.436	0.298	1
Fam114a2	0.00324	-0.23587	0.458	0.328	1
Glrx3	0.870029	-0.23605	0.739	0.632	1
Crat	7.75E-05	-0.23635	0.339	0.222	1
Fam165b	0.359262	-0.23659	0.779	0.654	1
Wibg	0.038013	-0.23677	0.233	0.175	1
Synj2bp	0.051365	-0.2369	0.412	0.325	1
Upf3a	0.000161	-0.2376	0.485	0.339	1
Tgs1	0.000982	-0.23764	0.6	0.441	1
Eif4e	0.193768	-0.23772	0.715	0.581	1
Sac3d1	0.004353	-0.23773	0.264	0.181	1
Lsmd1	0.452508	-0.23775	0.688	0.58	1
Phc2	0.036001	-0.2379	0.536	0.415	1
Ppm1a	2.23E-05	-0.2379	0.464	0.306	0.442253
Commd5	0.000888	-0.23794	0.282	0.187	1
Acadl	0.008053	-0.23816	0.533	0.398	1
Ang	0.000864	-0.23819	0.452	0.321	1
Ric8	0.013186	-0.23828	0.339	0.246	1
Alkbh3	0.000148	-0.2383	0.367	0.246	1
Scarb1	0.010979	-0.23834	0.87	0.726	1
1600020E(	0.002374	-0.23842	0.224	0.149	1
Dnpep	0.009083	-0.23844	0.427	0.323	1
Cabin1	0.001066	-0.23854	0.321	0.217	1

Gart	9.18E-05	-0.23864	0.267	0.168	1
Bet1	0.012732	-0.23867	0.633	0.503	1
Ufd1l	0.001508	-0.23868	0.336	0.234	1
Dusp11	0.284195	-0.23874	0.582	0.482	1
Mcm7	0.009407	-0.23881	0.297	0.212	1
Hiat1	0.008016	-0.23887	0.476	0.354	1
6030429G(	0.001048	-0.2389	0.403	0.279	1
Tnks2	4.15E-05	-0.23899	0.53	0.358	0.825042
Arhgef1	0.079886	-0.23926	0.558	0.436	1
Mark3	0.002544	-0.23928	0.321	0.227	1
Chkb	8.03E-05	-0.23931	0.391	0.263	1
Chchd1	0.181157	-0.23944	0.785	0.654	1
Erf	0.006912	-0.23971	0.361	0.267	1
Mrpl43	0.84354	-0.23973	0.648	0.564	1
Palm	0.02074	-0.23975	0.2	0.139	1
Lass2	0.657596	-0.23986	0.685	0.574	1
Tnfaip1	3.76E-06	-0.23989	0.488	0.321	0.074577
Prkch	0.003609	-0.23992	0.285	0.2	1
Pdia4	0.373028	-0.24004	0.727	0.656	1
Pyhin1	0.042628	-0.24006	0.618	0.505	1
Dguok	0.004138	-0.24032	0.673	0.497	1
Trex1	0.071779	-0.24037	0.712	0.57	1
Rbck1	0.007887	-0.24068	0.618	0.477	1
Acot9	0.062146	-0.2408	0.603	0.482	1
Plekhf2	0.009861	-0.24114	0.479	0.359	1
Psma5	0.848698	-0.24116	0.885	0.771	1
Tep1	0.019522	-0.24123	0.761	0.6	1
Lcp2	0.159736	-0.24141	0.894	0.727	1
Pank2	0.002191	-0.24143	0.482	0.352	1
Hexb	0.72589	-0.24145	0.8	0.704	1
Zfp52	0.05481	-0.24153	0.1	0.069	1
Fut8	0.023779	-0.24176	0.215	0.157	1
Katna1	0.004421	-0.24218	0.442	0.32	1
Usp34	0.016531	-0.24224	0.367	0.267	1
Sf3a3	0.012352	-0.24249	0.361	0.268	1
Trove2	8.29E-05	-0.24254	0.155	0.084	1
Srpk1	0.001547	-0.2426	0.355	0.248	1
Atp5j2	0.021966	-0.24274	0.985	0.959	1
Dcps	0.125973	-0.24276	0.43	0.339	1
Cct3	0.797575	-0.24278	0.694	0.585	1
Coq7	0.014678	-0.24292	0.494	0.366	1
Mgea5	0.001981	-0.24307	0.412	0.292	1
Bre	5.43E-05	-0.24314	0.394	0.258	1

Sdhc	0.879002	-0.24347	0.803	0.7	1
Tomm34	0.016484	-0.24354	0.445	0.337	1
Taf12	0.020987	-0.2437	0.464	0.35	1
Tmem9b	0.516067	-0.24379	0.727	0.618	1
Uchl5	0.000116	-0.24392	0.555	0.39	1
Sord	0.036859	-0.24398	0.239	0.175	1
Msl1	0.003215	-0.24404	0.464	0.338	1
Calcrl	0.02147	-0.24405	0.136	0.092	1
Riok1	0.062927	-0.24408	0.203	0.154	1
Ythdc2	0.337497	-0.24415	0.13	0.107	1
Ssna1	0.105281	-0.2444	0.636	0.514	1
Acot7	0.000151	-0.24441	0.164	0.09	1
Txlna	0.000335	-0.24459	0.333	0.219	1
Tbl3	0.015048	-0.24475	0.179	0.124	1
Scaf8	0.005173	-0.24477	0.215	0.146	1
Ilvbl	0.012594	-0.24526	0.348	0.259	1
Slc38a10	0.015575	-0.2457	0.485	0.37	1
Hnrnpul2	0.100974	-0.24583	0.694	0.569	1
Fuca2	0.04272	-0.24585	0.688	0.556	1
Sap18	0.03531	-0.24599	0.248	0.184	1
Spcs2	0.115843	-0.24604	0.915	0.818	1
Fkbp4	0.13928	-0.24607	0.755	0.603	1
Dhx16	0.066607	-0.24638	0.197	0.149	1
Mrpl4	0.018158	-0.24646	0.652	0.51	1
Zfp281	0.000963	-0.24682	0.421	0.299	1
Taf11	0.275993	-0.24689	0.391	0.324	1
Irf3	0.009938	-0.24708	0.464	0.348	1
Sept2	0.216675	-0.24726	0.779	0.65	1
Mesdc1	0.018495	-0.2473	0.521	0.401	1
Nek7	0.011018	-0.24731	0.294	0.211	1
Insig2	0.000195	-0.24737	0.364	0.239	1
Vkorc1	0.048822	-0.24742	0.703	0.571	1
Rcn1	0.998304	-0.24766	0.115	0.111	1
Tmem43	0.012447	-0.24768	0.333	0.244	1
Hsd3b7	0.004399	-0.24793	0.418	0.308	1
Dmwd	0.003436	-0.24803	0.252	0.173	1
Apoc1	0.009348	-0.24804	0.997	0.997	1
Aars	0.000109	-0.24805	0.591	0.419	1
Ifi203	0.910502	-0.24825	0.876	0.754	1
Rps15a-ps4	0.098611	-0.24843	0.858	0.767	1
Aldh7a1	0.001201	-0.24845	0.306	0.208	1
Morf4l1	0.310006	-0.24853	0.964	0.906	1
Prpf4b	0.0272	-0.24882	0.488	0.374	1

Dhx36	0.000763	-0.24894	0.288	0.19	1
Rmnd5b	0.038606	-0.24896	0.2	0.148	1
Snhg6	0.124508	-0.24898	0.558	0.452	1
Srf	0.014214	-0.24908	0.236	0.168	1
Trnt1	5.17E-06	-0.24937	0.409	0.254	0.102667
Coq9	0.741441	-0.24941	0.245	0.222	1
Ptov1	0.047198	-0.24962	0.621	0.486	1
Pfkfb2	0.066321	-0.24968	0.106	0.076	1
Axl	0.293994	-0.24987	0.979	0.947	1
Lsm12	0.005575	-0.2499	0.6	0.472	1
Birc3	0.020254	-0.2499	0.485	0.374	1
Ccdc134	0.002948	-0.25004	0.179	0.114	1
Pdia3	0.002176	-0.25009	0.979	0.949	1
Rnf113a2	0.001834	-0.25041	0.261	0.177	1
Dad1	0.004215	-0.25048	0.942	0.906	1
5930434B(	0.000711	-0.25049	0.291	0.195	1
Tmed2	0.133919	-0.25057	0.942	0.889	1
Palld	4.95E-05	-0.25061	0.148	0.077	0.983967
Atp11b	0.015187	-0.25083	0.148	0.1	1
Mcm6	0.003831	-0.25121	0.403	0.293	1
Tmem205	0.299441	-0.25127	0.767	0.648	1
Vamp2	0.023714	-0.25133	0.33	0.247	1
Smndc1	0.002495	-0.25155	0.461	0.335	1
Rnf11	0.026273	-0.25169	0.206	0.147	1
Rbm8a	0.566639	-0.25174	0.83	0.72	1
2310009B:	2.37E-05	-0.25185	0.57	0.383	0.469749
Pcmt1	0.015289	-0.25199	0.452	0.342	1
Gosr2	0.008837	-0.25209	0.539	0.401	1
Polr2d	0.004881	-0.25216	0.315	0.227	1
Fcgr1	0.143222	-0.25261	0.939	0.879	1
Usp16	0.000156	-0.25262	0.388	0.264	1
Fam120a	0.032955	-0.25264	0.688	0.549	1
Leng8	1.02E-05	-0.25267	0.458	0.303	0.202823
Lrrc59	0.016559	-0.25391	0.63	0.497	1
Erp44	0.885775	-0.25394	0.736	0.607	1
Sec11a	0.505531	-0.25431	0.794	0.662	1
Ncoa4	0.742071	-0.25431	0.776	0.655	1
Nudcd3	0.006497	-0.25435	0.552	0.42	1
Tomm40	0.022162	-0.25442	0.533	0.407	1
Fdft1	0.320317	-0.25466	0.103	0.084	1
Usp15	0.000458	-0.25473	0.291	0.191	1
Ndufb6	0.601619	-0.25497	0.861	0.775	1
Rara	0.029325	-0.25528	0.291	0.217	1

Psme3	0.001305	-0.25529	0.533	0.384	1
Synj1	0.001599	-0.25534	0.627	0.476	1
Sf3b4	0.013716	-0.25579	0.576	0.436	1
Acadvl	0.54084	-0.25582	0.318	0.276	1
Zfp771	0.011905	-0.25591	0.245	0.174	1
Tbc1d14	0.001506	-0.25601	0.364	0.25	1
Dcun1d5	0.357427	-0.25669	0.782	0.642	1
Wdfy4	0.035695	-0.2567	0.476	0.373	1
Rnf2	0.151871	-0.25684	0.264	0.211	1
Kpna1	0.020645	-0.25687	0.185	0.132	1
2410001C2	0.203265	-0.2571	0.736	0.59	1
Setd5	3.69E-05	-0.2571	0.506	0.34	0.7337
Idh3b	0.586305	-0.25711	0.682	0.565	1
Ube2v2	0.030798	-0.25718	0.403	0.309	1
Rcn2	0.652044	-0.25723	0.348	0.302	1
2010107E0	0.046255	-0.25736	0.964	0.948	1
Med17	0.013444	-0.25739	0.245	0.176	1
5730494N0	0.020139	-0.25745	0.473	0.357	1
Mrpl10	0.006717	-0.25758	0.321	0.234	1
Copg	0.048509	-0.25762	0.518	0.415	1
Mrpl2	0.121118	-0.25766	0.473	0.391	1
Cdv3	0.1676	-0.25766	0.676	0.544	1
Pwwp2a	3.75E-05	-0.25773	0.27	0.163	0.745019
Smarcc1	0.000197	-0.25786	0.412	0.282	1
A530032D	4.11E-05	-0.25802	0.548	0.388	0.816899
Ppcdc	0.02819	-0.25806	0.112	0.075	1
Rhog	0.137829	-0.25815	0.924	0.847	1
Vps29	0.650935	-0.25827	0.785	0.684	1
Brix1	0.004761	-0.2583	0.403	0.292	1
Ski	0.022449	-0.25833	0.397	0.291	1
Elf2	1.15E-05	-0.2584	0.261	0.151	0.22753
Mta2	0.009661	-0.25851	0.415	0.31	1
Psmd3	0.201168	-0.25854	0.624	0.505	1
Fam134a	0.000906	-0.25854	0.57	0.414	1
Gemin7	0.886244	-0.25883	0.727	0.641	1
Ecm1	0.866815	-0.25891	0.745	0.64	1
Stt3b	0.777326	-0.25892	0.442	0.392	1
Fam46a	0.915559	-0.25899	0.812	0.703	1
Gtf3a	0.031436	-0.25905	0.6	0.473	1
Nsf	0.006345	-0.25906	0.324	0.234	1
Fbxo11	0.003553	-0.25906	0.242	0.169	1
4632428N0	0.470396	-0.25916	0.797	0.705	1
Rps26	6.17E-06	-0.25927	0.997	0.995	0.122531

5730437N(	9.77E-05	-0.25929	0.552	0.379	1
Aif1	0.091045	-0.25932	0.988	0.98	1
Seh1l	0.120699	-0.25943	0.385	0.313	1
Gm16517	0.002218	-0.25948	0.603	0.445	1
Pnkp	0.010541	-0.25948	0.379	0.274	1
Tipin	0.092269	-0.2595	0.297	0.235	1
2610101N	0.096834	-0.25979	0.624	0.493	1
Clpx	0.044457	-0.25987	0.236	0.177	1
D030056L2	0.006508	-0.25995	0.191	0.129	1
Gpaa1	0.031912	-0.26003	0.503	0.408	1
Asnsd1	0.354128	-0.26007	0.491	0.404	1
Mast2	3.56E-05	-0.26008	0.209	0.118	0.706369
Tpst2	0.267583	-0.26024	0.767	0.624	1
E2f5	0.002477	-0.26026	0.176	0.112	1
2410015M	0.224076	-0.26031	0.888	0.811	1
Zdhhc3	0.001871	-0.26034	0.418	0.301	1
Acd	2.08E-05	-0.2605	0.452	0.296	0.413142
Fbxl6	0.000201	-0.26063	0.488	0.338	1
Dym	6.47E-05	-0.26067	0.358	0.233	1
Sipa1	0.151044	-0.26068	0.539	0.446	1
Creg1	0.00881	-0.26069	0.982	0.971	1
Dnajc21	4.91E-06	-0.26072	0.461	0.297	0.097537
Naa10	0.172063	-0.26075	0.361	0.287	1
Ube2w	0.003685	-0.26077	0.409	0.294	1
Fbxo6	0.036789	-0.26087	0.688	0.528	1
Rnf13	0.627636	-0.26105	0.782	0.665	1
Mtf1	0.004229	-0.26118	0.248	0.172	1
Asf1a	0.007551	-0.26136	0.218	0.146	1
Impad1	0.03697	-0.26166	0.118	0.081	1
AI316807	0.011775	-0.26175	0.691	0.531	1
Armc1	0.00211	-0.26192	0.439	0.313	1
Rbm28	9.64E-05	-0.26193	0.491	0.33	1
Odf2	0.037213	-0.26204	0.179	0.131	1
Dynll2	0.674446	-0.26217	0.767	0.659	1
Ndufs1	0.002501	-0.2622	0.297	0.209	1
Spg7	0.000932	-0.26235	0.448	0.325	1
Dusp3	0.145676	-0.2624	0.773	0.658	1
Ddx39b	0.804886	-0.26256	0.73	0.618	1
Dnajc10	0.002693	-0.26274	0.327	0.224	1
Arhgap18	0.01619	-0.26278	0.345	0.262	1
Cryl1	0.015788	-0.26287	0.433	0.327	1
Glud1	0.200218	-0.26293	0.864	0.792	1
Tax1bp3	0.03344	-0.26298	0.582	0.455	1

Banf1	0.798755	-0.26305	0.761	0.665	1
Stip1	0.094621	-0.26309	0.624	0.494	1
Lrwd1	0.02027	-0.26327	0.215	0.155	1
Pcyt2	0.043616	-0.26328	0.2	0.149	1
BC056474	0.738352	-0.26329	0.858	0.769	1
Mrfap1	0.017644	-0.26341	0.955	0.9	1
Ccdc50	0.090724	-0.26343	0.833	0.699	1
Smpd1	0.033147	-0.26416	0.252	0.187	1
Fam188a	0.01185	-0.26417	0.361	0.266	1
Sdhd	0.604854	-0.26428	0.797	0.671	1
Trim12c	0.211127	-0.26429	0.312	0.262	1
Rps6kb1	0.031893	-0.26441	0.364	0.275	1
Arhgap30	0.209561	-0.26442	0.721	0.583	1
Gnpda2	0.002231	-0.26478	0.194	0.127	1
Phpt1	0.248699	-0.26496	0.585	0.497	1
Anks3	0.001406	-0.26511	0.133	0.078	1
Slc25a38	0.001907	-0.26527	0.188	0.118	1
Nubp2	0.225636	-0.2653	0.479	0.406	1
Mrps26	0.11	-0.26539	0.533	0.423	1
Trip6	0.003953	-0.26542	0.112	0.065	1
Rbm26	0.000787	-0.26543	0.473	0.335	1
Cops4	0.039399	-0.26619	0.664	0.51	1
Cerk	0.000249	-0.26628	0.682	0.509	1
Ppp2r1a	0.341589	-0.26667	0.615	0.509	1
Micall1	0.004668	-0.26683	0.1	0.058	1
Frmd8	0.000584	-0.26699	0.23	0.143	1
Socs7	0.111186	-0.26704	0.188	0.146	1
R3hcc1	0.106059	-0.26709	0.103	0.075	1
Atf1	0.007349	-0.26718	0.527	0.392	1
Efnb1	0.004716	-0.26722	0.152	0.095	1
Hck	0.11647	-0.26731	0.927	0.855	1
BC005624	0.002747	-0.26733	0.667	0.509	1
Rrp1	0.56097	-0.26733	0.758	0.648	1
Ndufb8	0.036543	-0.2674	0.955	0.899	1
Ptges3	0.685364	-0.26755	0.827	0.739	1
Grpel1	0.546628	-0.2679	0.691	0.595	1
Zc3h7a	0.000904	-0.2685	0.358	0.244	1
Imp4	0.015876	-0.26852	0.445	0.342	1
Plekha8	0.013056	-0.26859	0.139	0.093	1
Actr10	0.130775	-0.26867	0.652	0.535	1
Xiap	0.00059	-0.26872	0.588	0.423	1
Nxf1	0.003263	-0.2689	0.348	0.24	1
2010011I2	0.007317	-0.26906	0.155	0.101	1

Arl3	0.031078	-0.26923	0.367	0.278	1
Sppl3	0.006454	-0.26944	0.336	0.243	1
Hes6	0.00133	-0.26948	0.418	0.298	1
Adcy7	0.011521	-0.2695	0.567	0.436	1
2810432D(	0.00187	-0.26952	0.367	0.262	1
0610007L(	0.000174	-0.26953	0.664	0.478	1
Casp7	0.008133	-0.26956	0.27	0.189	1
Rnaseh2c	0.952428	-0.2696	0.664	0.565	1
C6	0.470863	-0.26967	0.455	0.439	1
Gbas	0.231647	-0.26984	0.285	0.244	1
Utp6	0.000568	-0.26992	0.239	0.152	1
Vapa	0.248811	-0.27002	0.942	0.841	1
Ccm2	0.021784	-0.27027	0.512	0.403	1
Qdpr	0.020727	-0.27051	0.588	0.452	1
Arl6ip6	0.011143	-0.27059	0.303	0.218	1
Nsmce1	0.019302	-0.27059	0.542	0.42	1
Skp1a	0.893495	-0.27103	0.736	0.639	1
Ppp2r4	0.032488	-0.27113	0.658	0.506	1
Gtf2f1	0.130669	-0.27113	0.336	0.272	1
Klhdc4	0.010557	-0.27114	0.309	0.223	1
Wdr1	0.170488	-0.27122	0.894	0.796	1
Cul4a	8.56E-05	-0.27144	0.33	0.21	1
Hadh	0.988113	-0.27161	0.152	0.142	1
Mmrn2	0.000172	-0.27184	0.133	0.071	1
Ube2d3	0.001354	-0.27186	0.973	0.934	1
Ei24	0.048494	-0.27187	0.515	0.402	1
Csk	0.320383	-0.27191	0.855	0.73	1
Clpp	0.081854	-0.27193	0.473	0.372	1
Ndufs6	0.05634	-0.27198	0.873	0.825	1
Ccdc86	0.202728	-0.2721	0.567	0.461	1
Ss18	0.000308	-0.27224	0.436	0.29	1
Mpp6	0.007803	-0.27239	0.685	0.548	1
Timm13	0.217925	-0.2725	0.918	0.862	1
Acat1	0.816046	-0.27264	0.521	0.438	1
2310009A(	0.109491	-0.2728	0.576	0.471	1
Rassf3	4.26E-05	-0.27289	0.409	0.27	0.845245
Scoc	0.018979	-0.27306	0.367	0.271	1
Fbxl8	0.004046	-0.27306	0.167	0.11	1
Pnn	0.290108	-0.27309	0.536	0.444	1
Unc50	0.044293	-0.27315	0.661	0.52	1
Dync1i2	0.064969	-0.27318	0.724	0.582	1
Cdc20	0.00026	-0.27318	0.115	0.06	1
Ranbp1	0.276988	-0.27351	0.864	0.82	1

Ndufs8	0.229368	-0.27383	0.821	0.732	1
Cebpg	0.055361	-0.27384	0.803	0.66	1
Nosip	0.25096	-0.27394	0.397	0.333	1
Akt2	0.000729	-0.27405	0.527	0.373	1
Map3k14	0.000404	-0.27424	0.267	0.171	1
Yme1l1	0.314218	-0.27424	0.315	0.261	1
Akap8	0.005552	-0.27437	0.312	0.222	1
Atg4d	0.04119	-0.27444	0.152	0.107	1
Gpr108	0.061012	-0.27446	0.576	0.47	1
Yipf4	0.400073	-0.27475	0.636	0.519	1
Vamp7	0.086936	-0.27485	0.373	0.298	1
Dapk3	0.005025	-0.27489	0.536	0.4	1
Stam	0.000302	-0.27493	0.173	0.102	1
2010012O(	0.05364	-0.27495	0.333	0.26	1
Adcy4	0.000513	-0.27508	0.409	0.284	1
Mrpl15	0.118228	-0.27521	0.582	0.468	1
Cops7a	0.003283	-0.27526	0.697	0.511	1
Zc3h11a	0.019406	-0.27562	0.476	0.356	1
Sertad2	0.246521	-0.2758	0.318	0.262	1
Lyn	0.029665	-0.27585	0.955	0.898	1
Gaa	0.017833	-0.27585	0.491	0.373	1
Mcts1	0.100681	-0.27592	0.752	0.621	1
Fam100a	0.001856	-0.27611	0.724	0.562	1
Mrpl42	0.346064	-0.27613	0.912	0.841	1
Tk2	3.93E-06	-0.27635	0.485	0.318	0.078099
1500015A(	0.113975	-0.27635	0.118	0.09	1
Lage3	0.008602	-0.27655	0.667	0.525	1
BC017612	0.540087	-0.2766	0.109	0.094	1
Man1c1	0.173168	-0.27668	0.348	0.281	1
Slc25a32	6.26E-06	-0.2768	0.185	0.098	0.12436
Mrps25	0.008257	-0.27684	0.673	0.522	1
Cisd3	0.080435	-0.27691	0.621	0.485	1
Pitpnb	0.002501	-0.27706	0.348	0.242	1
Prkcb	0.332117	-0.27715	0.77	0.653	1
Ubl7	0.474771	-0.27716	0.533	0.448	1
Epsti1	0.334007	-0.2773	0.894	0.81	1
N4bp2l1	2.50E-05	-0.27737	0.536	0.365	0.496935
E2f1	0.000323	-0.2774	0.355	0.246	1
Bak1	0.132318	-0.2774	0.779	0.645	1
Zfp869	9.78E-05	-0.27754	0.3	0.192	1
Ccdc75	0.001264	-0.27758	0.352	0.244	1
Scamp1	0.006533	-0.27796	0.297	0.219	1
Gbp9	0.032629	-0.27797	0.252	0.188	1

Rdm1	0.004886	-0.27814	0.233	0.16	1
Got2	0.194621	-0.27815	0.509	0.405	1
Ddost	0.523847	-0.27847	0.858	0.742	1
Ndufc1	0.222786	-0.2786	0.909	0.824	1
Ppp2r5d	0.001121	-0.2786	0.209	0.133	1
Psmc3	0.640465	-0.2786	0.755	0.657	1
Dus2l	0.0432	-0.27864	0.139	0.098	1
Glcci1	0.002427	-0.27864	0.148	0.089	1
Cetn3	0.857133	-0.27874	0.645	0.578	1
Aarsd1	0.000635	-0.27875	0.448	0.315	1
Ciapin1	0.002143	-0.2788	0.385	0.272	1
Shmt2	0.155126	-0.27884	0.27	0.217	1
Ppa1	0.136993	-0.27884	0.339	0.27	1
Pcbd2	0.159741	-0.27886	0.736	0.596	1
Manf	0.001511	-0.27893	0.912	0.87	1
Atg4b	0.007638	-0.27893	0.321	0.235	1
Ctage5	0.266168	-0.27904	0.77	0.647	1
Rnaseh2b	0.053074	-0.27905	0.436	0.351	1
Hmgxb3	0.081227	-0.27916	0.133	0.099	1
Aph1a	0.175684	-0.27926	0.706	0.566	1
Ddx19a	0.06337	-0.27929	0.255	0.2	1
Eif2a	0.303702	-0.27932	0.409	0.34	1
Eif2s3x	0.007392	-0.27937	0.561	0.423	1
Bet1l	0.035323	-0.27947	0.412	0.321	1
Eci1	0.021448	-0.27956	0.412	0.311	1
Tbcc	0.016131	-0.27963	0.221	0.157	1
Hspd1	0.564378	-0.27987	0.806	0.716	1
Shc1	0.011033	-0.28006	0.339	0.247	1
Psme2	0.001538	-0.2802	0.961	0.948	1
Thoc6	0.045683	-0.28025	0.252	0.189	1
BC017647	0.692226	-0.28052	0.133	0.12	1
Acbd3	0.000532	-0.28054	0.515	0.368	1
Slc35b1	0.076275	-0.28065	0.552	0.441	1
Parp2	0.000194	-0.28066	0.497	0.343	1
Fam176b	0.694091	-0.28068	0.545	0.5	1
Ttc39c	0.004414	-0.28069	0.233	0.159	1
Anapc16	0.001759	-0.28069	0.5	0.366	1
Rapgef1	0.000117	-0.28077	0.309	0.193	1
Ids	0.00223	-0.28078	0.4	0.293	1
Twistnb	0.119093	-0.28078	0.433	0.351	1
Smad4	8.40E-05	-0.28101	0.367	0.244	1
Slc25a11	0.925514	-0.28131	0.733	0.636	1
Tsc22d2	0.001082	-0.28147	0.23	0.148	1

Isg20l2	0.245456	-0.28162	0.133	0.108	1
2810405K(	0.012595	-0.28184	0.567	0.446	1
Vps4b	0.165789	-0.28205	0.648	0.521	1
Zmym5	0.11816	-0.28208	0.239	0.19	1
Pptc7	1.41E-05	-0.28216	0.248	0.143	0.280971
Galns	0.331618	-0.28222	0.124	0.105	1
Efha1	8.64E-06	-0.28248	0.379	0.237	0.171579
Trim28	0.095352	-0.28253	0.539	0.43	1
Gps1	0.003047	-0.28268	0.558	0.411	1
Tubb2a	0.575893	-0.28289	0.539	0.457	1
Cd72	0.420866	-0.28299	0.927	0.818	1
Uck1	0.000102	-0.28309	0.248	0.151	1
Nudc	0.960783	-0.28314	0.858	0.744	1
Fbxo9	0.020964	-0.28319	0.212	0.151	1
Ranbp3	0.004393	-0.28323	0.309	0.219	1
Krr1	6.09E-05	-0.28343	0.242	0.145	1
Gne	0.001547	-0.28355	0.109	0.06	1
Tmf1	0.00861	-0.28357	0.618	0.482	1
Dnajc2	0.025082	-0.28372	0.685	0.528	1
Ergic2	0.063348	-0.28375	0.582	0.459	1
Rarres1	0.000111	-0.28381	0.309	0.205	1
H2-Ke2	0.321532	-0.28389	0.724	0.66	1
Pes1	0.019042	-0.28393	0.57	0.44	1
Hmgcs1	0.372852	-0.28403	0.167	0.139	1
Bnip3	0.10241	-0.28464	0.176	0.135	1
Vps53	0.003699	-0.28465	0.248	0.172	1
Acad8	0.066114	-0.28466	0.242	0.186	1
Tmem65	0.001822	-0.28475	0.652	0.474	1
Fmnl3	6.59E-05	-0.28475	0.252	0.151	1
Tsn	0.9645	-0.28492	0.664	0.573	1
Pacs1	0.416819	-0.28493	0.103	0.087	1
Tmed4	0.013852	-0.28509	0.5	0.381	1
Polr2h	0.009962	-0.28536	0.376	0.282	1
Sep15	8.83E-05	-0.28558	0.961	0.928	1
Ezh1	6.36E-05	-0.28574	0.258	0.159	1
1110065P2	8.43E-05	-0.28586	0.427	0.287	1
Fam18b	0.009678	-0.2859	0.388	0.287	1
Ifi204	0.877076	-0.28596	0.906	0.837	1
Gstm1	0.553087	-0.28604	0.403	0.358	1
Ufm1	0.162532	-0.28611	0.661	0.537	1
Lrch4	0.00531	-0.28617	0.445	0.322	1
Dbp	0.016039	-0.28623	0.158	0.108	1
Il10ra	0.007469	-0.28624	0.624	0.499	1

Ino80e	0.034082	-0.28639	0.367	0.281	1
Mrps6	0.102253	-0.2865	0.582	0.455	1
Zc3h14	0.005221	-0.28664	0.336	0.242	1
Uxt	0.000119	-0.28675	0.6	0.44	1
Pxk	0.006059	-0.28679	0.445	0.333	1
Mphosph8	0.006839	-0.2868	0.403	0.294	1
Capza1	0.502706	-0.28684	0.77	0.663	1
Ilk	0.481945	-0.28701	0.721	0.59	1
Cyld	5.49E-05	-0.28722	0.397	0.264	1
Ahcy	0.008871	-0.2873	0.415	0.295	1
Arhgap17	0.011484	-0.28742	0.576	0.432	1
Xpa	0.000268	-0.28796	0.397	0.269	1
Myl12b	0.000142	-0.28809	0.945	0.896	1
Mrpl36	0.454811	-0.28827	0.803	0.652	1
2310033P(	0.040062	-0.28856	0.309	0.233	1
Kif16b	0.002358	-0.28863	0.155	0.097	1
Snx11	0.145121	-0.28864	0.233	0.19	1
Dnajc7	0.972703	-0.28865	0.639	0.548	1
Tor1aip2	0.010607	-0.28868	0.548	0.416	1
Pdk3	0.000153	-0.28907	0.376	0.245	1
Ensa	0.84654	-0.28909	0.682	0.591	1
Actb	0.026703	-0.28929	1	1	1
2310001A:	0.037256	-0.28943	0.245	0.185	1
Nab2	1.72E-05	-0.28949	0.548	0.374	0.341203
Api5	0.065359	-0.2895	0.542	0.426	1
Zkscan17	0.009206	-0.28959	0.164	0.111	1
Ddx18	0.003282	-0.28966	0.609	0.466	1
Taf5l	0.020961	-0.28971	0.224	0.161	1
Rbm17	0.798232	-0.28978	0.815	0.702	1
Gorasp2	0.066521	-0.2898	0.573	0.456	1
Evi5	0.115509	-0.29006	0.406	0.32	1
BC029722	0.031712	-0.29008	0.394	0.31	1
Zcchc11	6.21E-05	-0.29019	0.476	0.32	1
March7	0.003508	-0.29021	0.182	0.117	1
ORF61	0.00031	-0.29023	0.53	0.383	1
Ppil1	0.04906	-0.29025	0.242	0.184	1
Ppp1r35	0.190136	-0.29046	0.355	0.289	1
Phf14	0.012368	-0.29051	0.376	0.28	1
Pla2g16	0.944635	-0.29064	0.806	0.703	1
4933411K:	0.000575	-0.29073	0.179	0.108	1
Picalm	0.389932	-0.29073	0.861	0.729	1
2810407C(	0.744063	-0.29073	0.8	0.69	1
As3mt	0.037942	-0.29084	0.203	0.147	1

Rnf166	0.116046	-0.2911	0.658	0.527	1
Ralb	0.215174	-0.2914	0.718	0.587	1
Rab11fip2	0.000108	-0.29142	0.236	0.146	1
Mrpl11	0.001061	-0.29144	0.639	0.472	1
Lfng	0.013251	-0.29146	0.7	0.566	1
Nol12	0.000118	-0.2915	0.527	0.365	1
Dstyk	0.004053	-0.29154	0.182	0.12	1
Brox	0.049162	-0.29159	0.467	0.359	1
Saal1	0.087164	-0.29163	0.133	0.099	1
Cebpz	0.003428	-0.29175	0.524	0.396	1
Romo1	0.152309	-0.29217	0.894	0.839	1
Cdk11b	0.016583	-0.29218	0.47	0.36	1
H1f0	0.096296	-0.29227	0.488	0.381	1
Pon3	0.001757	-0.29247	0.585	0.426	1
Gnl1	0.007659	-0.29253	0.43	0.334	1
Fli1	0.026383	-0.29275	0.552	0.429	1
Hivep2	1.85E-05	-0.29277	0.215	0.122	0.367782
Ada	0.021871	-0.2928	0.37	0.273	1
AI607873	0.336879	-0.29288	0.867	0.741	1
Dhrsx	0.00169	-0.29311	0.615	0.441	1
Cdkn2aipn	6.38E-06	-0.29369	0.506	0.341	0.126692
Gng10	0.895908	-0.29371	0.518	0.446	1
Rinl	0.018686	-0.29393	0.427	0.333	1
Rufy1	0.019072	-0.29401	0.306	0.225	1
Dcaf12	0.140596	-0.29418	0.464	0.372	1
Mpi	0.013347	-0.29418	0.218	0.156	1
Wee1	0.001774	-0.29431	0.194	0.124	1
Ogfrl1	0.140267	-0.29447	0.815	0.681	1
Sptlc2	0.628001	-0.29462	0.603	0.514	1
Prdx1	7.87E-06	-0.29465	0.997	0.994	0.156391
Ppwd1	0.712239	-0.29469	0.115	0.106	1
Ermp1	0.102935	-0.29478	0.176	0.136	1
Agap1	9.63E-05	-0.29485	0.227	0.136	1
Sort1	0.009109	-0.29488	0.1	0.06	1
Samd9l	0.017788	-0.29516	0.561	0.426	1
Cat	0.751376	-0.29527	0.612	0.527	1
Lrch1	0.001226	-0.2953	0.239	0.159	1
Anpep	5.41E-05	-0.2955	0.179	0.1	1
Hgs	0.00066	-0.29599	0.403	0.278	1
Snd1	0.089325	-0.29612	0.548	0.456	1
Hspa4	0.191504	-0.29617	0.67	0.551	1
Pqbp1	0.153791	-0.2962	0.536	0.442	1
Tmed1	0.30915	-0.29632	0.106	0.087	1

Ctdsp2	0.219961	-0.29642	0.376	0.314	1
Rpa2	0.013005	-0.29673	0.248	0.18	1
Mgat2	0.228926	-0.29673	0.694	0.547	1
A930005H	0.011691	-0.29674	0.227	0.159	1
Wdr61	0.060106	-0.29709	0.521	0.401	1
Slc35a2	0.147332	-0.29713	0.261	0.211	1
Tox4	0.004586	-0.29714	0.482	0.35	1
Mrpl34	0.25192	-0.29717	0.791	0.648	1
2310004N	0.001889	-0.29741	0.761	0.579	1
Mospd2	0.021293	-0.29744	0.164	0.111	1
Cyb5r4	0.776866	-0.29752	0.506	0.435	1
Trpm7	0.007658	-0.29758	0.276	0.193	1
Casc3	0.002571	-0.29762	0.385	0.279	1
2810408I1	0.023962	-0.2979	0.121	0.082	1
Egln2	0.038222	-0.29792	0.533	0.414	1
Atp13a2	0.014777	-0.29803	0.821	0.666	1
Ndufb4	0.248646	-0.29824	0.912	0.825	1
Nub1	7.60E-05	-0.2984	0.439	0.295	1
Btbd1	0.35354	-0.29863	0.591	0.495	1
Polr2l	0.059008	-0.2987	0.873	0.82	1
Sdhb	0.020699	-0.2987	0.891	0.824	1
Trappc1	0.174781	-0.29872	0.673	0.559	1
Dcun1d1	0.00121	-0.29889	0.391	0.27	1
Uba3	0.224592	-0.29897	0.227	0.183	1
P4ha1	0.499964	-0.29928	0.694	0.58	1
Hsd17b4	0.080056	-0.29939	0.824	0.686	1
Rnf10	0.880916	-0.29943	0.564	0.492	1
0610037L1	0.005976	-0.29972	0.448	0.338	1
Cmtm3	0.076345	-0.29979	0.861	0.732	1
Tars	0.007031	-0.29983	0.415	0.317	1
Rnf5	0.019337	-0.30011	0.536	0.416	1
Brd9	0.00368	-0.30016	0.264	0.184	1
Ola1	0.04681	-0.30028	0.618	0.481	1
Elf4	0.000226	-0.30039	0.524	0.376	1
Epc1	0.001075	-0.30051	0.339	0.231	1
Serinc5	0.012617	-0.30053	0.103	0.063	1
Taf15	0.010916	-0.30054	0.442	0.33	1
Med8	0.001199	-0.3006	0.606	0.443	1
Chd9	0.120342	-0.30063	0.224	0.18	1
Fancl	0.11249	-0.30084	0.112	0.083	1
Clec4d	8.23E-06	-0.30101	0.406	0.269	0.163453
Mrpl12	0.406895	-0.30104	0.742	0.681	1
Exosc3	0.007516	-0.30124	0.397	0.288	1

Braf	0.000238	-0.30128	0.324	0.214	1
Fam65a	3.56E-05	-0.30153	0.303	0.191	0.706511
Bbip1	0.249064	-0.30163	0.533	0.45	1
Gtpbp4	0.054846	-0.3017	0.536	0.412	1
2010107H(	0.106572	-0.30186	0.618	0.503	1
Mospd1	0.056159	-0.30193	0.136	0.1	1
Sarnp	0.974811	-0.30199	0.773	0.657	1
Chac2	0.112049	-0.30199	0.112	0.082	1
Cr1l	0.218355	-0.30205	0.494	0.404	1
1810006K2	0.086189	-0.30224	0.924	0.868	1
Flad1	0.897319	-0.30236	0.158	0.154	1
Ddx42	0.035	-0.30237	0.439	0.339	1
Ehmt2	1.46E-05	-0.30289	0.688	0.491	0.289108
Aldh9a1	0.001526	-0.30341	0.512	0.378	1
Cnot8	0.002073	-0.30355	0.5	0.365	1
Rrm1	0.105604	-0.30369	0.255	0.199	1
Pak1ip1	0.017357	-0.3037	0.527	0.389	1
Rnpep	0.566734	-0.30418	0.858	0.739	1
Arhgap27	0.055661	-0.30445	0.197	0.146	1
Ext2	0.003969	-0.30452	0.406	0.301	1
Slc9a3r1	0.968853	-0.30452	0.745	0.628	1
Tm9sf3	0.207269	-0.3047	0.755	0.668	1
Mettl3	0.003176	-0.30484	0.161	0.103	1
Tmem38b	0.167673	-0.30488	0.209	0.167	1
2210016L2	6.38E-05	-0.305	0.452	0.302	1
Zcchc2	2.75E-06	-0.30512	0.455	0.291	0.054633
Tbc1d10b	2.85E-05	-0.30517	0.512	0.351	0.566159
Mphosph1	0.00847	-0.30523	0.476	0.356	1
H2-Q8	0.258968	-0.30524	0.267	0.223	1
Lsm10	0.002859	-0.30528	0.597	0.446	1
Myo7a	1.61E-05	-0.3054	0.482	0.323	0.319903
Rnf220	0.000397	-0.30558	0.461	0.321	1
Actr6	0.010561	-0.3057	0.139	0.092	1
Vav3	1.13E-05	-0.30579	0.291	0.176	0.224747
Smpdl3a	0.001364	-0.3058	0.973	0.941	1
Htatip2	0.002813	-0.30586	0.621	0.466	1
Dld	0.044402	-0.30614	0.439	0.338	1
Exosc1	0.009664	-0.30647	0.306	0.224	1
Hadhb	0.553917	-0.30663	0.518	0.433	1
Ccdc59	0.012561	-0.30666	0.521	0.39	1
Ppil2	0.157179	-0.30688	0.436	0.353	1
Myc	0.296328	-0.30707	0.27	0.224	1
Slc7a6os	0.000543	-0.30748	0.294	0.193	1

Gpr137b-p	1.76E-05	-0.30753	0.67	0.485	0.350107
Surf2	0.019388	-0.30759	0.221	0.161	1
Dusp6	0.407039	-0.30776	0.645	0.531	1
Flii	0.000912	-0.30786	0.476	0.337	1
Anxa4	0.000102	-0.30792	0.712	0.522	1
Zfc3h1	0.010177	-0.30798	0.245	0.176	1
Pdhb	0.016831	-0.30816	0.439	0.328	1
Arl6ip4	0.044279	-0.3084	0.57	0.449	1
Txnrd1	0.581648	-0.30848	0.606	0.519	1
Zfp672	0.016883	-0.30872	0.206	0.146	1
Enpp4	0.000109	-0.30875	0.406	0.269	1
Snhg3	0.545353	-0.3088	0.758	0.649	1
Aasdhppt	0.040282	-0.30898	0.288	0.219	1
Mknk1	8.74E-05	-0.30915	0.445	0.301	1
Thoc7	0.151674	-0.30932	0.676	0.549	1
Prkab1	0.002627	-0.30944	0.258	0.175	1
Tmem11	0.104389	-0.30983	0.503	0.388	1
Tpst1	0.374975	-0.30985	0.306	0.263	1
Dtnb	0.000715	-0.31001	0.17	0.101	1
Cebpa	0.303725	-0.31006	0.791	0.644	1
Plin3	0.616249	-0.31008	0.1	0.088	1
Rai12	0.012301	-0.31027	0.424	0.318	1
Tcn2	0.004704	-0.31031	0.861	0.804	1
N4bp2l2	0.090501	-0.31039	0.445	0.352	1
Slc14a1	0.037489	-0.3104	0.188	0.14	1
Oaz1	0.224813	-0.31057	0.955	0.899	1
Ttc5	0.058543	-0.31062	0.242	0.187	1
Yif1b	0.359859	-0.31091	0.7	0.578	1
Fut7	7.11E-06	-0.31115	0.33	0.203	0.141215
Mup20	0.084098	-0.31119	0.567	0.45	1
Mrps14	0.660844	-0.31137	0.842	0.727	1
Lpin2	8.26E-06	-0.31146	0.573	0.403	0.164071
Oxsr1	0.000294	-0.31159	0.255	0.162	1
Tprkb	0.002336	-0.31171	0.324	0.227	1
Lonp2	0.076852	-0.31179	0.306	0.241	1
Lrpap1	0.548664	-0.31183	0.709	0.619	1
Gngt2	0.000404	-0.31199	0.982	0.978	1
D1Ertd622	0.019349	-0.31214	0.5	0.374	1
Cs	0.566424	-0.31223	0.606	0.506	1
AB124611	0.068645	-0.31224	0.918	0.842	1
Cd302	0.072234	-0.31265	0.985	0.94	1
Prpf38a	0.002367	-0.31284	0.409	0.294	1
Mrps12	0.428182	-0.31288	0.755	0.61	1

Derl2	0.071904	-0.31318	0.697	0.539	1
Stx18	0.000803	-0.3132	0.288	0.191	1
Mthfd1	0.003312	-0.31321	0.188	0.121	1
Polr2e	0.70757	-0.31325	0.548	0.465	1
Asgr2	0.032851	-0.31332	0.112	0.076	1
Nptn	0.372214	-0.31344	0.855	0.751	1
Ntpcr	0.068263	-0.3137	0.797	0.681	1
Pdlim4	0.157188	-0.31406	0.833	0.698	1
Safb	0.005373	-0.3141	0.409	0.295	1
Commd2	0.00398	-0.31412	0.421	0.302	1
Larp7	0.028071	-0.31425	0.47	0.358	1
Pycrl	1.42E-05	-0.31425	0.488	0.321	0.281975
Ino80b	0.002439	-0.31433	0.403	0.286	1
Atp5o	0.019357	-0.31454	0.942	0.895	1
Rgl2	0.02599	-0.31463	0.364	0.282	1
Ccz1	0.007326	-0.31466	0.661	0.507	1
Znhit1	0.623697	-0.3148	0.724	0.613	1
Rce1	2.08E-05	-0.3148	0.448	0.284	0.413352
Ssr3	0.664847	-0.31483	0.791	0.647	1
Atic	0.141215	-0.31501	0.294	0.235	1
Psip1	0.078087	-0.3151	0.297	0.234	1
Pex16	0.000304	-0.31538	0.382	0.263	1
Rbmxl1	0.437835	-0.31547	0.53	0.443	1
Usp7	0.002251	-0.31573	0.355	0.248	1
Tmem127	0.015325	-0.31585	0.324	0.237	1
Rnf38	1.69E-05	-0.31588	0.276	0.162	0.334701
Tmx1	0.246399	-0.3161	0.836	0.693	1
Ccdc53	0.085407	-0.31611	0.621	0.506	1
BC003965	0.016702	-0.31619	0.388	0.291	1
Rrs1	0.058571	-0.31628	0.333	0.257	1
Trim27	0.002638	-0.31629	0.43	0.32	1
Ide	0.023191	-0.31642	0.352	0.265	1
B230120H	0.000697	-0.31648	0.167	0.098	1
Tmem222	0.130264	-0.31654	0.415	0.337	1
Ccdc115	0.022345	-0.31664	0.639	0.498	1
Fxr1	0.139942	-0.31699	0.385	0.316	1
Pou2f2	0.393657	-0.31704	0.536	0.454	1
Ppcs	0.005166	-0.31727	0.167	0.107	1
Mrpl40	0.009631	-0.31753	0.624	0.471	1
Commd6	0.20801	-0.31772	0.661	0.533	1
Man1b1	0.000775	-0.31773	0.321	0.219	1
Cmtm6	0.424101	-0.31777	0.476	0.405	1
Zbp1	0.449422	-0.31779	0.845	0.708	1

Usf1	0.028873	-0.31792	0.361	0.27	1
Spen	0.087415	-0.31793	0.242	0.19	1
Mrps34	0.075902	-0.31807	0.561	0.439	1
Mllt4	0.021161	-0.3181	0.248	0.178	1
Numa1	0.000771	-0.31812	0.436	0.312	1
Phf12	0.000839	-0.3183	0.276	0.18	1
Dnajc3	0.313954	-0.3183	0.739	0.657	1
Abhd14b	0.015868	-0.31836	0.264	0.189	1
Prkce	0.007628	-0.31852	0.103	0.062	1
Stx12	0.006568	-0.31862	0.624	0.472	1
2810001G	0.196055	-0.31869	0.115	0.09	1
Fam110a	0.24751	-0.31872	0.103	0.08	1
Fbxo22	0.041373	-0.31885	0.364	0.28	1
Abcg1	0.054607	-0.31891	0.758	0.628	1
Fdps	0.525695	-0.31893	0.355	0.304	1
Pisd	0.000281	-0.31898	0.397	0.271	1
Erh	0.02362	-0.31916	0.882	0.835	1
BC002163	0.010625	-0.31922	0.315	0.227	1
Ufsp2	0.008393	-0.31922	0.573	0.431	1
Ndufaf2	0.003115	-0.31942	0.373	0.272	1
C1d	0.612907	-0.31945	0.606	0.53	1
Mfhas1	0.000728	-0.31981	0.394	0.268	1
Tmem87b	7.94E-05	-0.31988	0.367	0.237	1
Atg3	0.840992	-0.31992	0.752	0.634	1
Bag6	0.031409	-0.32023	0.458	0.351	1
Ccndbp1	0.021874	-0.3204	0.445	0.339	1
Gnptab	0.012793	-0.3205	0.182	0.124	1
Dcaf11	0.077297	-0.3208	0.388	0.303	1
Lifr	0.037221	-0.32084	0.215	0.158	1
Atp2a2	0.048076	-0.32089	0.53	0.404	1
Suds3	0.22812	-0.32109	0.564	0.47	1
Dhrs4	0.261027	-0.3211	0.361	0.3	1
Baz2b	0.031472	-0.32113	0.27	0.202	1
Bpnt1	0.036652	-0.32132	0.264	0.198	1
Hmgcl	0.246571	-0.32142	0.548	0.45	1
Rabep1	2.64E-06	-0.32151	0.53	0.359	0.052432
Cops6	0.71175	-0.32156	0.709	0.617	1
Copz2	0.031524	-0.32156	0.294	0.214	1
Ruvbl2	0.009615	-0.32157	0.3	0.216	1
Stard10	0.727341	-0.32169	0.118	0.105	1
Aktip	0.212747	-0.32181	0.152	0.12	1
Exosc7	0.131381	-0.32186	0.321	0.259	1
Gatm	0.824197	-0.32216	0.797	0.708	1

Dtx3l	0.001479	-0.32266	0.655	0.505	1
Rusc2	0.000113	-0.32272	0.179	0.1	1
Phtf1	0.107277	-0.32273	0.109	0.08	1
Pfdn2	0.496862	-0.32279	0.855	0.749	1
Atxn7l3b	0.241561	-0.32279	0.7	0.561	1
Cox6a1	0.000584	-0.32286	1	0.984	1
Rtcd1	0.000118	-0.32296	0.361	0.24	1
Tcerg1	0.000159	-0.32327	0.433	0.291	1
Cpsf7	0.017571	-0.32328	0.239	0.176	1
Os9	0.771602	-0.32332	0.691	0.582	1
Mrpl3	0.100113	-0.32342	0.576	0.452	1
Cbl	0.00033	-0.32347	0.406	0.281	1
Birc6	0.013414	-0.32367	0.382	0.283	1
Sucla2	0.038282	-0.32385	0.409	0.315	1
Bckdha	0.224809	-0.32405	0.315	0.256	1
Cdc42ep2	0.011383	-0.32418	0.497	0.387	1
Ldlr	0.000231	-0.32434	0.297	0.19	1
Ak3	0.138761	-0.32434	0.2	0.158	1
Ifnar1	0.00473	-0.32443	0.621	0.477	1
Dnalc4	0.005516	-0.32443	0.324	0.235	1
Pigx	0.045081	-0.32444	0.47	0.377	1
Cox6a2	0.000162	-0.32452	0.17	0.097	1
Jmjd6	0.025582	-0.32456	0.276	0.204	1
Ptplad2	0.004466	-0.32464	0.694	0.548	1
Grk6	0.501086	-0.32467	0.37	0.319	1
Gtf2h1	0.000301	-0.32475	0.355	0.236	1
Gmppa	0.004394	-0.325	0.324	0.232	1
2010003O	0.068994	-0.3254	0.558	0.434	1
Slmap	0.273133	-0.32542	0.255	0.216	1
Mettl10	0.000161	-0.32567	0.264	0.166	1
Hars	0.051973	-0.32573	0.479	0.362	1
Xdh	0.034871	-0.32598	0.597	0.464	1
Lipe	0.00106	-0.32599	0.23	0.15	1
Nod1	8.14E-06	-0.32601	0.594	0.422	0.161648
Prkaca	0.001136	-0.32604	0.561	0.413	1
Mkl1	5.87E-06	-0.32608	0.4	0.256	0.116502
Epn2	0.036982	-0.32613	0.167	0.12	1
Immp1l	0.395889	-0.32617	0.552	0.459	1
Bdp1	0.005094	-0.32618	0.23	0.158	1
Dennd4c	0.0027	-0.32636	0.245	0.167	1
1300018J1	0.224749	-0.32658	0.212	0.173	1
Memo1	0.02061	-0.32714	0.255	0.191	1
Trf	9.86E-05	-0.32722	0.988	0.981	1

Hdac2	0.083536	-0.32729	0.445	0.348	1
Cops3	0.145739	-0.32729	0.321	0.258	1
Kif13a	0.321082	-0.32733	0.148	0.123	1
Camk2g	0.002962	-0.32741	0.182	0.117	1
Tram1	0.253839	-0.3275	0.794	0.688	1
Mylip	0.002196	-0.32756	0.488	0.366	1
Mzt1	0.113002	-0.32757	0.318	0.258	1
Safb2	0.003861	-0.32763	0.448	0.323	1
1500011H:	0.004274	-0.32782	0.13	0.079	1
Fam13b	0.002178	-0.32788	0.194	0.125	1
Tmem33	0.342757	-0.32798	0.688	0.556	1
Rgs19	0.169702	-0.32811	0.679	0.555	1
Ddhd1	0.000171	-0.32815	0.597	0.441	1
Tmem208	0.507699	-0.32819	0.815	0.688	1
1110002B(	0.26083	-0.32822	0.715	0.628	1
Copb1	0.221187	-0.32844	0.564	0.455	1
Traf7	0.004228	-0.32876	0.348	0.245	1
Mapk7	0.00106	-0.32885	0.264	0.174	1
Becn1	0.038088	-0.32915	0.564	0.434	1
1110059E2	0.000135	-0.32915	0.324	0.209	1
Atp5e	0.001921	-0.32919	0.988	0.979	1
Ptgr2	0.124193	-0.32938	0.276	0.221	1
B9d2	0.000677	-0.32938	0.452	0.318	1
Ssbp3	0.005475	-0.32941	0.27	0.185	1
Chmp2b	0.087884	-0.32942	0.558	0.426	1
Tmem64	0.002587	-0.32956	0.261	0.177	1
Trp53	0.777517	-0.3297	0.606	0.508	1
Mmadhc	0.138152	-0.32975	0.37	0.289	1
Diap2	0.1813	-0.3298	0.409	0.341	1
Cbr3	0.826621	-0.33001	0.1	0.101	1
Stk25	0.000592	-0.33006	0.439	0.304	1
Zeb2	0.057028	-0.33009	0.836	0.694	1
Ube2n	0.021938	-0.33036	0.894	0.824	1
Fbxo34	0.006703	-0.33044	0.218	0.149	1
Mrps30	0.020269	-0.33052	0.361	0.274	1
Snrpa	0.062203	-0.3306	0.621	0.489	1
Letm1	0.065435	-0.33065	0.455	0.354	1
Ociad1	0.961676	-0.33065	0.724	0.614	1
Hiatl1	0.099513	-0.33073	0.379	0.302	1
Avl9	0.025054	-0.33076	0.191	0.135	1
Sgta	0.047158	-0.33085	0.588	0.463	1
Hrsp12	0.150156	-0.33136	0.394	0.317	1
Mrps36	0.775209	-0.33142	0.767	0.665	1

Nat9	3.76E-06	-0.33155	0.436	0.281	0.074716
Crk	0.049918	-0.3317	0.47	0.36	1
Yeats4	0.930438	-0.33174	0.488	0.434	1
Rcsd1	0.361321	-0.33204	0.409	0.339	1
Kpna3	0.000183	-0.33208	0.373	0.255	1
Rpn1	0.988148	-0.33218	0.824	0.718	1
Qsox1	0.005386	-0.33239	0.203	0.133	1
Cops2	0.002719	-0.33254	0.448	0.326	1
Rps6kb2	0.052795	-0.33265	0.106	0.073	1
Exosc5	0.02397	-0.33272	0.597	0.468	1
Coq5	0.01271	-0.33284	0.291	0.21	1
0610007P1	0.230011	-0.33289	0.406	0.326	1
Zfp687	0.008339	-0.33314	0.118	0.073	1
Scarb2	0.98332	-0.33338	0.833	0.732	1
Dnajc12	0.010194	-0.33343	0.2	0.138	1
Thoc3	5.43E-05	-0.33366	0.345	0.22	1
Tspan31	0.396162	-0.33387	0.67	0.554	1
Rae1	0.244994	-0.33393	0.37	0.302	1
Hsd17b12	0.070647	-0.33398	0.779	0.621	1
Cyhr1	0.013073	-0.33406	0.321	0.234	1
Pml	0.000557	-0.3346	0.57	0.416	1
Stam2	0.109185	-0.33472	0.209	0.163	1
1810013D1	0.003284	-0.33483	0.521	0.38	1
6820431F2	0.143112	-0.33508	0.448	0.361	1
Psen1	0.026828	-0.33511	0.527	0.402	1
Pdcl3	0.309363	-0.33513	0.615	0.491	1
Ubtd1	0.000741	-0.33534	0.561	0.42	1
BC029214	0.267186	-0.3354	0.185	0.152	1
Phxr4	0.003068	-0.3355	0.194	0.126	1
Fam36a	0.348777	-0.33553	0.515	0.431	1
Ppme1	0.110479	-0.33563	0.224	0.174	1
Wipi2	0.034083	-0.3357	0.194	0.14	1
Cdyl	0.000495	-0.33575	0.245	0.159	1
Plscr3	0.010752	-0.33609	0.3	0.217	1
Ddx52	0.017773	-0.33649	0.288	0.211	1
Tom1l2	0.000434	-0.33671	0.103	0.052	1
Nckap1l	0.071328	-0.3368	0.782	0.649	1
Hsdl2	0.174209	-0.33699	0.233	0.186	1
Stag1	0.008138	-0.33699	0.291	0.206	1
Cnih	0.380009	-0.33711	0.579	0.47	1
Srm	0.000113	-0.33721	0.558	0.384	1
Klf7	0.002722	-0.33733	0.261	0.175	1
1810035L1	0.958234	-0.33738	0.873	0.767	1

Adrb2	0.804367	-0.33745	0.455	0.421	1
Spire1	0.008054	-0.33792	0.221	0.157	1
Tdp2	0.000382	-0.33793	0.203	0.123	1
Bcap29	0.001894	-0.33797	0.461	0.334	1
Kcp	0.000334	-0.33801	0.115	0.06	1
Mrpl20	0.19734	-0.33802	0.836	0.733	1
Wapal	0.039711	-0.33804	0.461	0.352	1
Tacc1	0.000721	-0.33805	0.455	0.315	1
Ormdl2	0.066478	-0.33812	0.673	0.539	1
5133401N(	0.259705	-0.33817	0.703	0.586	1
Strn4	0.261829	-0.33867	0.361	0.297	1
Minpp1	0.007669	-0.33872	0.421	0.311	1
Usp3	0.003146	-0.33938	0.324	0.229	1
Mcfd2	0.332484	-0.33941	0.803	0.658	1
Fam125b	0.012312	-0.33968	0.23	0.166	1
Ppm1d	0.088124	-0.33977	0.158	0.119	1
Rnf146	0.02717	-0.33994	0.445	0.339	1
Bccip	0.0733	-0.33999	0.606	0.475	1
Zdhhc12	0.029841	-0.34015	0.227	0.167	1
Rap1gds1	0.000147	-0.34028	0.633	0.447	1
Ciz1	0.022093	-0.34029	0.276	0.205	1
Rab8b	0.000428	-0.34034	0.712	0.531	1
Tmem57	0.000217	-0.34044	0.245	0.154	1
Ppfia1	0.003763	-0.34062	0.252	0.171	1
Stx8	0.002548	-0.34069	0.645	0.487	1
Pcyt1a	0.006663	-0.3407	0.291	0.21	1
Clec14a	0.001382	-0.34071	0.158	0.097	1
Pde4dip	0.000518	-0.34071	0.191	0.116	1
Cxx1c	0.001332	-0.34071	0.233	0.15	1
C130022K2	0.00023	-0.34085	0.388	0.263	1
Ube4b	0.065474	-0.34102	0.261	0.201	1
Thap11	0.014569	-0.34116	0.415	0.316	1
Xrcc4	0.024731	-0.34131	0.191	0.134	1
Impa1	0.103604	-0.34134	0.273	0.215	1
Lpin1	0.006894	-0.34151	0.145	0.093	1
Lactb	4.14E-05	-0.34156	0.464	0.306	0.823034
Elmo2	0.009719	-0.34172	0.397	0.293	1
Cic	0.008918	-0.34175	0.418	0.306	1
Ssh2	0.219512	-0.34226	0.642	0.518	1
Mrpl38	0.039629	-0.34252	0.379	0.287	1
Leng1	0.001254	-0.34258	0.258	0.168	1
Mbtps1	1.70E-05	-0.34263	0.336	0.208	0.33763
Rfc5	0.011477	-0.34282	0.248	0.178	1

Herc1	0.060073	-0.34286	0.379	0.293	1
Med28	0.059117	-0.34286	0.864	0.777	1
Zbtb20	0.15347	-0.34286	0.152	0.116	1
Kif2a	0.000819	-0.34292	0.255	0.165	1
Jmjd8	0.062032	-0.34293	0.112	0.079	1
Snapc5	0.192693	-0.343	0.755	0.629	1
Otub1	0.059642	-0.34321	0.533	0.414	1
Trim30a	0.180856	-0.34383	0.83	0.717	1
Was	0.002206	-0.34392	0.6	0.441	1
Urm1	0.091921	-0.34413	0.4	0.316	1
Cep110	0.102379	-0.34413	0.227	0.175	1
Brwd1	0.221577	-0.34465	0.206	0.166	1
Slc35f5	6.73E-06	-0.34482	0.352	0.218	0.133621
Ubtf	0.648967	-0.34496	0.533	0.443	1
1810063B(	0.025191	-0.34497	0.518	0.399	1
Tmcc1	0.062686	-0.34513	0.439	0.348	1
Nip7	0.00188	-0.34516	0.527	0.38	1
Acad9	0.066683	-0.34518	0.173	0.129	1
Slc43a2	0.02047	-0.34545	0.773	0.632	1
Tnfrsf21	0.103728	-0.34593	0.579	0.456	1
Zfp207	0.429727	-0.34596	0.6	0.477	1
Hps1	0.003771	-0.34607	0.321	0.234	1
Tsfm	0.120985	-0.34609	0.333	0.266	1
Rbm7	0.33601	-0.34622	0.767	0.664	1
Rbks	0.000831	-0.34633	0.191	0.119	1
Tmem167	0.020253	-0.34634	0.87	0.797	1
Dock8	0.443076	-0.34639	0.561	0.486	1
Ltbr	0.018204	-0.3464	0.77	0.615	1
Sf3a2	0.008242	-0.34658	0.342	0.247	1
Zfp110	0.00402	-0.34667	0.197	0.13	1
Traf2	0.012394	-0.34687	0.276	0.198	1
Ddb1	0.028301	-0.34698	0.558	0.432	1
Stag2	0.109607	-0.34737	0.564	0.464	1
Nhlrc2	0.078911	-0.34746	0.203	0.152	1
Akirin1	0.397526	-0.34755	0.552	0.452	1
9530068E(	0.407852	-0.34759	0.658	0.558	1
Hint3	0.009193	-0.34776	0.345	0.251	1
Phf3	0.006519	-0.34818	0.382	0.276	1
Uap1l1	0.020657	-0.34823	0.682	0.541	1
Dohh	0.267501	-0.34826	0.521	0.444	1
Gbe1	0.040149	-0.34859	0.176	0.128	1
Ttc35	0.013634	-0.3486	0.621	0.46	1
Hint2	0.467455	-0.34874	0.627	0.522	1

Gipc1	0.215422	-0.34901	0.542	0.44	1
Slc39a7	0.124766	-0.34913	0.648	0.532	1
Eif2b5	0.000101	-0.34914	0.4	0.262	1
Trip12	0.067769	-0.34942	0.548	0.441	1
5031439G(	4.29E-06	-0.3496	0.724	0.52	0.085124
Hectd3	0.07485	-0.34969	0.127	0.092	1
BC016423	0.01551	-0.34975	0.115	0.074	1
Polr3k	0.039647	-0.34987	0.418	0.312	1
Gm15800	4.07E-05	-0.34999	0.139	0.07	0.808378
Atp13a1	0.024478	-0.34999	0.385	0.298	1
Ebpl	5.47E-05	-0.35011	0.421	0.281	1
Sav1	0.160969	-0.35011	0.318	0.256	1
Zmat5	0.002934	-0.35031	0.591	0.442	1
Blmh	0.136315	-0.35054	0.406	0.321	1
Asah1	0.030577	-0.35059	0.945	0.884	1
Mcmbp	0.322304	-0.35064	0.503	0.418	1
Pdzd11	0.183074	-0.35072	0.633	0.526	1
Rpa3	0.063665	-0.35081	0.397	0.315	1
Pfkfb4	0.003365	-0.35083	0.236	0.16	1
2810021B(	0.106127	-0.35085	0.376	0.298	1
Ankrd54	0.003819	-0.35101	0.221	0.151	1
Esyt1	0.065545	-0.35127	0.367	0.278	1
Agpat3	0.011779	-0.35142	0.691	0.53	1
Ubap1	0.007895	-0.35158	0.388	0.278	1
Mina	0.009484	-0.3516	0.23	0.161	1
Aatk	0.004629	-0.35192	0.152	0.095	1
Cspp1	0.005163	-0.35194	0.124	0.076	1
Zfp830	0.068152	-0.35195	0.221	0.167	1
Mfn2	0.000243	-0.35202	0.2	0.12	1
Tmem97	0.000203	-0.35219	0.367	0.247	1
Abcd2	0.007802	-0.3525	0.215	0.146	1
Fez2	0.012611	-0.35257	0.576	0.435	1
Scamp3	0.058816	-0.35257	0.552	0.43	1
Aurkb	0.003563	-0.35265	0.167	0.108	1
Rabggtb	0.009266	-0.35283	0.512	0.388	1
Tmem70	0.000707	-0.35283	0.5	0.354	1
Nolc1	0.031574	-0.35305	0.409	0.312	1
2310011J0	0.043429	-0.35346	0.527	0.416	1
Pde12	0.593558	-0.35351	0.121	0.107	1
Vapb	0.999895	-0.35352	0.8	0.663	1
Lst1	5.31E-05	-0.35365	0.976	0.963	1
Cmc1	0.018692	-0.35387	0.661	0.511	1
Tiprl	0.257623	-0.35426	0.388	0.317	1

Entpd4	0.205549	-0.35462	0.224	0.181	1
Ddx46	0.009204	-0.35468	0.448	0.328	1
Vav1	0.679571	-0.35471	0.718	0.61	1
Mocos	0.005069	-0.35475	0.124	0.076	1
Svil	0.015428	-0.3548	0.179	0.124	1
1810063B(	0.000202	-0.35488	0.445	0.309	1
8430410K2	0.138711	-0.35522	0.148	0.117	1
Slc44a1	0.06525	-0.3553	0.13	0.094	1
Chchd4	0.0062	-0.3555	0.579	0.426	1
Tmem221	0.238035	-0.35557	0.142	0.116	1
Ppp1r10	0.218791	-0.35589	0.267	0.218	1
Prrc2a	0.019684	-0.35605	0.524	0.404	1
Smarcb1	0.490493	-0.35625	0.342	0.293	1
Cdk13	0.014183	-0.35629	0.315	0.228	1
Xpot	0.000238	-0.35649	0.255	0.161	1
Deaf1	0.366294	-0.35651	0.161	0.133	1
Ngfrap1	0.259712	-0.35667	0.088	0.104	1
Snrnp25	0.019132	-0.35682	0.388	0.293	1
Srpk2	0.11156	-0.35711	0.221	0.169	1
Hmgcr	0.02549	-0.35741	0.264	0.196	1
Ap1g1	0.002037	-0.35746	0.394	0.279	1
D17Wsu1(	0.676104	-0.35748	0.779	0.648	1
2310008H(	0.010941	-0.35781	0.388	0.292	1
Slc29a1	0.419284	-0.35801	0.955	0.892	1
March8	3.36E-06	-0.35824	0.485	0.31	0.066761
Chd1	0.060579	-0.35825	0.455	0.343	1
Tmco3	0.000373	-0.35827	0.242	0.154	1
Mosc2	0.238069	-0.35843	0.691	0.616	1
Skiv2l	0.002548	-0.35902	0.303	0.209	1
Rsl24d1	0.038029	-0.35916	0.482	0.367	1
Lap3	0.66096	-0.35951	0.515	0.468	1
Glo1	0.151147	-0.35964	0.542	0.426	1
Stx16	0.249382	-0.35968	0.63	0.52	1
Acly	0.192462	-0.35968	0.676	0.537	1
Pdk1	0.00323	-0.35977	0.318	0.222	1
D10Jhu81e	0.006497	-0.35979	0.476	0.346	1
Ddx50	0.821361	-0.35981	0.294	0.265	1
Stx6	0.006198	-0.35993	0.339	0.243	1
Mrpl16	0.000506	-0.35993	0.436	0.305	1
Chd6	0.010317	-0.36014	0.239	0.169	1
Rab11fip5	0.0003	-0.36032	0.17	0.097	1
Mrpl44	0.071747	-0.36042	0.315	0.244	1
Scfd1	0.503254	-0.36047	0.279	0.241	1

Psmc1	0.883489	-0.36083	0.733	0.635	1
Cdipt	0.000836	-0.36145	0.648	0.489	1
1110004E(	0.075855	-0.36153	0.173	0.13	1
Pold1	0.012958	-0.36166	0.185	0.127	1
0610030E2	0.000917	-0.36169	0.509	0.361	1
Tsg101	0.591268	-0.36198	0.648	0.539	1
Pigy	0.000876	-0.36227	0.524	0.369	1
Magt1	0.061504	-0.36227	0.718	0.58	1
Mrpl27	0.135281	-0.36232	0.697	0.562	1
Kcnq1ot1	0.849817	-0.36264	0.342	0.299	1
Gm13157	0.000644	-0.36266	0.394	0.271	1
Bank1	0.012518	-0.36289	0.545	0.422	1
Casp6	0.000439	-0.36302	0.279	0.18	1
Zfp800	0.020441	-0.36303	0.412	0.309	1
Cd68	0.003455	-0.36318	0.955	0.934	1
Abhd14a	2.62E-05	-0.3633	0.17	0.09	0.521179
Gm5617	0.00068	-0.36343	0.667	0.493	1
Chuk	0.1412	-0.36356	0.252	0.201	1
Lars	0.005104	-0.36462	0.439	0.322	1
Paip2b	0.027296	-0.36475	0.118	0.08	1
Agps	0.004175	-0.36502	0.361	0.257	1
Aifm1	0.000883	-0.36528	0.382	0.265	1
Gm6222	0.12312	-0.3654	0.464	0.365	1
Ngdn	0.340308	-0.36546	0.494	0.403	1
Tmem50b	0.020693	-0.36558	0.315	0.237	1
Slc11a1	0.788688	-0.36582	0.942	0.895	1
Pigq	0.123678	-0.36589	0.236	0.186	1
4930455F2	0.062199	-0.36591	0.1	0.07	1
Dok2	0.24586	-0.36597	0.87	0.763	1
Dcaf7	0.000473	-0.36619	0.433	0.308	1
Tmem192	0.005168	-0.36628	0.752	0.587	1
Zcchc9	0.024538	-0.3663	0.376	0.281	1
Fam134c	0.046643	-0.36644	0.342	0.261	1
Coq10a	0.024669	-0.36646	0.282	0.21	1
Smarcad1	0.000518	-0.36667	0.236	0.15	1
Prkacb	0.015864	-0.36671	0.427	0.321	1
Ppid	0.058436	-0.3668	0.376	0.288	1
Afg3l1	0.017823	-0.36683	0.467	0.36	1
Stom	0.631793	-0.36683	0.718	0.601	1
Timm44	0.016334	-0.36699	0.503	0.377	1
Bckdk	0.036701	-0.36742	0.403	0.31	1
Atpaf1	0.010637	-0.36749	0.148	0.097	1
D15Ertd62	0.000354	-0.36762	0.497	0.351	1

Slamf6	0.006068	-0.36797	0.124	0.078	1
Slc25a10	0.042334	-0.368	0.303	0.234	1
Ap1b1	0.00026	-0.36815	0.636	0.462	1
Ilkap	0.078776	-0.36833	0.497	0.394	1
Mpv17l2	0.052238	-0.36878	0.594	0.467	1
Appl1	0.133507	-0.36906	0.233	0.186	1
Ehbp1l1	0.159945	-0.36909	0.727	0.579	1
Mnat1	0.089124	-0.36915	0.267	0.213	1
Coro7	0.003826	-0.36919	0.461	0.352	1
Cops5	0.727545	-0.36923	0.47	0.434	1
Nmi	0.119889	-0.36934	0.588	0.469	1
Snx17	0.9299	-0.36935	0.73	0.632	1
2810008M	0.657757	-0.36961	0.488	0.441	1
Ctps2	0.034919	-0.36967	0.273	0.201	1
C80913	0.237893	-0.36969	0.261	0.217	1
Fcrl1	0.004044	-0.36971	0.3	0.208	1
Mrps18c	0.943433	-0.36972	0.785	0.658	1
Xrcc6	0.234434	-0.36989	0.179	0.146	1
Praf2	0.000493	-0.36989	0.276	0.179	1
Ift52	0.000161	-0.37056	0.421	0.287	1
Fndc3b	0.103209	-0.37075	0.358	0.286	1
Ctso	0.250117	-0.37083	0.397	0.328	1
Pvr	7.03E-05	-0.37168	0.261	0.161	1
Tsen34	0.602666	-0.37174	0.627	0.51	1
Nit2	0.033938	-0.37177	0.279	0.208	1
Chst12	0.586965	-0.37182	0.1	0.105	1
Tssc1	0.102569	-0.37187	0.276	0.216	1
Acp2	0.805985	-0.37225	0.852	0.754	1
Atg5	0.000582	-0.37235	0.6	0.439	1
Ugp2	0.662189	-0.37245	0.597	0.494	1
Ostc	0.369559	-0.37253	0.836	0.73	1
Ranbp2	0.013118	-0.37267	0.327	0.234	1
Vta1	0.10639	-0.37274	0.467	0.384	1
Pdcd2l	1.54E-05	-0.37287	0.524	0.351	0.305157
Actl6a	0.007196	-0.37293	0.445	0.327	1
Hnrnpul1	0.056386	-0.37307	0.127	0.09	1
Rpain	0.000536	-0.37319	0.403	0.275	1
Malt1	0.001217	-0.37319	0.121	0.068	1
Glrx2	0.014002	-0.37333	0.667	0.518	1
Ssr1	0.455262	-0.3734	0.706	0.625	1
Grhpr	0.131313	-0.37351	0.106	0.078	1
Trrap	0.004311	-0.37357	0.309	0.215	1
Utp18	0.051789	-0.3736	0.3	0.229	1

Chmp1b	0.284094	-0.37373	0.476	0.386	1
Nme3	0.948401	-0.37382	0.194	0.185	1
Psmd5	0.008812	-0.3739	0.264	0.189	1
Csnk2a1	0.302183	-0.37392	0.597	0.472	1
Ggct	0.013295	-0.37425	0.339	0.249	1
Taok1	0.109166	-0.37427	0.436	0.365	1
Slc35c1	0.020044	-0.37437	0.23	0.166	1
Fbxo8	0.022888	-0.37441	0.236	0.176	1
Ilf3	0.979813	-0.37481	0.255	0.233	1
G3bp2	0.998373	-0.37493	0.694	0.6	1
2900092E1	0.220097	-0.37547	0.536	0.434	1
Smchd1	0.140624	-0.37559	0.579	0.464	1
Kif1c	0.000541	-0.37561	0.427	0.292	1
Sel1l	0.024527	-0.37568	0.43	0.33	1
Tank	0.000347	-0.37575	0.579	0.408	1
Rad51ap1	8.81E-06	-0.37602	0.173	0.089	0.174976
Abcd3	0.014438	-0.37613	0.361	0.266	1
Ipo5	0.000193	-0.37619	0.421	0.287	1
Fbxl12	0.805844	-0.37626	0.121	0.112	1
Fastk	0.009551	-0.37646	0.318	0.232	1
4932438A1	0.00072	-0.37649	0.285	0.188	1
Manbal	0.037984	-0.37653	0.558	0.441	1
1700034H1	0.897687	-0.3766	0.191	0.181	1
Rlim	0.001314	-0.37661	0.403	0.293	1
Parl	0.689507	-0.37662	0.458	0.397	1
Clstn3	0.001033	-0.37674	0.264	0.175	1
Mob3c	0.028362	-0.37688	0.348	0.266	1
Idh2	0.361019	-0.37692	0.648	0.533	1
Atp2c1	0.144021	-0.37714	0.461	0.377	1
Mtap1s	2.47E-05	-0.37726	0.394	0.26	0.491295
Srsf10	0.652465	-0.37728	0.555	0.467	1
Itga4	0.004013	-0.37728	0.494	0.35	1
Xpr1	0.034723	-0.37734	0.282	0.212	1
Rdbp	0.002942	-0.37739	0.479	0.351	1
Larp1	0.429037	-0.37744	0.548	0.459	1
Adssl1	0.125531	-0.37748	0.355	0.285	1
Lpgat1	0.287991	-0.37748	0.282	0.235	1
Adrbk2	0.000282	-0.37773	0.497	0.35	1
Calhm2	0.06001	-0.37784	0.473	0.365	1
Arid1b	0.128774	-0.37807	0.227	0.178	1
Dram1	0.022042	-0.3789	0.37	0.278	1
1500011K1	0.100575	-0.37902	0.752	0.625	1
Ube2e3	0.472266	-0.37918	0.506	0.426	1

Fbxw5	0.023427	-0.37933	0.315	0.235	1
Irf9	0.821268	-0.37944	0.433	0.376	1
1110049F1	0.002276	-0.37949	0.358	0.243	1
Tufm	0.086536	-0.37976	0.397	0.316	1
Hivep1	0.032417	-0.37989	0.273	0.203	1
Lancl1	0.005468	-0.38022	0.242	0.166	1
Smg6	0.002138	-0.38026	0.203	0.131	1
4933433P1	0.025517	-0.38087	0.391	0.295	1
Cml1	0.137817	-0.38114	0.152	0.115	1
Ptcd2	8.36E-05	-0.3815	0.512	0.355	1
Fbxl15	0.0076	-0.3815	0.206	0.14	1
Ncstn	0.00197	-0.38193	0.627	0.473	1
Kpnb1	0.102148	-0.38237	0.582	0.452	1
2410003K1	0.010565	-0.38242	0.6	0.45	1
Atad1	0.026431	-0.38252	0.376	0.281	1
Traf3	0.003625	-0.38271	0.206	0.137	1
2510003E0	0.000246	-0.38276	0.3	0.194	1
Pias1	0.000353	-0.38281	0.503	0.352	1
Zfp593	0.045537	-0.38282	0.358	0.272	1
Fam117b	0.009217	-0.38285	0.306	0.225	1
Esyt2	0.919854	-0.38291	0.112	0.106	1
Zfp592	0.061033	-0.38305	0.233	0.176	1
Pcm1	0.879188	-0.3831	0.379	0.347	1
Hcfc1	0.008267	-0.38311	0.352	0.256	1
Sft2d2	0.023705	-0.38331	0.448	0.349	1
9430038I0	0.000476	-0.38392	0.43	0.308	1
Phf21a	7.23E-05	-0.384	0.342	0.221	1
Hdac4	0.000403	-0.38408	0.121	0.064	1
Akap11	0.01224	-0.38409	0.158	0.105	1
Me2	0.009953	-0.38411	0.685	0.541	1
Ncoa1	0.000721	-0.38413	0.327	0.22	1
Ppif	0.292004	-0.38439	0.118	0.095	1
Pid1	0.001508	-0.38446	0.542	0.396	1
Park7	0.00383	-0.3845	0.909	0.877	1
Ltv1	0.024254	-0.38469	0.37	0.285	1
Orai3	0.00484	-0.38474	0.43	0.308	1
Hnrnpr	0.074568	-0.38477	0.485	0.383	1
1700081L1	0.0467	-0.38484	0.382	0.301	1
Eif2s3y	0.224949	-0.38488	0.239	0.195	1
Echdc1	0.520002	-0.38495	0.124	0.107	1
Tceal8	0.948544	-0.3853	0.167	0.155	1
Htatsf1	5.97E-05	-0.38541	0.448	0.302	1
Trappc6b	0.672846	-0.38581	0.688	0.61	1

Clptm1l	0.42343	-0.38585	0.791	0.69	1
Btbd10	0.639815	-0.38589	0.106	0.095	1
Golim4	0.118249	-0.38597	0.206	0.158	1
Snx12	0.005068	-0.38607	0.506	0.376	1
Ccdc28b	0.169649	-0.38641	0.261	0.206	1
Clip1	0.043331	-0.38655	0.382	0.288	1
Tmem5	0.04799	-0.38672	0.409	0.322	1
Slc39a3	0.000491	-0.38686	0.206	0.125	1
Ino80d	0.001943	-0.38686	0.291	0.203	1
Mtx1	0.051456	-0.38692	0.47	0.366	1
Tmem214	0.022353	-0.38719	0.561	0.438	1
Fars2	0.000827	-0.38742	0.245	0.163	1
D14Abb1e	0.013143	-0.38792	0.273	0.195	1
Aga	0.003172	-0.38798	0.618	0.456	1
Rnf20	0.000819	-0.38812	0.333	0.226	1
Rpa1	0.0282	-0.38832	0.376	0.289	1
Cpsf3l	0.032478	-0.38836	0.255	0.188	1
Rnf115	0.165565	-0.38848	0.636	0.506	1
Tmcc3	2.19E-05	-0.38875	0.633	0.452	0.435597
Tug1	0.146714	-0.38876	0.233	0.186	1
Lrp6	2.12E-05	-0.38878	0.582	0.403	0.421963
Smarca4	0.02229	-0.38888	0.564	0.431	1
Lpp	0.119352	-0.38907	0.233	0.181	1
Gpbp1l1	0.000849	-0.38909	0.324	0.211	1
Carkd	0.28556	-0.3892	0.476	0.395	1
Xpo7	3.00E-05	-0.38928	0.33	0.206	0.59573
Lsg1	0.048886	-0.38938	0.248	0.189	1
Snx1	0.174963	-0.38943	0.579	0.466	1
Ccdc117	0.307537	-0.38953	0.167	0.14	1
Suz12	0.002533	-0.3897	0.352	0.251	1
Ccdc55	0.024338	-0.38983	0.345	0.257	1
Uba5	0.006225	-0.38994	0.473	0.339	1
Iqsec1	0.00171	-0.39003	0.294	0.199	1
Fcgr4	0.073238	-0.39017	0.979	0.937	1
Comtd1	1.19E-05	-0.39038	0.558	0.38	0.236905
Pde6d	6.08E-05	-0.39045	0.467	0.314	1
Pxmp4	0.000558	-0.3905	0.188	0.114	1
Nubp1	0.017265	-0.39051	0.545	0.417	1
Dhps	6.83E-06	-0.39058	0.367	0.228	0.135636
Mysm1	6.62E-05	-0.39071	0.267	0.158	1
Timm50	1.51E-05	-0.39083	0.373	0.238	0.300678
Zfyve21	0.000293	-0.39121	0.188	0.11	1
Tbl1xr1	0.008731	-0.39125	0.3	0.216	1

Ehmt1	0.029031	-0.39177	0.23	0.171	1
Tmx2	0.00415	-0.39186	0.339	0.236	1
Coro1c	0.00025	-0.39194	0.585	0.423	1
Apoo	0.020479	-0.39218	0.33	0.25	1
Med11	0.087773	-0.39224	0.306	0.238	1
Aldh3b1	0.027748	-0.39247	0.73	0.58	1
Mapk8ip3	0.001732	-0.39279	0.133	0.079	1
Rgs14	0.111407	-0.39281	0.136	0.102	1
Skil	0.515771	-0.39292	0.658	0.546	1
Gnpnat1	0.044706	-0.39354	0.206	0.152	1
Sdc3	0.005499	-0.39382	0.988	0.964	1
Pknox1	0.000193	-0.394	0.245	0.151	1
Ccdc97	0.125073	-0.39408	0.242	0.193	1
1700037H(	0.001214	-0.39414	0.306	0.209	1
1810074P2	0.063834	-0.39422	0.239	0.186	1
Trim35	0.001157	-0.39481	0.324	0.221	1
Glod4	0.228643	-0.39485	0.539	0.44	1
Slc48a1	0.878045	-0.39497	0.845	0.766	1
Pldn	8.27E-05	-0.39599	0.345	0.229	1
Slc16a9	0.056248	-0.39604	0.267	0.206	1
Vwa5a	0.099482	-0.39613	0.533	0.443	1
Plekha3	0.004837	-0.3962	0.315	0.221	1
Far1	0.024719	-0.39648	0.185	0.132	1
Mrps15	0.524278	-0.39648	0.679	0.584	1
2900053A1	0.531951	-0.39662	0.785	0.668	1
Dbndd2	0.220426	-0.39663	0.83	0.68	1
Rnf216	0.013395	-0.39721	0.345	0.255	1
Prps2	0.002457	-0.39725	0.3	0.208	1
Tmem41b	0.004837	-0.39726	0.248	0.171	1
Med15	9.08E-05	-0.39742	0.37	0.24	1
Smad2	1.05E-05	-0.3975	0.506	0.331	0.208943
Bad	0.263887	-0.39777	0.488	0.401	1
Ppp4c	0.191228	-0.39811	0.782	0.697	1
Rnf25	0.472613	-0.39859	0.164	0.143	1
2610005L(	0.007183	-0.39865	0.3	0.213	1
Zfp511	0.000899	-0.39896	0.27	0.178	1
Ebna1bp2	0.400245	-0.39897	0.603	0.495	1
Ncoa6	0.003368	-0.39899	0.242	0.164	1
Klhdc10	1.82E-05	-0.399	0.297	0.183	0.361464
Pycr2	0.267604	-0.3992	0.503	0.413	1
Ube2j2	0.751107	-0.39928	0.482	0.428	1
2510012J0	0.000268	-0.39935	0.2	0.121	1
Ttc4	0.021229	-0.39983	0.194	0.137	1

Foxn3	0.118685	-0.39995	0.464	0.371	1
Dnaja3	0.181131	-0.40023	0.261	0.215	1
Rab31	0.048032	-0.40035	0.497	0.386	1
Amotl1	0.016439	-0.40061	0.109	0.069	1
Ube2d1	5.34E-06	-0.40092	0.43	0.27	0.106137
Srebf1	0.126318	-0.40111	0.233	0.186	1
Nat15	0.03266	-0.40132	0.455	0.362	1
Lclat1	0.00251	-0.40141	0.13	0.077	1
Zc3h13	0.006662	-0.40163	0.391	0.278	1
Ccdc101	0.004233	-0.40166	0.391	0.284	1
Fen1	0.184564	-0.40166	0.252	0.211	1
Rnmt	0.094878	-0.40167	0.297	0.234	1
Med19	0.021742	-0.40173	0.227	0.163	1
Zc3hc1	0.00365	-0.40177	0.23	0.158	1
Irak1	0.12785	-0.40239	0.7	0.558	1
Med1	0.000145	-0.40248	0.406	0.276	1
Med29	0.007249	-0.4026	0.588	0.441	1
2310010J1	0.013218	-0.40279	0.155	0.103	1
Filip1l	0.096656	-0.40281	0.77	0.699	1
Zc3h10	7.04E-06	-0.40298	0.252	0.144	0.139859
Atp5k	0.004293	-0.40311	0.845	0.782	1
Trip11	0.206381	-0.40311	0.236	0.19	1
Utp15	0.110755	-0.40342	0.17	0.13	1
Kpna4	0.400882	-0.40377	0.515	0.439	1
Snapc2	6.57E-06	-0.40381	0.312	0.187	0.130502
Akt1s1	0.101581	-0.40396	0.391	0.306	1
Eefsec	0.507798	-0.40409	0.255	0.223	1
Erlin1	0.007615	-0.40416	0.261	0.186	1
Usp14	0.07661	-0.40432	0.567	0.448	1
Ankrd10	0.016068	-0.40446	0.203	0.14	1
Clcn3	0.010808	-0.40461	0.318	0.228	1
Eed	0.011953	-0.40463	0.406	0.303	1
C87436	0.000538	-0.40464	0.318	0.214	1
Gnpat	0.001918	-0.40472	0.273	0.187	1
Mrps35	0.218126	-0.40483	0.352	0.288	1
Gnpda1	0.003009	-0.40486	0.697	0.542	1
Cnpy3	0.12171	-0.40492	0.691	0.56	1
Jkamp	0.13691	-0.40498	0.352	0.278	1
Snx14	0.24766	-0.40506	0.179	0.148	1
Napg	0.025254	-0.40525	0.497	0.383	1
Arfrp1	0.026095	-0.40543	0.442	0.326	1
Ccdc88b	0.00027	-0.40544	0.488	0.339	1
Hipk3	0.076151	-0.40558	0.312	0.237	1

Mgat1	0.079512	-0.4056	0.515	0.404	1
Ell	0.018136	-0.40564	0.252	0.18	1
Slamf8	7.04E-05	-0.40567	0.736	0.545	1
Gcdh	0.099491	-0.40587	0.215	0.166	1
Ap1m1	0.014479	-0.4062	0.424	0.305	1
Mrpl41	0.028755	-0.40624	0.503	0.385	1
Ube2q2	0.041944	-0.40627	0.273	0.209	1
Cdc14b	0.00556	-0.40639	0.115	0.07	1
Snrnp200	0.001588	-0.40639	0.373	0.259	1
Wdr18	0.016367	-0.40649	0.394	0.293	1
Gm14005	0.055591	-0.40705	0.421	0.341	1
Clk2	0.002945	-0.40717	0.191	0.122	1
2610039C1	0.318588	-0.40734	0.267	0.224	1
Mrps7	0.842602	-0.40755	0.53	0.462	1
Cnot3	0.024851	-0.40776	0.309	0.229	1
Dpy30	0.30339	-0.40778	0.618	0.566	1
2810002N0	0.003508	-0.40812	0.473	0.349	1
Ndufaf3	0.494059	-0.40819	0.23	0.194	1
Smad5	0.003558	-0.40829	0.236	0.158	1
Pbrm1	0.831319	-0.40861	0.524	0.462	1
Snrnp40	0.007034	-0.40873	0.403	0.292	1
Shb	0.000235	-0.40939	0.215	0.13	1
Hyi	6.61E-05	-0.40945	0.258	0.159	1
Gbp3	0.146773	-0.4095	0.67	0.547	1
Evi2a	0.814573	-0.40953	0.83	0.724	1
Nmt1	0.00026	-0.40981	0.555	0.401	1
Triap1	0.022852	-0.40983	0.609	0.465	1
Lats2	0.041636	-0.41046	0.27	0.2	1
Ngrn	0.056414	-0.41093	0.248	0.19	1
1810030O0	4.10E-05	-0.41102	0.467	0.32	0.813797
Setx	0.024857	-0.41114	0.191	0.137	1
Prpf6	0.020748	-0.41135	0.252	0.182	1
Sfswap	0.007862	-0.41149	0.188	0.126	1
Tgds	0.453607	-0.41179	0.155	0.133	1
Pigs	0.000476	-0.41202	0.455	0.317	1
Nob1	0.014632	-0.4121	0.273	0.197	1
Spsb2	0.02233	-0.41231	0.455	0.345	1
Sc5d	0.427267	-0.41234	0.136	0.116	1
Dcaf17	0.83648	-0.41244	0.148	0.138	1
Dars	0.063993	-0.41254	0.467	0.361	1
Mphosph6	0.011124	-0.41258	0.276	0.199	1
Ikbip	0.004703	-0.4129	0.203	0.135	1
Dusp10	0.00062	-0.41304	0.258	0.163	1

1810043H(	0.04444	-0.41336	0.755	0.593	1
Ppa2	0.207321	-0.41348	0.255	0.206	1
Dtx2	0.027724	-0.41382	0.158	0.111	1
Sin3a	0.064616	-0.41402	0.227	0.175	1
Ptp4a3	0.083792	-0.41443	0.148	0.174	1
Sirt7	0.227165	-0.41465	0.5	0.395	1
Mtx2	0.239896	-0.4151	0.57	0.475	1
BC003266	0.043742	-0.41514	0.542	0.414	1
Mrps9	0.298692	-0.4152	0.327	0.273	1
Pdia6	0.000885	-0.41572	0.891	0.821	1
Tjap1	0.091759	-0.41574	0.127	0.093	1
Mfsd10	0.015587	-0.41584	0.424	0.308	1
Atad2b	0.220073	-0.41637	0.109	0.086	1
Gbp2	0.013585	-0.41647	0.645	0.51	1
Dctpp1	8.56E-05	-0.41677	0.576	0.389	1
Pex7	0.002366	-0.41692	0.385	0.273	1
Siglec1	0.109307	-0.41699	0.37	0.302	1
Dimt1	0.254717	-0.41738	0.145	0.116	1
Osgin2	0.185657	-0.41743	0.158	0.126	1
Ergic1	6.61E-05	-0.41764	0.485	0.334	1
Acyp1	0.004191	-0.41776	0.27	0.183	1
Treml2	0.001244	-0.41783	0.215	0.14	1
Apeh	0.324633	-0.41814	0.255	0.211	1
Hpgd	5.27E-06	-0.41825	0.967	0.95	0.104753
E4f1	0.395034	-0.41854	0.13	0.11	1
Apip	0.000814	-0.41881	0.485	0.345	1
Nfyb	0.028414	-0.41887	0.209	0.151	1
Fasn	0.427512	-0.41952	0.164	0.14	1
Ppdpf	0.07641	-0.41986	0.83	0.745	1
Tmem107	0.004221	-0.41988	0.173	0.11	1
2310022B(	0.074099	-0.42008	0.167	0.126	1
Sfxn3	0.086149	-0.42013	0.215	0.162	1
5930416I1	0.001617	-0.42021	0.248	0.167	1
Dera	0.007114	-0.42039	0.439	0.328	1
Sar1b	0.34073	-0.42047	0.685	0.609	1
Wdr70	0.036088	-0.42092	0.233	0.174	1
Emilin2	0.112979	-0.42099	0.539	0.432	1
Scyl1	0.021968	-0.42138	0.415	0.314	1
Tlr12	0.005951	-0.42206	0.388	0.285	1
Cpsf6	0.011366	-0.42282	0.542	0.396	1
Pltp	0.000359	-0.42283	0.945	0.913	1
2210411K:	0.360801	-0.42335	0.139	0.115	1
Tmem106(	0.0052	-0.42342	0.655	0.501	1

Snrpd3	0.286817	-0.42343	0.788	0.691	1
Tspan14	0.019063	-0.42348	0.436	0.319	1
Tmem128	0.206759	-0.42357	0.506	0.411	1
Fmo5	0.499517	-0.42369	0.097	0.105	1
Atxn7l3	0.002018	-0.42377	0.267	0.178	1
Gm561	0.036631	-0.42388	0.53	0.413	1
Gm3219	0.084575	-0.42434	0.185	0.141	1
Ddx1	0.995558	-0.42456	0.294	0.274	1
Heatr6	0.003461	-0.42462	0.248	0.167	1
Pigt	0.655535	-0.42465	0.567	0.48	1
Crtap	0.126965	-0.425	0.233	0.185	1
Ptcd3	0.050825	-0.42504	0.182	0.135	1
Ampd3	0.013344	-0.4252	0.167	0.113	1
Iars2	0.00865	-0.42524	0.206	0.144	1
Rhebl1	0.021376	-0.42564	0.124	0.084	1
Fbxw2	0.916912	-0.4259	0.409	0.373	1
Rangrf	0.04368	-0.42612	0.124	0.087	1
Creb1	0.03725	-0.42618	0.403	0.31	1
Rrp15	0.037759	-0.42624	0.27	0.205	1
Rfc1	0.011007	-0.4266	0.485	0.37	1
Psmf1	0.007235	-0.42682	0.345	0.246	1
Cbx7	0.197139	-0.42702	0.13	0.102	1
Ercc1	0.015489	-0.42705	0.306	0.221	1
Myof	0.000192	-0.42719	0.503	0.351	1
Mthfs	0.000729	-0.4274	0.424	0.297	1
Thumpd1	0.935533	-0.42763	0.194	0.181	1
Mrpl45	0.053231	-0.42825	0.352	0.281	1
Ppan	0.004571	-0.42837	0.388	0.285	1
Ccdc90a	0.013739	-0.42852	0.521	0.4	1
Rps6ka1	0.85716	-0.42858	0.424	0.375	1
Pydc3	0.061996	-0.42867	0.555	0.46	1
Sdhaf2	0.001735	-0.42886	0.467	0.336	1
Fam172a	0.000737	-0.42889	0.382	0.266	1
Ccdc47	0.783246	-0.429	0.406	0.353	1
Hbs1l	0.831353	-0.4291	0.145	0.135	1
Cnot6	0.00087	-0.42916	0.182	0.111	1
Nr2c2ap	0.024925	-0.42917	0.524	0.418	1
Nipa2	0.399515	-0.42951	0.712	0.598	1
Mrps10	0.012441	-0.42958	0.388	0.294	1
Phospho2	0.117325	-0.42974	0.227	0.178	1
Pop7	0.009346	-0.42981	0.476	0.353	1
Fam129a	0.009222	-0.42985	0.612	0.474	1
Ankrd13c	0.008017	-0.4299	0.13	0.082	1

Hipk1	0.24842	-0.42996	0.391	0.318	1
Nisch	0.030476	-0.43008	0.406	0.307	1
Exosc8	0.02728	-0.43092	0.427	0.32	1
Ep400	0.000551	-0.43116	0.412	0.287	1
Dmap1	0.13576	-0.43176	0.142	0.11	1
Eci2	0.444238	-0.43176	0.533	0.44	1
Fut11	0.051028	-0.43212	0.145	0.105	1
Dnajc17	0.00251	-0.43216	0.227	0.151	1
Cacybp	0.516505	-0.43246	0.718	0.643	1
Idh3a	0.340511	-0.43255	0.439	0.367	1
Rpusd4	0.000172	-0.43271	0.233	0.144	1
Gabpb2	0.068816	-0.43276	0.206	0.154	1
Actr1b	0.122034	-0.43289	0.179	0.14	1
Nudt9	0.346273	-0.43292	0.73	0.595	1
Golt1b	0.005369	-0.43351	0.455	0.336	1
Timm10	0.097498	-0.43365	0.509	0.411	1
Tmem179l	0.703314	-0.43367	0.721	0.631	1
Qtrt1	0.020668	-0.43386	0.167	0.114	1
Dusp23	1.10E-05	-0.43412	0.458	0.309	0.219228
Egln1	0.007514	-0.43419	0.245	0.174	1
Xlr	0.001875	-0.43447	0.791	0.644	1
Ndufa3	2.92E-05	-0.43455	0.936	0.919	0.58032
Mad2l1	7.63E-05	-0.43463	0.248	0.149	1
Ddx55	0.130198	-0.43507	0.136	0.105	1
Cep192	0.004947	-0.43518	0.206	0.138	1
Brap	0.005878	-0.43536	0.239	0.163	1
Zranb1	0.226234	-0.43549	0.112	0.088	1
Cntln	0.000983	-0.43571	0.233	0.153	1
Phf20	0.021734	-0.43595	0.409	0.305	1
Fam54b	0.019821	-0.43606	0.548	0.412	1
Pik3cd	0.000156	-0.43614	0.57	0.4	1
Ccs	0.245628	-0.4366	0.455	0.377	1
St6gal1	0.009041	-0.43674	0.285	0.207	1
R3hdm2	0.00029	-0.43674	0.494	0.348	1
Dync1li1	0.915263	-0.43679	0.103	0.098	1
Mrpl46	0.17143	-0.43731	0.303	0.24	1
Plk1s1	0.263728	-0.43754	0.139	0.113	1
Ammecr1l	0.01353	-0.43776	0.361	0.269	1
Cpsf2	0.004074	-0.43823	0.376	0.271	1
Phyh	0.167083	-0.43855	0.539	0.42	1
Vps13b	0.0058	-0.43873	0.236	0.165	1
2610002J0	0.043344	-0.43883	0.412	0.322	1
Ykt6	0.140029	-0.43893	0.482	0.398	1

Hspb11	0.018761	-0.43899	0.394	0.286	1
Cd320	0.035652	-0.43907	0.176	0.129	1
Mvd	0.000327	-0.43937	0.218	0.136	1
Prmt2	0.120783	-0.43939	0.139	0.107	1
Lonp1	0.080428	-0.43944	0.288	0.225	1
Mepce	0.000658	-0.43986	0.273	0.181	1
Map4k3	0.690834	-0.44002	0.1	0.09	1
Commd8	0.770926	-0.44013	0.742	0.609	1
Zbtb25	0.001503	-0.44029	0.115	0.065	1
Vamp3	0.32468	-0.44046	0.555	0.47	1
Thap2	0.13251	-0.44047	0.115	0.087	1
Cd33	0.01618	-0.44047	0.327	0.246	1
Mrto4	0.378158	-0.44108	0.367	0.305	1
Crls1	0.000285	-0.44131	0.242	0.153	1
Snap23	0.451623	-0.4414	0.791	0.657	1
Nudt14	0.056442	-0.4415	0.303	0.233	1
4933434E2	0.858552	-0.44151	0.361	0.335	1
1810009A1	0.259037	-0.44159	0.642	0.513	1
Sp3	0.403105	-0.44161	0.282	0.283	1
Ppfibp2	0.003433	-0.44163	0.376	0.273	1
Zfml	0.070373	-0.44167	0.403	0.31	1
Rbm43	0.001147	-0.4417	0.276	0.185	1
Apoc2	0.607789	-0.44203	0.494	0.453	1
Eif4e3	0.004708	-0.44205	0.512	0.385	1
1600010M	0.026682	-0.44207	0.455	0.354	1
Ngly1	0.094517	-0.44218	0.318	0.246	1
Dnttip1	0.001675	-0.4423	0.394	0.279	1
Psmd6	0.899509	-0.44232	0.512	0.452	1
Fam3a	2.88E-05	-0.44237	0.424	0.282	0.571729
Gm4902	0.089938	-0.44243	0.694	0.563	1
Senp7	0.000622	-0.44287	0.136	0.076	1
Adsl	0.000883	-0.44308	0.439	0.312	1
Nmral1	0.008381	-0.44312	0.4	0.301	1
Ephx1	0.197769	-0.44321	0.361	0.298	1
Prpsap1	0.161313	-0.4435	0.427	0.343	1
Mapkap1	0.018847	-0.44367	0.358	0.263	1
Rnaset2b	0.030209	-0.44383	0.8	0.732	1
Ado	0.001263	-0.44387	0.442	0.318	1
Hist1h2ae	0.007601	-0.44387	0.261	0.185	1
Tfpt	0.111828	-0.44399	0.215	0.169	1
Xab2	0.000234	-0.44407	0.276	0.178	1
Irf2bp1	0.030237	-0.44411	0.17	0.119	1
Prpf19	0.458673	-0.44412	0.512	0.421	1

Rps6ka4	0.001068	-0.44479	0.388	0.266	1
Sco2	0.001795	-0.44494	0.503	0.371	1
Serpine1	0.00031	-0.44504	0.221	0.14	1
Rab5b	0.005114	-0.44514	0.409	0.294	1
Ripk2	0.077598	-0.44617	0.179	0.137	1
Zfp664	0.699274	-0.44625	0.139	0.125	1
Cramp1l	0.000155	-0.4465	0.155	0.085	1
Zfyve27	0.048449	-0.44673	0.152	0.11	1
Casd1	0.001809	-0.44689	0.215	0.14	1
Nt5c2	0.002389	-0.44763	0.215	0.14	1
Ltn1	0.004084	-0.44776	0.194	0.127	1
Plekhm3	0.005753	-0.44787	0.321	0.228	1
Senp3	0.001793	-0.4479	0.5	0.37	1
H2-K2	0.002822	-0.44806	0.333	0.237	1
Exoc3	0.002653	-0.44832	0.445	0.325	1
Ccdc104	0.171834	-0.44838	0.176	0.14	1
Isca2	0.122691	-0.4486	0.342	0.272	1
4921524J1	0.000112	-0.44871	0.358	0.234	1
Ascc3	0.001581	-0.44876	0.297	0.207	1
Acadsb	0.023067	-0.44876	0.209	0.149	1
Fam120b	0.082758	-0.4491	0.17	0.13	1
Pten	0.677972	-0.44925	0.785	0.676	1
Rpl34-ps1	0.358641	-0.44947	0.261	0.223	1
Rab28	0.025138	-0.44976	0.458	0.344	1
Sap30bp	0.002161	-0.44988	0.291	0.204	1
Nsd1	0.598391	-0.44991	0.267	0.255	1
Pigz	0.000114	-0.44998	0.176	0.1	1
Zfp292	0.134265	-0.45006	0.3	0.236	1
Mad2l2	0.000389	-0.45047	0.418	0.288	1
Tmem63b	0.027294	-0.45051	0.258	0.189	1
Pip4k2a	0.307773	-0.45064	0.491	0.413	1
Crtc2	0.006618	-0.45102	0.236	0.165	1
Creld1	0.160691	-0.4511	0.236	0.195	1
Cox19	0.329483	-0.45124	0.461	0.378	1
Zc3h4	0.084315	-0.45126	0.188	0.143	1
Rbm34	0.0274	-0.45132	0.348	0.263	1
Lsm7	0.492273	-0.45169	0.664	0.546	1
Nudt22	0.010104	-0.45175	0.179	0.122	1
Elmo1	0.006805	-0.4518	0.409	0.3	1
Uckl1	0.272842	-0.45197	0.306	0.253	1
Pold2	0.005509	-0.45218	0.33	0.238	1
B230219D2	0.698888	-0.45244	0.521	0.451	1
Tmem159	0.100939	-0.4528	0.279	0.218	1

Sgpp1	0.01365	-0.45306	0.645	0.484	1
Vnn3	4.40E-06	-0.45314	0.318	0.191	0.087343
Ppp1r9b	0.109631	-0.45317	0.648	0.55	1
Actr8	0.072332	-0.45333	0.252	0.193	1
Mesdc2	0.066957	-0.45352	0.467	0.359	1
Fcf1	0.088099	-0.45367	0.539	0.421	1
Dnajb13	0.000233	-0.45372	0.491	0.355	1
Agpat5	0.033859	-0.45394	0.636	0.5	1
Sart1	0.032076	-0.45399	0.391	0.295	1
Exoc1	0.111218	-0.45406	0.197	0.155	1
Utp23	0.000872	-0.45433	0.294	0.198	1
Khk	0.430901	-0.45448	0.758	0.66	1
Atg12	0.374497	-0.45458	0.348	0.297	1
Fbxo7	0.007735	-0.45461	0.264	0.186	1
Tbc1d9b	0.005578	-0.45499	0.382	0.274	1
Gcsh	0.001928	-0.45501	0.491	0.346	1
BC006779	0.006016	-0.45506	0.718	0.576	1
Nat6	0.017798	-0.45524	0.197	0.14	1
Dock2	0.012028	-0.45533	0.555	0.417	1
Fbrsl1	0.524352	-0.45539	0.121	0.107	1
Lias	0.035984	-0.45572	0.179	0.128	1
Gtf2a1	0.139188	-0.45665	0.358	0.288	1
Vgll4	0.093636	-0.45703	0.297	0.231	1
Stt3a	0.117193	-0.45714	0.561	0.455	1
Trim8	0.114986	-0.45715	0.636	0.499	1
Pdcd2	0.013674	-0.45731	0.324	0.239	1
A730098P1	0.067236	-0.45752	0.53	0.409	1
Trpc4ap	0.282734	-0.4576	0.591	0.492	1
Prim1	0.315961	-0.45807	0.13	0.105	1
Trim26	0.014362	-0.45823	0.424	0.317	1
Cab39l	0.020421	-0.45856	0.267	0.194	1
Peg13	0.091038	-0.45869	0.121	0.089	1
D1Bwg021	0.00175	-0.45872	0.273	0.187	1
Ttc28	0.54078	-0.45878	0.155	0.135	1
Wdr89	0.800257	-0.4589	0.567	0.489	1
Ubap2	0.026394	-0.45894	0.215	0.155	1
Cog4	0.002399	-0.45897	0.324	0.229	1
Fam116a	0.001757	-0.45912	0.345	0.242	1
Rbm41	0.093344	-0.45936	0.112	0.082	1
Klc4	0.000221	-0.45942	0.488	0.343	1
Mcee	0.644575	-0.45955	0.682	0.558	1
Gm20199	0.006566	-0.45957	0.124	0.077	1
Trim36	2.24E-05	-0.46016	0.152	0.08	0.443986

Cln8	0.005326	-0.46017	0.827	0.666	1
Zdhhc6	0.00017	-0.46059	0.43	0.291	1
1110037F(	0.001562	-0.4607	0.258	0.172	1
Edem2	0.620025	-0.46078	0.57	0.473	1
Angel2	0.028921	-0.46103	0.321	0.241	1
6330578E1	0.041218	-0.46116	0.715	0.574	1
R3hdm1	4.78E-05	-0.46125	0.324	0.207	0.95015
Ppp2r5c	0.184613	-0.46137	0.53	0.431	1
Ccdc58	0.323251	-0.4615	0.197	0.167	1
D2Ertd750	0.001019	-0.46174	0.142	0.083	1
Idh1	0.323798	-0.46191	0.736	0.639	1
Kif22	0.023881	-0.46193	0.106	0.068	1
Maf	0.278089	-0.46277	0.927	0.834	1
Il3ra	0.204591	-0.46279	0.1	0.077	1
Hnrnpl	0.486757	-0.46287	0.482	0.406	1
Eif2ak2	0.000251	-0.46292	0.473	0.333	1
2410002O	0.722495	-0.46301	0.206	0.186	1
Cdc23	0.109568	-0.4632	0.103	0.075	1
Ppp3r1	0.032717	-0.46337	0.536	0.408	1
Zfhx3	0.004057	-0.46346	0.642	0.479	1
Dido1	0.038975	-0.46349	0.209	0.153	1
Nfatc3	9.37E-05	-0.46361	0.303	0.191	1
Mrap	0.000156	-0.46367	0.418	0.284	1
Nom1	0.143107	-0.46384	0.194	0.153	1
2810013P(	0.066834	-0.46419	0.412	0.324	1
2610002M	0.105976	-0.46475	0.352	0.285	1
Mfap3	2.87E-05	-0.46533	0.324	0.203	0.570849
Med16	0.239532	-0.4654	0.161	0.13	1
Sigmar1	0.026231	-0.46551	0.409	0.315	1
Bola2	0.140976	-0.46599	0.836	0.783	1
Pef1	0.115219	-0.46626	0.267	0.211	1
Nsfl1c	0.438422	-0.46628	0.479	0.41	1
Uimc1	0.088839	-0.46628	0.303	0.234	1
Ythdf1	0.063304	-0.46631	0.345	0.271	1
Gpank1	0.009075	-0.46637	0.236	0.161	1
Pmpcb	0.708783	-0.46638	0.361	0.314	1
Vps41	0.012382	-0.46655	0.455	0.344	1
Adnp	0.134416	-0.46659	0.261	0.206	1
Commd9	0.009677	-0.46678	0.376	0.277	1
Dnajc14	0.009457	-0.46682	0.397	0.283	1
Chchd7	0.009129	-0.46692	0.406	0.302	1
Mybbp1a	0.028486	-0.46728	0.421	0.324	1
Rfng	0.014164	-0.46757	0.179	0.127	1

Lrp4	0.00374	-0.46757	0.23	0.155	1
Stat2	0.005001	-0.46795	0.497	0.38	1
Abl1	0.036674	-0.46796	0.373	0.285	1
Dr1	0.535022	-0.46815	0.352	0.306	1
Mrpl37	0.001032	-0.46823	0.367	0.255	1
Rpl7l1	0.028357	-0.46844	0.582	0.444	1
Slc22a18	0.03118	-0.46884	0.245	0.183	1
Cdc16	0.051026	-0.469	0.291	0.223	1
Ythdf2	0.182902	-0.46968	0.406	0.323	1
AI314976	0.341929	-0.46981	0.312	0.261	1
Gcn1l1	0.007111	-0.47004	0.221	0.149	1
Pxmp3	0.024679	-0.47009	0.47	0.369	1
Nceh1	0.008973	-0.4703	0.67	0.528	1
Cpne1	0.007865	-0.47034	0.191	0.127	1
1110012L1	0.002414	-0.47068	0.382	0.282	1
Naa30	0.009868	-0.47082	0.179	0.121	1
Gmip	0.00285	-0.4709	0.476	0.351	1
Slc25a24	0.953722	-0.47092	0.124	0.12	1
Akr1e1	0.028385	-0.47102	0.385	0.291	1
Fam193a	0.009808	-0.47146	0.197	0.137	1
Arhgef5	2.68E-05	-0.47175	0.148	0.076	0.532875
Tfg	0.696744	-0.47181	0.527	0.444	1
Utp3	0.585755	-0.47189	0.588	0.495	1
Zfp277	0.00039	-0.47206	0.342	0.227	1
Sult1a1	0.328087	-0.47218	0.367	0.31	1
Creb3	0.283271	-0.47222	0.503	0.413	1
Spcs3	0.112311	-0.47264	0.524	0.421	1
Rnf26	0.013336	-0.47287	0.23	0.164	1
Fnbp1	0.014063	-0.47297	0.461	0.356	1
Msl3	0.101233	-0.47297	0.336	0.267	1
Mrpl22	0.013368	-0.47314	0.412	0.304	1
Toe1	0.13157	-0.47318	0.103	0.077	1
Kcna2	0.507407	-0.47346	0.142	0.151	1
Fam82a2	0.000255	-0.47356	0.294	0.191	1
Parp6	0.020253	-0.47369	0.139	0.095	1
Secisbp2l	0.013615	-0.47374	0.473	0.351	1
Mgl2	0.009071	-0.47378	0.124	0.079	1
Ubr7	0.00015	-0.4738	0.279	0.175	1
Mrpl21	0.033893	-0.47385	0.573	0.434	1
Polr2i	0.40655	-0.47397	0.661	0.521	1
Clasrp	0.448354	-0.47405	0.115	0.099	1
Gcc2	0.028212	-0.47405	0.285	0.211	1
Usp24	0.005692	-0.47442	0.242	0.166	1

Pola1	0.001089	-0.47444	0.273	0.184	1
Hp	1.08E-05	-0.47467	0.391	0.454	0.214119
Idi1	0.154548	-0.47476	0.1	0.074	1
Rnft1	0.356638	-0.47492	0.33	0.28	1
2310044G	0.035574	-0.47493	0.145	0.102	1
Fbxo33	5.63E-05	-0.475	0.37	0.235	1
Mki67ip	0.121505	-0.47525	0.342	0.269	1
Prpf8	0.039363	-0.47533	0.406	0.31	1
Gfer	0.263062	-0.47568	0.561	0.459	1
Mrc1	0.379999	-0.47583	0.955	0.856	1
Npm3-ps1	0.191924	-0.4759	0.133	0.105	1
Zc3h6	0.000231	-0.47594	0.112	0.056	1
Pop5	0.273625	-0.47601	0.418	0.336	1
Pex19	0.562954	-0.47644	0.215	0.192	1
Dpp3	0.13693	-0.47648	0.391	0.321	1
Thumpd3	0.115312	-0.4769	0.43	0.347	1
AK010878	0.018777	-0.47694	0.294	0.214	1
2210021J2	0.031243	-0.47702	0.312	0.241	1
Igsf8	0.154302	-0.47719	0.727	0.61	1
Ppih	0.055705	-0.47734	0.23	0.174	1
Dalrd3	0.00998	-0.47785	0.252	0.174	1
5730508B(	0.248829	-0.47791	0.297	0.248	1
Cog2	0.008562	-0.47798	0.361	0.269	1
Il6ra	0.806268	-0.47845	0.33	0.297	1
2410016O	0.002983	-0.47873	0.164	0.105	1
Cherp	0.036704	-0.47876	0.282	0.211	1
Stradb	0.037822	-0.47882	0.139	0.098	1
Srgap2	1.70E-05	-0.47885	0.6	0.42	0.337063
Sdccag3	0.010641	-0.47894	0.406	0.29	1
Snx8	0.000115	-0.47919	0.615	0.444	1
AF251705	0.070327	-0.47929	0.955	0.927	1
Gatad2b	0.048927	-0.47937	0.185	0.134	1
Nup85	0.278687	-0.47948	0.194	0.16	1
Ddx60	0.065923	-0.4797	0.409	0.323	1
Trp53i11	0.008315	-0.47976	0.133	0.086	1
Stat5b	0.019626	-0.47976	0.282	0.208	1
Agpat6	0.003176	-0.47993	0.412	0.299	1
Rpf2	0.015913	-0.47995	0.442	0.33	1
Sgpl1	0.092645	-0.48004	0.779	0.655	1
Ppm1k	7.96E-05	-0.48044	0.288	0.185	1
Cap1	0.585625	-0.48067	0.4	0.352	1
Vopp1	0.000157	-0.4811	0.476	0.335	1
Pros1	0.280821	-0.48136	0.112	0.127	1

Scnm1	0.011151	-0.48165	0.276	0.195	1
Mtmr3	0.002524	-0.4817	0.336	0.234	1
Rhobtb1	0.007442	-0.48222	0.288	0.206	1
Atp9b	0.03291	-0.48226	0.285	0.215	1
Btk	0.000341	-0.48235	0.6	0.43	1
Tmx3	0.130222	-0.4824	0.388	0.314	1
Rb1	0.032857	-0.48252	0.242	0.175	1
Pdxdc1	0.005174	-0.4828	0.4	0.293	1
Nkiras2	0.063942	-0.48316	0.479	0.371	1
Lgals2	0.003261	-0.48327	0.127	0.075	1
Gspt1	0.870947	-0.48332	0.585	0.491	1
Wdr91	2.96E-05	-0.48358	0.309	0.195	0.586959
Zfand1	0.124754	-0.48367	0.1	0.073	1
Pou2f1	1.33E-05	-0.48383	0.148	0.073	0.264237
Tmub2	0.08236	-0.48422	0.306	0.244	1
Sh3pxd2a	0.01134	-0.48462	0.152	0.1	1
Tmem126a	0.097598	-0.48468	0.567	0.456	1
Gclm	0.349135	-0.48508	0.3	0.25	1
Fam173b	1.18E-05	-0.48526	0.527	0.357	0.234686
Cdk17	0.00042	-0.48559	0.312	0.203	1
Ypel5	0.078601	-0.48617	0.555	0.439	1
Tradd	0.000459	-0.4862	0.497	0.348	1
Mbtd1	0.000326	-0.48622	0.309	0.201	1
Gmfb	0.119086	-0.48635	0.548	0.45	1
Agtrap	0.026984	-0.48646	0.539	0.418	1
Itfg1	0.40512	-0.48652	0.467	0.386	1
Gmps	0.3698	-0.48656	0.309	0.263	1
Abt1	0.337235	-0.48703	0.17	0.143	1
Fam168b	0.007208	-0.4873	0.694	0.539	1
Afg3l2	0.000229	-0.48749	0.397	0.263	1
Xaf1	0.420363	-0.48753	0.636	0.541	1
Tbc1d2b	1.69E-05	-0.48757	0.503	0.343	0.33611
Gmppb	0.929627	-0.48777	0.176	0.17	1
Vdr	0.000753	-0.48777	0.139	0.08	1
Cd2bp2	0.00279	-0.4885	0.318	0.219	1
Rpe	0.068539	-0.48861	0.255	0.194	1
Itgal	0.37818	-0.48929	0.912	0.822	1
Gnptg	0.000176	-0.4894	0.455	0.315	1
Med4	0.008636	-0.48945	0.252	0.174	1
Rab32	0.837015	-0.48956	0.921	0.857	1
Efemp2	0.325138	-0.48957	0.209	0.172	1
2010110P(	7.53E-05	-0.48991	0.467	0.317	1
Vill	0.000441	-0.4902	0.694	0.526	1

Med9	0.000998	-0.49039	0.452	0.325	1
Mrps5	0.536765	-0.4906	0.33	0.277	1
Yif1a	0.076414	-0.49068	0.473	0.363	1
Shkbp1	0.015381	-0.49073	0.436	0.337	1
Pdgfrl	0.000911	-0.49073	0.318	0.227	1
Syt11	0.03187	-0.49079	0.13	0.089	1
Dctn6	0.838574	-0.49166	0.424	0.378	1
Mrpl55	0.019536	-0.49174	0.624	0.472	1
Tpp1	0.256687	-0.49199	0.821	0.699	1
Cisd2	0.231691	-0.49208	0.824	0.719	1
Urgcp	0.004451	-0.49258	0.248	0.173	1
Sipa1l1	1.11E-05	-0.4933	0.494	0.344	0.220694
Chtf8	0.145265	-0.4933	0.485	0.385	1
Pla2g7	0.691487	-0.49333	0.47	0.42	1
Tmem55a	0.02234	-0.49364	0.409	0.315	1
Klhl22	0.000103	-0.49415	0.164	0.089	1
2410017P(	0.069871	-0.49432	0.564	0.454	1
Stk38l	0.065561	-0.49438	0.127	0.091	1
Flywch1	0.015717	-0.49471	0.176	0.122	1
Fam58b	1.59E-05	-0.49472	0.397	0.251	0.315667
Dcxr	0.42562	-0.49481	0.712	0.596	1
Tubg1	0.031986	-0.49516	0.227	0.166	1
Hsph1	0.018449	-0.49533	0.473	0.354	1
Cul5	0.142212	-0.49538	0.279	0.227	1
Nol6	0.267263	-0.49554	0.145	0.118	1
Ptdss1	0.007541	-0.49555	0.397	0.298	1
Tanc1	0.066122	-0.49559	0.112	0.079	1
A830080D	0.150866	-0.49596	0.106	0.08	1
Npepl1	0.013204	-0.49597	0.655	0.499	1
Batf	0.010008	-0.49641	0.355	0.266	1
Mef2a	0.001453	-0.49642	0.473	0.335	1
Nr2f6	0.052832	-0.49674	0.564	0.434	1
C330006A:	0.242313	-0.49689	0.418	0.349	1
Tnpo2	0.000335	-0.49722	0.291	0.196	1
Tmem37	0.737503	-0.49739	0.882	0.798	1
Alkbh6	0.013131	-0.49743	0.279	0.201	1
Fam158a	2.39E-05	-0.49765	0.227	0.128	0.475273
Exosc6	0.113945	-0.49774	0.442	0.351	1
Psmd9	0.530005	-0.49814	0.364	0.311	1
Nop14	0.005109	-0.49847	0.345	0.247	1
Erbb2ip	0.015917	-0.49848	0.515	0.385	1
Paip1	0.009193	-0.49878	0.345	0.257	1
Acads	0.208484	-0.49896	0.503	0.399	1

Cpeb2	0.036295	-0.49904	0.218	0.161	1
Ganab	0.009728	-0.49911	0.445	0.328	1
Sec23b	0.061314	-0.49954	0.279	0.216	1
Klf11	0.000108	-0.49958	0.182	0.103	1
Kat2b	1.22E-05	-0.5001	0.406	0.26	0.243046
Foxo4	0.026892	-0.50066	0.121	0.08	1
Pmpca	0.031001	-0.50084	0.273	0.202	1
Fam98b	0.018134	-0.50109	0.276	0.206	1
Atp7a	0.000109	-0.5013	0.579	0.41	1
Eps15	0.009472	-0.50131	0.467	0.347	1
Ncapd2	4.24E-06	-0.50167	0.173	0.087	0.084157
Trap1	0.042774	-0.50192	0.339	0.257	1
5031425F1	0.003836	-0.50199	0.164	0.105	1
Zfp385a	0.005193	-0.50245	0.606	0.458	1
Ndnl2	0.290083	-0.50263	0.361	0.296	1
Mtf2	0.092572	-0.50296	0.227	0.177	1
Slc4a7	0.002713	-0.50349	0.273	0.187	1
Fam193b	0.284798	-0.50354	0.1	0.079	1
Arhgap39	0.020374	-0.50367	0.191	0.136	1
1110058L1	0.038889	-0.50393	0.652	0.51	1
Cgrrf1	0.00449	-0.50397	0.433	0.317	1
Bambi-ps1	0.143543	-0.50417	0.185	0.147	1
N6amt2	0.005881	-0.50429	0.427	0.315	1
Sertad3	0.122801	-0.50429	0.164	0.123	1
Zfp414	0.000175	-0.50434	0.348	0.233	1
Stau1	0.057937	-0.50458	0.303	0.228	1
Nfs1	0.125282	-0.50461	0.291	0.233	1
Sumf1	0.054009	-0.50467	0.539	0.429	1
Tnfsf9	0.00032	-0.50481	0.3	0.201	1
1110001A1	0.122136	-0.50541	0.673	0.535	1
D19Bwg13	0.607993	-0.50568	0.315	0.272	1
Panx1	0.02852	-0.50591	0.152	0.105	1
Herpud2	0.680745	-0.50597	0.37	0.339	1
1600012H0	0.791329	-0.50634	0.261	0.237	1
Tspyl1	0.005404	-0.50689	0.455	0.326	1
3200002M	0.00011	-0.50704	0.679	0.503	1
Prex1	0.00061	-0.50713	0.573	0.414	1
Tmem164	0.000254	-0.50766	0.536	0.39	1
Dirc2	0.000768	-0.5078	0.339	0.232	1
Tlk1	5.06E-05	-0.50781	0.442	0.293	1
Pfdn4	0.919302	-0.50804	0.403	0.352	1
Evi2b	0.140125	-0.50823	0.555	0.436	1
Soat1	0.193745	-0.50859	0.748	0.638	1

Plaa	0.052459	-0.5087	0.442	0.338	1
Zdhhc7	5.07E-05	-0.50891	0.461	0.306	1
Gm16039	0.000454	-0.50902	0.248	0.159	1
2810004N1	0.000725	-0.50908	0.497	0.344	1
Trappc10	0.003597	-0.50915	0.282	0.192	1
Mcm2	0.017806	-0.5093	0.339	0.256	1
Dcp2	0.000115	-0.50938	0.648	0.467	1
Rmnd5a	0.005643	-0.50941	0.53	0.4	1
Golga2	2.76E-06	-0.50959	0.242	0.134	0.054716
Eif2b1	0.010359	-0.50985	0.273	0.193	1
Parp10	1.83E-05	-0.51045	0.385	0.246	0.364374
Aph1c	2.68E-06	-0.51066	0.479	0.319	0.053185
Pja2	0.007082	-0.51104	0.342	0.246	1
Brf1	0.00398	-0.51109	0.203	0.137	1
Tspan4	0.762179	-0.51126	0.815	0.688	1
Snip1	0.01878	-0.51127	0.17	0.116	1
Atp13a3	0.211648	-0.51149	0.367	0.304	1
Rdh10	0.011848	-0.51208	0.133	0.086	1
Tex264	0.181395	-0.51233	0.664	0.541	1
Arhgap10	9.42E-06	-0.51234	0.43	0.273	0.187117
Gpatch8	0.087059	-0.51264	0.273	0.21	1
B4galt1	0.030594	-0.51315	0.512	0.392	1
Ppp1r37	0.001269	-0.51321	0.176	0.107	1
Zkscan14	0.017794	-0.51323	0.136	0.091	1
Trmt1	0.758421	-0.51336	0.306	0.273	1
Engase	0.010875	-0.51355	0.512	0.383	1
Sec24c	0.044722	-0.51358	0.376	0.3	1
Alkbh7	0.002686	-0.5136	0.197	0.129	1
Klhl9	0.861368	-0.51383	0.448	0.387	1
Ubxn7	0.007229	-0.51399	0.121	0.075	1
Itch	0.287127	-0.51413	0.403	0.322	1
B3gnt1	0.784724	-0.51429	0.148	0.138	1
Rit1	0.097074	-0.51484	0.645	0.495	1
Oxa1l	0.106756	-0.51498	0.445	0.348	1
Mapk14	0.671026	-0.51575	0.645	0.544	1
4930526I1	0.237053	-0.51599	0.233	0.193	1
Fahd1	0.032662	-0.51615	0.136	0.094	1
Secisbp2	0.247175	-0.51627	0.155	0.126	1
Cyp4f13	0.003282	-0.51643	0.194	0.126	1
4933439F1	0.040427	-0.51665	0.194	0.14	1
Lrrc42	0.020161	-0.51691	0.209	0.15	1
Colec12	0.00248	-0.51696	0.291	0.202	1
Rasa1	0.093295	-0.51696	0.482	0.388	1

Mkks	0.04346	-0.51722	0.464	0.359	1
D930014E1	0.000989	-0.51753	0.415	0.289	1
Ptk2b	0.047277	-0.51764	0.639	0.483	1
Polr3e	0.010029	-0.51793	0.185	0.125	1
Apex1	0.367186	-0.51824	0.642	0.543	1
Srp68	0.219724	-0.51881	0.336	0.272	1
Adck2	7.86E-06	-0.51952	0.279	0.167	0.156184
Nxt1	0.216872	-0.51967	0.476	0.379	1
Tmppe	0.001156	-0.51975	0.185	0.118	1
Lztr1	0.105863	-0.51998	0.145	0.11	1
Prkar2a	0.018669	-0.52032	0.258	0.192	1
Abhd4	0.048866	-0.52033	0.27	0.209	1
Hddc2	0.000358	-0.52057	0.273	0.175	1
Haghl	0.103032	-0.52122	0.303	0.241	1
Dpp8	0.006833	-0.52169	0.227	0.158	1
Exosc2	1.49E-05	-0.52236	0.27	0.162	0.295267
Lbp	0.54348	-0.52248	0.115	0.1	1
Cyp4f16	6.89E-05	-0.52273	0.406	0.274	1
Mrps11	0.027235	-0.52273	0.403	0.305	1
Kidins220	0.001825	-0.52276	0.345	0.24	1
5730494M	0.001198	-0.52297	0.345	0.235	1
Cyp2e1	0.801394	-0.52344	0.115	0.105	1
Cnnm4	0.0637	-0.52361	0.115	0.081	1
Tor3a	0.725355	-0.5238	0.785	0.651	1
Nipsnap3b	0.199092	-0.52389	0.521	0.417	1
Vps13a	0.004166	-0.52407	0.2	0.134	1
Itgb5	0.508169	-0.52419	0.842	0.77	1
Cttn	0.070425	-0.52422	0.327	0.249	1
Tmem188	0.013789	-0.52452	0.339	0.249	1
Slc25a16	0.136245	-0.52456	0.1	0.074	1
Pigyl	0.039471	-0.5246	0.633	0.487	1
Nudcd2	0.012025	-0.5251	0.433	0.325	1
Akap7	7.92E-05	-0.52511	0.148	0.078	1
Ccdc84	0.00278	-0.52512	0.158	0.099	1
Sphk2	0.006817	-0.5252	0.321	0.228	1
Stoml2	0.562459	-0.52569	0.512	0.43	1
Wdr3	0.020498	-0.52631	0.206	0.148	1
Pld2	0.013929	-0.5265	0.252	0.187	1
Arrdc3	0.090232	-0.52787	0.467	0.365	1
Bid	0.00067	-0.5301	0.288	0.189	1
Prps1l3	0.396404	-0.53048	0.142	0.153	1
Mbd6	0.000152	-0.53099	0.273	0.17	1
Dnlz	0.343191	-0.53149	0.452	0.365	1

Ppp2r5b	0.006864	-0.53163	0.161	0.105	1
Zfp740	0.001336	-0.53196	0.294	0.201	1
Arap2	0.00385	-0.53197	0.436	0.322	1
Zc3h7b	0.06516	-0.532	0.167	0.124	1
Arfgap1	0.761677	-0.53209	0.236	0.215	1
Ubash3b	0.000586	-0.53229	0.324	0.218	1
Cdca7	0.017334	-0.53237	0.13	0.085	1
Kcnab2	0.810489	-0.53248	0.173	0.17	1
Phkg2	0.770255	-0.53266	0.179	0.165	1
Epor	0.00388	-0.53319	0.303	0.218	1
Phlpp2	0.00209	-0.53323	0.142	0.085	1
Srprb	0.019751	-0.5336	0.415	0.31	1
Atf6	0.008328	-0.53366	0.385	0.279	1
Skiv2l2	0.000271	-0.53392	0.233	0.144	1
Tmem115	0.005695	-0.53443	0.333	0.237	1
Extl3	0.000234	-0.53461	0.376	0.249	1
Arcn1	0.080671	-0.53467	0.409	0.317	1
Gtf3c6	0.073351	-0.53484	0.415	0.327	1
Crebbp	0.047636	-0.53489	0.288	0.214	1
1700012B1	0.000132	-0.53492	0.403	0.269	1
Lnp	0.012598	-0.53509	0.142	0.093	1
Osbpl1a	0.032175	-0.53517	0.224	0.167	1
Il13ra1	0.05897	-0.53532	0.745	0.569	1
Haus4	3.41E-06	-0.53532	0.288	0.169	0.067693
Mri1	0.088908	-0.53552	0.315	0.245	1
Senp1	0.006984	-0.53552	0.261	0.182	1
Sfxn1	0.698792	-0.53556	0.327	0.282	1
Alg5	0.113844	-0.5371	0.688	0.534	1
Fam192a	0.062456	-0.53721	0.494	0.376	1
Clu	0.421856	-0.53757	0.258	0.217	1
Rrp36	0.007119	-0.53793	0.285	0.199	1
Obfc2a	0.124454	-0.53868	0.488	0.376	1
Apba3	0.030327	-0.53875	0.206	0.15	1
Dnajc11	0.047053	-0.53894	0.239	0.181	1
Atp6v1h	0.013325	-0.53917	0.536	0.4	1
Zmynd8	0.052378	-0.53939	0.464	0.366	1
Eif4ebp3	0.00051	-0.53958	0.285	0.187	1
Dicer1	0.344528	-0.53961	0.17	0.143	1
Dhx40	0.109076	-0.53999	0.297	0.237	1
Lmf1	0.044944	-0.54014	0.182	0.131	1
Lpar6	0.139632	-0.54044	0.548	0.437	1
Sharpin	0.031366	-0.54064	0.588	0.454	1
Mta1	0.00638	-0.54081	0.267	0.187	1

Rcan1	0.869894	-0.54095	0.464	0.409	1
Klrk1	0.00698	-0.54113	0.624	0.481	1
Dtd1	0.083158	-0.54148	0.258	0.203	1
Exosc4	0.016377	-0.54177	0.6	0.462	1
Cars	0.000613	-0.54208	0.273	0.177	1
Trmt2b	0.191353	-0.54244	0.173	0.141	1
Amn1	0.029001	-0.54247	0.264	0.203	1
Cryz	0.118981	-0.54278	0.121	0.089	1
Qrich1	0.017988	-0.54343	0.373	0.267	1
Wdr45	0.07796	-0.5438	0.279	0.217	1
Chka	0.002563	-0.54397	0.336	0.237	1
Pcmtd2	0.000248	-0.54412	0.155	0.086	1
D10Wsu1(	0.000422	-0.54428	0.224	0.139	1
Nacc2	9.86E-05	-0.54446	0.373	0.247	1
Spred1	0.006611	-0.54506	0.176	0.12	1
Acap2	0.011786	-0.54516	0.455	0.336	1
Stard3nl	0.774013	-0.54535	0.803	0.692	1
Lmf2	0.13403	-0.54553	0.324	0.259	1
Ston2	0.561793	-0.5456	0.179	0.159	1
Txnrd2	0.003508	-0.54579	0.376	0.266	1
Paqr7	0.005829	-0.54607	0.13	0.079	1
Anapc7	0.004034	-0.54611	0.261	0.177	1
1810008A:	0.939084	-0.54655	0.315	0.286	1
1300010F(	0.003355	-0.5466	0.115	0.067	1
Slc33a1	0.080708	-0.54684	0.148	0.108	1
Insr	0.001273	-0.54698	0.248	0.16	1
Th1l	0.095903	-0.54715	0.188	0.142	1
Cstf2	0.004475	-0.5474	0.215	0.147	1
Ccdc41	0.003725	-0.54773	0.267	0.186	1
Cpne2	0.005658	-0.54788	0.518	0.377	1
Gm6377	0.00122	-0.54792	0.739	0.579	1
Rnf19a	0.020528	-0.54798	0.167	0.116	1
Slc9a8	0.200693	-0.54835	0.124	0.099	1
Mtcp1	0.003266	-0.54843	0.412	0.298	1
Arfip2	0.004175	-0.54846	0.239	0.167	1
Rfwd2	0.01701	-0.5485	0.358	0.268	1
Cdk19	0.022684	-0.54899	0.221	0.159	1
Arfgap2	0.331605	-0.54909	0.306	0.254	1
Rpf1	0.276919	-0.54918	0.327	0.264	1
Rrp9	0.061753	-0.54937	0.245	0.184	1
Dcaf13	0.049013	-0.54974	0.427	0.335	1
Cyp27a1	0.01848	-0.54991	0.482	0.369	1
Rspry1	0.01936	-0.55044	0.294	0.22	1

Tnfsf15	0.024885	-0.55053	0.152	0.107	1
Ppapdc2	0.000463	-0.55188	0.164	0.096	1
Gmeb2	0.000624	-0.55217	0.173	0.102	1
Sssca1	0.308699	-0.55219	0.297	0.246	1
Cdk10	0.024164	-0.55236	0.239	0.172	1
Lyrm2	0.762848	-0.55246	0.297	0.263	1
Rbm4	0.035639	-0.55335	0.17	0.12	1
Zfp637	0.005071	-0.5539	0.312	0.22	1
Rnpepl1	0.180045	-0.55414	0.436	0.361	1
Ccng1	0.309664	-0.55421	0.43	0.355	1
Ccdc71	0.003461	-0.55436	0.203	0.131	1
Kti12	0.440901	-0.55475	0.161	0.136	1
Snx9	0.001954	-0.55487	0.218	0.144	1
Apool	0.000604	-0.55528	0.336	0.234	1
L3mbtl2	0.005183	-0.5554	0.155	0.099	1
Bola1	0.382568	-0.55617	0.388	0.326	1
Tmem26	0.020271	-0.55674	0.491	0.385	1
Ttpal	0.00389	-0.55677	0.136	0.083	1
Oasl2	0.000953	-0.55733	0.712	0.546	1
Pkib	0.616357	-0.55745	0.8	0.705	1
Faf2	8.50E-06	-0.55748	0.336	0.201	0.168731
Ppp6r1	0.041648	-0.5575	0.415	0.321	1
Sart3	0.002868	-0.55771	0.206	0.135	1
Eftud2	0.196046	-0.55803	0.279	0.23	1
Ccl24	0.231465	-0.55823	0.909	0.872	1
Wbscr22	0.159117	-0.55828	0.252	0.201	1
Asxl2	0.38413	-0.55865	0.37	0.314	1
Uqcc	0.711031	-0.55871	0.118	0.12	1
Nomo1	6.11E-05	-0.55887	0.258	0.155	1
Tmem185l	0.001727	-0.55918	0.321	0.217	1
Bmyc	0.036746	-0.55991	0.17	0.119	1
Ikbkg	0.011802	-0.56108	0.255	0.185	1
Ubr3	0.003763	-0.5612	0.258	0.174	1
Bcl2l13	0.009163	-0.56124	0.188	0.127	1
Decr1	0.297093	-0.56175	0.245	0.205	1
Mau2	0.042466	-0.56216	0.379	0.295	1
Tbcel	0.00075	-0.56233	0.185	0.112	1
Smyd3	0.001458	-0.56238	0.155	0.091	1
Gfpt1	0.226096	-0.56252	0.309	0.258	1
Slc12a7	0.000181	-0.56282	0.497	0.345	1
1110034G:	5.71E-05	-0.56401	0.27	0.167	1
Adm	0.183949	-0.56418	0.115	0.089	1
A630007B(	0.777066	-0.56516	0.115	0.106	1

Pex3	0.001877	-0.5653	0.239	0.158	1
Usp32	0.008095	-0.56583	0.224	0.153	1
Plod1	0.377952	-0.56624	0.812	0.692	1
Pex11b	0.050446	-0.56634	0.312	0.238	1
Ly96	0.306365	-0.56653	0.812	0.67	1
Mitd1	0.007445	-0.5666	0.424	0.315	1
Rap2c	0.081798	-0.56735	0.585	0.463	1
Csad	0.081554	-0.56737	0.209	0.156	1
D19Wsu16	0.266238	-0.56744	0.433	0.354	1
Atg9a	0.157503	-0.56759	0.176	0.14	1
Trak2	0.002124	-0.5677	0.158	0.096	1
1810013L2	0.016321	-0.56787	0.291	0.211	1
Rgp1	0.003014	-0.56801	0.13	0.078	1
Abcd4	0.003411	-0.56803	0.364	0.269	1
Slc5a3	0.247679	-0.56809	0.127	0.103	1
Slc27a4	0.005932	-0.56909	0.158	0.102	1
Tagap	0.125365	-0.56913	0.215	0.171	1
2610524H0	0.000112	-0.56975	0.361	0.24	1
Bysl	0.039877	-0.56997	0.248	0.184	1
Ttf1	0.000722	-0.56998	0.209	0.131	1
Cd59a	0.00315	-0.57002	0.224	0.149	1
Slc10a7	0.041323	-0.57033	0.155	0.112	1
Fbxl14	5.27E-05	-0.5725	0.382	0.248	1
Slc38a7	0.000111	-0.57256	0.364	0.244	1
Ebag9	0.042329	-0.57376	0.315	0.243	1
Zyg11b	0.020557	-0.57407	0.236	0.171	1
Aim1	0.236592	-0.57425	0.324	0.268	1
Ap2a1	0.152501	-0.57464	0.336	0.268	1
Yipf2	0.009767	-0.57465	0.182	0.123	1
C2cd2l	2.19E-05	-0.57514	0.236	0.137	0.43403
Tsta3	0.449959	-0.57515	0.53	0.439	1
Ubn2	0.278272	-0.57518	0.4	0.331	1
Stat6	0.244046	-0.57524	0.542	0.444	1
Tbkbp1	0.000335	-0.57537	0.379	0.258	1
Gstz1	0.491548	-0.5758	0.155	0.133	1
Fam53a	0.263227	-0.57583	0.321	0.268	1
Galnt10	0.078894	-0.57585	0.224	0.177	1
Dmxl1	0.011363	-0.5759	0.415	0.306	1
Fam160a2	0.000359	-0.57622	0.139	0.076	1
Prdm1	1.65E-05	-0.57677	0.336	0.216	0.328442
Ahctf1	0.133443	-0.57682	0.176	0.138	1
Dennd1a	0.008406	-0.57722	0.367	0.27	1
Tcirg1	0.018928	-0.57756	0.752	0.583	1

Ascc2	0.09086	-0.57775	0.158	0.12	1
Tmem131	0.006127	-0.57828	0.358	0.258	1
Dpcd	0.007357	-0.5783	0.412	0.3	1
Rras2	0.689595	-0.57922	0.203	0.18	1
Axin1	0.000269	-0.57943	0.27	0.172	1
Zfpl1	0.007097	-0.57945	0.391	0.281	1
Nup54	0.002679	-0.57965	0.258	0.172	1
2810008D(	0.001833	-0.57993	0.373	0.267	1
Pitpnc1	0.426117	-0.58021	0.582	0.491	1
Tomm40l	3.07E-05	-0.58087	0.306	0.188	0.609724
Nt5c3l	0.22134	-0.58116	0.164	0.13	1
Gstp1	0.122069	-0.58201	0.364	0.361	1
Cant1	0.012304	-0.58205	0.403	0.306	1
Ddx49	0.110008	-0.58216	0.321	0.251	1
Cyb561d2	0.071234	-0.58221	0.439	0.35	1
Herc2	0.131489	-0.58245	0.358	0.283	1
Otud7b	0.116194	-0.58245	0.136	0.103	1
Zfp524	7.13E-05	-0.5825	0.37	0.242	1
Orc3	0.000143	-0.5827	0.23	0.141	1
Farsa	0.100283	-0.583	0.339	0.266	1
Nsun2	0.033911	-0.58306	0.512	0.379	1
Zswim7	1.36E-05	-0.58325	0.23	0.132	0.269898
Macrod1	0.006612	-0.58363	0.218	0.149	1
Usp18	0.001083	-0.58412	0.615	0.453	1
Gba	0.020162	-0.58472	0.661	0.515	1
Dhx32	0.008984	-0.58495	0.191	0.129	1
Ppp3cb	0.05236	-0.58496	0.206	0.153	1
Gosr1	0.014878	-0.58518	0.258	0.184	1
Hsf1	0.091669	-0.58541	0.264	0.211	1
Larp4b	0.005266	-0.58546	0.506	0.371	1
Pik3cg	0.004838	-0.58614	0.221	0.152	1
Nin	0.285184	-0.5867	0.23	0.191	1
Cdc37l1	0.055203	-0.58677	0.23	0.175	1
Gcc1	0.098601	-0.58704	0.115	0.084	1
Zfp386	0.012784	-0.58762	0.139	0.09	1
Smek1	0.58561	-0.58804	0.252	0.22	1
Ranbp10	0.000674	-0.58852	0.206	0.127	1
Scd1	0.196883	-0.58858	0.339	0.334	1
Osbpl8	0.072423	-0.58868	0.203	0.15	1
4930506M	0.002863	-0.58943	0.63	0.499	1
Zcchc10	0.120897	-0.58953	0.167	0.129	1
Prrc1	0.004062	-0.58991	0.376	0.265	1
0610009O	0.252304	-0.59044	0.209	0.172	1

Slc25a12	0.00338	-0.59053	0.352	0.247	1
Gusb	0.282388	-0.59086	0.712	0.582	1
Athl1	0.026466	-0.59111	0.179	0.127	1
Dnmt1	0.000194	-0.59118	0.406	0.266	1
Mrps18b	0.448808	-0.59145	0.373	0.313	1
Phf10	0.149673	-0.59146	0.315	0.254	1
Morc3	0.006607	-0.59152	0.539	0.393	1
Pex5	2.42E-05	-0.59164	0.424	0.284	0.480224
Nrip1	0.002764	-0.59168	0.409	0.287	1
Wrn	0.007395	-0.59186	0.227	0.154	1
Stxbp3a	0.056916	-0.59209	0.476	0.366	1
Lgals8	0.007215	-0.5926	0.594	0.439	1
C330007P(	0.000283	-0.59314	0.436	0.299	1
Cttnbp2nl	2.50E-05	-0.59328	0.682	0.481	0.496859
Clp1	0.040987	-0.59332	0.17	0.121	1
Fmnl2	0.001164	-0.59339	0.382	0.264	1
Agl	0.036533	-0.59361	0.112	0.076	1
Ttc15	0.004229	-0.59386	0.215	0.145	1
Cdc27	0.00771	-0.59432	0.242	0.174	1
U2af1l4	0.017574	-0.59442	0.442	0.338	1
Tubgcp3	0.019136	-0.59473	0.173	0.119	1
Map3k5	0.030167	-0.59477	0.194	0.14	1
Rnf41	0.002155	-0.59477	0.333	0.235	1
1110021J0	0.046429	-0.59517	0.2	0.147	1
Stk3	0.000412	-0.59555	0.339	0.227	1
Snap47	0.03758	-0.59576	0.167	0.118	1
Chordc1	0.004015	-0.59581	0.409	0.29	1
A430005L1	0.131516	-0.59599	0.494	0.393	1
Dram2	0.095448	-0.59619	0.803	0.718	1
Mum1	0.08962	-0.59628	0.133	0.097	1
Fggy	0.016943	-0.5965	0.212	0.152	1
Hells	0.00428	-0.59663	0.152	0.097	1
Asl	0.327228	-0.59687	0.303	0.245	1
Pilra	0.002914	-0.59773	0.939	0.906	1
Ulk2	0.121812	-0.59776	0.412	0.336	1
Eef1e1	0.186829	-0.59826	0.418	0.353	1
Pex14	0.054912	-0.5985	0.233	0.176	1
Slc39a1	0.697932	-0.59851	0.603	0.521	1
Itfg3	0.004581	-0.59869	0.697	0.533	1
Ccdc127	0.064972	-0.59915	0.273	0.208	1
Mir1931	0.01627	-0.59925	0.13	0.085	1
Pex6	0.266602	-0.59968	0.2	0.166	1
Dnajc24	0.001563	-0.60007	0.342	0.238	1

Fabp7	0.007351	-0.60008	0.939	0.909	1
Btd	0.260143	-0.60009	0.127	0.103	1
Lrrc57	0.458366	-0.60021	0.133	0.116	1
Tnk2	0.000172	-0.60024	0.279	0.177	1
Wash	0.311646	-0.6003	0.394	0.332	1
Lrrc8d	0.129514	-0.60031	0.206	0.159	1
Armc10	0.299455	-0.60058	0.197	0.16	1
Dapp1	0.051464	-0.6006	0.333	0.258	1
Chd1l	0.005731	-0.60084	0.139	0.088	1
Rfesd	0.850525	-0.60136	0.1	0.094	1
2210018M	0.260342	-0.60145	0.112	0.088	1
Taf3	0.501204	-0.60151	0.239	0.207	1
1810026J2	0.041795	-0.60174	0.142	0.101	1
4930453N	0.182304	-0.60299	0.239	0.193	1
1810029B	0.008536	-0.60314	0.258	0.185	1
Chchd5	0.060464	-0.60334	0.397	0.306	1
Mipep	0.293181	-0.6034	0.127	0.103	1
Dock10	0.851653	-0.60351	0.567	0.492	1
2210408F2	0.198066	-0.60357	0.103	0.079	1
Polg	0.553155	-0.60402	0.194	0.168	1
2410091C	0.496991	-0.60405	0.118	0.102	1
Lix1	0.004676	-0.60409	0.13	0.079	1
Pccb	0.00778	-0.60415	0.155	0.101	1
Otud5	0.014949	-0.60439	0.327	0.244	1
Rpap3	0.001931	-0.60457	0.2	0.129	1
Noc2l	0.013275	-0.60459	0.464	0.345	1
D030016E	0.165606	-0.60539	0.173	0.135	1
Rnf215	0.002753	-0.6058	0.233	0.156	1
Ctnnd1	0.533579	-0.6061	0.627	0.562	1
Map3k11	4.95E-05	-0.60634	0.476	0.31	0.98327
Mtus1	0.156326	-0.60672	0.612	0.5	1
Supt16h	0.108503	-0.60683	0.391	0.318	1
Ctps	0.398518	-0.60708	0.106	0.09	1
Tor1b	5.26E-05	-0.60797	0.385	0.247	1
BC147527	0.006338	-0.60804	0.252	0.177	1
Mettl5	0.000477	-0.60883	0.297	0.194	1
Atxn1	0.047928	-0.60928	0.333	0.254	1
Nudt8	0.01214	-0.60934	0.197	0.137	1
Acsf2	0.001759	-0.60951	0.348	0.249	1
Cnot10	8.23E-06	-0.60959	0.248	0.142	0.163527
Wipf2	0.137581	-0.6102	0.221	0.174	1
Dnajc30	0.019027	-0.61056	0.282	0.204	1
Xrcc6bp1	0.0483	-0.61076	0.139	0.101	1

Cse1l	0.119946	-0.61107	0.255	0.198	1
Hspbp1	0.021309	-0.61118	0.327	0.241	1
Hmgxb4	0.016883	-0.61138	0.191	0.134	1
Pgrmc2	0.002799	-0.61165	0.309	0.211	1
Fbxo4	0.01519	-0.6119	0.467	0.362	1
Pepd	0.540676	-0.61195	0.73	0.596	1
Pdf	0.01466	-0.61217	0.445	0.333	1
Taf2	0.042498	-0.6123	0.139	0.099	1
Cbll1	0.405891	-0.61266	0.13	0.109	1
Mlx	0.00489	-0.61289	0.324	0.232	1
Vhl	0.002806	-0.61297	0.242	0.161	1
Ssfa2	0.009609	-0.61357	0.482	0.354	1
Aacs	0.001157	-0.61476	0.112	0.062	1
Pcnt	0.059681	-0.61613	0.191	0.142	1
Galk1	0.014508	-0.61624	0.345	0.252	1
Pgam5	0.007474	-0.61663	0.203	0.134	1
Oas1g	0.066772	-0.61686	0.455	0.355	1
Ocel1	8.69E-05	-0.61703	0.394	0.253	1
Galnt7	0.000581	-0.61704	0.382	0.268	1
Fam126a	0.00081	-0.61717	0.315	0.206	1
3110040N	0.005562	-0.61742	0.255	0.175	1
Hhex	0.274462	-0.61764	0.427	0.347	1
Casp1	0.43103	-0.6178	0.633	0.523	1
Aldh3a2	0.023398	-0.61824	0.282	0.208	1
Tmem189	5.21E-06	-0.61829	0.445	0.288	0.103392
Arl5a	0.397329	-0.61851	0.515	0.431	1
Raver1	0.005279	-0.61901	0.3	0.21	1
Suv420h1	0.185118	-0.61939	0.17	0.135	1
Ppp1r7	0.004455	-0.61982	0.382	0.278	1
Bfar	0.362308	-0.62008	0.421	0.356	1
Zfp260	0.007639	-0.62012	0.152	0.097	1
Pygl	0.482561	-0.62015	0.242	0.237	1
Thap7	0.129709	-0.62024	0.294	0.233	1
Uhrf1	0.028048	-0.62064	0.173	0.123	1
Slc30a7	0.01486	-0.62107	0.373	0.273	1
Mpzl1	0.060446	-0.62115	0.188	0.14	1
Cenpc1	0.002908	-0.62116	0.115	0.067	1
Pwp1	0.303522	-0.6214	0.23	0.193	1
Cdk5	0.00108	-0.62145	0.276	0.186	1
Gbp6	7.52E-05	-0.62171	0.339	0.222	1
Slc35b2	0.041236	-0.62227	0.542	0.426	1
Atn1	0.039976	-0.6228	0.233	0.174	1
Pnpla7	0.179896	-0.62347	0.318	0.258	1

Ppm1h	0.387648	-0.6236	0.491	0.414	1
Msl2	0.350772	-0.62364	0.258	0.218	1
Ublcp1	0.219723	-0.62391	0.264	0.215	1
Prkrip1	0.020191	-0.62401	0.339	0.251	1
Cdadc1	0.007422	-0.62429	0.215	0.146	1
Caml	0.037409	-0.62517	0.3	0.222	1
Grsf1	0.041131	-0.62566	0.412	0.322	1
Dhrs1	0.143916	-0.62592	0.818	0.734	1
Nck1	0.728188	-0.62656	0.436	0.372	1
Mettl1	0.44639	-0.62671	0.145	0.125	1
Pno1	0.000216	-0.62677	0.403	0.282	1
Tmem63a	0.006218	-0.62678	0.388	0.284	1
Inpp4a	6.26E-05	-0.62702	0.218	0.127	1
Sh2b1	0.212546	-0.62707	0.152	0.121	1
Wrnip1	0.921556	-0.62751	0.179	0.165	1
Pla2g15	0.00735	-0.6277	0.891	0.746	1
Zfp746	0.253513	-0.62782	0.142	0.114	1
Slc30a9	4.41E-05	-0.62816	0.348	0.219	0.875897
Pola2	0.011859	-0.62822	0.282	0.206	1
Zfp639	0.004261	-0.62833	0.312	0.219	1
Slc38a1	0.752056	-0.62855	0.164	0.15	1
Art3	0.001679	-0.62896	0.327	0.234	1
Piga	8.86E-05	-0.6292	0.1	0.046	1
Josd1	6.33E-06	-0.62958	0.373	0.231	0.125722
Bop1	0.065375	-0.62981	0.394	0.309	1
Tcf20	0.000725	-0.63093	0.273	0.181	1
Rcl1	0.598862	-0.63094	0.148	0.132	1
Cep63	0.003811	-0.63139	0.17	0.108	1
Clpb	0.100634	-0.63213	0.188	0.146	1
Azi2	0.009885	-0.63226	0.455	0.328	1
Exoc7	0.015098	-0.6324	0.321	0.238	1
Katnb1	0.071734	-0.63283	0.127	0.094	1
Scap	0.070178	-0.63291	0.261	0.201	1
Trpv4	0.001421	-0.63312	0.197	0.126	1
Mcat	0.000722	-0.63369	0.252	0.163	1
Plxnb3	0.077098	-0.6343	0.121	0.087	1
Tbxas1	0.671371	-0.63464	0.873	0.768	1
Nufip1	0.005929	-0.63465	0.385	0.284	1
C3ar1	0.002781	-0.63483	0.882	0.724	1
Kif1b	0.5144	-0.6349	0.318	0.27	1
Chd8	0.007322	-0.63518	0.315	0.225	1
Golga5	0.009673	-0.63543	0.306	0.22	1
Ube2z	0.076292	-0.63559	0.455	0.351	1

Mlycd	0.032869	-0.63584	0.367	0.279	1
Acer3	0.124631	-0.63593	0.676	0.559	1
1110038D1	0.005533	-0.63694	0.242	0.162	1
Sept8	0.003433	-0.63695	0.4	0.285	1
1190007I0	0.04076	-0.63699	0.367	0.282	1
Acot8	0.000379	-0.63727	0.418	0.289	1
Mfsd11	0.000121	-0.6374	0.627	0.439	1
Gm10336	0.000357	-0.63749	0.173	0.1	1
BC035044	0.274356	-0.63756	0.173	0.141	1
Tspan17	0.041849	-0.63776	0.382	0.296	1
Lrrc45	0.051766	-0.63786	0.142	0.102	1
Pdgfa	0.001813	-0.63805	0.236	0.157	1
Atxn7l1	0.146537	-0.63851	0.221	0.175	1
Gm12250	0.00254	-0.63851	0.439	0.317	1
Abi3	0.143399	-0.63865	0.736	0.646	1
Dpep2	0.000214	-0.63869	0.288	0.188	1
Prmt5	0.128225	-0.63874	0.185	0.141	1
Wdr74	0.373108	-0.63913	0.303	0.252	1
Nrf1	0.029636	-0.63917	0.212	0.155	1
Gigyf2	0.00925	-0.63924	0.191	0.131	1
H2-T10	0.051199	-0.63969	0.176	0.129	1
N6amt1	0.132538	-0.63972	0.218	0.17	1
Fbxl17	0.000833	-0.63989	0.276	0.182	1
Slc1a3	1.48E-05	-0.63994	0.573	0.396	0.293665
Plekhm1	0.009526	-0.64034	0.376	0.281	1
Clcc1	0.005657	-0.64053	0.412	0.296	1
Wdr53	0.051249	-0.64149	0.142	0.103	1
Fam40a	0.020813	-0.64159	0.185	0.131	1
St7l	0.076986	-0.64196	0.239	0.185	1
Tubgcp2	7.58E-05	-0.64233	0.2	0.119	1
Endog	0.098985	-0.64234	0.248	0.192	1
Asb8	0.020304	-0.64275	0.282	0.204	1
Mre11a	0.000512	-0.64319	0.112	0.059	1
Srfbp1	0.060115	-0.64321	0.167	0.124	1
Cpsf3	0.087845	-0.64341	0.355	0.282	1
Emr1	0.055233	-0.6436	0.976	0.941	1
Pank1	0.017025	-0.64371	0.191	0.133	1
Gss	0.150806	-0.64416	0.164	0.128	1
Ndufaf4	0.137209	-0.64439	0.339	0.27	1
Kptn	0.06914	-0.64454	0.106	0.074	1
Pcyox1	0.057025	-0.64456	0.642	0.495	1
Sik3	0.057693	-0.64488	0.109	0.076	1
Rif1	0.001747	-0.64531	0.173	0.107	1

Cno	0.097511	-0.64572	0.394	0.314	1
Metrn	0.006096	-0.64599	0.285	0.2	1
Srrd	2.93E-05	-0.64627	0.448	0.297	0.581253
Cby1	0.000763	-0.64634	0.264	0.17	1
Mrps31	0.324506	-0.64642	0.176	0.147	1
Lass4	0.165068	-0.64654	0.191	0.152	1
Kdm2b	0.016979	-0.64699	0.285	0.206	1
Aggf1	0.033228	-0.64756	0.27	0.201	1
Tmub1	0.207382	-0.64821	0.233	0.19	1
E430025E2	2.51E-05	-0.64847	0.497	0.343	0.498869
Ptgs1	0.001	-0.65031	0.961	0.888	1
Rin3	0.207695	-0.65085	0.167	0.132	1
Xpo1	0.202203	-0.65086	0.37	0.294	1
Atp8a1	0.039829	-0.65121	0.576	0.445	1
Rgmb	0.278384	-0.65155	0.2	0.165	1
Mterfd1	0.052721	-0.65185	0.215	0.159	1
Ahsa2	0.908587	-0.65208	0.227	0.212	1
Atpaf2	0.000532	-0.65222	0.227	0.141	1
Dnajc25	0.044543	-0.65224	0.236	0.175	1
Uso1	0.382363	-0.65239	0.297	0.247	1
Tmem129	0.296331	-0.65256	0.115	0.095	1
Ppp2r3c	0.183864	-0.6526	0.23	0.188	1
Adamdec1	0.826943	-0.65283	0.152	0.14	1
Ubqln2	0.959043	-0.65303	0.276	0.248	1
Haus5	0.009537	-0.65305	0.121	0.076	1
Sdf2l1	0.019171	-0.65309	0.794	0.758	1
Gpn2	0.021756	-0.6539	0.167	0.115	1
Zdhhc16	0.000137	-0.65392	0.315	0.198	1
Rap2a	0.296088	-0.65518	0.145	0.12	1
Rabif	0.036074	-0.65523	0.2	0.144	1
Mut	0.220332	-0.65537	0.164	0.131	1
Smn1	0.094135	-0.65571	0.294	0.23	1
Ncbp2	0.001062	-0.65761	0.482	0.326	1
Dbr1	0.156355	-0.65809	0.148	0.115	1
Dak	0.847467	-0.65881	0.112	0.103	1
Tmem223	0.815958	-0.65883	0.203	0.18	1
4930572J0	0.002441	-0.65921	0.361	0.25	1
Btbd2	0.049298	-0.65967	0.255	0.191	1
Slc12a6	0.979128	-0.65985	0.273	0.251	1
Dhx8	0.031402	-0.65998	0.179	0.13	1
Lcmt1	0.140635	-0.66004	0.352	0.282	1
Ankrd40	0.00578	-0.66011	0.415	0.305	1
Ylpm1	5.86E-05	-0.66013	0.221	0.128	1

Tyk2	0.02304	-0.6605	0.27	0.2	1
1300002E1	0.002362	-0.6605	0.248	0.164	1
Ppp4r1	0.001198	-0.66057	0.327	0.222	1
Zzef1	0.185724	-0.66059	0.255	0.205	1
Neurl1a	0.013416	-0.66068	0.109	0.069	1
Itpripl1	0.659955	-0.66115	0.197	0.175	1
Sgk3	0.216226	-0.66117	0.491	0.404	1
Tmem110	0.002284	-0.66177	0.255	0.169	1
Dcun1d3	0.005439	-0.66193	0.106	0.062	1
Wdr82	0.089301	-0.66208	0.245	0.189	1
Pogz	0.011307	-0.6621	0.124	0.079	1
Dusp12	0.072544	-0.66217	0.248	0.191	1
Specc1l	0.016338	-0.66222	0.321	0.236	1
Sema4c	0.000505	-0.66233	0.379	0.258	1
3830406C1	0.626897	-0.66251	0.479	0.405	1
Galc	0.0225	-0.66285	0.23	0.17	1
Psme4	0.058963	-0.66298	0.367	0.281	1
Ddit4	0.312834	-0.66359	0.212	0.172	1
Arhgap21	0.000106	-0.6637	0.279	0.174	1
Expi	0.002559	-0.66378	0.676	0.521	1
Trmt61b	0.000314	-0.6638	0.224	0.138	1
Ipo11	0.6015	-0.66411	0.103	0.092	1
Dnajc4	0.055065	-0.66416	0.327	0.244	1
Ap4s1	0.501251	-0.66442	0.406	0.341	1
Myst1	0.005335	-0.66459	0.233	0.16	1
Man2b2	0.005189	-0.66482	0.476	0.355	1
Rars2	0.007397	-0.66487	0.161	0.105	1
Plagl2	0.006892	-0.66655	0.336	0.243	1
Agap3	3.18E-05	-0.66718	0.361	0.235	0.631715
Coq6	0.000349	-0.66722	0.148	0.082	1
Nup88	0.052892	-0.66749	0.276	0.21	1
Cln3	0.001454	-0.66752	0.497	0.361	1
Papd4	0.205208	-0.66764	0.612	0.495	1
Shmt1	0.00241	-0.66779	0.106	0.059	1
Folr2	0.000369	-0.66795	0.985	0.976	1
Fam53c	9.60E-05	-0.668	0.267	0.162	1
Tle1	0.013253	-0.66801	0.155	0.103	1
Dpp9	0.003484	-0.66839	0.352	0.25	1
Sec24b	0.000981	-0.66841	0.291	0.194	1
Dom3z	0.190826	-0.66902	0.221	0.179	1
Anapc2	0.149518	-0.66998	0.321	0.259	1
Mtmr12	0.000155	-0.67118	0.176	0.099	1
Paox	0.003923	-0.67134	0.597	0.432	1

Atf6b	0.867062	-0.67186	0.397	0.349	1
Foxj2	0.000105	-0.67189	0.324	0.207	1
Bcl2a1a	0.000161	-0.6719	0.8	0.63	1
Map3k7	0.009837	-0.67195	0.176	0.119	1
Pard6a	0.037097	-0.67206	0.212	0.155	1
Nudt5	0.055115	-0.67229	0.415	0.319	1
Tbc1d5	0.001623	-0.67276	0.355	0.253	1
Gas2l1	0.0973	-0.67342	0.306	0.241	1
Igf1	0.000112	-0.67362	0.955	0.923	1
Pgm1	0.038297	-0.67412	0.188	0.138	1
Ppp2r1b	0.255779	-0.67437	0.139	0.114	1
Whsc1	0.000847	-0.67441	0.33	0.225	1
Lrpprc	0.004641	-0.67455	0.203	0.134	1
Rab3il1	0.004443	-0.67455	0.564	0.42	1
Tsr1	0.344549	-0.67494	0.139	0.117	1
Tmem229l	0.022818	-0.67505	0.258	0.185	1
Bcor	0.008363	-0.67561	0.182	0.123	1
Eri1	0.005402	-0.67563	0.461	0.327	1
Arsk	0.107027	-0.67615	0.148	0.114	1
Znfx1	0.099428	-0.67665	0.679	0.561	1
Msr1	0.663176	-0.67678	0.939	0.912	1
Rpp30	0.062687	-0.67685	0.252	0.19	1
Adi1	0.38643	-0.67721	0.2	0.173	1
Apol9b	0.000369	-0.67722	0.312	0.209	1
Trpv2	0.005104	-0.67793	0.488	0.367	1
Zmym2	0.001067	-0.67814	0.182	0.111	1
Timm9	0.095086	-0.67846	0.261	0.203	1
Hlx	0.001408	-0.67851	0.582	0.437	1
B230378P2	0.000476	-0.67881	0.697	0.512	1
Kifap3	0.001145	-0.67884	0.248	0.161	1
Meaf6	0.02723	-0.67939	0.324	0.242	1
Upf1	0.006657	-0.67999	0.412	0.299	1
Sptlc1	0.003478	-0.68046	0.373	0.269	1
Tmem41a	0.049728	-0.68119	0.221	0.162	1
Gm12216	0.06043	-0.68122	0.142	0.102	1
Pex10	3.06E-05	-0.68133	0.148	0.077	0.607123
1200009I0	0.000209	-0.68262	0.427	0.304	1
Zfp617	0.000127	-0.68278	0.139	0.074	1
Tk1	0.01982	-0.68298	0.497	0.383	1
Bcl9l	0.011813	-0.68377	0.115	0.072	1
1190002N	0.119466	-0.68392	0.221	0.17	1
X99384	0.074813	-0.68392	0.345	0.269	1
Med25	0.018543	-0.68427	0.355	0.256	1

Igf2r	0.474316	-0.68428	0.212	0.186	1
Pik3ca	0.007629	-0.68441	0.342	0.24	1
Tfb2m	0.558516	-0.68465	0.118	0.104	1
Limk2	0.039027	-0.68494	0.103	0.068	1
Ranbp9	0.009411	-0.68497	0.276	0.199	1
Rnf168	0.03103	-0.68498	0.2	0.144	1
Cutc	0.00596	-0.68506	0.252	0.173	1
Lipo1	0.16301	-0.68528	0.145	0.115	1
Zdhhc4	0.065177	-0.6862	0.312	0.238	1
Slfn8	0.000845	-0.68645	0.358	0.244	1
Casp2	0.000564	-0.68649	0.179	0.106	1
Rprd2	0.015993	-0.68657	0.215	0.149	1
Nbr1	0.515594	-0.68663	0.288	0.25	1
Acbd5	0.066102	-0.68669	0.358	0.274	1
Tmem39b	0.260429	-0.68685	0.161	0.13	1
Tmem19	0.303472	-0.68721	0.221	0.181	1
Dock11	0.019024	-0.68747	0.355	0.262	1
Ift57	0.188931	-0.68785	0.155	0.123	1
Prkaa1	9.12E-05	-0.68835	0.27	0.169	1
Iqgap2	0.187196	-0.68841	0.479	0.393	1
Mettl16	0.037759	-0.68845	0.233	0.176	1
Fbxo3	0.813265	-0.68907	0.206	0.189	1
Cwf19l2	0.018723	-0.68914	0.185	0.128	1
Tusc2	0.193068	-0.68966	0.367	0.298	1
Fibp	0.351637	-0.69003	0.433	0.364	1
Ythdf3	0.131901	-0.69055	0.388	0.303	1
Cryzl1	0.003225	-0.69066	0.47	0.334	1
E130309D(	0.018422	-0.6908	0.236	0.168	1
Phf6	0.047155	-0.69109	0.23	0.174	1
Mrps28	0.851764	-0.69151	0.542	0.477	1
Slc25a1	0.000502	-0.69224	0.288	0.185	1
Btaf1	0.000518	-0.69224	0.436	0.3	1
Rab13	3.86E-05	-0.69258	0.339	0.224	0.767227
1700017B(	0.002631	-0.69355	0.309	0.214	1
Ccdc25	0.247145	-0.69392	0.252	0.204	1
Tmem39a	0.040504	-0.69493	0.179	0.126	1
Zbtb11	0.004576	-0.69503	0.17	0.108	1
Ccnh	0.161258	-0.69523	0.388	0.305	1
Twf1	0.064874	-0.69556	0.664	0.513	1
Ecd	0.011819	-0.69564	0.267	0.191	1
Mtif2	0.265697	-0.69571	0.148	0.119	1
Rasgrp2	0.138145	-0.69572	0.8	0.71	1
Stoml1	0.056599	-0.69573	0.245	0.184	1

Cenpt	0.000749	-0.69667	0.13	0.074	1
Mx1	3.02E-05	-0.69682	0.245	0.145	0.599243
Mtmr2	0.011998	-0.69685	0.291	0.211	1
Rilpl1	0.000105	-0.69708	0.248	0.151	1
BC003331	0.162463	-0.69752	0.324	0.264	1
Stx2	0.070827	-0.6978	0.188	0.14	1
Rngtt	0.001932	-0.69826	0.188	0.12	1
Hdac9	0.016697	-0.69836	0.242	0.177	1
Bcas3	0.001062	-0.69853	0.355	0.249	1
Ankrd16	0.356837	-0.69898	0.118	0.098	1
St3gal4	0.015488	-0.69899	0.755	0.707	1
Snhg7	0.020049	-0.69945	0.121	0.078	1
Coro2a	0.007273	-0.69976	0.179	0.118	1
Nrp	0.001139	-0.69976	0.452	0.323	1
Spns1	0.358013	-0.69986	0.224	0.188	1
Ecsit	0.016762	-0.70012	0.188	0.133	1
St6galnac6	0.395263	-0.70046	0.121	0.102	1
2610027L1	0.00405	-0.70157	0.406	0.289	1
Eno3	0.067862	-0.70171	0.206	0.159	1
Rbbp5	0.003931	-0.70251	0.161	0.103	1
Cdc42bpb	0.425028	-0.7031	0.258	0.221	1
1500011B(	0.611348	-0.70415	0.194	0.173	1
Nup214	0.055502	-0.70417	0.227	0.17	1
2610203C2	0.001992	-0.70443	0.218	0.143	1
Gtpbp2	0.011386	-0.70463	0.312	0.228	1
Zfp120	0.003302	-0.70571	0.191	0.122	1
Aqr	0.089859	-0.70625	0.294	0.227	1
Zfhx2	4.59E-05	-0.70644	0.209	0.121	0.912392
Adck1	0.067053	-0.70649	0.191	0.144	1
2410004B1	0.042803	-0.70696	0.279	0.211	1
0610009B2	0.19902	-0.70771	0.439	0.357	1
Exosc10	0.000341	-0.70811	0.273	0.172	1
Sephs1	0.298026	-0.7082	0.215	0.177	1
Mtif3	0.020284	-0.70918	0.148	0.101	1
Brd1	0.003289	-0.70928	0.376	0.268	1
Zwint	0.019238	-0.70933	0.124	0.081	1
Zzz3	0.003071	-0.71021	0.233	0.153	1
Shoc2	0.072928	-0.71036	0.282	0.216	1
Acox3	0.016652	-0.71119	0.191	0.132	1
Lrrk1	0.138398	-0.71158	0.239	0.192	1
Oxnad1	0.025624	-0.71158	0.121	0.081	1
Parp12	0.000513	-0.71185	0.597	0.44	1
Ccnk	0.024522	-0.71268	0.239	0.173	1

Scamp4	0.012058	-0.71271	0.433	0.32	1
Rpl34	0.969169	-0.71331	0.279	0.252	1
Tbcd	8.75E-05	-0.71379	0.258	0.159	1
Daglb	0.006076	-0.71395	0.642	0.477	1
Poc1b	0.005877	-0.71411	0.109	0.065	1
Acot1	0.30361	-0.71521	0.476	0.392	1
G530011O	1.69E-05	-0.71551	0.709	0.524	0.335092
Relt	0.929319	-0.71621	0.127	0.123	1
Wdtc1	0.00339	-0.71667	0.224	0.147	1
1700021K1	0.000136	-0.71719	0.167	0.093	1
Neurl4	0.001137	-0.7173	0.121	0.068	1
Pgap1	0.000208	-0.7179	0.242	0.153	1
Nop2	4.81E-05	-0.71811	0.309	0.19	0.955227
Pak1	0.000497	-0.71847	0.706	0.535	1
Tmem8	0.007332	-0.71853	0.145	0.094	1
Ncbp1	0.000796	-0.71858	0.248	0.16	1
A230046K(	0.137784	-0.71882	0.421	0.33	1
Snx27	0.137389	-0.71907	0.345	0.271	1
Ercc4	0.039942	-0.71932	0.109	0.073	1
A630001G	0.000769	-0.72002	0.282	0.187	1
Pigk	0.080217	-0.72078	0.421	0.332	1
Cirh1a	0.010717	-0.72121	0.255	0.181	1
Lyrm5	0.173078	-0.72158	0.176	0.141	1
Btbd19	0.834818	-0.7216	0.133	0.132	1
Rassf2	0.011241	-0.72163	0.282	0.204	1
Ulk1	0.022933	-0.72356	0.209	0.152	1
Ube4a	0.375073	-0.7245	0.179	0.149	1
Nr1h3	0.000552	-0.72462	0.952	0.919	1
Etfdh	0.012899	-0.72514	0.242	0.171	1
Top3b	0.249915	-0.72531	0.145	0.12	1
Osbpl2	0.262546	-0.72546	0.179	0.145	1
Ppp6r3	0.004872	-0.72549	0.333	0.242	1
Ankib1	0.859086	-0.72562	0.155	0.145	1
Dhrs7b	0.032566	-0.7258	0.445	0.343	1
Aatf	0.07055	-0.72592	0.276	0.216	1
Zfp410	0.368091	-0.72621	0.124	0.105	1
9030624J0	0.163442	-0.72667	0.455	0.377	1
E030024N1	0.001566	-0.72678	0.388	0.267	1
Trip4	0.531222	-0.7278	0.139	0.122	1
Fkbp15	7.78E-06	-0.72794	0.57	0.373	0.154486
Orc4	0.00607	-0.7285	0.3	0.211	1
Kdm5c	0.13104	-0.72886	0.291	0.228	1
Cadm1	0.512117	-0.72896	0.894	0.822	1

Ep300	0.064075	-0.72973	0.282	0.22	1
Rap2b	0.610997	-0.72983	0.679	0.558	1
Znhit2-ps	0.046574	-0.72993	0.267	0.199	1
Zfp445	0.091229	-0.72995	0.203	0.157	1
Usp33	0.00098	-0.73017	0.176	0.108	1
Cdkal1	0.027968	-0.73034	0.127	0.086	1
Abcc4	0.013385	-0.73092	0.133	0.087	1
Cib2	0.563807	-0.73165	0.173	0.151	1
Fam195a	0.005958	-0.73169	0.367	0.267	1
Aimp2	0.039228	-0.73216	0.309	0.231	1
Osgin1	0.199303	-0.7325	0.2	0.158	1
Clspn	0.009603	-0.73263	0.112	0.069	1
Zfp579	0.001911	-0.73285	0.133	0.079	1
Zkscan1	0.007999	-0.73329	0.127	0.08	1
Herc4	0.807948	-0.73358	0.291	0.261	1
Smarcd1	1.90E-05	-0.73368	0.273	0.161	0.376409
Ddx28	0.001946	-0.73395	0.173	0.11	1
Trak1	0.089524	-0.73419	0.161	0.12	1
Prkra	0.026401	-0.73482	0.391	0.298	1
Cog7	0.024634	-0.73494	0.264	0.191	1
Ndufaf1	0.001892	-0.73498	0.148	0.088	1
Slc7a8	0.323324	-0.73505	0.821	0.723	1
Med6	0.006486	-0.73515	0.206	0.14	1
Pdzd8	0.38649	-0.73539	0.203	0.168	1
Galnt2	0.225406	-0.73565	0.488	0.391	1
Ano10	0.013579	-0.73584	0.245	0.182	1
Pias2	0.956814	-0.73602	0.239	0.219	1
Cept1	0.192336	-0.73682	0.703	0.63	1
Naip1	0.000288	-0.73718	0.164	0.093	1
Ifit2	0.00214	-0.73743	0.612	0.475	1
Adam9	0.08129	-0.73748	0.609	0.485	1
Bst1	0.09022	-0.73967	0.273	0.218	1
Dctn5	0.01882	-0.74007	0.533	0.4	1
Plrg1	0.020545	-0.74184	0.197	0.139	1
Pop4	0.000674	-0.74201	0.285	0.185	1
Ctdspl2	0.633164	-0.74218	0.1	0.089	1
B230208H	0.13223	-0.74317	0.609	0.505	1
Kcnj16	0.871704	-0.74344	0.142	0.132	1
Cenpk	0.001571	-0.74351	0.121	0.069	1
Kctd6	6.01E-05	-0.74363	0.397	0.26	1
Npl	0.012402	-0.74397	0.161	0.107	1
Slc9a6	0.000939	-0.74494	0.267	0.171	1
1110032A(	0.043799	-0.74517	0.158	0.111	1

Gm4951	0.008111	-0.74533	0.618	0.477	1
Rab3gap1	0.001857	-0.7454	0.23	0.147	1
Capn7	0.043417	-0.74568	0.194	0.144	1
Gmpr2	0.358878	-0.74574	0.267	0.265	1
Oraov1	0.039957	-0.74586	0.13	0.091	1
Abce1	0.280507	-0.74588	0.33	0.272	1
Igf2bp3	0.759204	-0.74602	0.109	0.11	1
4933407H1	0.237058	-0.74691	0.118	0.093	1
Rpgrip1	0.511952	-0.74755	0.23	0.198	1
Ddx56	0.184645	-0.74808	0.239	0.192	1
4933424B0	0.029255	-0.74831	0.179	0.128	1
Ggps1	0.019329	-0.74848	0.142	0.096	1
H6pd	0.000972	-0.74927	0.276	0.18	1
Eaf1	0.051009	-0.7493	0.206	0.153	1
AI837181	0.000319	-0.75096	0.445	0.305	1
P2ry14	0.175939	-0.75102	0.3	0.248	1
Rdh14	0.064253	-0.75115	0.285	0.218	1
BC028528	0.92891	-0.75172	0.191	0.181	1
Dhcr7	0.000155	-0.75181	0.339	0.227	1
Cc2d1b	0.20783	-0.75189	0.233	0.19	1
Calcoco1	0.022144	-0.75216	0.161	0.111	1
Fes	0.674768	-0.75219	0.585	0.501	1
Ckap2l	0.018771	-0.75248	0.109	0.07	1
Snx7	0.172131	-0.75252	0.167	0.129	1
Zrsr1	3.55E-05	-0.75366	0.103	0.045	0.70463
Eif2b4	0.011148	-0.75457	0.206	0.141	1
9830001H0	8.26E-05	-0.75489	0.324	0.205	1
Rab40c	0.00087	-0.75544	0.161	0.095	1
Chsy1	0.10558	-0.75588	0.127	0.094	1
0610011L1	0.063481	-0.75623	0.164	0.123	1
Zfp219	0.01485	-0.75668	0.152	0.102	1
Zfp395	0.149549	-0.75672	0.148	0.117	1
Ttc38	0.033422	-0.75718	0.139	0.099	1
Parp9	0.489961	-0.75765	0.424	0.364	1
Lair1	0.747379	-0.75785	0.888	0.788	1
Ltc4s	0.280968	-0.75793	0.312	0.256	1
Fam136a	0.035807	-0.75866	0.458	0.357	1
Fbxw7	0.706808	-0.75867	0.142	0.143	1
Zkscan3	0.025207	-0.7606	0.252	0.182	1
Mettl6	0.020037	-0.7606	0.239	0.174	1
Nudt18	0.127217	-0.76148	0.376	0.306	1
Myef2	0.148807	-0.76191	0.161	0.124	1
Ttc9c	0.016498	-0.76197	0.318	0.236	1

Tbrg3	0.018849	-0.76304	0.152	0.102	1
Dcaf15	0.221395	-0.76316	0.209	0.166	1
Grlf1	0.001846	-0.76323	0.1	0.054	1
Erlin2	0.002488	-0.76329	0.339	0.243	1
Dmpk	0.483098	-0.76375	0.924	0.818	1
Heatr3	0.871838	-0.7639	0.142	0.142	1
Hsdl1	0.152177	-0.76398	0.242	0.19	1
Srek1ip1	0.00054	-0.76427	0.397	0.268	1
Rabggta	0.323741	-0.76497	0.221	0.185	1
Kcnj10	0.627803	-0.76583	0.173	0.175	1
9030425E1	0.000937	-0.76617	0.27	0.177	1
Stab2	0.032897	-0.7662	0.548	0.425	1
Actn1	0.005626	-0.76673	0.827	0.78	1
Adck4	0.058754	-0.7671	0.23	0.173	1
Trmt2a	0.721034	-0.76732	0.215	0.193	1
Sec24a	0.027063	-0.76768	0.158	0.109	1
Naglu	0.000287	-0.76806	0.476	0.335	1
Fdxr	0.006154	-0.76857	0.173	0.112	1
Rgl1	0.681978	-0.76955	0.806	0.714	1
Tbc1d8	0.00026	-0.76973	0.236	0.145	1
Prr24	0.000169	-0.77018	0.245	0.154	1
Lrrc47	0.000159	-0.77126	0.382	0.248	1
Arl11	0.000312	-0.77189	0.342	0.225	1
1110059G1	0.205762	-0.77205	0.37	0.298	1
Cdk7	0.003435	-0.77248	0.218	0.144	1
Exosc9	0.036899	-0.77299	0.245	0.185	1
Ccdc69	0.0141	-0.77321	0.124	0.08	1
Enoph1	0.01322	-0.77325	0.215	0.151	1
Hdhd2	0.072336	-0.77377	0.391	0.302	1
Nfatc2ip	0.063067	-0.77426	0.103	0.071	1
Zfp787	0.000365	-0.77431	0.406	0.273	1
Exoc6	0.02465	-0.77454	0.276	0.202	1
Timm22	0.109891	-0.77563	0.385	0.302	1
Kdm3a	0.008918	-0.77577	0.258	0.189	1
1110020G0	0.642482	-0.77614	0.148	0.131	1
Cyp4v3	6.48E-06	-0.77704	0.603	0.426	0.128757
1300001I0	0.187892	-0.77812	0.27	0.218	1
Ap1g2	0.07471	-0.7783	0.155	0.113	1
Ankle2	0.380752	-0.77851	0.197	0.164	1
Rhbdf2	0.038343	-0.77859	0.403	0.306	1
Wdr5	0.048239	-0.77881	0.245	0.183	1
Xylt2	0.001376	-0.77915	0.209	0.132	1
1700029F0	0.193524	-0.77982	0.303	0.241	1

Nuak1	0.0011	-0.78008	0.136	0.079	1
1110034A:	0.063306	-0.78042	0.124	0.089	1
Ubxn8	0.188538	-0.78065	0.345	0.277	1
Car13	5.59E-06	-0.781	0.182	0.094	0.111024
Ap3s2	0.006624	-0.78108	0.282	0.199	1
Pik3r4	0.002806	-0.78125	0.164	0.102	1
Gm9781	0.000272	-0.78147	0.373	0.244	1
Atmin	0.001395	-0.78218	0.333	0.234	1
Dhx9	0.045581	-0.78261	0.421	0.324	1
Mast3	0.000925	-0.78263	0.309	0.209	1
Otud4	0.042777	-0.78324	0.294	0.224	1
Dnajb14	0.011839	-0.78345	0.297	0.216	1
Golph3l	0.214592	-0.78512	0.218	0.177	1
Amacr	0.053196	-0.78533	0.203	0.153	1
Nop16	0.847053	-0.78605	0.255	0.229	1
Zfp367	0.000491	-0.78607	0.115	0.061	1
Rcc1	0.033595	-0.7862	0.433	0.351	1
Abhd11	0.147907	-0.78658	0.391	0.302	1
Eepd1	0.001164	-0.78696	0.245	0.159	1
Nup153	0.004156	-0.7871	0.309	0.213	1
Nup93	0.256863	-0.7872	0.203	0.167	1
BC051226	0.000702	-0.78745	0.212	0.135	1
Iars	0.136329	-0.78774	0.209	0.168	1
Pdcd7	0.302665	-0.78788	0.318	0.262	1
Msh2	0.123505	-0.78845	0.13	0.097	1
Dnm1l	0.000437	-0.78847	0.397	0.266	1
Aim2	0.009222	-0.78848	0.436	0.322	1
Slc12a8	3.31E-05	-0.78853	0.279	0.167	0.657671
Trappc9	0.014853	-0.78867	0.315	0.23	1
Ormdl1	0.089377	-0.7897	0.236	0.183	1
Kri1	0.180509	-0.79015	0.155	0.123	1
Ofd1	0.001026	-0.79111	0.158	0.094	1
Tbc1d22a	0.002015	-0.79137	0.503	0.37	1
Zfp945	0.022168	-0.79189	0.167	0.116	1
Cdc40	0.063166	-0.79231	0.333	0.253	1
C030044B:	0.075605	-0.79261	0.188	0.141	1
Zcchc8	0.002771	-0.79278	0.236	0.158	1
Herc6	9.44E-05	-0.79331	0.415	0.281	1
Inf2	0.212941	-0.79385	0.176	0.139	1
Ncln	0.060804	-0.79395	0.494	0.372	1
BC037034	0.00074	-0.79468	0.433	0.299	1
2010305A:	0.007911	-0.79503	0.245	0.173	1
Rnf8	0.011814	-0.79546	0.188	0.126	1

Atl3	1.74E-05	-0.79616	0.318	0.193	0.344742
Wdr67	0.006372	-0.79621	0.345	0.252	1
Alg1	0.740326	-0.79633	0.415	0.356	1
Tbrg4	0.00013	-0.79681	0.264	0.163	1
5730403B1	0.000308	-0.7977	0.218	0.134	1
Atg14	0.026171	-0.79841	0.124	0.084	1
AI314180	0.035316	-0.79842	0.239	0.177	1
Ercc5	0.047716	-0.79875	0.103	0.07	1
Ubfd1	0.010268	-0.79879	0.485	0.363	1
Gnl2	0.363852	-0.79975	0.209	0.176	1
Pcnxl3	9.34E-05	-0.80185	0.282	0.176	1
Arg2	0.006198	-0.80204	0.203	0.14	1
Chmp7	0.234329	-0.80239	0.242	0.197	1
Decr2	0.039349	-0.80266	0.145	0.104	1
Pddc1	0.574543	-0.80293	0.333	0.282	1
Esrra	0.016805	-0.80298	0.464	0.353	1
Senp2	0.022028	-0.80301	0.415	0.315	1
Gdpd3	0.000167	-0.80381	0.333	0.223	1
Pus1	0.055013	-0.80398	0.336	0.259	1
Abhd13	0.001539	-0.80525	0.27	0.181	1
Klhl5	0.006613	-0.80542	0.339	0.244	1
Pde4a	0.029547	-0.80552	0.236	0.173	1
Gm15417	0.032	-0.80724	0.276	0.213	1
Pelp1	0.025539	-0.80744	0.248	0.183	1
Tspyl2	0.000384	-0.80831	0.248	0.157	1
Naip5	0.084879	-0.80843	0.233	0.179	1
Nqo2	0.062746	-0.80851	0.348	0.276	1
Nol11	0.01796	-0.80863	0.185	0.128	1
Git2	0.421893	-0.80895	0.306	0.256	1
Dse	0.946152	-0.80973	0.733	0.633	1
E230008N1	0.00032	-0.81006	0.127	0.068	1
Sacm1l	0.088936	-0.81011	0.291	0.224	1
Nedd4l	0.039068	-0.81018	0.161	0.114	1
Ccl25	0.002246	-0.81038	0.109	0.061	1
Adam10	0.150476	-0.81209	0.724	0.649	1
Dgcr14	0.014846	-0.81215	0.185	0.131	1
Sos2	0.284333	-0.8122	0.161	0.131	1
Dnajc13	0.015851	-0.81249	0.427	0.311	1
Mtpap	0.151197	-0.81257	0.245	0.194	1
Rnf111	0.058568	-0.81276	0.33	0.253	1
Aen	0.098772	-0.81276	0.221	0.174	1
Zadh2	0.013166	-0.81298	0.212	0.147	1
Plod3	0.675793	-0.81314	0.339	0.294	1

Rufy3	7.50E-05	-0.81328	0.336	0.218	1
Dedd	0.002658	-0.8138	0.23	0.155	1
Tsen15	0.072451	-0.81516	0.185	0.138	1
Tbc1d9	0.01384	-0.81656	0.412	0.319	1
Sesn1	0.010281	-0.8171	0.324	0.226	1
Prpf31	0.295425	-0.81777	0.345	0.29	1
Klhl2	0.102112	-0.81926	0.124	0.092	1
Gab1	0.163648	-0.8194	0.1	0.075	1
Pkmyt1	0.000353	-0.82007	0.194	0.119	1
Pphln1	0.006788	-0.82027	0.491	0.369	1
Arntl	0.312759	-0.82049	0.109	0.088	1
1110057K(	0.09526	-0.82102	0.212	0.167	1
Gtpbp5	0.069785	-0.82111	0.152	0.112	1
Mfsd7b	0.001834	-0.82131	0.252	0.17	1
Maml1	0.000731	-0.82134	0.188	0.113	1
Acvr1	0.008	-0.82147	0.106	0.064	1
Cpsf4	0.147247	-0.82225	0.339	0.271	1
Atp10d	0.00033	-0.82238	0.158	0.09	1
Wbp7	0.000802	-0.82286	0.221	0.14	1
Papd7	4.04E-06	-0.82347	0.248	0.14	0.080165
Nt5dc1	0.895232	-0.82381	0.106	0.104	1
Itgav	0.142535	-0.82443	0.5	0.39	1
Nacc1	0.001555	-0.82449	0.433	0.306	1
Nsun6	0.000196	-0.82463	0.139	0.074	1
Nudt3	0.07859	-0.82464	0.364	0.283	1
Tgfbr1	0.020238	-0.82519	0.418	0.315	1
2010321M	0.06813	-0.82529	0.194	0.144	1
Tarbp2	0.1621	-0.82537	0.336	0.28	1
Cox11	0.847146	-0.8255	0.112	0.105	1
Parvb	0.000209	-0.82609	0.648	0.46	1
Lars2	0.029662	-0.82625	0.239	0.177	1
Nup37	0.167642	-0.82639	0.139	0.107	1
Gm885	0.193921	-0.82692	0.5	0.414	1
Fxn	0.240415	-0.8276	0.242	0.197	1
Ripk3	0.07043	-0.82803	0.585	0.467	1
Rnf185	0.000496	-0.82825	0.233	0.147	1
Dbf4	0.157662	-0.82947	0.185	0.15	1
Gopc	0.92881	-0.82979	0.136	0.127	1
Whsc2	0.720352	-0.83037	0.155	0.138	1
St6galnac4	0.370512	-0.83177	0.518	0.423	1
Slc35a1	0.069607	-0.83197	0.255	0.194	1
Pi4ka	0.008479	-0.8322	0.252	0.175	1
Anapc4	0.004904	-0.83225	0.245	0.166	1

Ikzf5	0.00075	-0.83249	0.139	0.079	1
Cluap1	0.859034	-0.83412	0.127	0.119	1
Birc2	0.014508	-0.83432	0.3	0.217	1
1810014F1	0.04608	-0.83497	0.439	0.348	1
Tada1	0.247088	-0.83541	0.227	0.188	1
Tex2	0.013993	-0.83561	0.142	0.095	1
Cox15	0.274697	-0.8363	0.1	0.079	1
Foxk1	0.053898	-0.83698	0.179	0.132	1
Ankra2	0.367061	-0.83795	0.212	0.178	1
Lrrc41	0.010385	-0.83841	0.37	0.263	1
Slc25a20	0.003456	-0.83984	0.258	0.177	1
Nf2	0.000444	-0.83991	0.185	0.109	1
Fam178a	0.061662	-0.83997	0.321	0.249	1
Nanp	0.481007	-0.84052	0.1	0.085	1
Zfp948	0.003416	-0.84074	0.109	0.063	1
Mettl11a	0.86791	-0.84108	0.194	0.182	1
Vps54	2.58E-05	-0.84263	0.294	0.182	0.511701
Gbp8	0.130649	-0.84292	0.588	0.489	1
Fsd2	0.045636	-0.84434	0.194	0.143	1
Rnaseh1	0.200488	-0.84482	0.267	0.215	1
Ppp2cb	0.507955	-0.84492	0.267	0.227	1
Cep250	0.047915	-0.84546	0.155	0.112	1
Pigc	0.001277	-0.84641	0.224	0.143	1
Adck5	0.019027	-0.8471	0.236	0.173	1
Smurf1	0.39586	-0.84754	0.13	0.11	1
Dyrk1a	5.31E-05	-0.84762	0.288	0.175	1
Cd300lb	0.000722	-0.8491	0.47	0.345	1
Opa1	0.137062	-0.84916	0.209	0.161	1
Ints12	0.001612	-0.84926	0.279	0.186	1
Zbtb1	0.011437	-0.8506	0.182	0.123	1
Ftsj3	0.04725	-0.85064	0.258	0.192	1
Tlk2	0.010561	-0.8509	0.245	0.173	1
Camk1d	0.409728	-0.85099	0.573	0.477	1
Atad3a	0.057781	-0.85102	0.267	0.206	1
Rpp14	0.144172	-0.85137	0.273	0.216	1
Zbtb22	0.021234	-0.85203	0.176	0.123	1
Znhit3	0.017352	-0.85278	0.194	0.134	1
Scaf4	0.135326	-0.85294	0.142	0.108	1
Cpt1a	0.712859	-0.85298	0.452	0.387	1
Clasp1	0.435192	-0.85399	0.118	0.099	1
Banp	0.576839	-0.85478	0.148	0.13	1
Ctsf	0.565597	-0.8551	0.333	0.279	1
Usp42	0.015332	-0.8554	0.133	0.087	1

Fkbp5	0.029574	-0.8555	0.406	0.306	1
Ppp5c	0.170506	-0.85611	0.485	0.369	1
Smc5	0.072535	-0.85638	0.206	0.156	1
4930471M	6.87E-05	-0.8571	0.6	0.428	1
Gpkow	0.019658	-0.85717	0.206	0.146	1
Scyl3	0.164548	-0.85734	0.136	0.106	1
Dolk	5.00E-05	-0.85736	0.139	0.071	0.99277
5730455P1	0.000989	-0.85749	0.112	0.061	1
C230081A1	0.001519	-0.85803	0.336	0.235	1
2810006K2	0.001255	-0.8583	0.173	0.105	1
Ostm1	0.020955	-0.85837	0.376	0.281	1
Zbtb2	0.008599	-0.85861	0.13	0.081	1
1810049H1	0.041938	-0.86047	0.203	0.148	1
Ccr5	0.010327	-0.86138	0.73	0.681	1
Farsb	0.013871	-0.86145	0.348	0.254	1
Acer2	0.00339	-0.86157	0.115	0.068	1
Rrp8	0.002269	-0.86204	0.248	0.166	1
Tbp	0.058136	-0.86322	0.103	0.071	1
Eps15l1	0.033148	-0.86324	0.306	0.23	1
Tfrc	0.359395	-0.86344	0.206	0.171	1
Rxra	0.64862	-0.86357	0.724	0.625	1
Cables1	4.60E-05	-0.86368	0.136	0.068	0.914178
Chaf1a	0.003892	-0.86404	0.148	0.093	1
Kat2a	0.010361	-0.86536	0.321	0.232	1
Nudt2	0.083915	-0.86657	0.318	0.248	1
Ubxn2b	0.292359	-0.86662	0.1	0.081	1
Zfp608	0.015617	-0.86686	0.118	0.075	1
Ap3m1	0.160436	-0.8675	0.242	0.192	1
Fam175b	0.014098	-0.86791	0.348	0.261	1
Rrp1b	0.009477	-0.86866	0.1	0.06	1
Fam26f	0.004887	-0.8689	0.827	0.665	1
Fbxw8	0.06495	-0.86933	0.197	0.15	1
Pcgf1	0.216777	-0.87011	0.118	0.093	1
Plcl1	0.011143	-0.87084	0.158	0.105	1
Ear2	0.009964	-0.87172	0.721	0.704	1
Prmt3	0.098141	-0.87308	0.182	0.139	1
Zfp467	0.061677	-0.87343	0.636	0.492	1
Parg	0.107144	-0.87375	0.145	0.11	1
Gpd1l	0.106554	-0.8742	0.155	0.115	1
Slc35b4	0.005281	-0.87467	0.282	0.2	1
Icos	0.020688	-0.87475	0.333	0.252	1
Map2k4	0.153115	-0.87512	0.218	0.173	1
Gm12504	0.018362	-0.87528	0.148	0.099	1

Tirap	0.182716	-0.87549	0.121	0.096	1
Hdac6	0.067028	-0.87641	0.103	0.073	1
Drosha	0.020017	-0.87794	0.164	0.113	1
3110002H1	0.005615	-0.87827	0.188	0.126	1
Cep164	1.10E-05	-0.87849	0.306	0.182	0.219175
Slc7a2	3.36E-06	-0.87946	0.476	0.327	0.066691
Trappc8	0.033095	-0.87969	0.282	0.21	1
Rreb1	0.012532	-0.88052	0.37	0.276	1
Fam98a	0.023142	-0.88071	0.139	0.094	1
Zfp932	0.009754	-0.88109	0.148	0.098	1
Arap3	0.000218	-0.88165	0.121	0.062	1
Myst2	0.004859	-0.88259	0.306	0.219	1
Ptplad1	0.003735	-0.88274	0.315	0.22	1
Wdr41	0.000594	-0.8833	0.47	0.335	1
Dph5	0.235233	-0.88391	0.148	0.12	1
Rnf103	0.076508	-0.88411	0.461	0.359	1
Atp11c	0.003738	-0.88467	0.294	0.207	1
Nprl2	0.470285	-0.88484	0.152	0.132	1
Ccdc90b	4.33E-05	-0.88516	0.388	0.248	0.85933
Vrk2	0.046616	-0.88578	0.588	0.46	1
Rpl30	0.923235	-0.88632	0.109	0.103	1
Suv420h2	0.056582	-0.88664	0.206	0.151	1
Dlat	0.23099	-0.88673	0.306	0.25	1
Ctdp1	0.028492	-0.88768	0.221	0.165	1
Hmga2-ps1	0.089957	-0.88775	0.124	0.092	1
Pigu	0.451707	-0.88803	0.224	0.194	1
Trmt61a	0.067215	-0.88929	0.148	0.11	1
Zmym3	0.184793	-0.88968	0.13	0.102	1
Med12	0.015008	-0.89006	0.276	0.199	1
2510009E0	0.947396	-0.89097	0.412	0.368	1
Lilra6	0.021237	-0.89152	0.167	0.119	1
Pold3	0.000259	-0.89236	0.367	0.245	1
Ddx58	0.018462	-0.89242	0.485	0.368	1
Hspa13	0.581196	-0.89294	0.188	0.186	1
Serpinb1b	0.002167	-0.89336	0.127	0.074	1
Slc41a3	0.000749	-0.89351	0.376	0.26	1
Ocrl	0.004615	-0.89377	0.276	0.193	1
Isoc1	0.014137	-0.89383	0.261	0.189	1
Rfxank	0.004246	-0.89398	0.209	0.14	1
Cd300e	0.564778	-0.89439	0.403	0.354	1
Sec22a	0.556789	-0.89474	0.121	0.105	1
Slc4a1ap	0.092486	-0.89487	0.155	0.116	1
Optn	0.00437	-0.89511	0.324	0.228	1

Ogfod2	0.004301	-0.89539	0.27	0.185	1
Crlf2	0.000427	-0.89544	0.158	0.23	1
Rg9mtd1	0.240372	-0.89629	0.097	0.114	1
Ttyh3	0.005771	-0.89633	0.521	0.387	1
Ifih1	0.008287	-0.89665	0.415	0.309	1
Adap2	0.000378	-0.89859	0.7	0.516	1
Hltf	0.006583	-0.89881	0.4	0.288	1
Slc52a2	6.07E-06	-0.89886	0.315	0.19	0.120595
Plcl2	0.003611	-0.89887	0.294	0.208	1
2310003L2	0.000506	-0.89965	0.203	0.126	1
Rhot1	0.044166	-0.8999	0.255	0.191	1
2310046O	0.685157	-0.89999	0.112	0.116	1
5830433M	0.176848	-0.90004	0.133	0.103	1
Trim37	0.403996	-0.90063	0.118	0.1	1
Zfp259	0.570436	-0.90141	0.421	0.352	1
Cep350	0.162569	-0.90186	0.285	0.226	1
Aagab	0.08469	-0.90253	0.388	0.301	1
Ptrh1	0.152463	-0.90508	0.106	0.081	1
Slc29a3	0.099519	-0.90512	0.421	0.335	1
Rmi1	1.36E-05	-0.90578	0.406	0.257	0.270851
Gdpd1	4.98E-05	-0.90645	0.433	0.294	0.989535
Amz2	0.190866	-0.90656	0.242	0.194	1
Golm1	0.013085	-0.90778	0.164	0.109	1
Haus8	9.40E-06	-0.90796	0.488	0.321	0.186742
Zfp362	0.00225	-0.90804	0.224	0.149	1
Phrf1	0.683572	-0.90849	0.2	0.178	1
Tmem51	0.212295	-0.9085	0.752	0.667	1
Gm5431	0.36499	-0.90917	0.164	0.138	1
Dmtf1	0.105734	-0.90935	0.158	0.118	1
Pdgfc	0.01968	-0.90935	0.47	0.366	1
Slc7a7	0.625906	-0.90937	0.727	0.623	1
Wdr36	0.022079	-0.90989	0.315	0.234	1
Src	0.002139	-0.9103	0.17	0.106	1
Terf2	0.002048	-0.9103	0.248	0.169	1
Zdhhc5	0.028509	-0.9105	0.239	0.178	1
Rad17	0.054305	-0.91091	0.188	0.137	1
Eif2c4	0.091293	-0.91177	0.139	0.102	1
Repin1	0.000196	-0.91192	0.152	0.084	1
Mgat4a	6.06E-06	-0.91247	0.312	0.18	0.120253
Pak4	0.081517	-0.91266	0.118	0.086	1
Prpf4	0.102569	-0.91291	0.188	0.146	1
Usp31	0.000875	-0.91312	0.103	0.054	1
Arl16	9.13E-05	-0.91342	0.127	0.064	1

Ldlrap1	0.003321	-0.91351	0.361	0.261	1
Tfip11	0.000652	-0.9143	0.233	0.147	1
Apobr	0.038338	-0.91465	0.448	0.359	1
Txndc16	0.040334	-0.91469	0.173	0.123	1
Clip2	0.308485	-0.91569	0.115	0.094	1
5530601H(	0.010341	-0.91609	0.191	0.132	1
Tbc1d1	0.069105	-0.91614	0.524	0.408	1
Prep	0.66828	-0.91719	0.127	0.113	1
Alg6	0.440595	-0.91749	0.1	0.085	1
Slc30a5	0.458894	-0.91797	0.339	0.283	1
Xpo6	0.002939	-0.918	0.361	0.252	1
Rbl2	0.083527	-0.91816	0.2	0.155	1
Etv5	0.037601	-0.92005	0.518	0.409	1
Abcc5	0.009607	-0.92016	0.445	0.335	1
Ddx10	0.066142	-0.92036	0.239	0.182	1
Rasgef1b	0.025746	-0.9211	0.927	0.788	1
Aldh16a1	0.079321	-0.92117	0.342	0.269	1
Nfx1	0.034175	-0.92118	0.155	0.107	1
Edc3	0.014082	-0.92156	0.115	0.072	1
1500001M	3.07E-05	-0.92259	0.239	0.14	0.60913
Tulp4	0.588779	-0.92365	0.176	0.154	1
Pik3r5	0.000509	-0.92381	0.227	0.144	1
1110054M	0.025316	-0.9242	0.182	0.127	1
Arhgap19	0.002618	-0.92771	0.564	0.423	1
Mtl5	0.008357	-0.92798	0.106	0.064	1
Irak4	0.020846	-0.92804	0.53	0.414	1
Mrpl49	0.108325	-0.92813	0.261	0.199	1
Ap3d1	0.054037	-0.92817	0.482	0.377	1
Ate1	0.070152	-0.92846	0.264	0.201	1
Rad1	0.108309	-0.92862	0.164	0.126	1
Fcna	0.000647	-0.92945	0.976	0.96	1
Taf6	0.019072	-0.92972	0.212	0.153	1
Gas2l3	4.91E-06	-0.92983	0.333	0.205	0.097512
Cul4b	0.001231	-0.93079	0.315	0.22	1
Mbip	0.015084	-0.93084	0.191	0.132	1
Pds5a	0.276466	-0.93086	0.103	0.082	1
Rabep2	0.943712	-0.93088	0.118	0.113	1
Sdad1	0.073602	-0.93096	0.17	0.128	1
Ctr9	0.006102	-0.93188	0.191	0.126	1
Ubqln4	0.018545	-0.93195	0.248	0.18	1
Rab7l1	6.97E-05	-0.93298	0.361	0.233	1
Tmem86a	0.433287	-0.93404	0.924	0.826	1
Smpd2	0.371936	-0.93466	0.121	0.1	1

Zc3h3	0.003709	-0.93493	0.2	0.133	1
Gtf2h4	0.029012	-0.93515	0.167	0.116	1
Nthl1	0.012191	-0.93652	0.112	0.07	1
Rab19	0.900962	-0.9369	0.106	0.101	1
Itgb3bp	0.124561	-0.93694	0.194	0.151	1
Socs5	0.036543	-0.93718	0.127	0.088	1
Pdcl	0.339505	-0.93863	0.164	0.136	1
1700096K1	0.003215	-0.93907	0.148	0.092	1
Siglece	0.507564	-0.93919	0.852	0.756	1
Sympk	0.153143	-0.93971	0.345	0.271	1
Map3k10	0.000141	-0.93972	0.142	0.076	1
Phf11	0.20803	-0.94012	0.324	0.27	1
Ino80	0.338713	-0.94072	0.155	0.129	1
Tmem199	0.058759	-0.94083	0.352	0.267	1
Zfp513	0.070121	-0.94144	0.227	0.173	1
Orm1	0.103174	-0.94156	0.115	0.141	1
Cog3	0.091617	-0.94194	0.115	0.085	1
5730419I0	0.003713	-0.94272	0.112	0.065	1
Micall2	0.10433	-0.94302	0.182	0.139	1
Mib2	0.448352	-0.94321	0.13	0.111	1
Snapc1	0.109133	-0.9439	0.27	0.211	1
1810048J1	0.000562	-0.94462	0.182	0.11	1
Fbxo38	0.003167	-0.94479	0.327	0.227	1
Akr1c13	0.017602	-0.94541	0.464	0.349	1
B4galt4	0.091393	-0.94605	0.282	0.216	1
Col4a3bp	0.188604	-0.94605	0.148	0.116	1
1700021F0	0.916101	-0.94723	0.239	0.224	1
Gpr55	0.040953	-0.94726	0.124	0.086	1
2010109K1	0.072266	-0.94735	0.424	0.326	1
Serpina3g	0.000118	-0.94752	0.658	0.501	1
Ccdc106	1.95E-05	-0.94763	0.527	0.361	0.386373
Ppfia4	0.003029	-0.94779	0.521	0.391	1
Ambp	1.08E-05	-0.94795	0.173	0.268	0.21394
Acp6	0.156236	-0.94808	0.203	0.163	1
Nup155	3.05E-05	-0.94939	0.197	0.11	0.60478
Ppp2r3d	0.963347	-0.94975	0.167	0.16	1
Fbf1	0.01038	-0.94985	0.1	0.06	1
Gfm1	0.008404	-0.95085	0.2	0.135	1
Cdk18	0.005275	-0.95107	0.158	0.102	1
Vcpip1	0.014372	-0.95124	0.27	0.199	1
Ppil3	0.555863	-0.95199	0.306	0.267	1
Rcbtb1	0.630541	-0.95309	0.136	0.119	1
Slc27a1	0.05491	-0.95378	0.212	0.161	1

Mtap	0.096709	-0.95451	0.212	0.161	1
Fance	0.103341	-0.95601	0.155	0.116	1
5033406O	3.37E-05	-0.95676	0.385	0.249	0.669962
Tcf7l2	0.491314	-0.95677	0.773	0.69	1
Nsun5	0.004909	-0.95723	0.124	0.076	1
Rabl5	0.006468	-0.95774	0.536	0.396	1
Stambp	1.08E-05	-0.95951	0.345	0.211	0.214101
Thada	0.001601	-0.95954	0.109	0.061	1
Ccdc43	0.004732	-0.96028	0.155	0.098	1
Zgpat	0.055006	-0.96079	0.173	0.128	1
Icmt	0.002881	-0.96101	0.182	0.118	1
Ndst1	0.004106	-0.96127	0.348	0.249	1
Armc5	0.086915	-0.96128	0.2	0.152	1
Helz	0.287751	-0.96156	0.13	0.107	1
1500002O	0.155564	-0.9618	0.227	0.18	1
Zfp187	0.184613	-0.96193	0.224	0.178	1
Zbtb24	0.185337	-0.96202	0.115	0.089	1
Tars2	0.012094	-0.96267	0.185	0.125	1
Ikbke	0.000146	-0.96367	0.448	0.307	1
Tex10	0.090263	-0.96371	0.161	0.123	1
Pacs2	0.000381	-0.96374	0.364	0.237	1
Ddx26b	0.000103	-0.96459	0.206	0.12	1
Whamm	0.053323	-0.96461	0.267	0.204	1
Ddhd2	0.081017	-0.96495	0.164	0.122	1
Pou6f1	0.012852	-0.96543	0.115	0.073	1
1810032O	0.000314	-0.96554	0.306	0.197	1
Ddx19b	0.000935	-0.96586	0.17	0.104	1
Thoc5	0.015692	-0.96624	0.248	0.176	1
Plxna4	0.012843	-0.96636	0.112	0.07	1
Tmem161a	0.007013	-0.96646	0.288	0.205	1
Pygb	0.301091	-0.96722	0.43	0.344	1
Adh1	0.093318	-0.96765	0.091	0.116	1
Zfp53	0.314994	-0.96895	0.106	0.086	1
Aldh1l1	0.909152	-0.96945	0.115	0.113	1
Dennd1c	0.0139	-0.96963	0.309	0.228	1
Rnpc3	0.227562	-0.97138	0.133	0.105	1
Casp8ap2	0.148885	-0.97145	0.227	0.177	1
Abcf3	0.273066	-0.97146	0.161	0.13	1
Pik3r2	0.004708	-0.97328	0.291	0.204	1
AU019823	0.007988	-0.97341	0.2	0.135	1
Pdhx	0.002087	-0.97347	0.155	0.094	1
1700009P1	0.349328	-0.97372	0.127	0.108	1
Sclt1	0.814513	-0.97421	0.109	0.101	1

Alyref2	0.017325	-0.97447	0.188	0.132	1
Wdr77	0.281891	-0.97523	0.236	0.191	1
Kif23	0.000567	-0.97615	0.233	0.149	1
Aaas	0.000271	-0.97635	0.236	0.148	1
Fam102a	0.404321	-0.97677	0.2	0.167	1
B9d1	0.005917	-0.9772	0.121	0.074	1
Fzd7	0.007041	-0.97729	0.167	0.111	1
4933403F(	0.011826	-0.97781	0.236	0.166	1
Nt5dc2	0.512844	-0.97803	0.118	0.102	1
Afmid	7.74E-05	-0.97834	0.321	0.203	1
Aco1	0.005082	-0.97849	0.391	0.277	1
Nxt2	0.000119	-0.97865	0.467	0.328	1
Galt	0.146277	-0.97996	0.245	0.197	1
Slc39a11	0.54707	-0.9805	0.176	0.157	1
Nudt16	0.054472	-0.98337	0.321	0.247	1
Slc25a45	0.159328	-0.984	0.348	0.283	1
Aptx	0.396486	-0.98506	0.233	0.201	1
5430427O	0.002583	-0.98547	0.424	0.311	1
Med14	0.018588	-0.98555	0.227	0.162	1
Pgm2l1	0.070716	-0.98562	0.212	0.16	1
Smg5	0.783834	-0.98578	0.13	0.119	1
Gmeb1	0.00232	-0.98588	0.194	0.123	1
Hk3	0.089012	-0.98738	0.679	0.523	1
Tdrd3	0.912497	-0.98768	0.106	0.1	1
Tmem80	0.016072	-0.98801	0.252	0.181	1
Snx13	0.013546	-0.98891	0.27	0.191	1
Camsap2	0.027864	-0.98895	0.139	0.094	1
Nbas	0.003415	-0.99003	0.115	0.068	1
Snx30	0.004427	-0.99052	0.333	0.24	1
Fam118a	0.004075	-0.99053	0.127	0.076	1
Tusc1	0.011806	-0.99126	0.164	0.111	1
Med27	0.230865	-0.99148	0.136	0.108	1
Drg2	0.048044	-0.9917	0.324	0.249	1
Klraq1	0.000216	-0.9921	0.397	0.256	1
Sepn1	0.000887	-0.99291	0.355	0.245	1
Ube3b	8.99E-05	-0.99356	0.273	0.166	1
Med24	0.011642	-0.99397	0.148	0.099	1
Zfp217	0.00805	-0.99453	0.288	0.206	1
Txndc11	0.321299	-0.99499	0.179	0.148	1
Gpatch4	0.018882	-0.99535	0.255	0.186	1
Letmd1	0.004416	-0.99546	0.27	0.186	1
Zfyve19	0.883804	-0.99608	0.167	0.16	1
Hist1h4h	0.044278	-0.99613	0.103	0.069	1

Sergef	0.000816	-0.99666	0.248	0.16	1
Tmem68	0.001401	-0.99676	0.342	0.242	1
Tatdn1	0.032504	-0.99688	0.176	0.127	1
Thoc1	0.187518	-0.99736	0.203	0.161	1
Nt5m	0.00192	-0.99822	0.242	0.161	1
Rrn3	0.018504	-1.00007	0.161	0.108	1
AI987944	0.006904	-1.00025	0.167	0.109	1
Psmg3	0.105957	-1.00118	0.355	0.277	1
Inpp5b	0.048404	-1.00185	0.161	0.116	1
Pmm2	0.101416	-1.00247	0.264	0.207	1
Stard5	0.108823	-1.00263	0.612	0.49	1
Fubp3	0.007657	-1.00284	0.179	0.121	1
Foxn2	0.000326	-1.00289	0.309	0.202	1
Kctd13	0.120849	-1.00376	0.236	0.186	1
Tyw1	0.00078	-1.00402	0.197	0.122	1
Stab1	0.002787	-1.00458	0.394	0.284	1
Zhx1	0.007898	-1.00485	0.33	0.239	1
Traf6	0.001311	-1.00633	0.173	0.104	1
D2Wsu81e	0.000256	-1.00638	0.252	0.155	1
Hps5	0.003614	-1.00695	0.224	0.152	1
Gins2	0.00098	-1.00761	0.336	0.232	1
Pick1	0.141709	-1.00764	0.13	0.099	1
Wdr12	0.139778	-1.00979	0.233	0.183	1
Fam118b	0.114432	-1.01099	0.233	0.183	1
Kpna6	0.014636	-1.01106	0.242	0.171	1
4930444A(	0.003108	-1.01205	0.258	0.175	1
Bnip1	0.002723	-1.01248	0.279	0.193	1
P2ry13	0.540992	-1.01278	0.558	0.448	1
Pikfyve	0.078777	-1.01369	0.391	0.316	1
Gna15	0.03087	-1.01493	0.306	0.23	1
Mapk9	0.00033	-1.01519	0.476	0.324	1
Cenph	0.030125	-1.01598	0.127	0.088	1
C1rl	0.000698	-1.01612	0.294	0.199	1
Ipo8	0.00038	-1.01615	0.276	0.179	1
Polr3h	0.014866	-1.01704	0.258	0.182	1
Mrpl47	0.002966	-1.01719	0.173	0.109	1
Asb13	0.557131	-1.01887	0.176	0.154	1
Cryba4	0.011871	-1.01934	0.179	0.122	1
Tgfbrap1	0.025255	-1.0194	0.233	0.17	1
Fyco1	0.002941	-1.02004	0.185	0.12	1
Lbx2	0.091875	-1.02034	0.227	0.178	1
Cpn1	0.614614	-1.02148	0.109	0.096	1
Setd1a	0.026514	-1.02167	0.155	0.107	1

Frrs1	0.083103	-1.02192	0.342	0.268	1
Umps	0.0119	-1.02248	0.348	0.256	1
Pcolce2	7.93E-06	-1.02285	0.185	0.282	0.157451
Rad51l3	0.005911	-1.02483	0.197	0.131	1
2210012G(	0.14472	-1.026	0.264	0.21	1
A630033H	0.515202	-1.02645	0.158	0.168	1
Krit1	0.016232	-1.0284	0.182	0.125	1
Enox2	0.001829	-1.02912	0.318	0.22	1
Manba	0.413574	-1.02925	0.145	0.123	1
Cinp	0.028418	-1.02988	0.276	0.208	1
Rnf121	0.158478	-1.03	0.118	0.088	1
Ikbkap	0.304916	-1.03132	0.106	0.085	1
Kcnk13	0.000732	-1.03235	0.548	0.394	1
Arhgap23	0.000499	-1.03273	0.133	0.073	1
I830077J0:	0.021154	-1.03341	0.176	0.122	1
Zswim6	0.004339	-1.03435	0.409	0.288	1
Tmem168	0.000675	-1.03437	0.464	0.326	1
Asxl1	0.13426	-1.03488	0.206	0.16	1
Exoc4	0.000561	-1.03692	0.221	0.139	1
Slc38a9	0.089963	-1.03734	0.185	0.141	1
Zdhhc17	0.002855	-1.03871	0.112	0.064	1
Ccdc22	0.083687	-1.03941	0.445	0.349	1
Ccnc	0.002618	-1.03985	0.197	0.128	1
9430016H(	0.01407	-1.03987	0.297	0.214	1
Abi2	0.103959	-1.04079	0.139	0.104	1
Hist2h2be	0.018752	-1.04104	0.109	0.07	1
Dopey1	0.091255	-1.04183	0.148	0.111	1
1700040I0	0.008002	-1.04204	0.333	0.249	1
Treml4	0.738476	-1.04277	0.827	0.746	1
Nkiras1	0.288989	-1.04417	0.167	0.136	1
Slc25a22	0.025882	-1.0446	0.188	0.133	1
Isoc2a	0.001752	-1.04524	0.279	0.187	1
Exoc2	0.305815	-1.04593	0.182	0.15	1
Exoc6b	0.016631	-1.04634	0.164	0.113	1
BC018507	0.772312	-1.04681	0.118	0.109	1
Zfp191	0.375184	-1.04734	0.127	0.105	1
Noa1	0.083403	-1.04766	0.139	0.102	1
Rab3gap2	0.00047	-1.04984	0.27	0.176	1
Vps13c	0.001231	-1.05071	0.191	0.12	1
Paqr9	0.47679	-1.05201	0.603	0.474	1
Bcl2l12	0.321958	-1.05249	0.197	0.163	1
Eef2k	0.288307	-1.05415	0.115	0.092	1
Vps8	4.83E-05	-1.05488	0.161	0.087	0.959659

Uspl1	0.542523	-1.05553	0.139	0.12	1
Gtf2e1	0.011985	-1.05684	0.109	0.068	1
Armc8	0.449966	-1.05795	0.494	0.415	1
Mex3c	0.258253	-1.0581	0.148	0.119	1
Havcr2	0.015825	-1.05898	0.315	0.237	1
Gpt2	1.02E-05	-1.0591	0.606	0.424	0.203242
Atxn1l	0.000251	-1.05941	0.273	0.174	1
Hfe	0.325823	-1.06264	0.839	0.741	1
Dopey2	0.000488	-1.0632	0.303	0.204	1
Hist1h1d	0.043987	-1.06345	0.185	0.135	1
Akna	0.525467	-1.06375	0.233	0.202	1
6030458C1	0.001195	-1.06437	0.261	0.172	1
Patl1	0.004716	-1.06466	0.336	0.241	1
1110054O	9.54E-06	-1.06568	0.206	0.111	0.189454
Rnasel	5.26E-05	-1.06805	0.515	0.363	1
Ube3c	1.47E-05	-1.06819	0.224	0.125	0.291181
Haus7	0.02692	-1.06889	0.288	0.213	1
1810044D	0.000148	-1.06913	0.121	0.062	1
Snrnp35	0.000748	-1.06941	0.188	0.114	1
Ttc32	0.007821	-1.07237	0.391	0.276	1
Kcne3	0.004835	-1.07406	0.127	0.077	1
1810012P1	0.075248	-1.07501	0.221	0.171	1
Cacna1s	0.024484	-1.07519	0.148	0.103	1
Elp3	0.003445	-1.07552	0.179	0.115	1
Fam45a	0.23256	-1.07596	0.23	0.186	1
1110028C1	0.072261	-1.07618	0.118	0.085	1
Tcta	0.040168	-1.07688	0.158	0.112	1
D230025D	0.00038	-1.07762	0.164	0.094	1
P2ry12	0.317993	-1.07808	0.53	0.437	1
Fem1a	0.388913	-1.0796	0.109	0.09	1
Atg4a	0.057079	-1.07976	0.142	0.104	1
Slc25a44	0.196016	-1.08015	0.182	0.144	1
Fgd2	0.668332	-1.08049	0.709	0.584	1
Nf1	0.021436	-1.08227	0.242	0.172	1
Atad5	0.000783	-1.08365	0.127	0.07	1
Atxn3	0.000147	-1.08369	0.224	0.134	1
Fam82b	0.143145	-1.08375	0.206	0.16	1
Cops7b	0.013531	-1.08382	0.145	0.095	1
Ptk2	0.006715	-1.08494	0.282	0.202	1
4833419F2	0.000168	-1.08723	0.285	0.183	1
Eif2ak3	0.007063	-1.08733	0.13	0.082	1
Mapk8	0.001069	-1.08758	0.176	0.106	1
Llgl1	0.143479	-1.08794	0.103	0.076	1

Gpr77	0.117539	-1.08829	0.218	0.169	1
Wdyhv1	0.065717	-1.08918	0.361	0.279	1
Gtf3c1	0.004857	-1.08968	0.173	0.111	1
Cln6	0.230993	-1.0897	0.494	0.408	1
Hist4h4	0.006648	-1.09193	0.23	0.157	1
Ppp1r13b	0.416628	-1.09279	0.242	0.209	1
Prcc	0.849329	-1.09303	0.1	0.092	1
Tinf2	0.000761	-1.09312	0.23	0.149	1
Dhdds	0.047293	-1.09325	0.273	0.206	1
Exoc8	0.041759	-1.09359	0.136	0.096	1
Stxbp5	0.000319	-1.09406	0.2	0.12	1
Fahd2a	0.109105	-1.09424	0.112	0.083	1
Timd4	0.0004	-1.09534	0.9	0.868	1
Clec9a	0.00018	-1.09575	0.255	0.161	1
Dlg1	0.000151	-1.0959	0.197	0.116	1
Pofut1	0.234812	-1.09624	0.145	0.117	1
Rasal2	0.003436	-1.09658	0.182	0.118	1
Wdr55	0.015192	-1.09679	0.315	0.23	1
Fkrp	0.540881	-1.09807	0.158	0.136	1
Rad9	0.736294	-1.09852	0.106	0.108	1
BC032203	0.016433	-1.09989	0.124	0.08	1
Riok2	0.621509	-1.10011	0.176	0.177	1
Slamf9	0.002609	-1.10015	0.718	0.554	1
Ccdc91	0.032881	-1.10021	0.212	0.156	1
Tnip2	0.000298	-1.10083	0.3	0.193	1
Zdhhc9	0.110863	-1.10384	0.358	0.291	1
St7	0.000775	-1.10395	0.291	0.19	1
Fbxl20	0.323992	-1.10499	0.23	0.193	1
Trim33	0.009996	-1.10506	0.206	0.14	1
Gm11545	0.034729	-1.10517	0.112	0.074	1
Wdr48	0.125301	-1.10622	0.197	0.152	1
Oas1a	0.777502	-1.10716	0.742	0.609	1
Pinx1	0.200482	-1.10722	0.152	0.119	1
Homer3	0.102578	-1.10726	0.148	0.116	1
Helb	0.012063	-1.10872	0.145	0.095	1
Pnp2	0.005036	-1.1098	0.161	0.102	1
Pcdh17	2.04E-05	-1.11019	0.124	0.059	0.404276
Tnpo1	0.014197	-1.11043	0.164	0.112	1
E230029C(	0.0022	-1.11057	0.403	0.282	1
2310037I2	0.020708	-1.11062	0.161	0.11	1
Pik3ap1	0.128717	-1.11168	0.118	0.09	1
Gpn3	0.016164	-1.11522	0.158	0.106	1
Dhx30	0.146707	-1.1154	0.194	0.151	1

Kif21b	3.24E-05	-1.11733	0.206	0.113	0.643405
Nlrc5	0.001216	-1.11861	0.355	0.246	1
Zfp64	0.089029	-1.12144	0.118	0.086	1
Eif2c1	0.176476	-1.12353	0.191	0.152	1
Wdfy1	0.035675	-1.1243	0.23	0.169	1
Naa25	0.014967	-1.12566	0.203	0.141	1
Nle1	0.140495	-1.12646	0.118	0.089	1
Tnip3	0.00017	-1.12712	0.433	0.298	1
Erap1	0.325816	-1.1277	0.215	0.182	1
Hps3	0.104201	-1.12774	0.382	0.294	1
Slc37a4	0.005874	-1.12997	0.145	0.091	1
Lcorl	0.027347	-1.13005	0.215	0.154	1
Foxm1	0.000522	-1.1301	0.1	0.051	1
Nek6	0.326749	-1.13022	0.115	0.095	1
Ptpn9	0.027394	-1.13089	0.291	0.219	1
Zxdc	0.015068	-1.13162	0.164	0.11	1
Zfx	0.001689	-1.13223	0.355	0.246	1
Ftsj1	0.142824	-1.1328	0.139	0.107	1
Mtg1	0.016191	-1.13348	0.142	0.096	1
Metap1d	0.167709	-1.13494	0.191	0.152	1
Zxda	0.005714	-1.13509	0.13	0.08	1
Nnt	0.067759	-1.13548	0.182	0.133	1
Cars2	0.045638	-1.13552	0.248	0.186	1
Mcoln1	7.29E-06	-1.13637	0.312	0.189	0.144799
Dock1	0.832867	-1.13795	0.13	0.122	1
Ogfod1	1.41E-05	-1.13935	0.139	0.068	0.279184
Plcb3	0.001202	-1.14089	0.209	0.132	1
Dynlt1a	0.014849	-1.14121	0.53	0.391	1
A430084P(	0.835559	-1.14292	0.821	0.723	1
Fam20b	0.000225	-1.14352	0.236	0.147	1
Ankrd26	0.001261	-1.14353	0.118	0.067	1
Rqcd1	0.010343	-1.14418	0.203	0.14	1
Zkscan6	0.016972	-1.14681	0.136	0.09	1
Mms19	0.031211	-1.14701	0.206	0.15	1
Cd3eap	0.003946	-1.14806	0.279	0.193	1
Frmd4b	0.442332	-1.14838	0.761	0.636	1
Lrrc14	8.79E-06	-1.14899	0.233	0.128	0.174553
Pitrm1	0.039096	-1.15204	0.179	0.127	1
Hic1	0.000129	-1.15226	0.458	0.319	1
Ctla2b	0.921629	-1.15316	0.909	0.814	1
Tlr7	0.011223	-1.15434	0.542	0.411	1
Gm14548	0.006873	-1.15508	0.161	0.105	1
Stk10	0.155804	-1.15536	0.403	0.312	1

Vps33b	0.6569	-1.15625	0.115	0.102	1
Nudt1	0.024011	-1.15657	0.173	0.121	1
Gm14446	0.307815	-1.15694	0.106	0.085	1
Stard4	0.003238	-1.15705	0.142	0.087	1
1110031I0	0.101851	-1.15716	0.206	0.16	1
Arfgef2	0.000149	-1.15768	0.324	0.216	1
Gpn1	0.005991	-1.15806	0.258	0.178	1
BC049352	0.002824	-1.15831	0.336	0.239	1
Fmn1	0.003528	-1.15848	0.185	0.12	1
Tdp1	0.122058	-1.16243	0.127	0.094	1
Nfam1	0.034585	-1.16259	0.736	0.604	1
Znrf2	0.005178	-1.16306	0.279	0.193	1
Il18bp	1.19E-05	-1.16349	0.924	0.894	0.235471
Cysltr1	0.000474	-1.16615	0.485	0.345	1
Fhad1	0.000956	-1.16858	0.127	0.071	1
Bmp2k	0.001045	-1.17021	0.191	0.12	1
Ppip5k2	0.986253	-1.17033	0.103	0.1	1
Mogs	0.002169	-1.17033	0.388	0.269	1
B430306N(	0.002144	-1.17167	0.588	0.436	1
3110056K(	0.040228	-1.17263	0.133	0.092	1
Rfc3	5.62E-05	-1.17317	0.345	0.224	1
Mtor	0.000347	-1.17367	0.188	0.112	1
Hey1	0.005397	-1.17447	0.148	0.094	1
Entpd7	0.001147	-1.17598	0.242	0.16	1
Ccdc126	0.003817	-1.1776	0.182	0.117	1
Wdr13	0.202545	-1.17776	0.179	0.142	1
Madd	0.002959	-1.17997	0.464	0.333	1
Ptpro	0.078489	-1.18038	0.342	0.271	1
Pde1b	0.000352	-1.18176	0.285	0.185	1
Ercc3	0.00021	-1.18309	0.239	0.148	1
Mob1a	8.97E-05	-1.18416	0.142	0.074	1
Cyb5r1	0.06373	-1.1843	0.485	0.391	1
Mlxip	0.000144	-1.1856	0.312	0.203	1
Ckap5	0.169298	-1.1859	0.233	0.189	1
Unc13a	0.00356	-1.18612	0.173	0.11	1
Cnpy4	0.209873	-1.18614	0.197	0.162	1
Tatdn2	0.063498	-1.18655	0.312	0.24	1
Zfp609	0.00808	-1.18669	0.164	0.108	1
Hap1	0.009608	-1.1867	0.23	0.164	1
Ireb2	0.002798	-1.18869	0.194	0.126	1
Donson	0.009034	-1.18931	0.133	0.085	1
Uhrf1bp1	0.014731	-1.18973	0.142	0.094	1
1810043G(	0.002916	-1.19021	0.264	0.179	1

Map3k4	0.307133	-1.19294	0.197	0.165	1
Zufsp	0.008672	-1.19335	0.191	0.128	1
Sfxn2	0.636807	-1.19355	0.136	0.121	1
Dhx38	0.036118	-1.19465	0.321	0.247	1
2210404J1	0.107688	-1.19602	0.109	0.08	1
S1pr2	0.000134	-1.19645	0.315	0.203	1
Zw10	0.028689	-1.19657	0.124	0.085	1
Pigf	0.173821	-1.19701	0.303	0.24	1
Tha1	0.011568	-1.19725	0.103	0.064	1
AU040320	0.011248	-1.19741	0.206	0.143	1
Mycbp	0.000412	-1.19954	0.333	0.217	1
Slc16a7	0.031105	-1.20099	0.145	0.101	1
Tpk1	0.100951	-1.20138	0.103	0.074	1
Serf1	0.373928	-1.20142	0.182	0.151	1
Slc17a5	0.143061	-1.20166	0.182	0.142	1
Fbxo31	0.008233	-1.20215	0.239	0.166	1
Tmtc1	3.47E-06	-1.2034	0.176	0.088	0.068987
Mrpl1	0.471674	-1.20482	0.17	0.175	1
Chpf2	0.002135	-1.20501	0.233	0.155	1
Frmd4a	0.025367	-1.20704	0.6	0.483	1
Saa3	0.161043	-1.20825	0.103	0.079	1
Tlr13	0.002682	-1.20866	0.455	0.343	1
Synrg	0.016843	-1.2092	0.17	0.116	1
Pcca	0.15171	-1.21047	0.173	0.138	1
Alkbh1	0.021834	-1.21172	0.17	0.12	1
Gphn	0.001477	-1.21261	0.227	0.147	1
Pilrb1	0.003392	-1.21396	0.918	0.846	1
Nid2	0.003729	-1.21656	0.197	0.13	1
Zfp710	0.034198	-1.2168	0.482	0.361	1
Slc25a26	0.005825	-1.21684	0.17	0.112	1
Tbce	0.235479	-1.21912	0.115	0.09	1
Mertk	0.000453	-1.22141	0.291	0.191	1
Rabepk	0.130564	-1.22201	0.148	0.112	1
Sec23ip	0.70168	-1.22225	0.161	0.143	1
Dnajc18	0.022644	-1.22316	0.145	0.099	1
Serpinc1	0.001761	-1.22437	0.121	0.186	1
Adar	0.429378	-1.2244	0.336	0.287	1
Hook2	0.00684	-1.22499	0.121	0.074	1
Sec24d	0.210603	-1.22509	0.191	0.153	1
Ano8	0.053208	-1.22828	0.106	0.074	1
Plekhg3	4.31E-06	-1.22831	0.27	0.157	0.085677
Celf4	0.009543	-1.22878	0.197	0.134	1
1110002N1	0.003578	-1.22901	0.173	0.109	1

Slc35b3	0.024274	-1.22913	0.179	0.124	1
Il15	0.000144	-1.23126	0.621	0.453	1
Vps45	0.025112	-1.23264	0.245	0.182	1
Yars2	0.00484	-1.23267	0.136	0.085	1
Paxip1	0.003312	-1.23549	0.176	0.112	1
Tom1	0.500419	-1.23792	0.742	0.621	1
Bco2	0.659982	-1.23862	0.103	0.092	1
Aurka	0.00593	-1.23876	0.103	0.062	1
Cp	0.532342	-1.23992	0.291	0.248	1
Rnf169	0.002461	-1.24059	0.233	0.154	1
Ropn1l	3.35E-06	-1.2424	0.461	0.301	0.066513
C230052I1	2.99E-05	-1.24245	0.182	0.099	0.593926
Heatr1	0.106651	-1.24259	0.197	0.153	1
Alg3	0.082208	-1.24349	0.239	0.185	1
Gpr35	0.002541	-1.24351	0.315	0.221	1
Mrps22	0.468289	-1.24607	0.167	0.14	1
Gm19705	0.000367	-1.24666	0.142	0.08	1
Pitpnm1	0.074629	-1.24928	0.348	0.26	1
Rnf40	0.26991	-1.24962	0.152	0.123	1
Fam149b	0.029535	-1.24984	0.13	0.089	1
Tmco4	0.011316	-1.24998	0.197	0.138	1
Fbxo32	0.00663	-1.25066	0.23	0.161	1
Ptges2	0.637679	-1.25325	0.167	0.166	1
Gpatch2	0.108367	-1.25425	0.103	0.076	1
Prmt7	0.191218	-1.25471	0.209	0.165	1
Scly	0.050948	-1.25825	0.255	0.195	1
Spg20	0.000821	-1.2593	0.309	0.207	1
Bmf	6.07E-05	-1.25972	0.118	0.056	1
Edem3	0.149444	-1.26174	0.136	0.103	1
Mrrf	0.009435	-1.26322	0.279	0.199	1
Lman2l	0.001532	-1.26423	0.306	0.208	1
Kif19a	0.008571	-1.2664	0.185	0.123	1
Pomgnt1	0.00509	-1.26706	0.206	0.137	1
Acaca	0.781112	-1.26823	0.118	0.108	1
Gm6548	0.093294	-1.26847	0.236	0.186	1
Itga9	0.791181	-1.26852	0.682	0.579	1
Slc45a3	0.009045	-1.26876	0.336	0.25	1
Fam113a	0.002648	-1.26898	0.118	0.069	1
Ankmy2	0.082679	-1.27077	0.148	0.111	1
Phf8	0.001698	-1.27077	0.233	0.153	1
Hipk2	0.250224	-1.27198	0.215	0.179	1
Gpr137	0.014516	-1.27311	0.239	0.172	1
Zdhhc13	0.986994	-1.2734	0.139	0.134	1

Kdm6a	2.47E-05	-1.27483	0.258	0.153	0.489931
Tysnd1	0.310082	-1.27663	0.176	0.146	1
Vkorc1l1	0.440718	-1.27822	0.112	0.095	1
Klhl36	0.040182	-1.27824	0.173	0.125	1
Ints8	0.275849	-1.27908	0.106	0.085	1
2700062C(	0.476038	-1.27926	0.239	0.202	1
Tcf19	0.036071	-1.27968	0.118	0.081	1
D6Wsu163	0.08134	-1.28036	0.303	0.244	1
Slc25a14	0.155604	-1.2809	0.115	0.088	1
1110019D:	0.0021	-1.28093	0.112	0.064	1
Zfp954	0.494804	-1.28171	0.124	0.105	1
0610009L1	0.000278	-1.28206	0.224	0.142	1
2310030N(	0.628555	-1.28213	0.121	0.106	1
Tmem141	0.936096	-1.28266	0.779	0.668	1
Ung	0.010414	-1.28325	0.152	0.1	1
Chm	8.20E-05	-1.2833	0.282	0.176	1
Naa16	0.389698	-1.28367	0.088	0.1	1
Opa3	0.007079	-1.28384	0.173	0.114	1
Depdc5	0.08483	-1.2871	0.109	0.078	1
Tstd2	0.602481	-1.28826	0.109	0.116	1
Igj	0.071927	-1.28927	0.118	0.086	1
2310067B:	0.055559	-1.29081	0.139	0.1	1
Rnf170	0.093397	-1.29138	0.179	0.134	1
Ggt5	0.11313	-1.29161	0.142	0.107	1
Rnf31	0.184237	-1.29166	0.533	0.439	1
Ptpn23	0.005126	-1.29251	0.215	0.146	1
Ralgapb	0.000153	-1.29403	0.233	0.141	1
Zfp532	5.81E-05	-1.29445	0.348	0.226	1
Mcm4	0.166431	-1.29873	0.206	0.163	1
Phactr4	0.001757	-1.30537	0.179	0.112	1
Thap1	0.000158	-1.30701	0.182	0.103	1
Golga3	1.11E-05	-1.30803	0.191	0.104	0.220784
1190005F2	0.997708	-1.30835	0.136	0.129	1
Phc3	0.084623	-1.30938	0.197	0.15	1
Pctp	0.006887	-1.3103	0.179	0.118	1
Samd4	0.001776	-1.31118	0.248	0.162	1
C330019G(	0.096388	-1.31172	0.152	0.113	1
Alg11	0.008965	-1.31175	0.124	0.078	1
Mios	0.001872	-1.31269	0.115	0.065	1
Mon1a	0.000436	-1.31347	0.155	0.088	1
Nsun4	0.015011	-1.31384	0.136	0.089	1
Asb6	0.127227	-1.31417	0.145	0.111	1
Cdca7l	0.015196	-1.31453	0.161	0.109	1

Haus1	0.133145	-1.31455	0.164	0.127	1
C030006K1	0.863615	-1.31571	0.109	0.102	1
A230050P2	0.042388	-1.32095	0.227	0.17	1
Alg9	0.489542	-1.322	0.145	0.128	1
Kctd18	0.010925	-1.32396	0.121	0.077	1
Wdr75	0.053457	-1.32581	0.17	0.124	1
Tmem143	0.683409	-1.33044	0.115	0.104	1
Ipo9	0.000605	-1.33119	0.312	0.204	1
Prpf18	0.018226	-1.33316	0.264	0.187	1
D19Ertd38	0.00754	-1.33548	0.152	0.097	1
Psd4	0.178999	-1.33713	0.152	0.121	1
Cox18	0.022728	-1.33714	0.221	0.158	1
Arhgap22	0.071429	-1.33808	0.215	0.17	1
Srbd1	0.001573	-1.34	0.109	0.06	1
Zfp512	0.002947	-1.34	0.118	0.068	1
Smpdl3b	0.155011	-1.34005	0.176	0.141	1
Gm13476	0.806844	-1.34293	0.558	0.498	1
Ankrd13d	0.007426	-1.34305	0.124	0.078	1
Slc9a1	0.026427	-1.34526	0.221	0.159	1
Rfx5	0.04787	-1.3488	0.103	0.07	1
Gtf2h2	0.155249	-1.34888	0.133	0.102	1
Slc23a2	0.283703	-1.34891	0.209	0.175	1
Sirt6	0.57799	-1.34936	0.1	0.087	1
Taf4a	0.11495	-1.35079	0.106	0.078	1
Elp2	0.09531	-1.35187	0.194	0.147	1
Bcl2a1c	0.074434	-1.35256	0.206	0.158	1
Fam160b2	0.000401	-1.3532	0.164	0.096	1
Carm1	0.024992	-1.35368	0.173	0.12	1
9330133O	0.118919	-1.35416	0.124	0.092	1
Vac14	1.68E-05	-1.35439	0.482	0.325	0.33298
Gdap2	0.434453	-1.35532	0.115	0.098	1
A830007P1	0.043785	-1.35709	0.294	0.218	1
Slc9a9	0.020841	-1.35735	0.5	0.399	1
Slc39a9	0.06261	-1.35915	0.185	0.139	1
Lats1	0.00065	-1.3611	0.185	0.11	1
8430406I0	0.398696	-1.3611	0.124	0.103	1
Hspa4l	0.005255	-1.36348	0.194	0.129	1
Sh3pxd2b	0.003655	-1.36368	0.224	0.153	1
Strada	0.23888	-1.36394	0.121	0.097	1
Kctd12b	0.000137	-1.36464	0.158	0.087	1
Mmaa	0.003176	-1.36478	0.136	0.082	1
Usp36	0.003927	-1.3648	0.218	0.146	1
Pik3c3	4.03E-05	-1.36515	0.279	0.174	0.800228

Slc37a3	0.002639	-1.36527	0.124	0.072	1
Zfp62	0.048559	-1.36858	0.161	0.116	1
Zbed4	0.000394	-1.36894	0.194	0.117	1
Trim30b	0.006711	-1.37167	0.179	0.121	1
Mtmr4	0.080688	-1.37242	0.124	0.089	1
C2cd2	0.616249	-1.37538	0.1	0.088	1
Aven	0.015615	-1.37563	0.194	0.133	1
Bub1b	0.000436	-1.3769	0.2	0.12	1
Fpr1	0.455194	-1.37727	0.8	0.678	1
Mrps27	0.007137	-1.37822	0.127	0.078	1
Suv39h1	0.071897	-1.37842	0.167	0.123	1
Trim30d	0.004339	-1.37887	0.491	0.375	1
Clock	0.116426	-1.38	0.152	0.114	1
Kdm4c	0.196118	-1.3801	0.121	0.095	1
Ints5	0.025724	-1.38216	0.103	0.066	1
Prr5l	0.03826	-1.38234	0.206	0.152	1
Pnpt1	0.497053	-1.38472	0.185	0.159	1
2810046L(	0.000338	-1.38489	0.158	0.089	1
Arsa	0.000962	-1.3864	0.358	0.245	1
Armcx5	0.000193	-1.39048	0.133	0.071	1
Rab2b	0.063862	-1.3908	0.127	0.09	1
Lsm14b	0.601634	-1.39119	0.127	0.112	1
Chn2	0.002405	-1.39138	0.242	0.165	1
Cep290	0.00555	-1.39241	0.239	0.164	1
Taf1b	0.879555	-1.39339	0.103	0.101	1
Fert2	0.000164	-1.39349	0.288	0.185	1
Trub1	0.003755	-1.3935	0.112	0.065	1
Mdn1	0.022904	-1.39552	0.145	0.098	1
Trim41	0.431883	-1.39653	0.43	0.365	1
Nme6	0.024404	-1.39681	0.2	0.145	1
Speg	0.009631	-1.39719	0.109	0.066	1
Immp2l	0.000889	-1.39914	0.161	0.095	1
Dhx58	0.014819	-1.4031	0.515	0.396	1
Cul2	0.001518	-1.40407	0.321	0.221	1
Gzf1	0.000243	-1.40433	0.279	0.179	1
Mecr	0.687181	-1.40566	0.145	0.129	1
Cnnm3	0.065047	-1.40603	0.124	0.09	1
Gpr65	0.24545	-1.40754	0.842	0.749	1
Agpat1	0.018136	-1.40804	0.182	0.125	1
C330018D:	9.37E-06	-1.4092	0.215	0.118	0.186092
Pogk	0.024646	-1.41017	0.109	0.072	1
Hn1l	0.035733	-1.41105	0.203	0.151	1
Cpsf1	0.069816	-1.41226	0.209	0.158	1

Atpbd4	0.05331	-1.4124	0.148	0.105	1
1110051M	0.012765	-1.41315	0.1	0.062	1
Wdr37	0.641443	-1.41316	0.133	0.137	1
BC002230	0.006736	-1.41364	0.118	0.072	1
6430548M	0.039706	-1.41379	0.376	0.287	1
Gm10033	0.0876	-1.41526	0.1	0.07	1
Snx16	0.14585	-1.41547	0.173	0.135	1
Manea	0.541794	-1.41613	0.118	0.103	1
Tsc2	0.011371	-1.41655	0.227	0.157	1
Cpeb3	5.00E-05	-1.41796	0.133	0.067	0.993492
1110018J1	2.51E-05	-1.42164	0.13	0.063	0.497549
Trmu	0.001029	-1.42224	0.136	0.079	1
Sh2d1b1	3.46E-05	-1.42969	0.27	0.166	0.686889
Pggt1b	0.048669	-1.43045	0.188	0.139	1
Batf2	0.003929	-1.43256	0.303	0.213	1
Cbr4	0.116004	-1.43409	0.209	0.163	1
Pan3	0.184391	-1.43625	0.255	0.201	1
Epb4.1l3	0.883053	-1.43744	0.515	0.441	1
Gon4l	0.00397	-1.43804	0.124	0.074	1
Meis3	0.00258	-1.44121	0.458	0.342	1
Zfp143	0.016333	-1.4415	0.118	0.075	1
Ppargc1b	0.003574	-1.44326	0.212	0.141	1
Npat	0.028378	-1.44479	0.139	0.095	1
Cc2d1a	0.003207	-1.44876	0.252	0.171	1
Prr12	0.183185	-1.44915	0.17	0.134	1
BC024659	0.02486	-1.44978	0.321	0.238	1
Ttc13	0.467678	-1.45155	0.179	0.157	1
Zfp646	0.026222	-1.45405	0.1	0.064	1
Lilra5	0.077285	-1.45776	0.824	0.74	1
Tubgcp5	0.007654	-1.46012	0.106	0.064	1
Rfwd3	0.050608	-1.46044	0.261	0.193	1
Chaf1b	0.000286	-1.46124	0.255	0.16	1
Chchd8	0.00838	-1.46183	0.312	0.229	1
Cd300ld	0.330038	-1.46304	0.912	0.858	1
Med23	0.043394	-1.47137	0.148	0.108	1
Irgm2	0.002917	-1.47477	0.345	0.241	1
Mis12	3.42E-05	-1.4775	0.17	0.091	0.678637
Pilrb2	0.034404	-1.48093	0.855	0.767	1
Lrrcc1	0.098119	-1.48104	0.124	0.093	1
Mtmr10	0.212727	-1.48358	0.167	0.135	1
Adrb1	0.692453	-1.48364	0.455	0.413	1
Dip2c	0.000842	-1.48489	0.248	0.163	1
Tifab	0.036702	-1.485	0.521	0.394	1

Heatr5a	0.070387	-1.48756	0.321	0.245	1
Snupn	6.05E-06	-1.48851	0.2	0.106	0.120126
Poglut1	0.037796	-1.48918	0.245	0.182	1
Sip1	0.016247	-1.48926	0.1	0.062	1
Wdr81	0.015793	-1.49074	0.252	0.182	1
Ncoa5	0.095495	-1.49287	0.188	0.143	1
Sumf2	0.208065	-1.4944	0.164	0.129	1
Arhgap12	0.002679	-1.49498	0.421	0.3	1
Morn2	0.024905	-1.49562	0.1	0.064	1
Wdr46	0.314269	-1.49631	0.191	0.156	1
Tmem42	0.006447	-1.49884	0.261	0.181	1
Zfp809	0.406569	-1.49934	0.118	0.099	1
Ncapg2	0.001269	-1.50021	0.333	0.229	1
D16H22S6	0.021785	-1.50151	0.255	0.185	1
Rbmx2	0.003489	-1.50287	0.27	0.187	1
Vezt	0.00224	-1.50511	0.242	0.164	1
Recql	0.419042	-1.5059	0.145	0.123	1
Map3k3	0.006041	-1.5064	0.173	0.111	1
Lin54	0.008036	-1.51	0.145	0.093	1
Lepr	0.014573	-1.51562	0.148	0.099	1
Setdb1	0.359961	-1.51615	0.161	0.134	1
Ints4	0.567603	-1.51687	0.1	0.087	1
Maml2	0.924468	-1.5216	0.152	0.141	1
Ehd3	0.002904	-1.52315	0.227	0.152	1
Dis3l	0.002904	-1.5233	0.103	0.057	1
Cpt2	0.712034	-1.52424	0.173	0.152	1
Naa40	0.173468	-1.52459	0.197	0.158	1
Tns4	0.000199	-1.52837	0.27	0.173	1
Parn	0.868914	-1.52935	0.127	0.119	1
Cd163	0.324706	-1.53371	0.509	0.48	1
Haus3	0.026256	-1.53666	0.106	0.07	1
Mgmt	0.005745	-1.5371	0.233	0.16	1
Hist1h2ac	0.843483	-1.53859	0.115	0.106	1
Syn1	0.031775	-1.5398	0.297	0.223	1
Ints9	0.006923	-1.541	0.191	0.128	1
Med22	0.000495	-1.54874	0.2	0.121	1
Poc5	0.015503	-1.54915	0.1	0.062	1
Cdc25a	0.003508	-1.55377	0.152	0.093	1
Mllt1	0.200687	-1.55533	0.112	0.088	1
Ttc21b	9.09E-06	-1.55547	0.164	0.083	0.180484
Pgap2	1.33E-05	-1.55645	0.348	0.227	0.263951
Ccdc130	0.045555	-1.55803	0.167	0.121	1
Akr1b10	0.005423	-1.55862	0.645	0.479	1

Prpsap2	0.025736	-1.55967	0.336	0.251	1
Mettl2	0.816079	-1.56001	0.13	0.119	1
Kif3b	0.000664	-1.56223	0.242	0.152	1
Dusp19	0.026926	-1.56378	0.188	0.131	1
Rfx7	0.472237	-1.56391	0.1	0.085	1
Rapgef2	0.003813	-1.56567	0.361	0.258	1
Dennd4b	0.016687	-1.5659	0.312	0.232	1
Sass6	0.010346	-1.56654	0.285	0.205	1
Cradd	0.213612	-1.56662	0.179	0.144	1
Ube2t	0.02534	-1.56721	0.145	0.098	1
Siae	0.004165	-1.56973	0.17	0.112	1
Rbm15	0.337852	-1.57002	0.115	0.092	1
Pla2g4a	0.774655	-1.57206	0.567	0.487	1
Lrp5	0.020444	-1.57366	0.439	0.335	1
Tdrd7	0.001488	-1.57432	0.285	0.192	1
Ttll3	0.065669	-1.57446	0.142	0.103	1
Srgap3	0.000381	-1.57454	0.264	0.173	1
Usp45	0.263405	-1.57465	0.136	0.112	1
Tspan15	0.076934	-1.57525	0.527	0.503	1
Arhgap4	0.309734	-1.57572	0.385	0.31	1
Rptor	0.058658	-1.57767	0.127	0.091	1
Pigm	0.282402	-1.57881	0.136	0.111	1
Wdsub1	0.141801	-1.57898	0.13	0.101	1
Camsap1	0.57598	-1.58584	0.106	0.092	1
Rgag4	0.000672	-1.58745	0.27	0.176	1
Rbp4	0.000183	-1.5876	0.267	0.326	1
Cwc22	0.996106	-1.59025	0.121	0.115	1
Edc4	0.007569	-1.59225	0.148	0.095	1
Gm608	0.143499	-1.59358	0.109	0.082	1
Actr5	0.062952	-1.59425	0.103	0.071	1
Rac3	0.370116	-1.59582	0.13	0.14	1
Wdr20a	0.007365	-1.59665	0.103	0.062	1
Lyrm4	0.005302	-1.59924	0.145	0.092	1
Nploc4	0.446808	-1.60037	0.273	0.231	1
Gm5506	0.204864	-1.60275	0.124	0.097	1
Pla2g6	3.07E-06	-1.6033	0.239	0.13	0.060996
Fgg	0.00031	-1.607	0.17	0.246	1
Tsen54	0.433884	-1.60798	0.115	0.098	1
Poc1a	0.000715	-1.61051	0.136	0.078	1
Dpy19l1	0.000967	-1.61248	0.261	0.167	1
Cox10	1.74E-05	-1.61579	0.224	0.125	0.345119
2700007P2	0.033319	-1.6168	0.13	0.088	1
Tmem101	0.065712	-1.61682	0.148	0.109	1

Ints1	0.027371	-1.61772	0.17	0.119	1
Cdc73	0.39475	-1.61896	0.13	0.107	1
Apitd1	0.002234	-1.61905	0.2	0.13	1
Naip6	0.002658	-1.62011	0.112	0.063	1
Nup205	0.047248	-1.62235	0.173	0.128	1
Fam179b	0.175844	-1.62319	0.109	0.083	1
Dpagt1	0.413665	-1.62412	0.218	0.185	1
Csnk1g1	0.073244	-1.62414	0.106	0.075	1
Gemin6	0.01551	-1.62655	0.161	0.109	1
Rc3h2	0.355033	-1.6269	0.215	0.178	1
Rpp38	0.000327	-1.6285	0.127	0.068	1
Coq4	0.017819	-1.62898	0.1	0.062	1
Slc10a3	0.004985	-1.63051	0.33	0.242	1
Csrp2bp	0.201905	-1.63302	0.215	0.175	1
Ddx23	0.027169	-1.63388	0.109	0.071	1
2210015D1	0.003692	-1.63487	0.312	0.216	1
Rhobtb2	0.321963	-1.63492	0.112	0.09	1
Slc22a17	0.09103	-1.63496	0.379	0.296	1
Sh3bp2	0.002274	-1.63625	0.394	0.281	1
Ticam2	0.033008	-1.63637	0.212	0.157	1
Anks1	0.000166	-1.63713	0.339	0.22	1
3110082I1	0.063824	-1.63788	0.167	0.123	1
Ypel2	0.000724	-1.63807	0.1	0.051	1
P2ry6	0.984894	-1.63829	0.661	0.562	1
Iffo1	0.000157	-1.63833	0.182	0.106	1
Hps4	0.007476	-1.643	0.152	0.098	1
Rfx1	0.003114	-1.64336	0.127	0.075	1
Aldoc	0.015512	-1.64355	0.291	0.216	1
Dusp28	0.027986	-1.64626	0.13	0.087	1
Ptprm	0.003968	-1.65047	0.345	0.243	1
Mad1l1	0.003701	-1.65063	0.13	0.077	1
Cbfa2t2	0.424684	-1.65098	0.109	0.091	1
Alg13	8.94E-06	-1.65269	0.273	0.16	0.177574
Hic2	0.007705	-1.65391	0.103	0.061	1
Aig1	0.013778	-1.65504	0.155	0.105	1
Grwd1	0.428755	-1.65676	0.094	0.104	1
Dhx29	0.002453	-1.65822	0.121	0.07	1
Cpped1	0.012047	-1.65889	0.191	0.127	1
Osbpl11	0.016867	-1.66018	0.318	0.231	1
St3gal3	0.020249	-1.66228	0.155	0.107	1
Tlr1	0.103782	-1.6627	0.312	0.248	1
Tmem104	0.001657	-1.66498	0.385	0.274	1
Tlr3	0.001631	-1.66695	0.221	0.143	1

Senp5	4.48E-05	-1.6682	0.252	0.149	0.889667
Tmem180	0.008117	-1.66853	0.148	0.097	1
Gm5069	0.029304	-1.66922	0.152	0.106	1
Tfpi	0.2486	-1.66967	0.136	0.109	1
Fadd	0.240424	-1.67021	0.13	0.105	1
Fhod1	0.153004	-1.67193	0.358	0.285	1
Cstf1	0.040043	-1.67365	0.176	0.125	1
Gab3	0.312074	-1.67656	0.179	0.152	1
Gas8	0.08824	-1.67823	0.124	0.09	1
Ankrd32	0.000112	-1.6793	0.13	0.068	1
Ctbs	0.28697	-1.68207	0.239	0.198	1
Hdac8	5.21E-05	-1.68483	0.121	0.06	1
Tm9sf4	0.000509	-1.6883	0.409	0.284	1
Fam86	0.037522	-1.68871	0.206	0.151	1
F630028O1	0.484867	-1.69282	0.233	0.235	1
Rgl3	0.010168	-1.69487	0.124	0.079	1
Stk35	0.001697	-1.69575	0.139	0.082	1
Zscan21	0.075875	-1.70324	0.106	0.074	1
Trpm2	0.128649	-1.70601	0.603	0.477	1
Vamp4	0.103892	-1.7066	0.164	0.125	1
Mettl8	0.030342	-1.70961	0.158	0.111	1
Ankrd39	0.024596	-1.70967	0.127	0.085	1
Rcan3	1.12E-05	-1.71337	0.236	0.138	0.222698
Ambra1	0.01468	-1.71877	0.155	0.103	1
Nol10	0.000367	-1.71911	0.158	0.09	1
Zfyve20	0.034737	-1.7207	0.215	0.154	1
Abcd1	0.121885	-1.72092	0.252	0.198	1
Lrrc61	0.129604	-1.72561	0.161	0.124	1
Tanc2	0.046454	-1.72893	0.327	0.25	1
Ccnt2	1.28E-05	-1.73617	0.142	0.069	0.254132
AW011738	1.76E-05	-1.73672	0.37	0.239	0.349734
Slc38a6	0.000534	-1.73791	0.339	0.228	1
Gsta3	0.031454	-1.73957	0.082	0.117	1
Mtmr1	0.222514	-1.74176	0.206	0.173	1
Car9	0.001466	-1.7465	0.176	0.108	1
Klhl15	0.030349	-1.74673	0.103	0.068	1
Gamt	0.804205	-1.74933	0.215	0.192	1
Snx24	0.007751	-1.75386	0.706	0.536	1
Coq3	0.073308	-1.75595	0.106	0.075	1
Thyn1	0.172845	-1.75671	0.43	0.337	1
Wbscr16	0.231335	-1.75879	0.142	0.112	1
Foxj3	0.177237	-1.76086	0.176	0.138	1
Hoxb8	0.001934	-1.76135	0.158	0.097	1

Nckipsd	0.000656	-1.76185	0.109	0.057	1
9030617O(	0.001325	-1.76271	0.245	0.162	1
Itsn1	0.435966	-1.76409	0.548	0.503	1
Plekhn1	0.009904	-1.76436	0.121	0.079	1
Mllt11	0.006521	-1.76837	0.145	0.091	1
Foxred1	0.011373	-1.76967	0.248	0.173	1
Alg12	0.002183	-1.77035	0.176	0.112	1
Lyve1	6.06E-06	-1.77531	0.309	0.189	0.120376
6530401N(	0.595889	-1.7765	0.218	0.189	1
Scmh1	0.991793	-1.77666	0.103	0.101	1
Tbc1d23	0.00724	-1.78017	0.279	0.194	1
Slc35d2	0.435225	-1.78151	0.133	0.112	1
Nbn	0.54557	-1.78256	0.206	0.179	1
Pvt1	8.58E-06	-1.78269	0.409	0.273	0.170396
Fbxo42	0.001811	-1.78474	0.261	0.173	1
Pign	0.000282	-1.79877	0.176	0.102	1
Nudt6	0.139366	-1.80198	0.121	0.092	1
Haus2	9.42E-06	-1.80429	0.106	0.046	0.187154
Oas3	3.33E-06	-1.80486	0.624	0.434	0.066083
Tnfrsf26	0.750363	-1.80578	0.139	0.128	1
Gm973	0.0011	-1.80598	0.115	0.063	1
Prkci	0.084986	-1.80693	0.115	0.083	1
Zfp236	0.028208	-1.81098	0.127	0.086	1
Smarcal1	0.049054	-1.81258	0.127	0.089	1
Alg8	0.034566	-1.81541	0.188	0.136	1
Creb3l3	0.000634	-1.81571	0.191	0.119	1
Zfp828	0.265363	-1.81851	0.115	0.093	1
Ddx31	0.138796	-1.81896	0.103	0.075	1
Tmco6	0.050059	-1.82062	0.155	0.112	1
Acot2	0.177404	-1.82526	0.218	0.174	1
G2e3	0.0088	-1.82715	0.136	0.086	1
Atg4c	0.753121	-1.828	0.224	0.199	1
Nprl3	0.002482	-1.8287	0.121	0.07	1
Fbxl4	0.045751	-1.82931	0.115	0.078	1
Hs2st1	0.002073	-1.82935	0.242	0.159	1
Usp12	0.106975	-1.83296	0.612	0.555	1
Akr1c12	0.000308	-1.83309	0.203	0.122	1
Alg14	0.006819	-1.83497	0.142	0.09	1
Gltpd1	0.041918	-1.83606	0.109	0.074	1
Aldh18a1	0.098191	-1.83607	0.206	0.158	1
Elk1	0.000834	-1.84312	0.242	0.158	1
6330409N(	0.015167	-1.84705	0.179	0.123	1
Ctns	0.001216	-1.85526	0.261	0.172	1

Gtpbp3	0.05037	-1.86007	0.158	0.113	1
Sap130	0.177416	-1.86138	0.155	0.121	1
Phf13	0.335183	-1.86354	0.121	0.101	1
Eral1	0.197776	-1.86629	0.103	0.079	1
5430411K1	4.38E-05	-1.87265	0.23	0.139	0.869322
Bcl2l2	0.152776	-1.87446	0.161	0.123	1
Guca1a	0.000943	-1.87888	0.779	0.601	1
Eid2b	0.149777	-1.87956	0.1	0.076	1
Bahd1	0.29399	-1.88308	0.17	0.14	1
Pde8a	0.032224	-1.88498	0.285	0.213	1
Cdk14	0.168077	-1.8854	0.191	0.152	1
Paqr4	0.000564	-1.88752	0.218	0.138	1
Usp21	0.30281	-1.90377	0.348	0.294	1
Sema6b	0.00593	-1.9047	0.139	0.088	1
Psd3	0.00032	-1.90723	0.233	0.148	1
Mthfr	0.000121	-1.91044	0.282	0.18	1
Brms1l	0.316994	-1.91145	0.118	0.096	1
Apex2	0.291662	-1.91187	0.1	0.08	1
Ift74	0.121766	-1.91247	0.136	0.103	1
Lig3	0.009516	-1.91352	0.127	0.08	1
Nvl	0.425815	-1.91521	0.13	0.111	1
Depdc7	0.014977	-1.91657	0.127	0.084	1
Gm5150	0.197582	-1.91669	0.615	0.503	1
Ccdc112	0.1394	-1.91676	0.185	0.148	1
Eya3	0.000603	-1.9184	0.212	0.131	1
Abcg3	0.088376	-1.92074	0.545	0.421	1
2610528E2	0.005493	-1.92456	0.185	0.123	1
AU022252	0.011499	-1.92547	0.179	0.122	1
Prss30	0.005646	-1.92571	0.106	0.063	1
Tmem175	0.034728	-1.92675	0.142	0.1	1
Ndst2	0.026967	-1.92919	0.285	0.211	1
Ampd2	0.491416	-1.92999	0.173	0.147	1
Mmgt1	0.990279	-1.93038	0.118	0.115	1
Dvl2	0.001151	-1.93413	0.158	0.096	1
Aplf	0.000275	-1.9347	0.115	0.059	1
Vps52	0.065185	-1.93701	0.185	0.139	1
Rnf214	0.972144	-1.94101	0.155	0.146	1
Tbc1d7	0.024578	-1.94111	0.155	0.107	1
A530064D	0.133896	-1.94276	0.245	0.198	1
Zfp282	0.028249	-1.94367	0.124	0.083	1
Zfp628	0.25673	-1.94984	0.1	0.079	1
Gtf2ird1	0.1633	-1.95646	0.133	0.101	1
D830031N	0.002953	-1.95688	0.161	0.101	1

Batf3	0.237667	-1.96324	0.664	0.606	1
Itih4	0.270136	-1.96498	0.118	0.132	1
Sh2b3	0.876299	-1.96813	0.179	0.165	1
D5Ertd579	0.566882	-1.97141	0.133	0.116	1
Pcsk7	0.000277	-1.97346	0.248	0.154	1
Zfp839	0.00706	-1.97545	0.17	0.113	1
Sh3bgrl2	0.09874	-1.97687	0.276	0.218	1
Gtpbp1	0.146049	-1.98639	0.209	0.166	1
1810011H:	0.347277	-1.98872	0.421	0.345	1
Fgd4	0.034242	-1.99292	0.206	0.147	1
Smcr7l	0.004081	-1.99638	0.176	0.113	1
Shprh	0.248667	-1.99762	0.103	0.081	1
Rad54l2	0.012251	-1.99772	0.109	0.068	1
Sipa1l2	0.050364	-1.99916	0.142	0.102	1
Sgcb	0.220415	-1.99959	0.115	0.089	1
Serpina1a	0.000672	-1.99968	0.294	0.35	1
Apob	0.000265	-2.00069	0.079	0.147	1
Pigv	0.055856	-2.0041	0.145	0.105	1
Sugp1	0.174564	-2.00552	0.191	0.149	1
Epb4.1l1	1.47E-05	-2.01519	0.276	0.164	0.292879
1810014B(	0.01046	-2.01642	0.1	0.061	1
Fga	0.008405	-2.01673	0.167	0.213	1
Khnyn	0.001003	-2.01854	0.103	0.054	1
Sec61a2	0.068017	-2.02665	0.103	0.072	1
Inppl1	0.166491	-2.027	0.391	0.315	1
Nup133	0.585453	-2.02933	0.109	0.096	1
Wdr24	0.163261	-2.03032	0.103	0.078	1
Zmynd15	0.013341	-2.0324	0.333	0.245	1
Tecpr2	5.04E-05	-2.0326	0.206	0.118	1
Pet112l	0.257899	-2.03662	0.133	0.106	1
Utp20	0.022046	-2.03812	0.103	0.066	1
Tti2	0.065965	-2.04145	0.1	0.069	1
Dcaf5	0.004184	-2.04204	0.209	0.138	1
Cd180	0.02252	-2.04337	0.155	0.105	1
Asb4	0.000126	-2.04663	0.139	0.075	1
Naip2	0.039508	-2.04984	0.318	0.237	1
Hpn	0.618881	-2.05288	0.176	0.157	1
Cog6	0.139475	-2.05413	0.106	0.079	1
Apbb3	7.36E-05	-2.05658	0.173	0.094	1
Zcwpw1	0.0003	-2.05861	0.248	0.158	1
2310028H:	0.00074	-2.05917	0.185	0.114	1
Sco1	0.0058	-2.07042	0.136	0.085	1
Pisd-ps1	0.010041	-2.07058	0.136	0.089	1

Mitf	0.00105	-2.07116	0.385	0.268	1
Cep68	0.377595	-2.07819	0.109	0.091	1
Mob1b	0.167188	-2.08501	0.133	0.102	1
Papd5	0.035461	-2.0886	0.248	0.186	1
Gpr89	0.1757	-2.09237	0.179	0.141	1
Tlr4	0.059908	-2.09462	0.321	0.25	1
Nup188	0.002763	-2.09523	0.133	0.08	1
Prkar1b	7.26E-05	-2.1004	0.252	0.153	1
Ttc7b	0.049207	-2.10305	0.1	0.067	1
Hmg20a	0.313116	-2.10557	0.112	0.091	1
Rap1gap2	0.139715	-2.11269	0.118	0.089	1
Trim46	0.003903	-2.11553	0.173	0.111	1
Casp9	0.011088	-2.11693	0.167	0.112	1
Angptl6	0.080174	-2.12521	0.127	0.092	1
Supt3h	0.001139	-2.1338	0.261	0.174	1
Nup43	0.235505	-2.13748	0.133	0.106	1
Cyp3a11	0.000177	-2.13996	0.076	0.145	1
Gpr160	0.104716	-2.14107	0.133	0.099	1
Ankrd24	0.000251	-2.14261	0.218	0.134	1
Efha2	0.000247	-2.14296	0.194	0.114	1
Pion	0.028292	-2.14538	0.406	0.309	1
Srp54a	0.164679	-2.14648	0.121	0.092	1
Pot1a	0.089295	-2.14733	0.167	0.125	1
Slc2a8	0.076713	-2.14772	0.245	0.185	1
Surf6	0.011814	-2.15096	0.127	0.081	1
Dzip3	0.248846	-2.15422	0.115	0.091	1
3110043O	0.003716	-2.15562	0.373	0.267	1
Polr3b	0.011751	-2.15721	0.139	0.09	1
Megf8	0.073517	-2.15818	0.127	0.093	1
Mthfsd	0.954445	-2.16081	0.121	0.117	1
Brwd3	0.781488	-2.16748	0.103	0.095	1
Cmpk2	0.01144	-2.16986	0.264	0.191	1
Abca9	8.54E-05	-2.18399	0.291	0.182	1
Mettl21a	0.062177	-2.19886	0.158	0.117	1
Herc3	0.00342	-2.19941	0.118	0.07	1
Prmt10	0.110103	-2.19959	0.133	0.101	1
Sept10	0.011307	-2.20173	0.167	0.111	1
Dock5	0.000402	-2.20716	0.185	0.109	1
Fam78a	0.493895	-2.2091	0.13	0.11	1
Eif2b3	0.70438	-2.21219	0.136	0.122	1
Ccdc93	0.282232	-2.21998	0.179	0.147	1
Snta1	0.177729	-2.22262	0.452	0.368	1
Ttyh2	0.002134	-2.23049	0.433	0.321	1

Rprd1a	0.036601	-2.23941	0.136	0.096	1
Dhx34	0.001923	-2.24109	0.209	0.134	1
Cwc27	0.016985	-2.24539	0.17	0.115	1
Klhl18	0.009924	-2.24974	0.121	0.076	1
Topbp1	0.001793	-2.25285	0.224	0.146	1
Pydc4	0.002005	-2.25919	0.445	0.322	1
Apoc3	0.000499	-2.27364	0.348	0.397	1
Abcc3	0.032663	-2.27611	0.658	0.618	1
Apoc4	0.642789	-2.27787	0.2	0.192	1
Tsen2	0.009794	-2.28491	0.109	0.067	1
Zbtb33	0.008183	-2.2867	0.164	0.108	1
Vps18	0.042321	-2.31139	0.252	0.189	1
H2-Q10	0.008636	-2.31263	0.136	0.186	1
A230051G	6.62E-05	-2.31725	0.115	0.055	1
Gtpbp8	0.100734	-2.3184	0.197	0.15	1
Gc	0.000683	-2.31901	0.245	0.31	1
1810021B2	0.005583	-2.32153	0.188	0.124	1
Sgsm3	4.44E-05	-2.32334	0.248	0.148	0.88224
0610010F0	0.554475	-2.32526	0.121	0.107	1
Nubpl	0.138291	-2.33222	0.152	0.118	1
Rrnad1	0.000592	-2.33563	0.1	0.051	1
C330006K0	0.025953	-2.3379	0.179	0.128	1
Arl1	0.041663	-2.34422	0.136	0.095	1
Apoa5	0.002194	-2.34518	0.055	0.106	1
Hlcs	0.012075	-2.34823	0.176	0.121	1
Atp6v0a2	0.041219	-2.34899	0.109	0.075	1
Orc2	0.001977	-2.35055	0.185	0.119	1
Pqlc2	0.003267	-2.35389	0.264	0.185	1
C920009B1	0.017325	-2.35871	0.2	0.143	1
E330016A1	2.43E-05	-2.36463	0.279	0.17	0.48354
Arhgap11a	0.048481	-2.36583	0.185	0.134	1
Nif3l1	0.42621	-2.37173	0.103	0.086	1
Nol9	0.020899	-2.37643	0.118	0.077	1
Cd200r1	0.031298	-2.3852	0.276	0.21	1
Hyou1	0.450086	-2.3886	0.121	0.103	1
Casp12	0.013687	-2.3894	0.115	0.073	1
Azgp1	0.047786	-2.39065	0.088	0.119	1
2410131K1	0.003809	-2.39135	0.109	0.063	1
Akap10	0.280652	-2.39213	0.155	0.123	1
Ccdc132	0.536639	-2.40697	0.176	0.154	1
Ipo13	0.231326	-2.41094	0.176	0.14	1
Ptger2	0.730267	-2.41502	0.167	0.152	1
Zfp935	0.001405	-2.41786	0.121	0.069	1

6330416G1	0.104891	-2.42357	0.445	0.348	1
Phkb	0.008947	-2.42877	0.179	0.12	1
D330023K1	0.07221	-2.43754	0.136	0.098	1
Fam124a	0.00411	-2.43926	0.106	0.062	1
1700113I2	0.06151	-2.4418	0.1	0.07	1
Scarf1	0.058267	-2.44299	0.161	0.115	1
Nfxl1	0.097928	-2.44336	0.464	0.368	1
Slc12a9	8.44E-05	-2.44419	0.248	0.153	1
Nme4	0.185031	-2.45214	0.185	0.148	1
Pram1	0.761211	-2.45573	0.173	0.154	1
Zxdb	0.000878	-2.45698	0.13	0.073	1
Galnt3	0.01948	-2.46184	0.136	0.092	1
Acn9	0.001243	-2.46901	0.155	0.092	1
Rasgrp1	0.601381	-2.47199	0.206	0.202	1
Tmem126l	0.749741	-2.47563	0.133	0.133	1
Enpp1	0.000172	-2.47812	0.373	0.256	1
Serpina1b	0.000349	-2.4839	0.309	0.362	1
Nlrx1	0.23977	-2.49065	0.1	0.078	1
Tbc1d25	0.000319	-2.49072	0.142	0.078	1
Kcnk6	0.125437	-2.49747	0.358	0.281	1
Rnf135	0.007949	-2.49864	0.239	0.163	1
Rhbdd1	0.124391	-2.5103	0.112	0.084	1
6330418K0	0.042192	-2.51306	0.148	0.105	1
Oplah	0.345724	-2.5174	0.109	0.088	1
Smyd5	0.001758	-2.52016	0.264	0.176	1
Mbd4	0.038625	-2.52127	0.103	0.069	1
Cep78	0.002734	-2.52259	0.109	0.062	1
Fkbpl	0.013958	-2.52339	0.152	0.102	1
Tmem62	0.00033	-2.52872	0.1	0.049	1
Cdk5rap2	0.151925	-2.53481	0.167	0.131	1
Adap1	0.007961	-2.53758	0.339	0.24	1
Oma1	0.724275	-2.5383	0.248	0.222	1
1200011M	0.025893	-2.53878	0.115	0.076	1
Spef1	0.036539	-2.54142	0.106	0.07	1
Iqsec2	0.000134	-2.54153	0.264	0.163	1
Zfp161	0.102316	-2.54651	0.106	0.076	1
Clasp2	0.569063	-2.5473	0.148	0.13	1
Tnnt1	0.023061	-2.55237	0.176	0.122	1
Zmynd19	0.025417	-2.55801	0.109	0.072	1
Rhbdd3	0.015965	-2.55832	0.215	0.153	1
Sema4b	0.133519	-2.5827	0.103	0.077	1
Tmem209	0.002617	-2.58287	0.224	0.148	1
Top1mt	0.001244	-2.59624	0.173	0.105	1

Nhlrc3	0.178446	-2.60801	0.133	0.105	1
Setdb2	0.003606	-2.60876	0.106	0.061	1
Alkbh2	0.223277	-2.60982	0.124	0.099	1
Cdk20	0.078673	-2.61975	0.115	0.083	1
Gm4013	0.029537	-2.62014	0.245	0.178	1
Mcoln2	1.10E-05	-2.62249	0.248	0.14	0.218396
Spg11	0.214334	-2.62557	0.112	0.087	1
Ganc	0.105473	-2.63207	0.158	0.121	1
Zfp691	0.011075	-2.63785	0.158	0.104	1
Ece2	0.02533	-2.65644	0.342	0.256	1
Scamp5	0.019363	-2.66164	0.436	0.327	1
Snx25	0.00208	-2.66933	0.245	0.162	1
Msto1	0.822646	-2.67352	0.1	0.101	1
Homer1	0.394596	-2.67837	0.136	0.115	1
Sfxn5	0.014525	-2.67871	0.158	0.107	1
F830016B(	0.020739	-2.68706	0.182	0.125	1
Ccdc163	0.261262	-2.693	0.127	0.102	1
Angpt2	0.002883	-2.70534	0.133	0.081	1
C130050O	0.052758	-2.70914	0.321	0.242	1
Serpina1e	0.095522	-2.71467	0.176	0.201	1
Kifc3	0.100029	-2.71649	0.303	0.235	1
Ly6g6d	0.075486	-2.72567	0.127	0.092	1
Pld1	0.047259	-2.73178	0.267	0.198	1
Trp53rk	0.159321	-2.7435	0.1	0.074	1
Rg9mtd3	0.035534	-2.75854	0.115	0.078	1
Tbc1d20	9.80E-05	-2.7615	0.145	0.077	1
Casp3	0.00171	-2.76409	0.139	0.083	1
2410022L(	0.021682	-2.77031	0.458	0.354	1
Rai1	0.008163	-2.79143	0.152	0.098	1
Mto1	0.41395	-2.7952	0.1	0.111	1
Abcb8	0.145355	-2.79527	0.112	0.085	1
Zfp90	0.014767	-2.80494	0.3	0.21	1
Wtip	0.045527	-2.81226	0.161	0.115	1
Dolpp1	0.005541	-2.82194	0.133	0.083	1
Tmem181a	0.727048	-2.82474	0.161	0.144	1
Serpina1d	0.000328	-2.8252	0.185	0.251	1
Gm15706	0.010955	-2.83337	0.197	0.138	1
Rab39	0.061544	-2.83447	0.158	0.114	1
Spats2	0.015135	-2.84227	0.1	0.062	1
Kdelc1	0.025479	-2.86457	0.121	0.08	1
Fam122b	0.003115	-2.8704	0.106	0.061	1
Tmc8	0.396278	-2.87983	0.164	0.136	1
D17H6S53	0.098507	-2.89081	0.164	0.123	1

2310040G:	0.007967	-2.89247	0.464	0.357	1
3300005D(	0.000404	-2.90423	0.27	0.176	1
6720456H:	0.026715	-2.91386	0.167	0.117	1
Arrdc2	0.262866	-2.9202	0.173	0.143	1
Gins1	0.00598	-2.92382	0.133	0.085	1
9330020H(	0.002089	-2.95432	0.121	0.07	1
Gm1943	0.092393	-2.95512	0.124	0.091	1
Ccdc61	0.016914	-2.95667	0.13	0.085	1
4930503L1	0.051846	-2.97835	0.139	0.101	1
Bloc1s3	0.134349	-2.98799	0.161	0.188	1
Pus10	0.055442	-2.99304	0.155	0.111	1
Cd80	0.074475	-3.00147	0.212	0.16	1
Mlf1ip	0.002112	-3.00614	0.115	0.067	1
Rad51l1	0.868948	-3.03797	0.118	0.116	1
Unk	0.028135	-3.045	0.103	0.068	1
Mag	0.000884	-3.06207	0.236	0.15	1
Anln	0.009056	-3.06343	0.1	0.06	1
Gm3336	0.01666	-3.06498	0.121	0.079	1
E2f3	1.67E-05	-3.08234	0.17	0.088	0.332119
Mbtps2	0.887656	-3.09629	0.176	0.171	1
Gm20257	0.007316	-3.12654	0.106	0.064	1
Kif15	0.000297	-3.13778	0.109	0.055	1
Taf6l	0.431149	-3.15244	0.133	0.113	1
Gm5523	0.119404	-3.15583	0.121	0.093	1
C8g	0.505325	-3.15767	0.097	0.105	1
1700029I0	0.053795	-3.17809	0.136	0.097	1
Idua	0.063217	-3.18298	0.182	0.135	1
Ces1c	0.000564	-3.19954	0.067	0.129	1
B3galnt1	0.147343	-3.20414	0.321	0.258	1
Samd1	3.81E-05	-3.20518	0.391	0.255	0.756096
Rpusd1	0.14646	-3.20668	0.109	0.082	1
Siglec5	0.046428	-3.21758	0.106	0.072	1
Gdpd5	0.098402	-3.23426	0.164	0.125	1
Rnf141	0.018498	-3.24419	0.197	0.139	1
1110046J0	0.005667	-3.26831	0.13	0.081	1
Kng1	0.173079	-3.28232	0.155	0.169	1
Agrn	0.000538	-3.28865	0.106	0.055	1
Tnfrsf11a	0.188319	-3.30344	0.276	0.224	1
2310008H(	0.05693	-3.30531	0.13	0.091	1
Hamp	0.025142	-3.30693	0.179	0.126	1
Mtap7d3	0.179495	-3.31848	0.115	0.088	1
Zmat1	0.090575	-3.32152	0.109	0.078	1
Rbm19	0.021703	-3.32177	0.133	0.088	1

Telo2	0.000801	-3.3228	0.142	0.082	1
9330151L1	0.12213	-3.32458	0.106	0.078	1
Rnf157	2.27E-05	-3.33299	0.2	0.11	0.450983
Atp6v0c-ps	0.293846	-3.34332	0.13	0.106	1
Gstp2	0.016996	-3.34985	0.091	0.133	1
Chst14	0.003866	-3.37215	0.215	0.142	1
Mfsd3	0.172231	-3.4191	0.152	0.12	1
Lrrc40	0.803924	-3.44525	0.106	0.098	1
Elmod2	0.001224	-3.45316	0.161	0.095	1
Lass6	0.004279	-3.50305	0.139	0.085	1
C030046E1	0.104155	-3.50745	0.115	0.085	1
Pyroxd2	6.39E-05	-3.56923	0.273	0.168	1
D630037F2	0.19122	-3.60497	0.121	0.094	1
Nckap5l	0.005632	-3.60967	0.115	0.069	1
Zfp384	0.002118	-3.65861	0.185	0.117	1
Pde8b	0.001807	-3.67924	0.248	0.169	1
Slc37a1	0.210131	-3.68128	0.145	0.116	1
Sesn2	0.004813	-3.72992	0.103	0.059	1
Gm12070	0.068451	-3.81996	0.133	0.096	1
Itpkb	2.76E-06	-3.83108	0.103	0.041	0.054783
Rwdd2b	0.001979	-3.87467	0.103	0.056	1
Poli	0.114085	-3.93218	0.103	0.076	1
F2	2.56E-05	-3.94968	0.097	0.18	0.508142
Entpd6	0.724663	-3.99919	0.133	0.12	1
Cenpo	0.11064	-4.00706	0.103	0.075	1
4930486L2	0.018088	-4.01838	0.155	0.105	1
Mrps2	0.728264	-4.07923	0.118	0.119	1
Lhpp	0.087615	-4.10107	0.112	0.081	1
Slc46a1	0.733619	-4.25799	0.164	0.149	1
Rps6kc1	0.489338	-4.25969	0.1	0.084	1
Noc3l	0.046258	-4.29332	0.1	0.066	1
Mtr	0.001389	-4.43334	0.197	0.122	1
Sh2b2	0.469745	-4.50988	0.103	0.088	1
Mblac1	0.024994	-4.8897	0.121	0.079	1
