# Supplementary material for: Transcriptomic characterization of Lonrf1 at the single-cell level under pathophysiological conditions
Source: J Biochem. 2023 Mar 8;173(6):459–69. doi: 10.1093/jb/mvad021 (PMC10226518; doi:10.1093/jb/mvad021)
Supplement: Web_Material_mvad021 [file web_material_mvad021.zip › Supplementary Table S2.pdf]

Supplementary Table S2

DEG LonFR1+vsLonRF1- in Tomhigh LSEC from normal liver

	p_val	avg_log2F(	pct.1	pct.2	p_val_adj
Lonrf1	0	1.456637	1	0	0
Ubtf	4.69E-08	0.134999	0.604	0.453	0.000853
Creb1	6.16E-08	0.139009	0.519	0.375	0.001119
Capza1	1.31E-07	0.130749	0.555	0.409	0.002379
Tfpi	1.38E-07	0.158729	0.829	0.721	0.002503
Tmcc1	1.95E-07	0.116838	0.55	0.396	0.003536
Cnot8	2.40E-07	0.125113	0.334	0.225	0.004362
Mogs	3.51E-07	0.108902	0.182	0.107	0.006372
Mindy1	4.76E-07	0.137053	0.443	0.317	0.00865
Fhod1	5.24E-07	0.117171	0.422	0.3	0.009532
Hhex	5.85E-07	0.145419	0.487	0.361	0.01064
Gas6	6.44E-07	0.12489	0.755	0.61	0.011703
Dcaf17	7.27E-07	0.11082	0.247	0.156	0.013211
Mepce	7.63E-07	0.129851	0.404	0.289	0.013864
Itga8	9.31E-07	0.13098	0.509	0.376	0.016922
Arl13b	1.01E-06	0.106555	0.307	0.207	0.018301
Etl4	1.57E-06	0.118169	0.272	0.182	0.02859
Rbm15b	3.13E-06	0.11008	0.332	0.23	0.056894
Zfx	3.30E-06	0.146095	0.421	0.313	0.059923
Fam222b	3.78E-06	0.124738	0.63	0.505	0.068738
Ppp3ca	4.91E-06	0.11965	0.832	0.726	0.089317
Gna13	6.16E-06	0.136667	0.745	0.625	0.111954
Adamts9	6.61E-06	0.101476	0.264	0.179	0.120083
Cacybp	6.75E-06	0.119133	0.45	0.337	0.122675
Tuba1b	7.17E-06	0.149424	0.753	0.614	0.130403
Gak	7.76E-06	0.100225	0.323	0.225	0.141084
Bola2	9.28E-06	0.129842	0.432	0.324	0.168605
Hexim1	9.48E-06	0.141456	0.778	0.655	0.172257
Nr2f1	1.00E-05	0.115589	0.475	0.357	0.18197
Hscb	1.02E-05	0.104475	0.21	0.136	0.185115
Enpp4	1.34E-05	0.10062	0.398	0.291	0.244089
Slc16a2	1.37E-05	0.112985	0.456	0.343	0.249901
Cdc37	1.44E-05	0.112615	0.658	0.535	0.261204
Lgals8	1.53E-05	0.113523	0.529	0.406	0.278741
Med13	1.80E-05	0.111719	0.634	0.501	0.327018
Mrpl13	1.82E-05	0.101378	0.316	0.222	0.331296
Dse	1.95E-05	0.11246	0.711	0.586	0.355029
Mb21d2	2.35E-05	0.118275	0.537	0.42	0.427902
Prelid1	3.15E-05	0.112945	0.554	0.443	0.572302
Phlda1	4.34E-05	0.158365	0.276	0.198	0.789552

Zbtb46	4.54E-05	0.127361	0.623	0.5	0.824955
Bmp2k	4.83E-05	0.108327	0.41	0.31	0.878272
Erg	5.70E-05	0.116938	0.338	0.251	1
Ywhah	6.75E-05	0.101148	0.708	0.576	1
Akr1b8	7.82E-05	0.132286	0.562	0.449	1
Rbm18	8.01E-05	0.118214	0.478	0.378	1
Stx2	8.44E-05	0.124899	0.642	0.535	1
Mpzl1	8.59E-05	0.101751	0.306	0.223	1
Adgrg3	8.95E-05	0.105398	0.813	0.686	1
Ctnnal1	0.000114	0.112111	0.224	0.157	1
Slk	0.000134	0.119812	0.885	0.801	1
Uhrf2	0.000139	0.111934	0.502	0.39	1
Smc6	0.000145	0.115842	0.781	0.683	1
Sarnp	0.000145	0.105122	0.499	0.393	1
Brwd1	0.000153	0.104333	0.442	0.343	1
Pitpnc1	0.000154	0.105878	0.923	0.849	1
Prdm2	0.000159	0.113915	0.586	0.48	1
Zfp710	0.000212	0.121309	0.575	0.464	1
Sema4c	0.000223	0.123386	0.585	0.481	1
Dlc1	0.000225	0.103402	0.986	0.952	1
Cdkn1a	0.000228	0.130056	0.655	0.56	1
Cfh	0.000241	0.118037	0.656	0.556	1
Arhgef15	0.000257	0.11343	0.91	0.82	1
Zkscan3	0.000299	0.103129	0.481	0.385	1
Wnt2	0.000314	0.11073	0.612	0.506	1
Klf7	0.000324	0.107712	0.962	0.904	1
Plpp1	0.000348	0.116798	0.938	0.861	1
Raly	0.00038	0.109135	0.705	0.595	1
Add3	0.000408	0.113973	0.85	0.781	1
Midn	0.000434	0.107224	0.673	0.554	1
Snx5	0.000461	0.115996	0.879	0.788	1
Oxr1	0.000469	0.108875	0.426	0.334	1
Ncoa3	0.000497	0.100947	0.788	0.69	1
Flrt1	0.000548	0.106882	0.314	0.238	1
Dennd4c	0.000574	0.111879	0.405	0.319	1
Pdcd6ip	0.000575	0.101655	0.721	0.61	1
Msrb3	0.000643	0.10497	0.83	0.726	1
Amotl2	0.000749	0.113691	0.762	0.643	1
Ppp1r10	0.00082	0.186555	0.651	0.571	1
Ier5	0.000833	0.124536	0.756	0.66	1
Fam193a	0.000986	0.109161	0.496	0.406	1
mt-Cytb	0.001191	-0.10403	0.999	1	1
Smad6	0.001515	0.105466	0.689	0.607	1

Cxcl10	0.001676	0.167464	0.708	0.63	1
Ube2i	0.001803	0.10073	0.553	0.459	1
Fcer1g	0.002326	0.111038	0.44	0.366	1
Phlpp1	0.0027	0.109047	0.314	0.251	1
Gpr146	0.003323	0.103128	0.54	0.453	1
Aamp	0.003414	0.11571	0.63	0.539	1
Gadd45b	0.003841	0.111814	0.564	0.484	1
Ntn4	0.003925	-0.17362	0.905	0.883	1
Tspan14	0.003976	0.100876	0.669	0.583	1
Mest	0.005111	0.105156	0.367	0.303	1
Sin3a	0.007541	0.112965	0.307	0.25	1
Kmt2e	0.024665	-0.13929	0.867	0.833	1
Phkg2	0.049461	-0.12323	0.163	0.187	1
Insr	0.068305	-0.19785	0.572	0.558	1
Fbxl7	0.097818	-0.13952	0.696	0.667	1
Slc45a4	0.101797	-0.19206	0.137	0.16	1
B4gat1	0.124203	-0.13368	0.216	0.229	1
Dmpk	0.181347	-0.10729	0.227	0.241	1
Dnajb14	0.183861	-0.15065	0.599	0.515	1
Esm1	0.185549	-0.13953	0.311	0.27	1
Tcp11l2	0.209398	-0.11494	0.325	0.329	1
Kcnq1ot1	0.23636	-0.17974	0.318	0.336	1
Gm26870	0.269371	-0.18111	0.168	0.147	1
Msr1	0.269627	-0.19009	0.555	0.529	1
Ppwd1	0.276292	-0.11249	0.229	0.238	1
Usp36	0.302755	-0.11372	0.194	0.198	1
Efnb1	0.335594	-0.14017	0.722	0.693	1
Dmxl2	0.343589	-0.10211	0.198	0.202	1
Tmsb10	0.346447	-0.11512	0.64	0.558	1
Neat1	0.347664	-0.14508	0.805	0.74	1
Plvap	0.355762	-0.11354	0.491	0.425	1
4930503L1	0.395951	-0.13445	0.438	0.413	1
Abl2	0.403282	-0.10793	0.265	0.265	1
Chmp5	0.45857	-0.10376	0.571	0.527	1
Prpf38b	0.475009	-0.12355	0.67	0.636	1
Ceacam1	0.483104	-0.12371	0.436	0.414	1
Ly6a	0.485457	-0.26069	0.494	0.476	1
Ndufs8	0.491312	-0.10032	0.311	0.303	1
Rela	0.508893	-0.10958	0.454	0.438	1
Senp6	0.550205	-0.10705	0.564	0.519	1
Hspa1a	0.560892	-0.14141	0.801	0.746	1
Myef2	0.565123	-0.10334	0.257	0.251	1
AY036118	0.610253	-0.10455	0.344	0.311	1

Zcchc17	0.638182	-0.10461	0.317	0.305	1
Gm14226	0.639426	-0.12588	0.396	0.38	1
Oaz2	0.643002	-0.10182	0.711	0.658	1
Prpf39	0.643911	-0.10206	0.414	0.395	1
Git2	0.719468	-0.10331	0.537	0.476	1
Mcee	0.782532	-0.1008	0.229	0.219	1
Fgfr1op2	0.801163	-0.10293	0.484	0.447	1
Ly6e	0.802264	-0.11668	0.763	0.719	1
Golga4	0.829941	-0.1078	0.64	0.604	1
Hoxb4	0.834468	-0.1027	0.588	0.535	1
Alb	0.84651	-0.11566	0.309	0.298	1
Rtp4	0.887377	-0.10689	0.419	0.394	1
Hspb1	0.975652	-0.16238	0.431	0.402	1
Mecom	0.978679	-0.11948	0.387	0.358	1
