# Supplementary material for: Transcriptomic characterization of Lonrf1 at the single-cell level under pathophysiological conditions
Source: J Biochem. 2023 Mar 8;173(6):459–69. doi: 10.1093/jb/mvad021 (PMC10226518; doi:10.1093/jb/mvad021)
Supplement: Web_Material_mvad021 [file web_material_mvad021.zip › Supplementary Table S3.pdf]

Supplementary Table S3

DEG LonFR1+vsLonRF1- in Tomlow LSEC from normal liver

	p_val	avg_log2F(	pct.1	pct.2	p_val_adj
Lonrf1	0	1.460828	1	0	0
Tnks1bp1	5.19E-07	0.137318	0.331	0.207	0.00944
Kri1	7.80E-07	0.144374	0.22	0.121	0.014183
Anapc5	1.21E-06	0.180389	0.53	0.386	0.02202
Zfp503	1.89E-06	0.183146	0.795	0.647	0.034345
Parp2	3.60E-06	0.109653	0.217	0.124	0.065516
Sec24b	3.80E-06	0.157789	0.411	0.284	0.0691
Armc8	4.77E-06	0.157643	0.291	0.185	0.086647
Cpeb2	5.62E-06	0.161545	0.238	0.147	0.102124
Creb3l1	6.66E-06	0.139347	0.244	0.15	0.121081
Arhgef3	6.71E-06	0.145299	0.388	0.268	0.121898
Ssbp1	1.60E-05	0.100193	0.209	0.126	0.290917
Lbh	1.69E-05	0.132874	0.415	0.293	0.306511
Atp6v0a1	2.33E-05	0.155736	0.563	0.421	0.423541
Cpsf7	2.41E-05	0.15496	0.417	0.298	0.437683
Arhgef11	2.76E-05	0.123128	0.372	0.261	0.500876
Peli1	2.77E-05	0.186677	0.587	0.467	0.503837
Zfp622	3.09E-05	0.102268	0.313	0.211	0.56075
Cdk13	4.09E-05	0.140817	0.49	0.374	0.742758
Egr1	4.40E-05	0.258726	0.858	0.782	0.800586
Tmem109	5.98E-05	0.12965	0.687	0.533	1
Arrdc1	6.09E-05	0.128694	0.283	0.19	1
Zranb2	6.44E-05	0.166742	0.465	0.356	1
Zbtb41	7.84E-05	0.122921	0.224	0.144	1
Pla2r1	8.55E-05	0.160165	0.61	0.493	1
Mast3	8.98E-05	0.14887	0.215	0.139	1
Scoc	9.12E-05	0.136887	0.409	0.304	1
Rab13	0.000101	0.111592	0.502	0.393	1
Eed	0.000109	0.128376	0.22	0.142	1
Appl1	0.000118	0.134313	0.354	0.252	1
Gtf2f1	0.000121	0.132285	0.313	0.223	1
Gigyf2	0.000125	0.12616	0.547	0.429	1
Adamts1	0.00013	0.234319	0.846	0.749	1
Il3ra	0.000131	0.133423	0.449	0.339	1
Il1a	0.000136	0.206761	0.64	0.536	1
Sh3bp5	0.000137	0.126815	0.97	0.923	1
Mest	0.000147	0.112403	0.433	0.32	1
Nono	0.000162	0.115119	0.608	0.478	1
Fam20a	0.000163	0.100996	0.264	0.177	1
1500011K1	0.000166	0.107686	0.24	0.157	1

Dennd4c	0.000176	0.118379	0.419	0.304	1
Ush1g	0.000179	0.10647	0.195	0.124	1
Ubn2	0.000181	0.1439	0.665	0.55	1
Fyttd1	0.000191	0.140236	0.461	0.35	1
Zfpm1	0.000201	0.132036	0.494	0.381	1
Cct4	0.000205	0.111477	0.518	0.408	1
Map1b	0.000229	0.136083	0.234	0.156	1
Zfp36l2	0.000231	0.15115	0.848	0.73	1
Ric8a	0.00025	0.10315	0.272	0.189	1
Vps39	0.000265	0.100061	0.244	0.165	1
Snx29	0.000273	0.100873	0.26	0.179	1
Rsad2	0.000287	0.123507	0.429	0.322	1
Fos	0.000288	0.267382	0.898	0.83	1
Sik1	0.000289	0.100739	0.173	0.107	1
Rc3h1	0.000303	0.131685	0.596	0.473	1
Chp1	0.000304	0.11669	0.435	0.327	1
Cdc42bpb	0.000335	0.119319	0.783	0.688	1
Mtmr1	0.000335	0.120917	0.185	0.119	1
Smc6	0.000359	0.139723	0.797	0.667	1
Arl4a	0.00037	0.101196	0.589	0.475	1
Hnrnpd	0.000377	0.102483	0.63	0.503	1
Prkd3	0.00039	0.125752	0.52	0.405	1
Rel	0.000397	0.140967	0.514	0.396	1
Mtdh	0.000428	0.156257	0.841	0.73	1
Calm3	0.000431	0.11717	0.553	0.449	1
Dync1li2	0.000437	0.133164	0.37	0.277	1
Abcc4	0.000448	0.118918	0.502	0.39	1
Akt3	0.000459	0.117995	0.274	0.19	1
Enpp5	0.000468	0.107103	0.327	0.236	1
Kctd12	0.000512	0.128562	0.547	0.451	1
Hnrnph1	0.000524	0.124356	0.892	0.794	1
Nabp1	0.000534	0.145768	0.23	0.156	1
Stk3	0.000549	0.131376	0.378	0.283	1
BC028528	0.000581	0.135886	0.368	0.281	1
Atxn1	0.000586	0.136872	0.467	0.361	1
Med13	0.000592	0.13562	0.598	0.486	1
Mafk	0.000601	0.129592	0.303	0.218	1
Ube2j2	0.000618	0.130357	0.358	0.266	1
Gatad2a	0.000638	0.127239	0.565	0.451	1
Ccnd2	0.000641	0.125986	0.864	0.779	1
Ahctf1	0.000653	0.14049	0.35	0.262	1
Fam189a2	0.000662	0.113709	0.343	0.252	1
2510009E(	0.000669	0.137359	0.498	0.399	1

Phactr4	0.000703	0.119949	0.47	0.372	1
Qsox2	0.000717	0.156427	0.181	0.121	1
Bicd2	0.000717	0.117552	0.734	0.636	1
Tceal8	0.000727	0.154025	0.429	0.33	1
Pcna	0.000733	0.10524	0.555	0.447	1
Ceacam1	0.000738	0.126853	0.478	0.374	1
Pld2	0.000755	0.105072	0.22	0.15	1
Pdia4	0.000762	0.107098	0.545	0.423	1
Lrrc8c	0.000765	0.139335	0.693	0.593	1
Chst15	0.00083	0.137909	0.923	0.845	1
Ccnt1	0.000853	0.118029	0.62	0.507	1
Fgd5	0.000853	0.137578	0.825	0.734	1
Rad50	0.000908	0.128224	0.28	0.204	1
Tiam1	0.000933	0.102183	0.254	0.179	1
Stab2	0.000987	0.120465	0.992	0.953	1
Alas1	0.000989	0.106002	0.226	0.155	1
Slc25a37	0.001041	0.104859	0.506	0.407	1
Numa1	0.001044	0.118121	0.675	0.548	1
Ets2	0.001048	0.125553	0.892	0.851	1
Copb1	0.001061	0.120402	0.492	0.398	1
Foxk1	0.001071	0.107686	0.22	0.154	1
Cd99l2	0.001076	0.110061	0.343	0.256	1
Smchd1	0.001136	0.128978	0.474	0.379	1
Riok3	0.001152	0.100228	0.364	0.27	1
Sft2d1	0.001194	0.110487	0.679	0.568	1
Zfp106	0.001223	0.115324	0.541	0.433	1
Atp6v1b2	0.001246	0.135304	0.415	0.327	1
Lats2	0.00128	0.105562	0.563	0.449	1
Phf12	0.001304	0.101528	0.331	0.251	1
Plbd2	0.001338	0.107501	0.417	0.319	1
Znrf1	0.001377	0.121253	0.86	0.787	1
Napg	0.00153	0.104391	0.339	0.254	1
Psmc1	0.001532	0.104533	0.453	0.349	1
Vps35	0.001584	0.103473	0.565	0.455	1
Dusp1	0.001695	0.141257	0.927	0.896	1
Ythdf2	0.00172	0.100856	0.374	0.287	1
Rnf220	0.001743	0.112809	0.423	0.333	1
Prickle1	0.001799	0.114932	0.691	0.568	1
Sgk1	0.001807	0.137703	0.937	0.879	1
Kdelr1	0.001832	0.101904	0.667	0.549	1
Ppp1r15a	0.001836	0.131436	0.929	0.858	1
Kank2	0.001876	0.129834	0.301	0.229	1
Gmpr	0.001913	0.106369	0.441	0.337	1

Junb	0.001917	0.140812	0.943	0.89	1
Sik3	0.001994	0.118015	0.427	0.335	1
Fosb	0.002003	0.155166	0.744	0.654	1
Fbxl3	0.002057	0.102776	0.533	0.428	1
Loxl2	0.002075	0.146783	0.287	0.213	1
Ywhae	0.002107	0.109952	0.872	0.766	1
Klf4	0.00217	0.199735	0.843	0.767	1
Igf1r	0.002215	0.115646	0.628	0.542	1
Suds3	0.002222	0.105345	0.455	0.356	1
Hmgb2	0.002239	0.105662	0.583	0.479	1
Tpbgl	0.002292	0.14815	0.85	0.789	1
Sgce	0.002314	0.101837	0.311	0.232	1
Wsb1	0.00234	0.111524	0.717	0.6	1
Cfap43	0.002375	0.136795	0.23	0.167	1
Galnt1	0.002488	0.110427	0.602	0.495	1
Smg5	0.002556	0.119347	0.289	0.214	1
Enc1	0.002633	0.109961	0.362	0.281	1
Lrba	0.002713	0.109487	0.195	0.136	1
Fus	0.002742	0.10426	0.963	0.906	1
Snx33	0.002745	0.10537	0.285	0.212	1
Icam1	0.002796	0.141835	0.72	0.613	1
Npepps	0.00299	0.10325	0.388	0.316	1
Slk	0.003243	0.118249	0.888	0.796	1
Cand1	0.003257	0.117156	0.323	0.247	1
Nfe2l1	0.003308	0.124397	0.85	0.75	1
St5	0.00347	0.113283	0.285	0.212	1
Ier2	0.003672	0.119003	0.632	0.557	1
Fmn1	0.003729	0.10431	0.22	0.158	1
Myc	0.003835	0.129582	0.122	0.079	1
Evi5	0.003933	0.115624	0.555	0.446	1
Golgb1	0.003935	0.103348	0.693	0.595	1
Rarb	0.004206	0.104412	0.376	0.291	1
Crybg3	0.004214	0.13865	0.411	0.319	1
Ino80	0.004421	0.101119	0.26	0.193	1
Btg2	0.00447	0.106312	0.841	0.754	1
Slc31a2	0.00455	0.125115	0.555	0.467	1
Hbegf	0.004921	0.122567	0.717	0.629	1
Zcchc6	0.004999	0.111068	0.904	0.849	1
Atf3	0.005389	0.137002	0.587	0.496	1
Snx18	0.005631	0.10564	0.47	0.386	1
Tpd52l2	0.005799	0.115104	0.386	0.312	1
Ints6	0.005799	0.133871	0.581	0.482	1
Sema3d	0.005846	0.110243	0.132	0.088	1

Jun	0.006252	0.123791	0.961	0.899	1
Emcn	0.006377	0.114573	0.809	0.71	1
Kcnq1ot1	0.006387	0.198565	0.352	0.284	1
Txlng	0.006388	0.119006	0.213	0.159	1
Zfp36	0.006627	0.110413	0.862	0.79	1
Otud5	0.006649	0.101718	0.327	0.25	1
Fgfr1	0.006905	0.103263	0.246	0.184	1
Abcg1	0.007059	0.112369	0.665	0.576	1
Gapdh	0.007223	-0.17062	0.732	0.73	1
Lasp1	0.007618	0.102716	0.669	0.573	1
Vcp	0.007817	0.109857	0.825	0.716	1
Sec14l1	0.008031	0.100603	0.516	0.439	1
Reep3	0.008779	-0.1263	0.85	0.826	1
Ikzf5	0.008884	0.120735	0.27	0.211	1
Gm15675	0.009029	0.121766	0.528	0.444	1
Atp6ap1	0.009186	0.101593	0.742	0.638	1
Kcnb1	0.009306	0.103628	0.709	0.636	1
Syne1	0.009561	0.116704	0.827	0.75	1
Fam160a2	0.009715	-0.12859	0.081	0.121	1
Fabp5	0.009913	0.111465	0.73	0.653	1
Lamtor3	0.010277	0.116849	0.547	0.466	1
Ankfy1	0.010283	0.111234	0.453	0.369	1
Rasd1	0.010283	0.119688	0.425	0.353	1
Epb41l2	0.010464	0.102801	0.927	0.874	1
Mbnl1	0.010518	0.107448	0.86	0.779	1
Crim1	0.010738	0.100743	0.904	0.821	1
P4hb	0.011031	0.107637	0.766	0.701	1
Man1a	0.011125	0.102983	0.811	0.707	1
Pik3r1	0.011221	0.106202	0.593	0.513	1
Tiparp	0.011547	0.137463	0.392	0.331	1
Mprip	0.011796	0.124196	0.799	0.701	1
Topors	0.011822	0.103271	0.573	0.496	1
Cyr61	0.011918	0.113921	0.323	0.256	1
Cited2	0.012092	0.113865	0.341	0.276	1
Kit	0.013435	0.1129	0.74	0.65	1
Col4a3bp	0.015048	0.117813	0.516	0.431	1
Ifitm3	0.015414	-0.1203	0.982	0.97	1
Rybp	0.016191	0.105036	0.48	0.396	1
Cox7a2	0.016651	-0.15939	0.72	0.721	1
Cdkn1a	0.017236	0.100875	0.618	0.556	1
Nr4a1	0.017652	0.120931	0.333	0.27	1
Thbd	0.018223	0.15675	0.449	0.376	1
Actb	0.018383	-0.13736	0.994	0.99	1

Sntb2	0.018438	0.110866	0.801	0.725	1
Rhob	0.019019	0.10074	0.913	0.873	1
Klhl4	0.019399	0.106091	0.248	0.197	1
C1ra	0.020167	0.12491	0.283	0.229	1
Rack1	0.020301	-0.12896	0.965	0.953	1
Tmcc3	0.020712	0.146499	0.817	0.745	1
Ier5	0.021167	0.103591	0.752	0.667	1
Sgpp1	0.021928	0.109092	0.839	0.753	1
Tacc2	0.023783	-0.15434	0.181	0.22	1
Clec1a	0.028599	0.112194	0.583	0.513	1
Cd55	0.028921	0.113301	0.984	0.953	1
Prrc2a	0.029311	0.102523	0.764	0.696	1
Rpl36	0.032718	-0.13085	0.978	0.95	1
Rpl32	0.033553	-0.14562	0.965	0.95	1
Chd4	0.033976	0.107915	0.866	0.778	1
B230219D	0.034923	-0.15903	0.703	0.701	1
Nectin2	0.034924	-0.14281	0.461	0.48	1
5730409E(	0.036212	-0.13223	0.14	0.174	1
Rps7	0.039377	-0.1179	0.97	0.953	1
Psmc4	0.04332	-0.10443	0.238	0.273	1
Klhl24	0.043914	0.102003	0.742	0.656	1
Rpl29	0.044715	-0.15255	0.957	0.93	1
Ctnnb1	0.044833	-0.17276	0.764	0.747	1
Cdc14a	0.044877	0.106079	0.283	0.234	1
Tbkbp1	0.047914	-0.13358	0.297	0.325	1
Srcap	0.048775	-0.13745	0.142	0.174	1
Dag1	0.052195	0.10138	0.591	0.516	1
Rprd1a	0.05227	-0.10145	0.081	0.111	1
Nmrk1	0.056573	-0.11666	0.093	0.122	1
Rpl23	0.059651	-0.10334	0.994	0.989	1
Cxcl9	0.060784	-0.27389	0.537	0.548	1
Vezf1	0.061429	0.104074	0.632	0.571	1
Sub1	0.065235	-0.15686	0.555	0.555	1
Ddx17	0.0674	-0.13532	0.697	0.711	1
Vti1a	0.069872	-0.13464	0.173	0.203	1
Pgam1	0.072909	-0.15546	0.339	0.356	1
Mbd1	0.073284	0.179176	0.494	0.436	1
Stat1	0.079382	-0.12536	0.421	0.447	1
Atp6v1e1	0.079634	-0.12717	0.661	0.66	1
Ltn1	0.082273	-0.12521	0.22	0.247	1
Tmed10	0.085056	-0.1002	0.799	0.781	1
Arpc1b	0.086014	-0.14575	0.634	0.617	1
Mvp	0.086936	-0.14979	0.335	0.353	1

Stk16	0.087822	-0.10113	0.169	0.2	1
Gm12840	0.088842	0.113075	0.874	0.788	1
Bloc1s1	0.089524	-0.13675	0.238	0.264	1
Plekhj1	0.091047	-0.12405	0.175	0.201	1
Trappc4	0.092495	-0.16316	0.264	0.283	1
Mrpl30	0.096592	-0.13426	0.321	0.35	1
Mrpl4	0.097373	-0.13181	0.289	0.314	1
Uba52	0.10178	-0.13347	0.884	0.85	1
Sem1	0.107161	-0.10683	0.87	0.833	1
9930111J2	0.107486	-0.15109	0.451	0.476	1
Actg1	0.112795	-0.18626	0.862	0.828	1
Paxbp1	0.114631	-0.1287	0.348	0.362	1
Endou	0.117996	0.105116	0.258	0.22	1
Slc45a4	0.118229	-0.2165	0.098	0.121	1
Dnajb14	0.123615	0.140861	0.573	0.505	1
Hspa1a	0.124635	0.153986	0.728	0.69	1
Elob	0.126021	-0.12939	0.77	0.705	1
Zfp512	0.126345	-0.11561	0.14	0.164	1
Araf	0.132289	-0.10658	0.358	0.382	1
Rplp0	0.133962	-0.12622	0.933	0.919	1
Rpl30	0.135231	-0.11399	0.98	0.964	1
Tmem164	0.136491	-0.13708	0.278	0.301	1
Med21	0.139955	-0.12919	0.226	0.249	1
Fer	0.141852	-0.1493	0.217	0.24	1
Rbp1	0.143262	-0.14185	0.146	0.17	1
Cd151	0.148198	-0.1228	0.498	0.493	1
Bcap31	0.158352	-0.10469	0.478	0.474	1
Psmd9	0.160316	-0.10363	0.199	0.221	1
Sod2	0.162268	-0.13097	0.244	0.264	1
Rps2	0.163759	-0.10599	0.976	0.947	1
Tmsb10	0.172253	-0.15858	0.614	0.602	1
Akt1s1	0.1735	-0.11071	0.24	0.256	1
Fam96b	0.18328	-0.12288	0.242	0.259	1
Fscn1	0.196769	-0.13312	0.463	0.464	1
Setd2	0.197732	-0.13739	0.463	0.456	1
Cavin1	0.200578	-0.11988	0.366	0.376	1
BC005561	0.201711	-0.14295	0.325	0.338	1
Inpp5f	0.207829	-0.11181	0.116	0.133	1
Gatsl2	0.208878	-0.10346	0.167	0.187	1
Rest	0.217154	-0.15197	0.366	0.367	1
Raph1	0.223412	-0.11504	0.858	0.811	1
Tecpr1	0.228925	-0.11885	0.608	0.574	1
Ifi27	0.235796	-0.10777	0.555	0.536	1

Upf3b	0.236048	-0.11715	0.258	0.275	1
1810022K(	0.247456	-0.11245	0.264	0.277	1
Fez2	0.247616	-0.1136	0.478	0.481	1
1110059G:	0.248117	-0.10626	0.187	0.204	1
Abhd16a	0.248157	-0.11441	0.394	0.399	1
Gimap9	0.249518	-0.12754	0.256	0.265	1
Vcam1	0.262082	-0.10279	0.567	0.509	1
Kif2a	0.264546	-0.11546	0.341	0.35	1
Chmp2a	0.265368	-0.10587	0.606	0.588	1
Pola2	0.273879	-0.1213	0.311	0.322	1
Usf1	0.280026	-0.11012	0.254	0.26	1
Csad	0.281923	-0.10084	0.199	0.216	1
BC004004	0.288985	-0.12629	0.236	0.243	1
Hist3h2a	0.29272	-0.15554	0.268	0.271	1
Arpc2	0.296078	-0.10182	0.764	0.723	1
Mbd2	0.30041	-0.10586	0.616	0.601	1
Vps72	0.30664	-0.1274	0.201	0.207	1
Cmtm4	0.307935	-0.10799	0.15	0.164	1
Crip2	0.31546	-0.10806	0.791	0.745	1
Hoxb5	0.315796	-0.10034	0.376	0.385	1
Spred1	0.322142	-0.1094	0.429	0.425	1
Ndufb11	0.345459	-0.12807	0.553	0.539	1
Crtap	0.357454	-0.10257	0.297	0.303	1
Pdpk1	0.361543	-0.10727	0.356	0.361	1
Khk	0.362247	-0.14317	0.262	0.267	1
Taf3	0.37461	-0.13413	0.374	0.362	1
Tm9sf4	0.382693	-0.10406	0.24	0.249	1
Egln1	0.387924	-0.11856	0.417	0.413	1
Ergic3	0.390275	-0.10633	0.337	0.339	1
Washc3	0.393754	-0.14081	0.339	0.336	1
Igkc	0.39405	-0.10557	0.189	0.199	1
Smim15	0.397035	-0.104	0.429	0.419	1
Ndufs8	0.411607	-0.1532	0.289	0.288	1
Zcchc17	0.412456	-0.12046	0.28	0.287	1
Tia1	0.422795	-0.10373	0.295	0.294	1
Cox5b	0.423531	-0.10072	0.693	0.66	1
Cyhr1	0.458203	-0.11979	0.398	0.388	1
Cct5	0.466556	-0.10147	0.378	0.381	1
Rgs3	0.466718	-0.10261	0.659	0.651	1
Esd	0.469313	-0.14153	0.398	0.378	1
Pdgfb	0.470886	-0.11292	0.122	0.13	1
Nrip1	0.47323	-0.10163	0.663	0.629	1
Rtraf	0.473292	-0.11024	0.512	0.493	1

Mrpl38	0.480968	-0.11273	0.27	0.274	1
Pxdn	0.487008	-0.1197	0.549	0.523	1
Gm42418	0.489385	-0.1684	1	1	1
Kdm7a	0.499201	-0.11199	0.242	0.244	1
Phlda1	0.506154	-0.18127	0.207	0.214	1
Myl12b	0.512333	-0.1154	0.675	0.618	1
Chrac1	0.514227	-0.11055	0.25	0.252	1
Rpl14	0.514631	-0.11194	0.949	0.924	1
Atxn7l3b	0.530241	-0.10901	0.76	0.706	1
Mpc2	0.531341	-0.10548	0.396	0.376	1
Ttc14	0.545719	-0.16562	0.343	0.336	1
Rbfa	0.55063	-0.10585	0.348	0.343	1
Cdk9	0.568003	-0.10258	0.447	0.425	1
Rwdd1	0.579532	-0.11782	0.53	0.509	1
Chmp4b	0.58697	-0.10398	0.659	0.628	1
Shoc2	0.608926	-0.10144	0.311	0.301	1
Coro1c	0.619013	-0.10847	0.604	0.55	1
Ewsr1	0.623843	-0.12207	0.579	0.527	1
Synrg	0.649518	-0.13938	0.313	0.303	1
Ube2m	0.653156	-0.12714	0.372	0.361	1
Dpm1	0.704035	-0.1157	0.309	0.295	1
Dnajc21	0.715759	-0.10498	0.242	0.239	1
Pgls	0.724576	-0.11811	0.396	0.379	1
Skp1a	0.770258	-0.10649	0.502	0.462	1
Vbp1	0.788857	-0.10176	0.266	0.257	1
Uqcr10	0.793734	-0.10648	0.632	0.573	1
Npl	0.830979	-0.12467	0.425	0.402	1
Exoc3l4	0.902391	-0.10623	0.341	0.32	1
Gm26870	0.919942	-0.21437	0.122	0.123	1
Ly6a	0.92514	-0.10259	0.465	0.444	1
Hspb1	0.948648	-0.1616	0.415	0.4	1
Fabp4	0.952403	-0.14055	0.837	0.809	1
Cxcl1	0.986706	-0.10632	0.199	0.194	1
