# Supplementary material for: Transcriptomic characterization of Lonrf1 at the single-cell level under pathophysiological conditions
Source: J Biochem. 2023 Mar 8;173(6):459–69. doi: 10.1093/jb/mvad021 (PMC10226518; doi:10.1093/jb/mvad021)
Supplement: Web_Material_mvad021 [file web_material_mvad021.zip › Supplementary Table S4.pdf]

Supplementary Table S4

DEG LonFR1+vsLonRF1- in Tomhigh LSEC from NASH liver

	p_val	avg_log2F(	pct.1	pct.2	p_val_adj
Lonrf1	0	1.331928	1	0	0
Enc1	2.67E-08	0.129318	0.469	0.338	0.000486
Rnf157	9.11E-08	0.109939	0.358	0.247	0.001655
Tbc1d2b	3.13E-07	0.119866	0.474	0.353	0.005684
Cyb5r1	9.15E-07	0.103491	0.331	0.232	0.016627
Cttnbp2nl	1.54E-06	0.102172	0.619	0.487	0.027956
Ifit2	1.66E-06	0.107465	0.43	0.32	0.030086
Ddit3	1.92E-06	0.104132	0.333	0.237	0.034823
Cd34	2.56E-06	0.107943	0.246	0.166	0.046537
tdTomato	3.52E-06	-0.13642	1	1	0.06391
Baiap2	2.34E-05	0.125478	0.507	0.404	0.42594
Mindy2	6.97E-05	0.102358	0.617	0.503	1
Tmem230	9.29E-05	0.105087	0.374	0.289	1
Gna13	0.000131	0.103674	0.801	0.702	1
Zmym5	0.000139	0.105814	0.608	0.498	1
Hexim1	0.000197	0.102236	0.839	0.736	1
Dync1i2	0.000258	0.163321	0.786	0.689	1
Ints6	0.00082	0.102894	0.764	0.687	1
Adamts1	0.001274	0.128303	0.873	0.804	1
Cebpd	0.01839	0.106232	0.796	0.723	1
Odc1	0.020421	0.105634	0.462	0.401	1
Bc1	0.031344	0.120924	0.124	0.098	1
Rab14	0.069995	-0.10525	0.869	0.81	1
Psen2	0.127529	-0.10602	0.869	0.831	1
Galnt18	0.210168	-0.12014	0.525	0.498	1
Cdyl	0.224287	-0.10589	0.246	0.253	1
Tuba1a	0.231231	-0.1184	0.423	0.421	1
Sult1a1	0.287509	-0.1231	0.388	0.388	1
Gm12216	0.294831	-0.12072	0.337	0.339	1
Immt	0.327329	-0.14165	0.421	0.401	1
Ccdc152	0.346234	-0.29093	0.526	0.518	1
Aimp1	0.393237	-0.10551	0.398	0.386	1
Micu1	0.404405	-0.11004	0.27	0.263	1
Rab12	0.408927	-0.10218	0.684	0.647	1
Clu	0.506023	-0.14532	0.279	0.255	1
Myo9a	0.529624	-0.11155	0.355	0.337	1
Rdx	0.54009	-0.11249	0.54	0.507	1
9930111J2	0.558099	-0.10194	0.747	0.696	1
Scoc	0.577964	-0.10831	0.351	0.33	1
Abcc9	0.594393	-0.10275	0.473	0.46	1

Cct8	0.634202	-0.11242	0.419	0.395	1
Tceal8	0.784318	-0.10442	0.407	0.365	1
Pbx1	0.844483	-0.10239	0.477	0.428	1
