# Supplementary material for: Transcriptomic characterization of Lonrf1 at the single-cell level under pathophysiological conditions
Source: J Biochem. 2023 Mar 8;173(6):459–69. doi: 10.1093/jb/mvad021 (PMC10226518; doi:10.1093/jb/mvad021)
Supplement: Web_Material_mvad021 [file web_material_mvad021.zip › Supplementary Table S5.pdf]

Supplementary Table S5

DEG LonFR1+vsLonRF1- in Tomlow LSEC from NASH liver

	p_val	avg_log2F(	pct.1	pct.2	p_val_adj
Lonrf1	0	1.353777	1	0	0
Fbxl14	1.47E-07	0.136146	0.307	0.2	0.002678
Mgmt	7.52E-07	0.108122	0.204	0.125	0.013674
Tango6	2.02E-06	0.11609	0.171	0.101	0.036679
Casp8ap2	2.41E-06	0.106763	0.491	0.372	0.043722
Ptbp3	1.01E-05	0.130149	0.954	0.884	0.18341
Heatr5a	1.16E-05	0.111192	0.297	0.21	0.210088
Slc25a51	1.36E-05	0.106727	0.448	0.334	0.247544
Dnajc10	1.37E-05	0.121695	0.525	0.414	0.24878
Rnaseh2c	1.56E-05	0.109753	0.312	0.221	0.284216
Ubr3	1.63E-05	0.104523	0.424	0.321	0.295595
Tmf1	2.13E-05	0.109194	0.699	0.569	0.386768
Flrt1	7.29E-05	0.109925	0.409	0.309	1
Banf1	7.70E-05	0.118597	0.416	0.321	1
Ccnd1	8.16E-05	0.135886	0.688	0.581	1
Slc38a10	8.50E-05	0.107954	0.698	0.587	1
Atox1	8.97E-05	0.108885	0.817	0.701	1
Ckap2l	9.81E-05	0.156861	0.404	0.311	1
Casp3	0.000113	0.122174	0.358	0.27	1
Mlxip	0.000124	0.103035	0.608	0.494	1
P2rx4	0.000145	0.110097	0.56	0.452	1
Abcc9	0.000155	0.117519	0.55	0.444	1
Eif4enif1	0.000183	0.101285	0.474	0.377	1
Otud7b	0.000214	0.116905	0.404	0.318	1
Steap2	0.00022	0.11121	0.211	0.148	1
Mrpl52	0.000225	0.102998	0.82	0.69	1
N4bp1	0.00027	0.100446	0.562	0.454	1
Lyve1	0.000321	0.140752	0.986	0.964	1
Lrp10	0.000336	0.102824	0.776	0.676	1
Osgin1	0.000448	0.110992	0.211	0.15	1
Mink1	0.000456	0.100865	0.577	0.478	1
Anxa5	0.00055	0.104719	0.937	0.862	1
Flt4	0.000662	0.118254	0.983	0.958	1
H2-T23	0.000677	-0.13258	0.959	0.957	1
Snrpc	0.000728	0.103141	0.448	0.356	1
Itga6	0.000897	0.116915	0.377	0.294	1
Gatm	0.001083	0.107723	0.898	0.825	1
Fzd8	0.001137	0.137919	0.263	0.198	1
Irf2	0.001263	0.107594	0.654	0.555	1
Nrp2	0.001367	0.102603	0.949	0.875	1

Sin3a	0.001975	0.103736	0.341	0.271	1
Slc43a2	0.002838	0.110578	0.834	0.745	1
Rad23a	0.003487	0.102076	0.567	0.48	1
Dusp7	0.004217	0.102745	0.467	0.391	1
Gm26870	0.00498	0.309228	0.229	0.177	1
Sgk1	0.007069	0.100175	0.976	0.924	1
Hexim1	0.009753	0.102277	0.827	0.74	1
Ppp1r10	0.010343	0.106981	0.779	0.71	1
Esm1	0.010597	0.104739	0.224	0.177	1
Irf7	0.033239	-0.15236	0.53	0.54	1
Ccdc12	0.039737	-0.12211	0.533	0.537	1
Ighm	0.071448	-0.13519	0.098	0.122	1
Actn4	0.078837	-0.10292	0.885	0.843	1
Crim1	0.095629	-0.11749	0.861	0.824	1
Kank3	0.099809	-0.16625	0.452	0.45	1
Snx21	0.10438	-0.13476	0.272	0.286	1
Wdr89	0.112422	-0.10244	0.742	0.69	1
Heca	0.124823	-0.14497	0.285	0.297	1
Mrpl24	0.140802	-0.10951	0.39	0.396	1
Ly6a	0.155326	-0.16766	0.803	0.79	1
Pim1	0.16956	-0.13252	0.638	0.615	1
Rb1	0.171845	-0.11114	0.251	0.268	1
Als2cl	0.173032	-0.11737	0.168	0.183	1
Foxp1	0.179453	-0.11077	0.706	0.689	1
Psmb8	0.204034	-0.1084	0.535	0.523	1
Parp1	0.218778	-0.16585	0.297	0.302	1
Gspt1	0.223208	-0.10343	0.526	0.507	1
Ppp4r3a	0.255516	-0.12461	0.312	0.312	1
Lsm12	0.275246	-0.10243	0.277	0.287	1
Acbd5	0.290213	-0.10723	0.292	0.296	1
Cdk11b	0.290507	-0.11949	0.696	0.653	1
Ube2d1	0.293743	-0.10385	0.321	0.323	1
Nxn	0.321891	-0.10832	0.418	0.411	1
Ntn4	0.332133	-0.10947	0.891	0.858	1
Dnttip1	0.333072	-0.11737	0.248	0.249	1
Stx12	0.335721	-0.10808	0.465	0.457	1
Dcn	0.346072	-0.16779	0.192	0.171	1
Prr5l	0.353272	-0.10865	0.365	0.366	1
Ehmt1	0.358492	-0.11089	0.38	0.379	1
Golga3	0.376484	-0.11048	0.345	0.344	1
Elovl1	0.392352	-0.10693	0.297	0.299	1
Chic2	0.412431	-0.12055	0.61	0.564	1
Isg15	0.415168	-0.23038	0.572	0.537	1

Itm2c	0.418209	-0.10762	0.718	0.684	1
Zfp280d	0.447597	-0.10965	0.306	0.302	1
Gbp7	0.45361	-0.1153	0.557	0.532	1
Ogfr	0.485933	-0.1026	0.319	0.317	1
BC003331	0.501143	-0.10114	0.292	0.292	1
Cul5	0.501908	-0.11405	0.377	0.361	1
Chd8	0.526269	-0.1119	0.453	0.439	1
Ifit3	0.543443	-0.16743	0.385	0.346	1
2610507B1	0.568179	-0.10709	0.482	0.454	1
Prkcb	0.57706	-0.10862	0.265	0.26	1
Kansl1	0.613872	-0.1022	0.484	0.456	1
Ifi206	0.657534	-0.11936	0.173	0.173	1
Tnrc6a	0.683009	-0.11065	0.598	0.567	1
Rbm6	0.705191	-0.10618	0.486	0.447	1
Vcam1	0.716363	-0.10575	0.62	0.575	1
F830016B0	0.74202	-0.13681	0.343	0.332	1
Aebp2	0.759561	-0.10085	0.331	0.317	1
Kcnq1ot1	0.770575	-0.20274	0.324	0.301	1
Pard3b	0.861569	-0.10505	0.418	0.378	1
Ifit1	0.967311	-0.21557	0.323	0.306	1
Ccdc152	0.982908	-0.19577	0.504	0.472	1
Alb	0.984277	-0.1153	0.346	0.328	1
Cited2	0.991431	-0.11509	0.389	0.365	1
Igkc	1	-0.10529	0.289	0.277	1
