# Supplementary material for: Transcriptomic characterization of Lonrf1 at the single-cell level under pathophysiological conditions
Source: J Biochem. 2023 Mar 8;173(6):459–69. doi: 10.1093/jb/mvad021 (PMC10226518; doi:10.1093/jb/mvad021)
Supplement: Web_Material_mvad021 [file web_material_mvad021.zip › Supplementary Table S6.pdf]

Supplementary Table S6

DEG LonFR1+vsLonRF1- in Tomhigh Fibro from wound

	p_val	avg_log2F(	pct.1	pct.2	p_val_adj
Lonrf1	1.90E-161	0.69896	1	0.006	3.94E-157
Wnt10b	2.33E-16	0.183635	0.122	0.003	4.84E-12
Cpne5	2.45E-11	0.457766	0.183	0.026	5.10E-07
Sh2b2	2.82E-11	0.255285	0.22	0.039	5.87E-07
Hhip	6.61E-11	0.24421	0.134	0.013	1.37E-06
Tfap2c	7.14E-11	0.171941	0.146	0.016	1.48E-06
Adgrb2	8.25E-11	0.107089	0.122	0.01	1.71E-06
Dio3	8.92E-11	0.523996	0.159	0.02	1.86E-06
Tfap2a	9.75E-10	0.33792	0.159	0.023	2.03E-05
Slc26a7	1.08E-09	0.717559	0.159	0.023	2.24E-05
Gng3	2.98E-09	0.313199	0.28	0.08	6.19E-05
Wif1	3.28E-09	0.587073	0.22	0.048	6.82E-05
Cox6b2	8.02E-09	0.279833	0.244	0.063	0.000167
Zfp618	1.05E-08	0.143704	0.134	0.019	0.000218
Cecr2	1.31E-08	0.146755	0.134	0.019	0.000273
Ddx5	2.90E-08	0.585578	1	0.999	0.000602
Lef1	3.83E-08	0.429057	0.183	0.039	0.000797
Adgrl3	4.93E-08	0.185107	0.159	0.029	0.001024
St6galnac5	5.72E-08	0.260465	0.207	0.049	0.001189
Igfbp3	6.27E-08	2.339568	0.415	0.176	0.001304
Lepr	6.30E-08	0.654746	0.28	0.09	0.001309
Ier5	8.51E-08	0.764199	0.988	0.897	0.001769
Jund	1.80E-07	0.408048	1	1	0.003747
Notum	2.00E-07	0.640936	0.207	0.054	0.004166
Nes	2.35E-07	0.127871	0.134	0.023	0.00488
Slc5a3	2.79E-07	0.639327	0.61	0.354	0.005801
Ezr	3.08E-07	0.166233	0.268	0.086	0.006403
Serf2	3.74E-07	-0.44629	0.963	0.997	0.007779
Nkrf	4.50E-07	0.138103	0.183	0.045	0.009359
Pde3a	5.04E-07	0.280927	0.378	0.156	0.010478
Pdcd4	5.54E-07	0.366574	0.878	0.643	0.011523
Gli1	5.72E-07	0.226588	0.183	0.045	0.011897
Pappa2	5.88E-07	0.753907	0.22	0.067	0.012215
Tox4	8.10E-07	0.264075	0.695	0.425	0.016832
Epha4	8.39E-07	0.328952	0.244	0.079	0.01745
Wnt6	1.09E-06	0.208789	0.134	0.026	0.02261
Zbtb17	1.25E-06	0.156809	0.305	0.115	0.026085
Map3k6	1.32E-06	0.317703	0.439	0.205	0.027449
Pcdh17	1.34E-06	0.143972	0.146	0.032	0.02782
Fbl	1.48E-06	0.303524	0.744	0.507	0.030836

Fxyd4	2.08E-06	0.208935	0.341	0.143	0.043176
Mcrip1	2.09E-06	-0.35664	0.744	0.876	0.043532
Prlr	2.40E-06	1.514135	0.22	0.071	0.049838
Eps15l1	2.65E-06	0.197622	0.634	0.365	0.055073
Irs2	2.78E-06	0.478446	0.878	0.694	0.057823
Mcl1	2.79E-06	0.338085	0.988	0.968	0.058018
Top1	2.86E-06	0.503263	0.951	0.927	0.059462
Rap1b	3.34E-06	0.359086	0.988	0.921	0.069488
Gm49085	3.46E-06	0.209013	0.183	0.051	0.071893
Zfand5	3.48E-06	0.723209	1	0.962	0.072277
Gm26615	3.93E-06	0.386406	0.549	0.325	0.081646
Nr4a1	3.96E-06	0.551566	0.976	0.898	0.082386
Calu	3.97E-06	-0.34537	0.927	0.981	0.082498
Alyref	4.85E-06	0.390803	0.756	0.543	0.100783
Wdr26	5.07E-06	0.35486	0.963	0.828	0.105369
Flvcr1	5.24E-06	0.218987	0.402	0.191	0.108939
Marcksl1	5.26E-06	0.428967	0.72	0.492	0.109293
Cd24a	5.43E-06	0.553572	0.207	0.067	0.112836
Ptma	6.36E-06	0.597206	1	1	0.132179
Nfat5	6.39E-06	0.37386	0.963	0.913	0.132883
Zfp622	7.06E-06	0.183877	0.707	0.441	0.146849
Slc38a2	7.15E-06	0.580309	1	0.978	0.148658
Coch	7.28E-06	1.139406	0.305	0.125	0.151312
Sorbs1	7.42E-06	0.206758	0.317	0.137	0.154293
Plekhh2	7.65E-06	0.239466	0.341	0.146	0.159118
Ep300	7.74E-06	0.352069	0.805	0.575	0.160996
Erf	8.10E-06	0.381719	0.744	0.517	0.168454
Eif4a1	8.32E-06	0.600934	1	0.975	0.17294
Tpm2	8.48E-06	0.173283	0.268	0.102	0.176335
Bcl2	9.60E-06	0.470405	0.305	0.135	0.199512
Hnrnpa1	9.86E-06	0.391947	0.976	0.888	0.204942
Ahdc1	1.06E-05	0.474854	0.756	0.568	0.220962
Nasp	1.13E-05	0.482748	0.756	0.581	0.234728
Klf10	1.13E-05	0.719354	0.537	0.338	0.235319
Hsp90aa1	1.13E-05	0.722955	1	0.981	0.235361
Ubc	1.19E-05	0.607601	1	0.999	0.247325
Cd200	1.21E-05	0.243019	0.171	0.048	0.250978
Hs3st3b1	1.23E-05	0.158069	0.159	0.044	0.255586
Stk35	1.26E-05	0.227251	0.463	0.252	0.26291
Cebpb	1.30E-05	0.777768	1	0.991	0.269727
Runx3	1.35E-05	0.304678	0.317	0.14	0.279921
Phf13	1.35E-05	0.302621	0.573	0.357	0.280226
Irf8	1.49E-05	0.117296	0.11	0.022	0.310769

Serpine2	1.51E-05	1.360779	0.354	0.179	0.31408
Dnaja1	1.61E-05	0.683408	1	0.939	0.334385
Dnajb1	1.64E-05	0.414403	0.866	0.703	0.341755
Sltm	1.69E-05	0.308852	0.768	0.559	0.3508
Ubash3b	1.75E-05	0.207555	0.171	0.049	0.364099
Kmt2c	1.82E-05	0.295359	0.854	0.635	0.378324
N4bp3	1.86E-05	0.111322	0.146	0.038	0.387353
Btg1	1.91E-05	0.381547	0.988	0.99	0.397238
Pim1	2.13E-05	0.634977	0.963	0.895	0.442428
Neat1	2.13E-05	0.508617	0.976	0.945	0.442616
Carmn	2.15E-05	0.244728	0.183	0.058	0.446481
Htra1	2.21E-05	-0.37926	0.927	0.984	0.459038
Fosl1	2.25E-05	0.291439	0.488	0.271	0.467983
Kdm5c	2.27E-05	0.165118	0.646	0.387	0.472109
Tnfrsf12a	2.47E-05	0.718427	0.89	0.735	0.514014
Daam2	2.50E-05	0.309601	0.268	0.111	0.520128
Cycs	2.69E-05	0.27627	0.756	0.524	0.559803
Rbm4b	2.81E-05	0.555148	0.524	0.349	0.584353
mt-Nd4	2.86E-05	-0.26626	1	0.999	0.594573
H3f3b	2.96E-05	0.400025	0.988	1	0.615565
Lmna	3.04E-05	0.438478	1	0.996	0.632864
Tmem176a	3.07E-05	0.202608	0.256	0.1	0.639175
Son	3.13E-05	0.497819	0.988	0.933	0.650769
Kalrn	3.31E-05	0.139506	0.183	0.06	0.687599
Arid1a	3.34E-05	0.368797	0.902	0.75	0.695138
Dnmt3a	3.71E-05	0.207702	0.695	0.444	0.771738
Ube2s	3.73E-05	0.605113	0.976	0.885	0.774445
Hspa5	3.74E-05	0.519439	1	0.996	0.778137
Ikzf4	3.76E-05	0.139946	0.195	0.064	0.781534
Zcchc18	3.79E-05	0.170045	0.134	0.035	0.78696
Zmym4	4.04E-05	0.20776	0.537	0.313	0.839326
Ankrd28	4.07E-05	0.230578	0.671	0.44	0.845771
Ubb	4.16E-05	0.44612	1	1	0.864106
Dnajb9	4.35E-05	0.511563	0.927	0.82	0.904647
Ube2e1	4.52E-05	0.297575	0.695	0.563	0.938702
Tob1	4.57E-05	0.589808	0.939	0.82	0.949729
Stat3	4.63E-05	0.471606	0.963	0.879	0.962472
Mknk2	4.76E-05	0.239041	0.878	0.696	0.99025
Eva1a	5.22E-05	0.161791	0.146	0.042	1
Gem	5.35E-05	0.60028	0.915	0.846	1
Nsun2	5.51E-05	0.158429	0.524	0.311	1
Ash1l	5.68E-05	0.399203	0.927	0.9	1
Prrc2a	6.09E-05	0.317095	0.866	0.726	1

Setd5	6.17E-05	0.282474	0.939	0.728	1
Eif4a2	6.62E-05	0.306165	0.951	0.86	1
Znrf1	6.66E-05	0.236165	0.463	0.274	1
Sbno1	7.22E-05	0.404819	0.866	0.716	1
Casp3	7.34E-05	0.213267	0.549	0.332	1
Vps37b	7.49E-05	0.152126	0.256	0.105	1
Pcolce	7.50E-05	-0.34561	0.939	0.98	1
Tenm3	7.86E-05	0.231815	0.207	0.076	1
1810058I2	8.13E-05	-0.35721	0.817	0.92	1
Mcmbp	8.20E-05	0.221263	0.61	0.371	1
Diaph1	8.50E-05	0.246084	0.89	0.71	1
Ccnl2	8.59E-05	0.248476	0.854	0.696	1
Hal	8.76E-05	0.105953	0.11	0.026	1
Lcn2	8.89E-05	0.152508	0.134	0.038	1
Fam114a1	9.43E-05	-0.29631	0.646	0.796	1
Usp7	9.43E-05	0.315609	0.805	0.678	1
Itsn2	9.86E-05	0.165035	0.61	0.384	1
Etf1	9.86E-05	0.266122	0.89	0.801	1
Tuba4a	0.0001	0.23566	0.317	0.154	1
Ing2	0.000107	0.316368	0.524	0.323	1
Tsc22d1	0.000107	0.488323	0.854	0.71	1
Ndnf	0.00011	0.240616	0.22	0.086	1
Nop56	0.00011	0.293143	0.744	0.546	1
mt-Co2	0.000111	-0.23443	1	1	1
Mkln1	0.000113	0.331322	0.854	0.656	1
Egr3	0.000115	0.313278	0.317	0.154	1
Ywhaz	0.000116	0.336483	0.963	0.932	1
Ifrd1	0.000116	0.31955	0.976	0.913	1
Junb	0.000119	0.811628	0.988	0.99	1
Nup50	0.000121	0.207031	0.585	0.378	1
Rbm15b	0.000123	0.306514	0.524	0.342	1
Arl4a	0.000127	0.393478	0.805	0.64	1
Trmt10a	0.000128	0.169635	0.293	0.14	1
Creb3l1	0.000128	-0.29753	0.866	0.936	1
Midn	0.000128	0.501636	0.915	0.854	1
Fbxl18	0.000131	0.21641	0.427	0.249	1
Stox2	0.000133	0.181797	0.5	0.294	1
Lman2l	0.000134	0.129602	0.232	0.098	1
Khdrbs1	0.000134	0.275933	0.854	0.702	1
Tra2b	0.000142	0.479051	0.951	0.886	1
Kctd1	0.000143	0.139824	0.134	0.039	1
Ptgir	0.000144	0.167896	0.159	0.052	1
Pi4k2a	0.000145	0.303018	0.732	0.575	1

Rexo1	0.000149	0.322067	0.537	0.361	1
Fzd8	0.000151	0.127686	0.305	0.144	1
Etv1	0.000155	0.139663	0.171	0.058	1
Hnrnpa2b1	0.000157	0.494071	0.988	0.965	1
Mex3a	0.000157	0.259453	0.305	0.151	1
Wsb1	0.000158	0.406049	0.988	0.934	1
Rbm39	0.000163	0.428994	0.988	0.971	1
Vegfa	0.000163	0.484021	0.732	0.595	1
Hnrnph1	0.000166	0.466455	1	0.961	1
Sesn2	0.000173	0.156871	0.378	0.197	1
Gm26532	0.000173	0.374196	0.573	0.394	1
Ptbp3	0.000175	0.35592	0.805	0.677	1
Ipmk	0.000176	0.252391	0.537	0.345	1
Suco	0.000182	0.251351	0.671	0.489	1
mt-Co3	0.000183	-0.19787	1	1	1
Emsy	0.000188	0.245501	0.646	0.429	1
Sp1	0.000189	0.212074	0.707	0.533	1
Vkorc1	0.000196	-0.27719	0.927	0.939	1
Hspa2	0.000216	0.508891	0.512	0.342	1
Nsd3	0.000217	0.36594	0.951	0.836	1
Ankrd11	0.000218	0.318928	0.927	0.831	1
Pcf11	0.00022	0.370496	0.878	0.737	1
Hspa8	0.000225	0.552761	1	0.999	1
Pabpc1	0.000227	0.28667	0.988	0.971	1
Yod1	0.000229	0.261527	0.512	0.326	1
Bgn	0.000236	-0.59553	0.963	0.993	1
Srsf10	0.000237	0.324287	0.805	0.636	1
Bmp4	0.00024	0.795628	0.634	0.453	1
Map3k1	0.000249	0.156099	0.39	0.221	1
Sertad2	0.000251	0.538766	0.744	0.626	1
Rspo3	0.000255	0.185657	0.159	0.054	1
Tgif2	0.000259	0.284262	0.244	0.115	1
Tmem176b	0.000264	0.164044	0.317	0.156	1
Ccnl1	0.000267	0.503403	1	0.953	1
Cavin3	0.000269	-0.31472	0.927	0.961	1
Stk40	0.00027	0.324353	0.756	0.607	1
Kdm2a	0.000277	0.325742	0.841	0.713	1
Ddhd1	0.000278	0.246911	0.671	0.476	1
Pofut2	0.000281	-0.26154	0.756	0.84	1
Tcp11l2	0.00029	0.266694	0.78	0.591	1
Hnrnpl	0.000294	0.486812	0.878	0.799	1
Nfkbia	0.000296	0.362048	1	0.987	1
Col13a1	0.000296	0.359274	0.183	0.071	1

Scube1	0.000297	0.136274	0.134	0.041	1
Phlpp1	0.000299	0.150439	0.415	0.226	1
Calm2	0.000299	0.317275	1	0.994	1
Rpl27	0.000302	0.244225	0.976	0.917	1
Piezo1	0.000304	0.240822	0.683	0.53	1
Arih1	0.000305	0.339159	0.89	0.792	1
Nufip2	0.000307	0.409862	0.939	0.86	1
Irf2	0.000316	0.401218	0.683	0.533	1
Il31ra	0.000317	0.121046	0.244	0.106	1
Papd5	0.000317	0.160212	0.585	0.383	1
Pbx3	0.000317	0.268597	0.305	0.159	1
Ube2o	0.000321	0.182025	0.561	0.37	1
Chka	0.000321	0.687433	0.817	0.696	1
Klf13	0.000323	0.605737	0.854	0.741	1
Rc3h1	0.000326	0.2216	0.89	0.731	1
Trak2	0.000329	0.117327	0.524	0.31	1
Gpbp1	0.000331	0.361774	0.805	0.667	1
Naa15	0.000333	0.308434	0.768	0.6	1
Dap	0.000336	-0.29656	0.695	0.859	1
Matr3	0.000346	0.28665	0.902	0.801	1
Serpinh1	0.000348	-0.31734	0.988	0.999	1
Pnrc1	0.00035	0.330303	0.976	0.959	1
Slc6a6	0.000353	0.75294	0.524	0.365	1
Rnf19a	0.000353	0.288517	0.732	0.574	1
Kdelr2	0.000353	-0.30151	0.963	0.983	1
Zfp655	0.000356	0.199643	0.573	0.387	1
Notch1	0.000367	0.263069	0.268	0.13	1
Ubr5	0.000368	0.25836	0.854	0.665	1
Adgrl1	0.000375	0.137298	0.305	0.147	1
Ilf2	0.000378	0.281244	0.72	0.527	1
Rcn1	0.000379	-0.31108	0.732	0.884	1
Coq10b	0.000379	0.345529	0.841	0.675	1
Ep400	0.000381	0.338808	0.744	0.624	1
Rbm18	0.000383	0.23082	0.695	0.509	1
mt-Co1	0.000402	-0.19291	1	1	1
Dusp5	0.000402	0.176559	0.561	0.368	1
Mlxip	0.000403	0.220897	0.732	0.525	1
Dynll2	0.000406	0.256132	0.854	0.687	1
Srrm2	0.000407	0.362197	0.976	0.923	1
Eln	0.000409	-0.51696	0.841	0.907	1
Syvn1	0.000417	0.160011	0.427	0.266	1
Pam	0.000431	-0.37003	0.805	0.907	1
Tgif1	0.000433	0.220191	0.78	0.623	1

Rbm25	0.000433	0.339499	0.951	0.882	1
H3f3a	0.000434	0.281631	1	1	1
Col5a1	0.000434	-0.22496	0.927	0.985	1
Fbxl7	0.000435	0.208504	0.305	0.156	1
Ythdc1	0.000436	0.274611	0.756	0.614	1
Brwd3	0.000461	0.1478	0.451	0.269	1
Cdc14a	0.000466	0.180368	0.232	0.105	1
Washc3	0.000475	-0.21583	0.341	0.517	1
Birc6	0.00048	0.225353	0.878	0.739	1
Fbxl14	0.000485	0.150863	0.463	0.263	1
Ythdc2	0.000491	0.137191	0.317	0.164	1
Mafk	0.000492	0.213055	0.646	0.504	1
Flt1	0.000513	0.127383	0.122	0.038	1
Rest	0.000528	0.321422	0.707	0.549	1
Sparc	0.000533	-0.40376	1	1	1
Cotl1	0.000556	0.140475	0.268	0.127	1
Sobp	0.000557	0.42111	0.341	0.191	1
Igf1r	0.000559	0.314634	0.915	0.825	1
Usp10	0.000559	0.190134	0.427	0.262	1
Larp1b	0.000568	0.168601	0.476	0.29	1
Cfl1	0.00057	0.425478	1	0.978	1
Baz1a	0.000572	0.26359	0.756	0.581	1
Ndel1	0.000579	0.244161	0.939	0.846	1
Adh7	0.000586	-0.38069	0.805	0.907	1
Vps13a	0.00059	0.198554	0.537	0.351	1
Ldlr	0.000602	0.274128	0.451	0.275	1
Csrnp1	0.000606	0.268949	0.927	0.815	1
1810013L2	0.000606	0.203765	0.72	0.559	1
Marveld1	0.000609	-0.33575	0.805	0.869	1
Setbp1	0.000614	0.164708	0.707	0.495	1
Zfc3h1	0.000625	0.188487	0.671	0.507	1
Hivep2	0.000628	0.312963	0.866	0.728	1
Slc16a10	0.000628	0.209615	0.488	0.333	1
Ptpn12	0.000634	0.237847	0.72	0.52	1
1700017B0	0.000638	0.327049	0.402	0.262	1
Safb2	0.000639	0.352189	0.695	0.514	1
mt-Nd1	0.000641	-0.23805	1	1	1
Irf2bp2	0.000654	0.253397	0.951	0.849	1
Map7d1	0.000659	0.298661	0.939	0.795	1
Sf3b1	0.00066	0.293951	0.951	0.904	1
2610037D0	0.000664	0.19081	0.317	0.167	1
Klf9	0.000675	0.357853	0.988	0.962	1
Arfgef1	0.000675	0.197108	0.805	0.604	1

Akap11	0.00068	0.191111	0.549	0.371	1
Rnps1	0.000681	0.261928	0.659	0.486	1
Kpna2	0.000692	0.157489	0.207	0.093	1
Npr2	0.0007	0.206763	0.646	0.464	1
Gjc1	0.000709	0.143885	0.207	0.09	1
Gdf11	0.00071	0.123891	0.256	0.122	1
Epop	0.000712	0.181589	0.122	0.039	1
Elf1	0.000717	0.276364	0.89	0.78	1
Kdm7a	0.000726	0.475388	0.854	0.786	1
Ppp1r10	0.000756	0.592752	0.854	0.734	1
Rasd1	0.000757	0.660995	0.512	0.368	1
Peli1	0.00076	0.283869	0.854	0.76	1
Rbbp6	0.000769	0.399527	0.902	0.878	1
Adh1	0.000776	-0.40579	0.78	0.885	1
Myrip	0.000795	0.105384	0.22	0.099	1
Zbtb10	0.000799	0.273555	0.707	0.55	1
Csnk1a1	0.000807	0.219414	0.976	0.948	1
Agpat3	0.000809	-0.20627	0.427	0.595	1
Srsf2	0.000816	0.324308	0.988	0.959	1
Ctdspl	0.000821	0.505316	0.463	0.3	1
Cd63	0.000825	-0.24351	1	0.999	1
Naa35	0.000829	0.149027	0.488	0.311	1
Kat6b	0.000829	0.180602	0.573	0.367	1
Oser1	0.000832	0.2295	0.683	0.507	1
Shox2	0.000833	0.258002	0.683	0.493	1
Srcin1	0.000833	0.161347	0.171	0.067	1
Btbd10	0.000834	0.12882	0.5	0.304	1
Eif5	0.000835	0.344987	0.988	0.971	1
Tuba1c	0.000886	0.471042	0.512	0.376	1
Foxn3	0.000894	0.230877	0.902	0.787	1
Nup214	0.000897	0.178277	0.402	0.237	1
Tmem41a	0.000907	0.105454	0.329	0.163	1
Gm26669	0.000909	0.210269	0.293	0.146	1
Bhlhe40	0.000965	0.412308	0.793	0.64	1
G2e3	0.000976	0.122962	0.329	0.176	1
Vasn	0.00098	0.327048	0.902	0.854	1
Maff	0.000991	0.482494	0.72	0.574	1
Ints6l	0.000992	0.258025	0.488	0.306	1
Grem2	0.000993	-0.37086	0.28	0.435	1
Zbtb7a	0.000996	0.541862	0.927	0.828	1
Ubxn2a	0.000997	0.172295	0.378	0.226	1
Nr1d2	0.000999	0.295892	0.695	0.54	1
Dusp10	0.00101	0.134151	0.622	0.415	1

Smap1	0.001013	-0.21921	0.573	0.702	1
Smdt1	0.001026	-0.24015	0.817	0.85	1
Mob1b	0.00103	0.192613	0.683	0.509	1
Sf3b3	0.001034	0.173197	0.634	0.48	1
Meg3	0.00104	-0.47125	0.902	0.956	1
Sfmbt1	0.001044	0.143247	0.366	0.205	1
Trp53	0.001058	0.34568	0.841	0.699	1
Lars2	0.001074	-0.80933	0.756	0.834	1
Ankrd17	0.001084	0.400595	0.927	0.84	1
Arf5	0.001093	-0.22075	0.902	0.967	1
Rbm33	0.001107	0.190838	0.598	0.424	1
Taf1	0.00112	0.182091	0.646	0.435	1
Kmt2e	0.001125	0.340365	0.963	0.87	1
Zfp36	0.001148	0.335916	0.988	0.972	1
Mfsd14a	0.001148	0.161527	0.537	0.36	1
Lima1	0.001154	-0.30906	0.622	0.747	1
Col3a1	0.001162	-0.31346	1	1	1
Chd4	0.001165	0.299332	0.963	0.911	1
Angptl2	0.001171	-0.34206	0.72	0.847	1
Fbxw7	0.001192	0.199186	0.427	0.268	1
Cfh	0.001213	0.153582	0.122	0.041	1
Chd1	0.001215	0.288758	0.671	0.527	1
Rarres2	0.001221	-0.26636	0.878	0.984	1
Bptf	0.001244	0.303381	0.951	0.828	1
Klf5	0.001251	0.274179	0.378	0.231	1
Egr2	0.001274	0.229969	0.402	0.24	1
Psd3	0.001284	0.162276	0.512	0.357	1
Klf16	0.001297	0.208468	0.415	0.258	1
B230369F2	0.001298	0.236446	0.341	0.192	1
Cdkn1a	0.00132	0.492905	0.915	0.862	1
Cyp51	0.001326	0.1183	0.402	0.237	1
Hnrnpab	0.001334	0.330618	0.939	0.898	1
Erp44	0.001335	-0.25113	0.683	0.732	1
Tob2	0.001335	0.550874	0.878	0.825	1
Tra2a	0.001341	0.435149	0.817	0.712	1
Dkk2	0.001342	0.447245	0.378	0.227	1
Ctsh	0.001357	-0.30939	0.89	0.965	1
Trrap	0.001359	0.218158	0.659	0.508	1
Syde2	0.001384	0.127848	0.366	0.205	1
Rcn3	0.001401	-0.27909	0.951	0.977	1
Epas1	0.001404	0.143753	0.244	0.119	1
Mllt6	0.001423	0.220802	0.561	0.378	1
Snrnp70	0.001434	0.273523	0.927	0.833	1

Sqle	0.001439	0.137869	0.244	0.121	1
Azin1	0.001445	0.346934	0.866	0.745	1
Eif1a	0.001464	0.379651	0.78	0.617	1
Pum1	0.001477	0.29478	0.866	0.755	1
Fbrs	0.001496	0.140265	0.488	0.328	1
Trim28	0.001507	0.242332	0.793	0.652	1
Tmem218	0.00153	0.114889	0.439	0.268	1
Skil	0.001542	0.394684	0.61	0.507	1
Rassf1	0.001542	0.247786	0.793	0.662	1
Mmp2	0.001545	-0.28857	1	0.993	1
Npc2	0.001549	-0.2491	0.976	0.991	1
Ckb	0.00155	0.294112	0.768	0.636	1
Med13	0.001573	0.209613	0.951	0.844	1
Tln2	0.001583	0.187384	0.305	0.17	1
Ist1	0.001607	0.212824	0.768	0.595	1
Kpna4	0.001617	0.205338	0.805	0.662	1
Lgr4	0.001622	0.16939	0.354	0.213	1
Gatad2b	0.001629	0.413723	0.841	0.706	1
Usp34	0.001642	0.217585	0.841	0.764	1
Ndst1	0.001648	0.218476	0.585	0.425	1
Tnrc6c	0.001673	0.205066	0.854	0.7	1
Fosl2	0.00172	0.413179	0.878	0.792	1
Atp5e	0.001735	-0.21578	0.951	0.964	1
Taf1d	0.001763	0.417376	0.793	0.659	1
Chchd2	0.001764	0.294023	1	0.983	1
Lgals7	0.001793	0.340562	0.341	0.21	1
Adgre5	0.001799	0.101371	0.207	0.095	1
Ccnd3	0.001799	0.517573	0.585	0.467	1
Dnm1l	0.001807	0.125397	0.585	0.387	1
Eif4e	0.001817	0.217892	0.878	0.766	1
Loxl2	0.001821	-0.22962	0.829	0.929	1
Actg1	0.001829	0.419502	1	0.997	1
Slc3a2	0.001832	0.343408	0.878	0.787	1
Kansl1	0.00185	0.22267	0.805	0.64	1
Itpkb	0.001865	0.196522	0.537	0.358	1
Frat2	0.001868	0.160107	0.305	0.173	1
Luzp1	0.001885	0.247727	0.756	0.6	1
Ndufb7	0.001901	-0.22365	0.646	0.735	1
Trib1	0.00191	0.250232	0.854	0.712	1
Brox	0.001928	0.150977	0.549	0.367	1
Brpf1	0.00194	0.123174	0.341	0.194	1
Brd4	0.001942	0.294065	0.866	0.767	1
Ppp2r2a	0.001963	0.304403	0.805	0.689	1

Ppp2ca	0.002051	0.17298	0.976	0.939	1
Il17ra	0.002057	0.189525	0.793	0.598	1
Ank3	0.002062	0.108447	0.11	0.036	1
Gnl3	0.002076	0.205236	0.695	0.549	1
Nup62	0.002077	0.190755	0.427	0.29	1
Acaa1a	0.002085	0.160308	0.768	0.569	1
Rhob	0.002116	0.404018	0.915	0.865	1
Slbp	0.002121	0.246631	0.671	0.518	1
Olfml3	0.002132	-0.29577	0.878	0.93	1
Trip12	0.002136	0.208867	0.878	0.824	1
Runx1	0.002154	0.415113	0.524	0.383	1
Arhgdia	0.002162	-0.24737	0.829	0.894	1
Hspb1	0.002174	1.138821	0.39	0.263	1
Gpbp1l1	0.002186	0.155959	0.561	0.36	1
Atox1	0.002186	-0.22343	0.902	0.926	1
Rnf103	0.002188	0.176797	0.585	0.4	1
Srpk1	0.002195	0.178963	0.61	0.443	1
Stac2	0.0022	0.164646	0.317	0.176	1
Ctsb	0.002217	-0.25933	0.951	0.997	1
Btbd7	0.002264	0.209656	0.829	0.721	1
Hmga1	0.00228	0.121935	0.183	0.08	1
Smad7	0.002288	0.463887	0.878	0.728	1
Sh3bgrl3	0.002313	-0.33506	0.756	0.787	1
Ccdc82	0.002316	0.143277	0.427	0.256	1
Cct7	0.002324	0.164767	0.841	0.684	1
Ankrd10	0.00233	0.185344	0.573	0.408	1
Fbxl22	0.002361	0.131682	0.244	0.125	1
Wnk1	0.002368	0.312206	0.963	0.93	1
Pou3f1	0.002389	0.122004	0.293	0.164	1
Cdk11b	0.002419	0.178066	0.72	0.524	1
Mmp14	0.002447	-0.36007	0.976	0.994	1
Sema3b	0.002468	-0.24085	0.634	0.799	1
Tmem167	0.002494	-0.29768	0.78	0.884	1
Slc2a13	0.00251	0.126006	0.268	0.144	1
Derl1	0.002537	-0.2659	0.78	0.836	1
mt-Cytb	0.00254	-0.26102	1	1	1
Jak1	0.002568	0.228521	0.927	0.878	1
Ahcyl2	0.002588	0.217036	0.561	0.399	1
Papd7	0.002592	0.183533	0.476	0.341	1
Ifitm1	0.002602	0.404135	0.366	0.227	1
Caskin2	0.002643	0.106171	0.354	0.217	1
Zbtb2	0.002664	0.287358	0.537	0.402	1
Zfp131	0.002732	0.580272	0.732	0.636	1

Nr1d1	0.002787	0.257768	0.5	0.346	1
Chkb	0.002798	0.194017	0.488	0.326	1
Apc	0.002805	0.181481	0.622	0.477	1
Fgfr1	0.002854	0.323944	0.878	0.776	1
Enc1	0.002902	-0.27092	0.415	0.547	1
Tbrg1	0.002936	-0.23186	0.744	0.836	1
Slc2a1	0.002961	0.218195	0.341	0.21	1
Gm17056	0.002963	0.171243	0.427	0.274	1
Timp2	0.002979	-0.26264	1	0.997	1
mt-Atp6	0.003057	-0.1841	1	1	1
Edf1	0.003092	-0.2331	0.78	0.854	1
4921524J1	0.003098	0.144232	0.524	0.371	1
Nab2	0.003105	0.442149	0.549	0.413	1
Cbx4	0.003156	0.249277	0.512	0.355	1
Sqstm1	0.003172	0.305988	0.951	0.895	1
Ggnbp2	0.003233	0.366392	0.841	0.755	1
Nenf	0.003262	-0.2242	0.927	0.961	1
Abl1	0.003263	0.162403	0.805	0.642	1
Brwd1	0.003341	0.16775	0.72	0.541	1
Cab39	0.003393	0.176887	0.671	0.523	1
Dalrd3	0.003394	0.108169	0.354	0.217	1
Hsd11b1	0.003417	0.426578	0.293	0.163	1
Snhg12	0.003448	0.148202	0.756	0.56	1
Zbtb11	0.003468	0.300541	0.573	0.419	1
Gm26545	0.003478	0.11056	0.207	0.105	1
Mob3c	0.003481	0.112579	0.524	0.349	1
Gpx1	0.003499	0.391729	0.951	0.949	1
Exoc6b	0.003525	0.293816	0.61	0.438	1
Cdkn2d	0.003545	0.119291	0.378	0.234	1
Tfrc	0.003564	0.173446	0.207	0.105	1
Fbn1	0.003567	-0.3027	0.951	0.994	1
Crabp2	0.003611	0.368122	0.232	0.13	1
Arid2	0.003626	0.234487	0.72	0.549	1
Zfp653	0.003639	0.123617	0.22	0.111	1
Ahctf1	0.003654	0.1821	0.646	0.514	1
Tfdp2	0.00366	0.107105	0.415	0.259	1
Ndufa13	0.003746	-0.19826	0.976	0.951	1
Atf5	0.003757	-0.2644	0.866	0.913	1
Brd1	0.003812	0.244414	0.744	0.633	1
Slc27a3	0.003819	0.114552	0.28	0.157	1
Gnl2	0.003852	0.302203	0.488	0.328	1
Zfp871	0.00386	0.156573	0.463	0.304	1
Trim16	0.003894	0.160286	0.451	0.298	1

Brd2	0.003895	0.516769	0.976	0.94	1
Pprc1	0.003942	0.100802	0.451	0.294	1
Kmt2d	0.003994	0.259118	0.598	0.453	1
Ccdc88a	0.004025	0.206192	0.707	0.572	1
Txndc5	0.004037	-0.19963	0.829	0.866	1
Gmeb2	0.004051	0.122073	0.293	0.17	1
Gm37494	0.00407	0.197428	0.354	0.217	1
Cebpa	0.004079	0.15437	0.354	0.224	1
Kif5b	0.004082	0.201418	0.951	0.86	1
Ric1	0.004124	0.234497	0.622	0.464	1
Lamtor2	0.004132	-0.21238	0.707	0.783	1
Sf3b5	0.004147	-0.21764	0.598	0.747	1
Mrps18a	0.004165	-0.15404	0.488	0.606	1
Rfxap	0.004189	0.108972	0.427	0.29	1
Ctnna1	0.004202	0.199736	0.854	0.748	1
Sms	0.004222	0.115266	0.232	0.121	1
Atn1	0.004235	0.1576	0.585	0.443	1
Rpain	0.004273	0.169392	0.402	0.261	1
Gm26523	0.004287	0.232851	0.378	0.246	1
H2afz	0.004312	0.517442	0.963	0.942	1
Cdon	0.004318	-0.27938	0.354	0.507	1
Srrm1	0.004332	0.171009	0.89	0.755	1
Kdm6a	0.004355	0.135715	0.585	0.426	1
Riok2	0.004359	0.12483	0.329	0.194	1
Saraf	0.004385	0.159665	0.854	0.723	1
Gli2	0.004402	0.147842	0.293	0.164	1
Hbegf	0.004409	0.224321	0.354	0.229	1
Copz2	0.004456	-0.26178	0.732	0.9	1
Ogt	0.004457	0.16481	0.659	0.504	1
Dhx30	0.004464	0.223116	0.5	0.345	1
Bri3	0.004552	-0.21502	0.915	0.942	1
Polr2m	0.00459	-0.18403	0.488	0.623	1
Cxcl16	0.004591	-0.24271	0.293	0.44	1
Aldh6a1	0.004609	0.131893	0.5	0.351	1
Kpna1	0.004629	0.172835	0.549	0.371	1
Sdc4	0.00468	0.493205	0.939	0.882	1
Bcl2l11	0.004683	0.185093	0.549	0.396	1
Rapgef6	0.004684	0.141405	0.634	0.46	1
Il17rd	0.004699	0.109368	0.378	0.236	1
Zfp644	0.004707	0.27692	0.72	0.556	1
Ndrg2	0.004735	0.195183	0.317	0.202	1
Il1rl2	0.004778	-0.20955	0.634	0.739	1
Ift20	0.004816	-0.25632	0.878	0.916	1

Dusp2	0.004818	0.666344	0.244	0.135	1
Abi3bp	0.004825	-0.36156	0.841	0.916	1
Rflnb	0.004826	0.13569	0.317	0.183	1
Kmt5b	0.004853	0.23353	0.72	0.584	1
Hnrnpdl	0.004863	0.325	0.89	0.84	1
Mbtd1	0.004865	0.31625	0.72	0.6	1
Ccnt1	0.004875	0.16424	0.695	0.517	1
Patz1	0.004894	0.108743	0.195	0.095	1
P4ha2	0.004916	-0.24514	0.598	0.725	1
P4hb	0.00495	-0.2129	0.915	0.975	1
Pik3r3	0.00499	0.1469	0.22	0.115	1
Stx3	0.00501	0.145476	0.39	0.242	1
Arid4b	0.00502	0.280883	0.866	0.815	1
Elavl1	0.00505	0.175313	0.878	0.729	1
Mfap2	0.005077	-0.22933	0.927	0.98	1
Col6a2	0.005113	-0.48257	0.878	0.902	1
Hexim1	0.005134	0.455303	0.793	0.709	1
Ostc	0.005136	-0.23672	0.854	0.917	1
Rtl8b	0.005143	0.137065	0.476	0.32	1
Siva1	0.005167	-0.23823	0.537	0.643	1
Arid3a	0.005176	0.162889	0.268	0.156	1
Hnrnpll	0.005246	0.172595	0.695	0.537	1
Kdm5a	0.005261	0.226902	0.854	0.741	1
Adrm1	0.005298	0.12954	0.659	0.533	1
Rictor	0.005323	0.212899	0.671	0.533	1
Eva1b	0.005377	-0.2342	0.829	0.834	1
Cdc42bpb	0.005388	0.133547	0.549	0.403	1
Ralgds	0.00541	0.127831	0.598	0.426	1
Trpm7	0.005411	0.167911	0.866	0.732	1
Hadh	0.005419	0.172048	0.561	0.412	1
Rnf44	0.005462	0.107984	0.5	0.336	1
Hnrnpu	0.005507	0.376165	0.939	0.916	1
Mark4	0.005533	0.110891	0.463	0.304	1
Tnxb	0.005578	-0.23426	0.829	0.937	1
Med12	0.005616	0.137989	0.463	0.325	1
Alkbh5	0.005636	0.275587	0.902	0.786	1
Kdelr1	0.005643	-0.23683	0.878	0.924	1
Txnrd1	0.005652	0.216892	0.732	0.598	1
Pknox1	0.005674	0.128463	0.415	0.274	1
Zdhhc3	0.005685	-0.19138	0.366	0.511	1
Usp19	0.005709	0.161153	0.659	0.493	1
Rgs5	0.00573	0.167434	0.122	0.047	1
Lipa	0.005756	0.133654	0.537	0.378	1

Bclaf1	0.005756	0.221452	0.902	0.755	1
Sod3	0.005766	-0.29677	0.634	0.742	1
Fam76b	0.005771	0.101651	0.329	0.194	1
Mapre1	0.005859	0.288296	0.976	0.892	1
Ift52	0.005862	-0.1295	0.122	0.253	1
Wwc2	0.0059	0.384991	0.866	0.705	1
Fstl3	0.005904	0.317335	0.598	0.466	1
Entpd2	0.005927	-0.2477	0.744	0.836	1
Inhba	0.005946	0.477421	0.268	0.162	1
Rnf111	0.005949	0.124956	0.549	0.389	1
Vgll4	0.00595	0.13411	0.439	0.306	1
Crtc3	0.005988	0.170099	0.744	0.584	1
Fnbp4	0.006	0.245446	0.5	0.346	1
Ssbp2	0.00604	0.233061	0.768	0.626	1
Pqlc1	0.006047	0.123543	0.512	0.344	1
Srebf2	0.006061	0.272353	0.61	0.453	1
Tmem160	0.006066	-0.20051	0.585	0.703	1
Ddx54	0.006115	0.115495	0.427	0.279	1
Vim	0.006129	0.24444	1	1	1
Desi2	0.006162	0.203178	0.732	0.588	1
Ddx21	0.006225	0.270662	0.671	0.524	1
Cadm3	0.006246	-0.24218	0.585	0.67	1
Vezf1	0.006249	0.211242	0.878	0.789	1
Tiam2	0.006271	0.126495	0.183	0.092	1
Ppl	0.006278	0.226858	0.488	0.358	1
Osr1	0.006318	-0.1645	0.122	0.253	1
Ftsj3	0.006348	0.155445	0.366	0.239	1
Tmem201	0.006353	0.125278	0.195	0.099	1
Sertad1	0.006371	0.267678	0.878	0.85	1
Dek	0.006381	0.302702	0.793	0.689	1
Sf3a3	0.006387	0.110763	0.451	0.294	1
Rrp1	0.006419	0.145006	0.732	0.553	1
Krtcap2	0.006458	-0.26698	0.927	0.958	1
Plcd3	0.006514	0.127756	0.195	0.099	1
Tulp4	0.006527	0.189002	0.951	0.843	1
Pfkl	0.006592	0.114562	0.28	0.159	1
Bmp2	0.006607	0.271623	0.305	0.191	1
Adrb2	0.006699	0.123252	0.195	0.093	1
Rnf24	0.006758	0.1332	0.537	0.38	1
Sox11	0.006765	0.105548	0.268	0.146	1
Cops7a	0.0068	-0.18226	0.402	0.511	1
Socs3	0.006814	0.552108	0.878	0.773	1
Mydgf	0.006854	-0.19953	0.707	0.77	1

Cdc34	0.006857	0.145513	0.439	0.303	1
Kdm6b	0.006866	0.272004	0.939	0.911	1
Gxylt2	0.006868	-0.27952	0.78	0.83	1
Arl5c	0.006911	0.171146	0.171	0.082	1
Mef2a	0.006936	0.206494	0.939	0.936	1
Rorb	0.006961	0.225194	0.366	0.24	1
Pwwp2a	0.00697	0.220286	0.451	0.32	1
Mapk8	0.007068	0.262053	0.732	0.611	1
Brd8	0.007084	0.152929	0.585	0.44	1
Phf23	0.007104	0.299957	0.756	0.588	1
Emp3	0.007124	-0.17253	0.963	0.952	1
Ginm1	0.007136	-0.25182	0.634	0.757	1
Htra2	0.007161	-0.12326	0.244	0.386	1
Mark2	0.0072	0.118908	0.659	0.491	1
Cnpy2	0.007251	-0.23738	0.89	0.93	1
Phf20l1	0.007251	0.171289	0.72	0.617	1
Dst	0.007312	0.382333	0.866	0.758	1
Cdh4	0.007324	-0.25193	0.671	0.783	1
Phf10	0.007328	0.124538	0.793	0.648	1
Ppp1r14a	0.007329	-0.37192	0.671	0.739	1
Ly6e	0.007382	-0.22376	0.829	0.884	1
Simc1	0.007383	0.12241	0.305	0.179	1
Phb2	0.007386	0.160833	0.78	0.694	1
Paxbp1	0.007444	0.116768	0.463	0.3	1
Sde2	0.007494	0.357109	0.756	0.652	1
Ost4	0.007536	-0.18941	0.902	0.948	1
Stxbp1	0.007545	0.106786	0.524	0.365	1
Cks2	0.007551	0.19602	0.378	0.243	1
Stau1	0.007574	0.179336	0.646	0.507	1
Srsf7	0.007588	0.284774	0.793	0.651	1
Ikbip	0.007623	-0.21468	0.756	0.82	1
Amd1	0.007665	0.157617	0.427	0.293	1
Cry1	0.007695	0.107981	0.341	0.213	1
Wee1	0.007697	0.151532	0.451	0.307	1
Col1a1	0.007703	-0.31045	1	1	1
Ube2e3	0.007705	0.18687	0.768	0.632	1
Ndufs6	0.007712	-0.17897	0.634	0.753	1
Snapc5	0.007768	-0.18327	0.378	0.521	1
Siah1a	0.007807	0.13895	0.5	0.371	1
Gng12	0.007811	-0.27222	0.915	0.94	1
Slc35d1	0.007834	0.114542	0.317	0.197	1
C1qtnf6	0.007852	-0.19878	0.78	0.911	1
Atp2a2	0.007871	0.15118	0.927	0.888	1

Ptbp2	0.007905	0.170498	0.451	0.319	1
Tnpo2	0.007913	0.126053	0.512	0.361	1
Vstm4	0.007959	-0.16159	0.22	0.352	1
Pde4d	0.007961	0.188501	0.561	0.448	1
Ak6	0.007961	0.201413	0.39	0.271	1
Malat1	0.007972	0.448492	1	0.999	1
Hdac4	0.007978	0.205477	0.683	0.559	1
Lox	0.007989	-0.28124	0.768	0.907	1
Txlng	0.008009	0.123983	0.268	0.157	1
Rab5a	0.008034	0.17615	0.817	0.757	1
Serf1	0.008041	-0.18586	0.524	0.672	1
Zcchc11	0.008042	0.191445	0.659	0.502	1
Ncor1	0.008096	0.185338	0.89	0.812	1
Tmem260	0.008117	-0.10855	0.11	0.231	1
Kdm3a	0.008165	0.16717	0.585	0.466	1
U2af1	0.008178	0.161396	0.695	0.555	1
Ugdh	0.008191	0.289273	0.927	0.854	1
Zmym5	0.008226	0.16578	0.744	0.6	1
Jmjd6	0.008263	0.257066	0.622	0.46	1
Ostm1	0.008292	0.107014	0.476	0.311	1
Pim3	0.008326	0.651128	0.72	0.633	1
Bcl6	0.008343	0.127025	0.439	0.309	1
Prrc2c	0.008357	0.265181	0.902	0.869	1
Tgfbr1	0.008417	0.170514	0.72	0.576	1
2700081O	0.008448	0.179564	0.695	0.553	1
Ptpn13	0.008465	0.146047	0.439	0.311	1
Katna1	0.008485	0.142192	0.512	0.378	1
Phf12	0.008498	0.138045	0.476	0.339	1
Bag3	0.008506	0.224309	0.915	0.825	1
Prdm2	0.008509	0.206159	0.622	0.514	1
Serping1	0.008523	-0.23425	0.951	0.991	1
Frem1	0.008527	0.201327	0.183	0.09	1
Sfpq	0.008531	0.284853	0.988	0.923	1
Fkbp10	0.008571	-0.19158	0.78	0.886	1
Errfi1	0.008588	0.30609	0.976	0.978	1
Selenow	0.008589	-0.19462	0.976	0.983	1
Cyr61	0.008608	0.593015	0.939	0.923	1
Cnot1	0.00864	0.139712	0.707	0.543	1
Itgbl1	0.008699	-0.27984	0.793	0.891	1
Eif5a	0.008704	0.262536	0.951	0.948	1
Gstm1	0.008783	-0.26279	0.768	0.809	1
Mmp23	0.008829	-0.23926	0.817	0.901	1
Taf4b	0.009038	0.16258	0.268	0.157	1

Jmjd1c	0.009111	0.211976	0.939	0.852	1
Prdm11	0.009131	0.111524	0.146	0.067	1
Camk2n1	0.009139	0.286314	0.671	0.55	1
Cdk12	0.009165	0.261126	0.732	0.613	1
Hspg2	0.009179	-0.24051	0.854	0.939	1
Tmem234	0.00918	-0.19471	0.756	0.803	1
Scmh1	0.009223	0.143507	0.537	0.365	1
Cntrl	0.009239	0.157586	0.317	0.204	1
Pabpn1	0.009247	0.186139	0.573	0.426	1
Zfp280d	0.009255	0.169261	0.39	0.25	1
Ppp2r5e	0.009285	0.157915	0.61	0.461	1
Uhrf2	0.00933	0.255246	0.732	0.575	1
Tcp1	0.009337	0.140137	0.768	0.646	1
Pik3c2a	0.009349	0.140475	0.488	0.351	1
Poldip3	0.009358	0.170391	0.646	0.544	1
Kcnq1ot1	0.009376	0.5076	0.756	0.633	1
Dstn	0.009377	0.173371	0.988	0.937	1
Crtc2	0.009416	0.181309	0.561	0.397	1
Ppil4	0.009462	0.160278	0.512	0.362	1
Vcp	0.009479	0.208587	0.915	0.932	1
Hyou1	0.009482	0.135777	0.512	0.345	1
Ntn4	0.009483	-0.26485	0.378	0.521	1
Dmpk	0.009518	0.102033	0.305	0.188	1
2810403A(	0.009523	0.144221	0.61	0.448	1
Rara	0.009612	0.157451	0.415	0.285	1
Fxr1	0.009677	0.16244	0.805	0.655	1
Mgst1	0.009693	-0.21068	0.915	0.955	1
Cdkl5	0.009693	0.107276	0.122	0.051	1
Zfp281	0.009796	0.110115	0.402	0.269	1
Tmx4	0.009811	0.156469	0.366	0.25	1
Fam104a	0.009815	0.154607	0.78	0.616	1
Sirt1	0.009909	0.251358	0.707	0.601	1
Aco2	0.009946	0.174706	0.732	0.622	1
Ube2d2a	0.009982	0.14928	0.866	0.821	1
Nfil3	0.010077	0.25947	0.72	0.553	1
Nup98	0.010149	0.290154	0.756	0.639	1
Ednra	0.010163	0.112274	0.232	0.127	1
Gigyf1	0.010168	0.141851	0.549	0.41	1
Polr2e	0.010232	-0.19969	0.524	0.616	1
Trim59	0.010249	0.115788	0.207	0.108	1
Arid5b	0.010258	0.174706	0.915	0.805	1
Lemd3	0.010271	0.114352	0.378	0.239	1
Ss18	0.010355	0.158587	0.659	0.536	1

Sesn3	0.010374	0.183898	0.573	0.434	1
Rtn4	0.010382	0.168652	0.976	0.967	1
Zfr	0.010409	0.192227	0.829	0.689	1
9930021J0	0.010531	0.21827	0.659	0.565	1
Gsk3a	0.010546	0.188234	0.805	0.665	1
Ifngr2	0.010602	-0.14668	0.293	0.415	1
Gabarap	0.01063	-0.194	0.976	0.996	1
Hnrnpc	0.010672	0.191247	0.927	0.85	1
Plk3	0.010676	0.132545	0.512	0.364	1
Surf4	0.010677	-0.22313	0.878	0.908	1
Pcbp2	0.010686	0.171332	1	0.972	1
Git2	0.010709	0.156221	0.573	0.445	1
Srsf3	0.010834	0.305831	0.939	0.87	1
Ubn2	0.010843	0.154783	0.829	0.691	1
Prkd3	0.010982	-0.2012	0.39	0.499	1
Tgfbi	0.010985	-0.31958	0.854	0.942	1
Ppic	0.011013	-0.19995	0.927	0.991	1
Trps1	0.01102	0.536535	0.78	0.75	1
Atg101	0.011057	0.132394	0.524	0.38	1
Cish	0.011125	0.102034	0.244	0.137	1
Ndufaf2	0.011241	0.145286	0.549	0.399	1
Htatip2	0.011252	-0.15524	0.317	0.441	1
Impdh2	0.011255	0.193386	0.78	0.659	1
Ppp1r18	0.011287	0.195577	0.707	0.592	1
Pnn	0.011311	0.326427	0.573	0.48	1
Hist4h4	0.011429	0.120155	0.122	0.052	1
Inhbb	0.011441	-0.2104	0.622	0.725	1
Gnai1	0.01145	0.1627	0.28	0.173	1
Cops6	0.011521	-0.18453	0.524	0.626	1
Pdcl3	0.011565	0.111017	0.72	0.543	1
Csad	0.011582	0.212105	0.5	0.36	1
Ywhae	0.011609	0.169079	0.963	0.943	1
Rsf1	0.011688	0.220481	0.72	0.606	1
Tmem263	0.011699	-0.20614	0.598	0.71	1
Jpt1	0.011713	0.273718	0.854	0.809	1
Spon2	0.011727	-0.34104	0.537	0.656	1
Hspb8	0.01176	0.293031	0.866	0.802	1
Ppm1a	0.011769	0.229981	0.915	0.801	1
Ccpg1	0.011779	-0.18193	0.415	0.517	1
Arl1	0.011793	-0.18084	0.878	0.921	1
Cdo1	0.011809	0.124443	0.354	0.226	1
Srek1	0.011814	0.243338	0.756	0.677	1
3830406C1	0.011833	-0.14506	0.28	0.397	1

Ghr	0.011845	-0.22786	0.78	0.884	1
Hprt	0.01185	0.115146	0.537	0.4	1
Clk4	0.011886	0.265987	0.61	0.486	1
Ldha	0.011894	0.253537	0.805	0.723	1
Atp13a3	0.012013	0.23063	0.72	0.559	1
Eif5b	0.012075	0.245725	0.817	0.732	1
Pla2g2e	0.012158	-0.31116	0.622	0.668	1
Abcg2	0.012179	0.111493	0.39	0.268	1
Hsp90ab1	0.012312	0.391063	1	1	1
Eloc	0.012346	0.262621	0.841	0.757	1
Tnfsf9	0.012402	0.184336	0.488	0.342	1
Polr2h	0.012411	0.145464	0.5	0.362	1
Oaz1	0.012431	0.18611	1	0.994	1
Hnrnpd	0.012438	0.246956	0.732	0.624	1
Nlgn2	0.012477	0.141504	0.463	0.323	1
Otud7b	0.012494	0.104981	0.463	0.325	1
Fem1c	0.012573	0.128059	0.378	0.243	1
Nsd2	0.01258	0.176916	0.329	0.208	1
Focad	0.012605	0.139396	0.22	0.128	1
Ncam1	0.012822	0.350274	0.415	0.291	1
Nav2	0.012848	0.156442	0.232	0.135	1
Ly6a	0.012955	-0.23024	0.866	0.977	1
Dhx15	0.012981	0.170533	0.817	0.713	1
Npm1	0.012983	0.485721	0.988	0.955	1
Ptk7	0.013015	0.244226	0.585	0.447	1
Zc3h12a	0.013059	0.12326	0.451	0.3	1
Rab34	0.013072	0.173204	0.841	0.747	1
Spaca6	0.013076	0.126954	0.451	0.323	1
Cep170b	0.01312	0.134245	0.402	0.284	1
Tmem183a	0.013131	0.12752	0.5	0.365	1
Cep164	0.01315	0.15017	0.439	0.304	1
Cyth1	0.013231	0.118319	0.366	0.252	1
Tax1bp1	0.013234	0.204107	0.927	0.863	1
Igf1	0.013334	-0.30638	0.902	0.942	1
Rangap1	0.01334	0.121546	0.341	0.223	1
Ptgfrn	0.013341	-0.19312	0.805	0.85	1
Atp11a	0.013356	0.116453	0.744	0.574	1
Hnrnpa3	0.013389	0.391166	0.927	0.898	1
Dynlrb1	0.013397	-0.20622	0.927	0.949	1
Gtf2f1	0.013401	0.161015	0.561	0.412	1
Hspa1a	0.013406	0.824543	0.89	0.875	1
Bmpr1a	0.013419	0.103164	0.756	0.594	1
Thoc6	0.01347	0.2738	0.659	0.507	1

Foxo3	0.013545	0.225195	0.634	0.527	1
Nbl1	0.013561	-0.309	0.902	0.974	1
Col5a2	0.013663	-0.16449	0.988	1	1
Scarf2	0.0137	-0.17879	0.866	0.9	1
Mesd	0.013763	-0.19934	0.5	0.598	1
Luc7l3	0.013764	0.328117	0.829	0.696	1
Hnrnpa0	0.013816	0.307918	0.963	0.923	1
Crebrf	0.01402	0.158154	0.805	0.655	1
Zbtb16	0.014196	0.149617	0.646	0.546	1
Slc6a2	0.014231	-0.12315	0.073	0.178	1
Thoc2	0.014289	0.175529	0.817	0.707	1
Fkbp9	0.014304	-0.18165	0.866	0.967	1
Cand1	0.014313	0.253105	0.646	0.52	1
Dag1	0.014367	-0.16929	0.878	0.914	1
Scarb1	0.014385	0.117295	0.207	0.116	1
Myd88	0.014401	0.213903	0.585	0.485	1
Pygm	0.014405	0.149981	0.268	0.172	1
Csnk2a2	0.014428	0.147181	0.659	0.523	1
Flot1	0.014464	-0.19178	0.537	0.635	1
Rbm28	0.014556	0.121205	0.5	0.377	1
Clic4	0.014601	0.247077	0.963	0.967	1
Nop58	0.014629	0.32338	0.683	0.568	1
Ube2q2	0.014644	0.198174	0.585	0.459	1
Nt5e	0.014706	-0.17623	0.439	0.539	1
Cdh13	0.014767	-0.17679	0.39	0.541	1
Hirip3	0.014872	0.100114	0.183	0.095	1
Mrc2	0.014877	-0.1991	0.866	0.891	1
Nradd	0.014899	0.156708	0.451	0.336	1
Fermt2	0.014941	0.33888	0.817	0.764	1
Ptdss1	0.015029	-0.14242	0.293	0.399	1
Srrt	0.01503	0.255894	0.573	0.445	1
Med13l	0.01503	0.251531	0.878	0.812	1
Ppp1r15a	0.015037	0.273896	0.976	0.921	1
Pole4	0.015057	0.158427	0.622	0.475	1
Bdh2	0.015123	-0.22149	0.549	0.662	1
AC160336.	0.015175	-0.20977	0.72	0.566	1
Prkar1a	0.015194	0.119885	0.951	0.869	1
Ddx3y	0.015205	0.168537	0.793	0.712	1
Mrpl10	0.015214	-0.11298	0.207	0.33	1
Seh1l	0.015235	0.11128	0.427	0.297	1
Zfp821	0.015296	0.119791	0.28	0.173	1
Rps4x	0.015532	-0.15406	1	0.999	1
Spag9	0.015568	0.184504	0.963	0.942	1

Fscn1	0.015596	0.19847	0.463	0.328	1
Zmiz1	0.015631	0.143723	0.927	0.868	1
Ptges3	0.015656	0.218793	0.805	0.787	1
Pfn2	0.015693	0.153166	0.341	0.22	1
Mex3c	0.015763	0.223346	0.646	0.534	1
Clk1	0.015885	0.342581	0.902	0.846	1
Mrpl36	0.015896	-0.16859	0.305	0.428	1
Mef2d	0.015939	0.201196	0.744	0.648	1
Ndufc2	0.015943	-0.21012	0.732	0.802	1
Twsg1	0.015987	-0.23068	0.817	0.868	1
Msl2	0.016009	0.22002	0.634	0.55	1
Rps27rt	0.016024	0.212045	0.841	0.737	1
Eif1	0.016092	0.316839	1	1	1
2810474O	0.016122	0.106953	0.646	0.488	1
Stk19	0.016246	0.148884	0.5	0.381	1
Cul1	0.016269	0.125133	0.732	0.635	1
Zrsr2	0.01632	0.174152	0.524	0.409	1
Srsf11	0.016366	0.133217	0.939	0.865	1
Tmem243	0.016398	0.101313	0.451	0.322	1
Arhgef17	0.016404	0.107657	0.317	0.207	1
Hp1bp3	0.016432	-0.16214	0.683	0.809	1
Fam105a	0.016472	-0.28117	0.537	0.616	1
Max	0.016475	0.144298	0.646	0.539	1
Rsl1d1	0.016562	0.160494	0.585	0.464	1
Stmn1	0.016686	0.207932	0.256	0.163	1
Rsrp1	0.016716	0.169498	0.988	0.978	1
Mxra7	0.016719	-0.19375	0.854	0.841	1
Ppp1cb	0.016724	0.162936	0.915	0.85	1
Fis1	0.016824	-0.1889	0.72	0.783	1
Pdhb	0.016904	0.126957	0.659	0.521	1
Gpx8	0.016926	-0.22172	0.878	0.888	1
Larp4	0.017265	0.227366	0.78	0.645	1
Zfp703	0.017284	0.264785	0.573	0.461	1
Rps17	0.017372	-0.1572	0.976	0.968	1
Sec31a	0.017429	-0.18775	0.805	0.897	1
Sh3d21	0.017434	0.100995	0.195	0.109	1
Gm14964	0.017491	0.18765	0.28	0.18	1
Camsap2	0.017569	0.165828	0.512	0.39	1
Zranb1	0.017599	0.243683	0.793	0.691	1
Ahnak	0.017649	0.153015	1	0.999	1
Scfd1	0.017709	-0.1306	0.305	0.422	1
Rfx7	0.017747	0.19068	0.585	0.432	1
Epc1	0.017842	0.131671	0.671	0.514	1

Bclaf3	0.017957	0.158665	0.305	0.199	1
Tmed3	0.017971	-0.2009	0.89	0.956	1
Cd68	0.017996	0.119941	0.195	0.109	1
Chmp4c	0.018102	-0.16027	0.22	0.328	1
Ccdc47	0.018222	0.118011	0.598	0.48	1
Gas6	0.018252	-0.29408	0.927	0.949	1
Ddr2	0.018261	-0.20383	0.927	0.901	1
Sulf2	0.01833	-0.25306	0.768	0.894	1
Mprip	0.018331	0.129592	0.829	0.677	1
Kptn	0.018377	0.120732	0.39	0.268	1
Mepce	0.018392	0.238249	0.671	0.528	1
Rps6kb1	0.018453	0.170583	0.805	0.674	1
Ankrd13c	0.018469	0.197613	0.451	0.323	1
Papss2	0.018481	-0.11892	0.146	0.247	1
Pan3	0.018545	0.132313	0.768	0.64	1
Iffo2	0.018555	0.185404	0.39	0.284	1
Akt1	0.018682	0.142584	0.902	0.755	1
Pspc1	0.018723	0.134158	0.329	0.215	1
Map1s	0.018799	0.100308	0.28	0.175	1
Serpina3n	0.018845	-0.29986	0.5	0.608	1
Sik1	0.018964	0.241007	0.671	0.572	1
Appbp2	0.018974	0.131615	0.805	0.67	1
Gna12	0.019118	0.153776	0.598	0.447	1
Mrpl38	0.019141	0.120478	0.427	0.295	1
Atxn10	0.019245	-0.19345	0.72	0.78	1
Cacna1g	0.019265	0.193895	0.671	0.562	1
Rad21	0.019388	0.140203	0.768	0.638	1
Camsap1	0.019427	0.101345	0.415	0.291	1
U2af2	0.019501	0.194606	0.659	0.575	1
Sub1	0.019524	0.257402	0.915	0.841	1
BC005537	0.019593	0.220043	0.951	0.917	1
Clcf1	0.019614	0.16281	0.183	0.1	1
Usp9x	0.019625	0.211309	0.89	0.822	1
Unc5b	0.019874	0.216168	0.28	0.185	1
Mdk	0.019916	0.172615	0.378	0.253	1
Tmem106l	0.020017	-0.18766	0.634	0.748	1
Cog5	0.020067	0.134475	0.427	0.306	1
Crebbp	0.020099	0.189852	0.902	0.824	1
Uqcrfs1	0.02011	0.128962	0.695	0.579	1
Styx	0.020135	0.114794	0.329	0.218	1
Sema3a	0.020194	-0.21787	0.28	0.41	1
Lama4	0.020196	-0.17743	0.61	0.738	1
Gm42715	0.020207	0.10253	0.354	0.24	1

Rb1cc1	0.020252	0.173044	0.817	0.718	1
Nr2c2	0.020415	0.127468	0.537	0.426	1
Gadd45b	0.020499	0.463376	0.963	0.882	1
Col6a1	0.020525	-0.49711	0.927	0.942	1
Prr14	0.020613	0.114891	0.524	0.4	1
Nbeal1	0.020662	-0.1845	0.695	0.748	1
Psmd11	0.020682	0.164891	0.817	0.712	1
Blvrb	0.020729	-0.21565	0.634	0.683	1
Rbmxl1	0.020748	0.129514	0.402	0.29	1
Mdm2	0.020766	0.149282	0.744	0.639	1
Ewsr1	0.020777	0.161668	0.866	0.742	1
Ikzf5	0.020882	0.111242	0.573	0.429	1
Cenpc1	0.020884	0.138395	0.354	0.24	1
Cic	0.020884	0.10912	0.5	0.349	1
Sec24d	0.020949	-0.16318	0.683	0.82	1
Tiparp	0.020984	0.25637	0.72	0.639	1
mt-Nd5	0.021044	-0.25375	0.976	0.972	1
Mafa	0.021129	0.111212	0.195	0.112	1
Cct4	0.021173	0.133717	0.89	0.737	1
Serpinf1	0.02131	-0.15924	0.988	0.975	1
Hcfc2	0.021314	-0.12648	0.232	0.351	1
Ndufs2	0.021347	-0.20551	0.78	0.82	1
Fus	0.021354	0.236038	0.976	0.932	1
Dennd4a	0.021467	0.114564	0.5	0.368	1
Suds3	0.021596	0.158975	0.72	0.563	1
Pxk	0.021676	-0.11531	0.415	0.531	1
Celf1	0.021685	0.108672	0.756	0.619	1
Idi1	0.021694	0.163927	0.256	0.162	1
Psap	0.021729	-0.1955	0.988	0.997	1
H6pd	0.02178	-0.1234	0.183	0.287	1
Hnrnpul1	0.021781	0.270153	0.829	0.713	1
Mafb	0.021849	0.141496	0.659	0.501	1
Lrrn4cl	0.022014	0.233297	0.707	0.611	1
Pdcd6ip	0.02214	0.155041	0.866	0.754	1
Cdc5l	0.022181	0.122812	0.585	0.432	1
Arpc3	0.022351	-0.17777	0.89	0.939	1
Cdk4	0.022356	-0.20781	0.598	0.719	1
Ybx1	0.022401	0.109331	0.976	0.983	1
Fam222b	0.022404	0.145663	0.232	0.143	1
Dyrk2	0.022568	0.180567	0.537	0.453	1
Mum1	0.022633	0.119144	0.317	0.211	1
Nolc1	0.022642	0.199546	0.451	0.344	1
Aqp1	0.022699	0.116276	0.171	0.093	1

Zcchc2	0.022752	0.101716	0.244	0.151	1
Plppr3	0.022753	0.123859	0.415	0.288	1
Rbm26	0.022792	0.150369	0.634	0.501	1
Fam129b	0.0228	0.256496	0.634	0.498	1
Sh3d19	0.022857	-0.17795	0.768	0.793	1
Sod2	0.022892	0.310109	0.695	0.569	1
Cox6c	0.022916	-0.16147	0.976	0.978	1
Ppp2cb	0.022962	0.137227	0.476	0.377	1
Utp18	0.023049	0.107695	0.354	0.236	1
Ctbp2	0.023059	0.195165	0.512	0.424	1
Snrpf	0.023087	0.142303	0.817	0.79	1
Art3	0.023115	0.135023	0.293	0.189	1
Aebp1	0.023123	-0.26665	0.988	0.991	1
0610010K1	0.023223	0.100256	0.439	0.314	1
Soga1	0.023258	0.131781	0.207	0.128	1
Cln5	0.023289	-0.13614	0.305	0.422	1
Commd1	0.023322	-0.16509	0.561	0.622	1
Tram2	0.023326	-0.16753	0.537	0.656	1
Fbxl20	0.023476	0.129761	0.537	0.412	1
Gphn	0.023574	0.118735	0.293	0.189	1
Slc39a13	0.023599	-0.18553	0.463	0.56	1
Map1lc3b	0.023636	0.215731	1	0.978	1
Mrps6	0.023643	0.243619	0.439	0.332	1
Gm47283	0.023671	0.785119	0.378	0.3	1
Peli2	0.023786	-0.15752	0.402	0.502	1
Calm3	0.023866	-0.17167	0.817	0.869	1
Thrap3	0.023885	0.20277	0.768	0.709	1
Senp2	0.023886	0.280785	0.659	0.553	1
Tlk2	0.023896	0.123602	0.61	0.457	1
Ier5l	0.02398	0.301107	0.439	0.33	1
Ctdspl2	0.024049	0.133414	0.488	0.355	1
Mpdu1	0.024114	-0.19622	0.451	0.541	1
Tdp2	0.024119	0.132949	0.402	0.278	1
Ldhb	0.024152	0.100526	0.146	0.076	1
Gla	0.024158	0.145355	0.293	0.199	1
Fcgrt	0.024241	-0.18509	0.939	0.972	1
Htra3	0.024256	-0.20966	0.927	0.965	1
Eno1	0.024418	0.127396	0.671	0.537	1
Cnbp	0.024542	0.217164	0.939	0.869	1
Sun2	0.024552	0.203822	0.683	0.591	1
Tgs1	0.024648	-0.11126	0.171	0.275	1
Fnbp1l	0.024659	0.118333	0.268	0.173	1
Grasp	0.024679	0.438057	0.646	0.559	1

Rrp9	0.02468	0.101252	0.232	0.144	1
Spire1	0.024726	0.101744	0.256	0.159	1
Rpl9-ps6	0.02477	0.145936	0.598	0.473	1
Bcl10	0.024902	0.131339	0.732	0.604	1
Yy1	0.024942	0.411805	0.902	0.792	1
Dennd5b	0.025024	0.140243	0.341	0.234	1
Elovl6	0.025075	0.165773	0.476	0.357	1
Ubr4	0.025223	0.165669	0.671	0.509	1
Lpar4	0.025407	-0.13934	0.146	0.245	1
Ext1	0.025478	0.137532	0.78	0.616	1
Mex3d	0.025483	0.124894	0.512	0.389	1
Atxn2l	0.025494	0.235807	0.744	0.672	1
Apoe	0.025613	0.870464	0.927	0.917	1
Thy1	0.025671	-0.15163	0.634	0.753	1
Qrich1	0.025721	0.116604	0.707	0.547	1
Etv6	0.025758	0.162813	0.329	0.223	1
Ifngr1	0.025859	-0.21889	0.72	0.78	1
Ciao1	0.025888	-0.10459	0.159	0.259	1
Cdc40	0.025969	0.117434	0.622	0.493	1
Phf21a	0.02605	0.124775	0.683	0.55	1
Fnip1	0.026277	0.233109	0.902	0.882	1
Slc38a10	0.026409	-0.15802	0.793	0.905	1
Gulp1	0.026496	0.125466	0.659	0.544	1
Sowahc	0.026589	0.128238	0.537	0.406	1
Mtus1	0.026683	0.124039	0.354	0.261	1
Mapk3	0.026802	-0.15233	0.549	0.643	1
Eci2	0.026964	0.126846	0.634	0.505	1
Cldnd1	0.027051	0.105578	0.671	0.544	1
Snai1	0.027098	0.518914	0.524	0.431	1
Ola1	0.027274	0.125252	0.524	0.406	1
Ube2g1	0.027344	0.15363	0.634	0.523	1
Ell	0.027358	0.123686	0.476	0.355	1
Tanc1	0.027405	0.263529	0.537	0.412	1
Sik3	0.027482	0.180587	0.817	0.671	1
Ap2s1	0.027643	-0.17165	0.683	0.723	1
Thbs2	0.027741	-0.21588	0.939	0.985	1
Dnajc10	0.027806	-0.15663	0.537	0.626	1
C3	0.027977	-0.51584	0.439	0.521	1
Zcchc6	0.028183	0.1317	0.768	0.684	1
S100a16	0.028241	-0.26211	0.768	0.811	1
Mylip	0.028252	0.162225	0.89	0.763	1
Nedd8	0.028278	-0.18307	0.829	0.891	1
Gm10076	0.028388	-0.16136	0.878	0.891	1

Fkbp7	0.02853	-0.18209	0.89	0.923	1
Capns1	0.028734	-0.14656	0.915	0.955	1
Pogz	0.028754	0.134306	0.451	0.342	1
Lncpint	0.028756	0.185729	0.659	0.518	1
Ubn1	0.028832	0.211544	0.805	0.687	1
Fam168a	0.028909	0.120802	0.646	0.546	1
Hmbs	0.02916	0.116076	0.354	0.247	1
Stat5a	0.02924	0.105779	0.39	0.278	1
Nr4a3	0.029257	0.245575	0.524	0.387	1
Vapa	0.029353	0.194314	0.915	0.873	1
Fbln2	0.029462	-0.22999	0.963	0.99	1
Rex1bd	0.029507	-0.17208	0.671	0.734	1
Sf1	0.029551	0.135682	0.768	0.712	1
Dusp8	0.029763	0.132603	0.39	0.274	1
Pten	0.02979	-0.1849	0.927	0.932	1
Dbndd2	0.029828	0.136689	0.134	0.07	1
Dync1h1	0.02989	0.1084	0.902	0.838	1
Gpx7	0.029903	-0.16061	0.683	0.753	1
Ppig	0.02992	0.287225	0.72	0.668	1
Snu13	0.030177	0.222958	0.829	0.786	1
Ccdc138	0.030183	0.114311	0.28	0.191	1
Uap1	0.0303	0.130638	0.878	0.847	1
Pptc7	0.030319	0.139293	0.573	0.477	1
Sos2	0.030368	0.100587	0.537	0.426	1
Far1	0.030418	0.158094	0.659	0.518	1
Inpp4a	0.030524	0.100786	0.402	0.284	1
March9	0.03057	0.125682	0.146	0.079	1
Cpeb2	0.030606	0.144223	0.573	0.454	1
Ints7	0.030701	0.111722	0.207	0.125	1
Tssc4	0.030892	0.13058	0.512	0.38	1
Lag3	0.0311	0.110502	0.232	0.144	1
Ebpl	0.031208	0.174329	0.439	0.339	1
Mrps18b	0.03122	0.105494	0.378	0.258	1
Dhps	0.031258	0.107366	0.439	0.336	1
Uqcrc2	0.031293	0.1079	0.646	0.531	1
Eif4h	0.031347	0.144201	0.89	0.795	1
Cast	0.031349	-0.18789	0.817	0.865	1
Srpk2	0.031374	0.12028	0.756	0.633	1
Enah	0.031447	0.368179	0.476	0.389	1
Slc25a4	0.031458	-0.19681	0.878	0.913	1
Hexa	0.031468	-0.20616	0.89	0.932	1
Irf1	0.031488	0.213001	0.89	0.799	1
Jam2	0.031679	0.123823	0.476	0.364	1

Fap	0.031692	0.12358	0.329	0.215	1
Nav3	0.031779	0.159655	0.159	0.087	1
Tmem18	0.031884	-0.1066	0.11	0.197	1
Atf4	0.031917	0.189775	0.988	0.972	1
Syncrip	0.031969	0.201198	0.866	0.755	1
Fbxo30	0.032078	0.194716	0.573	0.473	1
Map3k8	0.03208	0.187774	0.512	0.413	1
Prkab2	0.032125	0.165484	0.5	0.402	1
Zfp593	0.03226	0.135325	0.256	0.162	1
Hsd17b10	0.032564	-0.16942	0.476	0.54	1
Nfkbiz	0.03279	0.254817	0.829	0.818	1
Phkg2	0.032981	0.164601	0.378	0.279	1
Haus3	0.03308	0.100447	0.293	0.195	1
Gcnt2	0.033135	0.124823	0.256	0.164	1
Rel	0.033235	0.235694	0.549	0.463	1
Mllt10	0.033292	0.159373	0.598	0.505	1
Ilf3	0.033445	0.131695	0.659	0.52	1
Scarb2	0.033479	0.145203	0.549	0.434	1
Tubb4b	0.03358	0.243387	0.927	0.865	1
Egr1	0.03388	0.179306	0.988	0.988	1
Zc3hav1	0.033903	0.101112	0.805	0.699	1
Tmem45a	0.033963	-0.18793	0.659	0.75	1
Adam9	0.034055	0.131762	0.72	0.581	1
Tfe3	0.034232	0.143372	0.585	0.479	1
Arid4a	0.034397	0.230895	0.598	0.504	1
Ssr4	0.034445	-0.23433	0.963	0.945	1
Gusb	0.034477	-0.14193	0.561	0.648	1
Sav1	0.034525	-0.1716	0.634	0.703	1
Gtf2h1	0.034566	0.141672	0.549	0.428	1
Ring1	0.034638	0.103952	0.5	0.378	1
Ell2	0.034653	0.101281	0.622	0.489	1
Copz1	0.034731	-0.14748	0.573	0.664	1
Spen	0.034779	0.150406	0.573	0.483	1
Vit	0.034974	-0.20183	0.317	0.396	1
Denr	0.035032	-0.12914	0.5	0.603	1
Agfg1	0.035042	0.15482	0.634	0.48	1
Tns3	0.03507	0.325394	0.268	0.179	1
Spon1	0.035112	0.124301	0.646	0.518	1
Grn	0.035157	-0.16657	0.915	0.936	1
Nr4a2	0.035337	0.383056	0.561	0.456	1
Pmp22	0.035388	-0.20181	0.951	0.977	1
Ints13	0.035437	0.133855	0.378	0.275	1
Otud5	0.035484	0.121832	0.524	0.399	1

Cope	0.035634	-0.17764	0.817	0.859	1
Herc1	0.035743	0.113055	0.72	0.604	1
Pdgfrl	0.035796	-0.1653	0.732	0.801	1
Pcbp1	0.035815	0.143907	0.951	0.924	1
Ndufa4l2	0.035983	-0.30209	0.732	0.853	1
Sarnp	0.036023	0.103564	0.805	0.675	1
Reep3	0.036053	0.185112	0.915	0.917	1
Tmem242	0.036254	-0.1173	0.305	0.412	1
Gal	0.036319	-0.22077	0.098	0.189	1
Ctsa	0.036356	-0.15983	0.793	0.838	1
2410004B1	0.036974	0.102095	0.549	0.416	1
Evl	0.037156	0.143784	0.439	0.311	1
Lrrc8a	0.037237	0.126503	0.634	0.523	1
Gtf3c2	0.037251	0.101653	0.573	0.475	1
Itm2b	0.037386	-0.12075	1	1	1
Arrdc3	0.037464	-0.27989	0.305	0.396	1
Ldb1	0.037567	0.186459	0.707	0.616	1
Ankib1	0.037635	-0.12103	0.305	0.39	1
Usp15	0.037684	0.15364	0.524	0.454	1
Wwtr1	0.037851	0.196816	0.939	0.856	1
Ube2a	0.037856	0.118882	0.549	0.432	1
Auts2	0.037868	0.507961	0.354	0.269	1
Ptp4a1	0.03792	0.147656	0.89	0.872	1
Nfkb1	0.038148	0.189296	0.878	0.897	1
Cgnl1	0.038168	-0.11153	0.171	0.271	1
Cct8	0.038186	0.106981	0.805	0.707	1
Fmod	0.038284	0.780745	0.122	0.061	1
Npy1r	0.038303	-0.10385	0.073	0.159	1
Smc3	0.038346	0.217683	0.707	0.606	1
Actn1	0.038422	0.104597	0.195	0.119	1
Plekhh3	0.038605	0.123037	0.293	0.188	1
Lrp1	0.038754	-0.16471	0.976	0.997	1
Wtap	0.038813	0.141425	0.634	0.511	1
Tacc1	0.038953	0.135446	0.695	0.562	1
Dclk1	0.03897	-0.26622	0.585	0.697	1
Txnip	0.039046	-0.22218	0.671	0.751	1
Pum2	0.03906	0.122826	0.915	0.825	1
Galk1	0.03907	-0.16718	0.378	0.473	1
Pgpep1	0.039085	-0.12178	0.549	0.706	1
Tapbp	0.039507	-0.18826	0.878	0.886	1
Tmem97	0.039631	-0.10093	0.207	0.328	1
Nckap5l	0.039709	0.188358	0.415	0.316	1
Gsk3b	0.039754	0.238726	0.89	0.85	1

Mrpl4	0.039848	-0.13512	0.476	0.568	1
Usp53	0.039892	0.115629	0.427	0.323	1
Ypel3	0.039961	-0.17615	0.878	0.902	1
Gemin7	0.040265	-0.12103	0.378	0.488	1
Sema5a	0.040286	0.123561	0.451	0.319	1
Phldb1	0.040312	0.106626	0.841	0.745	1
Adamts2	0.040419	-0.21366	0.951	0.99	1
Rnf11	0.040649	0.110365	0.866	0.844	1
Rrm2b	0.040816	0.134021	0.573	0.459	1
Tsc22d2	0.041006	0.283548	0.768	0.703	1
Lhfp	0.041034	-0.15238	0.854	0.886	1
Pcsk6	0.041136	-0.12404	0.427	0.539	1
Terf2ip	0.041183	0.247444	0.329	0.22	1
Sfr1	0.041267	0.128363	0.963	0.956	1
Mmp3	0.041323	-0.40109	0.683	0.713	1
Calm1	0.041506	0.355096	0.951	0.965	1
Gm17501	0.041587	0.108796	0.183	0.108	1
Pbxip1	0.041768	-0.16218	0.646	0.737	1
Lpp	0.041811	0.175448	0.902	0.846	1
Fbxl6	0.041848	-0.10359	0.183	0.291	1
Crip2	0.041956	-0.21096	0.817	0.831	1
Arl4d	0.041975	0.173306	0.768	0.665	1
Arglu1	0.042024	0.103016	0.854	0.754	1
Pias2	0.042225	0.127605	0.561	0.435	1
Atxn2	0.042295	0.210238	0.756	0.686	1
Klhl7	0.042397	0.106557	0.427	0.333	1
Rbbp4	0.042587	0.277323	0.768	0.694	1
Magoh	0.042589	0.341862	0.744	0.623	1
Pkn1	0.042756	-0.10937	0.256	0.36	1
Nrn1	0.042865	0.214507	0.646	0.555	1
Shoc2	0.042888	0.136763	0.598	0.486	1
Ptbp1	0.042958	0.136811	0.951	0.895	1
Nr2f2	0.043298	0.199495	0.293	0.202	1
Sptlc2	0.043375	0.106494	0.671	0.587	1
Zhx1	0.043424	0.103754	0.451	0.352	1
Cox5b	0.043462	-0.1525	0.841	0.85	1
Eid1	0.043575	-0.19524	0.902	0.939	1
Pik3r1	0.043605	0.128027	0.915	0.834	1
Srgn	0.043863	0.134837	0.195	0.122	1
Myo1b	0.043986	0.23777	0.293	0.215	1
Naaa	0.04403	-0.21629	0.549	0.614	1
Cd276	0.044118	-0.11637	0.28	0.38	1
Polr3d	0.044166	0.105756	0.585	0.505	1

Uqcr11	0.044354	-0.10731	0.756	0.836	1
Ino80d	0.044368	0.166918	0.793	0.664	1
Plekha6	0.044516	0.106936	0.11	0.055	1
Pla2g5	0.044615	-0.27913	0.573	0.623	1
Tgfbr3	0.044706	-0.20925	0.939	0.965	1
Timp3	0.044745	-0.24898	0.841	0.856	1
Rac1	0.044862	-0.16113	0.927	0.924	1
Rnf19b	0.044923	0.147166	0.72	0.622	1
Rasa2	0.04503	0.116712	0.402	0.294	1
H2-T23	0.045119	-0.17537	0.744	0.779	1
Rtraf	0.045225	-0.18024	0.805	0.856	1
Podxl2	0.04526	0.106017	0.561	0.426	1
Eif3e	0.045277	0.162409	0.939	0.878	1
Strn3	0.045288	0.143459	0.854	0.809	1
Pdia3	0.045513	-0.1005	0.951	0.946	1
Phip	0.045527	0.208404	0.78	0.718	1
Anapc4	0.045592	-0.11538	0.268	0.373	1
Phf14	0.045602	0.105329	0.537	0.441	1
Ier3	0.045782	0.361177	0.976	0.967	1
Ppib	0.045871	-0.14448	0.988	0.984	1
Smo	0.045927	0.138487	0.695	0.576	1
Fkbp14	0.04625	-0.14312	0.646	0.713	1
Aebp2	0.046555	0.144162	0.707	0.619	1
Rnf145	0.046741	-0.13755	0.622	0.687	1
Ddit3	0.046746	0.20011	0.537	0.435	1
Srsf5	0.04685	0.252784	0.951	0.875	1
Map2k3	0.046951	0.183276	0.732	0.642	1
Wac	0.046957	0.312269	0.817	0.782	1
Thap4	0.046959	-0.10784	0.207	0.309	1
Pde10a	0.04696	0.105859	0.341	0.233	1
Tmem258	0.046988	-0.16715	0.793	0.873	1
Nxf1	0.047123	0.13985	0.817	0.729	1
Chtop	0.047217	0.148491	0.659	0.55	1
Abcc9	0.047308	0.171039	0.317	0.224	1
Smarca2	0.04737	0.164906	0.878	0.844	1
Fbxo32	0.047416	0.258938	0.524	0.409	1
Atp5g2	0.04746	-0.1921	0.939	0.93	1
Podn	0.047586	-0.19258	0.707	0.721	1
Hspa9	0.047727	0.221378	0.817	0.715	1
Atp11b	0.047756	0.199462	0.61	0.507	1
Helz	0.047985	0.14109	0.671	0.582	1
Zc3h15	0.048049	0.122658	0.878	0.77	1
Eprs	0.048109	-0.18979	0.768	0.796	1

Lamc3	0.048319	0.207859	0.549	0.45	1
Gm26802	0.048365	-0.30507	0.366	0.45	1
S100a13	0.048462	-0.18319	0.951	0.951	1
Cdc26	0.048621	-0.13016	0.646	0.699	1
Rbm6	0.048687	0.149713	0.585	0.47	1
Polr2f	0.048776	-0.11485	0.598	0.681	1
Col15a1	0.048964	-0.15525	0.866	0.929	1
Nudt4	0.049048	0.14748	0.72	0.63	1
Peg3	0.049304	0.179293	0.451	0.339	1
Akap8l	0.049451	0.104444	0.439	0.332	1
Spty2d1	0.049516	0.198087	0.768	0.693	1
Mrpl20	0.049625	-0.1033	0.634	0.731	1
Negr1	0.049783	-0.10574	0.11	0.192	1
Nectin2	0.049961	0.209271	0.232	0.157	1
Baz2a	0.049996	0.122204	0.561	0.431	1
Boc	0.05006	0.150925	0.537	0.424	1
Mrpl17	0.050115	-0.12217	0.524	0.651	1
Hnrnph3	0.050124	0.110792	0.537	0.437	1
Relb	0.050135	0.124132	0.512	0.392	1
Mapk1	0.050509	0.172413	0.817	0.745	1
Ddx27	0.050634	0.111494	0.28	0.198	1
Hip1	0.051081	0.138269	0.268	0.186	1
2900097C:	0.051113	-0.11856	0.829	0.876	1
Zfp385b	0.051152	-0.10652	0.122	0.21	1
Cherp	0.051343	0.141153	0.378	0.309	1
Hivep1	0.051387	0.148795	0.561	0.479	1
Cercam	0.051523	-0.14919	0.415	0.496	1
Zfp36l1	0.051549	0.235654	0.988	0.981	1
Capzb	0.051703	-0.14084	0.854	0.911	1
Bag1	0.05171	-0.16374	0.841	0.888	1
Dtnbp1	0.051753	0.1073	0.805	0.707	1
Sh3pxd2b	0.051981	-0.17402	0.768	0.827	1
Rnasel	0.052107	-0.10375	0.146	0.226	1
Srp19	0.05226	-0.1697	0.622	0.697	1
Atf3	0.052458	0.208109	0.951	0.865	1
Sar1a	0.052606	-0.14934	0.927	0.942	1
Arhgef12	0.052618	0.16271	0.939	0.898	1
Ube4b	0.052689	0.110883	0.659	0.571	1
Tpi1	0.052801	0.161254	0.768	0.654	1
Agtrap	0.053273	-0.16309	0.439	0.492	1
Tmem57	0.053336	0.16861	0.744	0.646	1
Sertad3	0.053358	0.131668	0.28	0.199	1
Nkd2	0.053432	0.180954	0.524	0.408	1

Purb	0.053608	0.276006	0.817	0.728	1
Rela	0.053926	0.132985	0.695	0.691	1
Atxn7l3	0.053949	0.102873	0.537	0.421	1
Cpsf7	0.054576	0.110695	0.5	0.38	1
Exoc5	0.054628	0.144684	0.72	0.61	1
Bdp1	0.054836	0.125266	0.5	0.418	1
Srsf1	0.054958	0.175934	0.598	0.523	1
Trim44	0.054997	0.118514	0.585	0.47	1
Ube2d3	0.055051	0.155185	1	0.983	1
Topors	0.05511	0.177483	0.671	0.572	1
Klf4	0.055419	0.255016	0.988	0.99	1
Ppp4r2	0.055588	0.121716	0.89	0.806	1
Timp1	0.055693	-0.44734	0.268	0.352	1
Slc50a1	0.056225	-0.17528	0.598	0.656	1
Ccnt2	0.056397	0.119764	0.415	0.322	1
Cyp26b1	0.056416	-0.28459	0.866	0.868	1
Zbtb1	0.056426	0.117921	0.451	0.333	1
Rai14	0.056472	0.112305	0.293	0.197	1
Eef1d	0.05652	-0.10343	0.915	0.929	1
Aga	0.056637	0.116626	0.707	0.614	1
Ndufv3	0.05664	-0.15306	0.72	0.757	1
Chd3os	0.057033	0.173539	0.171	0.103	1
Cd47	0.057183	-0.18387	0.963	0.987	1
Rhoj	0.057224	-0.17936	0.829	0.917	1
Lamp2	0.057286	-0.10547	0.976	0.984	1
Ftl1	0.057287	0.12073	1	1	1
Nnmt	0.057459	-0.18473	0.512	0.606	1
Il6ra	0.057514	0.139629	0.902	0.77	1
Mat2a	0.057656	0.333153	0.927	0.929	1
Birc3	0.057747	0.105934	0.646	0.521	1
mt-Nd3	0.05777	-0.10906	0.976	0.981	1
Runx1t1	0.057833	0.146691	0.671	0.578	1
Sdc1	0.05798	0.220681	0.61	0.489	1
Plod1	0.057985	-0.1288	0.866	0.913	1
Cbfa2t3	0.057993	0.145711	0.183	0.112	1
Gxylt1	0.058053	0.101639	0.451	0.352	1
Gpnmb	0.058106	-0.24272	0.805	0.888	1
Rbpms	0.058275	0.125	0.854	0.721	1
Srp72	0.058275	-0.18005	0.671	0.686	1
Nrd1	0.058402	0.103157	0.585	0.499	1
Wdr82	0.058562	0.101015	0.549	0.431	1
Lnpep	0.058646	0.109649	0.89	0.77	1
Mad2l1bp	0.058668	0.110676	0.293	0.213	1

Lgals9	0.058724	-0.19148	0.817	0.818	1
Slc25a24	0.058726	-0.11294	0.317	0.416	1
Dynll1	0.058748	0.205774	0.976	0.985	1
Nupr1	0.058924	-0.27091	0.976	0.984	1
Crk	0.059174	0.156457	0.805	0.709	1
Ndufa4	0.059191	-0.15474	0.854	0.934	1
Efemp1	0.059229	-0.21223	0.817	0.868	1
Tor1b	0.059251	-0.12722	0.488	0.6	1
Tshz3	0.059314	0.146021	0.329	0.237	1
Mrpl52	0.05933	-0.13973	0.817	0.885	1
Ubtd1	0.05937	0.11081	0.402	0.303	1
Myl6	0.059456	-0.17771	1	0.996	1
Bambi	0.059487	0.18476	0.244	0.166	1
Mrfap1	0.059695	0.178104	0.963	0.956	1
Rnf10	0.05993	0.141166	0.878	0.793	1
Ifitm3	0.06017	-0.17718	1	0.999	1
Cuta	0.060378	-0.1611	0.707	0.747	1
Cep350	0.060378	0.102078	0.451	0.354	1
Raf1	0.060394	0.159829	0.732	0.642	1
Apof	0.060487	-0.17148	0.037	0.102	1
Crabp1	0.060595	0.240482	0.512	0.413	1
Pdap1	0.060744	-0.14839	0.793	0.805	1
Ctnnb1	0.060841	0.219095	0.939	0.926	1
Ddx55	0.060975	0.100487	0.207	0.138	1
Ankrd12	0.061294	0.240754	0.841	0.805	1
G3bp1	0.061342	0.216668	0.829	0.741	1
Ugcg	0.06161	0.133936	0.768	0.658	1
Ptges	0.061947	0.118796	0.634	0.541	1
Mrpl41	0.062051	-0.12239	0.244	0.317	1
Gja1	0.062143	-0.21861	0.902	0.946	1
Gins4	0.062705	0.106989	0.28	0.189	1
Cdkn2aip	0.062791	0.159951	0.463	0.355	1
Cpz	0.062863	-0.24013	0.744	0.821	1
Rarg	0.062942	0.136081	0.793	0.665	1
Gas7	0.063143	-0.15467	0.537	0.598	1
Acot9	0.06316	0.102828	0.634	0.534	1
Clptm1l	0.063184	-0.13392	0.646	0.718	1
Selenom	0.06331	-0.15164	0.963	0.956	1
Pnkd	0.063419	-0.13666	0.451	0.521	1
Fip1l1	0.063487	0.134724	0.695	0.552	1
Brd9	0.064104	-0.12619	0.402	0.476	1
Mrpl54	0.064348	-0.14149	0.585	0.616	1
Hdac1	0.064827	0.113577	0.671	0.559	1

Tm4sf1	0.064862	-0.25296	0.463	0.531	1
Spred1	0.064968	0.137836	0.72	0.584	1
Col16a1	0.065125	-0.22676	0.927	0.958	1
Kdm5b	0.065305	0.185433	0.854	0.769	1
Atp5k	0.065355	-0.13503	0.817	0.852	1
Ly96	0.065475	-0.10346	0.22	0.3	1
Mtx1	0.065476	-0.11408	0.232	0.31	1
Cyb5r3	0.065637	-0.18319	0.89	0.872	1
Cpq	0.065667	-0.12731	0.841	0.891	1
mt-Nd4l	0.066206	-0.17421	0.951	0.956	1
Tmem184l	0.066504	-0.10785	0.5	0.595	1
Zfp945	0.066537	0.103814	0.451	0.349	1
Mpp5	0.066992	0.101265	0.646	0.509	1
Lsm2	0.067185	-0.1012	0.232	0.323	1
Ubtf	0.067545	0.119332	0.78	0.689	1
Scamp2	0.067559	-0.11436	0.463	0.547	1
Srm	0.067637	-0.12538	0.451	0.546	1
Cited2	0.067643	0.118182	0.585	0.47	1
Pkn2	0.06788	0.149831	0.854	0.766	1
Cryzl2	0.067918	-0.11079	0.293	0.4	1
Asxl1	0.068023	0.148076	0.415	0.329	1
Bax	0.068105	-0.13286	0.695	0.713	1
Sympk	0.068353	0.117939	0.463	0.376	1
Nedd4	0.068422	-0.12134	1	0.993	1
Prrx2	0.068599	-0.20694	0.744	0.787	1
Lman1	0.068661	-0.17682	0.829	0.894	1
Irx5	0.068744	-0.21842	0.537	0.566	1
Dpm1	0.068837	0.231294	0.707	0.623	1
Slc24a5	0.069013	0.123445	0.134	0.08	1
Ttc19	0.069025	0.111735	0.524	0.424	1
Trp53i13	0.069312	-0.11249	0.415	0.524	1
Ccdc80	0.069375	-0.2508	0.939	0.962	1
Nop2	0.069404	0.156214	0.366	0.282	1
Ipo5	0.069411	0.127108	0.61	0.485	1
Ssna1	0.069436	-0.13743	0.378	0.44	1
Ccbe1	0.069678	-0.18558	0.5	0.595	1
Sec61a1	0.069753	-0.13989	0.854	0.901	1
Tm7sf3	0.069788	-0.1363	0.841	0.901	1
Aprt	0.069817	-0.14559	0.622	0.662	1
Ggh	0.069922	-0.16927	0.671	0.726	1
Ncl	0.070167	0.380517	0.963	0.939	1
B9d2	0.070493	0.132472	0.293	0.215	1
Dgkh	0.070608	0.103493	0.28	0.202	1

Gls	0.070827	0.255301	0.902	0.866	1
Man1a	0.070882	-0.13835	0.939	0.964	1
Nek9	0.070893	-0.11288	0.476	0.537	1
Rps27	0.071014	0.166238	1	0.991	1
Dpysl3	0.071265	-0.1794	0.512	0.6	1
Crcp	0.071336	-0.10307	0.22	0.3	1
Fam208b	0.071377	0.110656	0.439	0.336	1
Stk3	0.071413	-0.13648	0.354	0.437	1
Furin	0.071528	0.19428	0.768	0.699	1
Eif4e2	0.07185	-0.13758	0.598	0.687	1
Snhg18	0.071884	-0.1401	0.793	0.805	1
Tmem150c	0.071967	-0.15011	0.268	0.349	1
Snrpd2	0.072018	-0.15131	0.756	0.776	1
Hdgfl3	0.072033	0.11053	0.5	0.386	1
Slc39a7	0.072428	-0.13318	0.768	0.837	1
Atp5j	0.072468	0.121625	0.963	0.932	1
Cpeb4	0.072547	0.185405	0.793	0.697	1
Pgf	0.072951	0.176742	0.354	0.263	1
Fam214a	0.073048	0.128947	0.427	0.332	1
Fibin	0.07313	-0.21893	0.402	0.469	1
Tram1	0.073194	-0.14017	0.829	0.838	1
Atf2	0.073629	0.14458	0.622	0.531	1
Yif1a	0.073701	-0.17943	0.659	0.671	1
Hoxd8	0.074107	-0.11336	0.329	0.418	1
Col1a2	0.074196	-0.21059	1	1	1
Tle1	0.074487	0.112921	0.683	0.553	1
Bcor	0.074853	0.261059	0.451	0.352	1
Ecm1	0.075128	-0.23249	0.902	0.891	1
Fam96b	0.075327	-0.1201	0.415	0.485	1
Rnase4	0.0757	-0.20124	0.963	0.994	1
Mat2b	0.07603	-0.13657	0.561	0.614	1
Gbf1	0.076081	0.129131	0.646	0.556	1
Lum	0.076222	-0.20572	0.915	0.996	1
Olfm1	0.076255	-0.18371	0.463	0.501	1
Ddx52	0.076391	0.121619	0.402	0.32	1
Kirrel	0.076616	0.110062	0.72	0.629	1
Dsel	0.077185	0.113993	0.622	0.524	1
Adam10	0.077286	0.123746	0.646	0.541	1
Col23a1	0.07742	0.127953	0.537	0.428	1
Sec61g	0.077488	-0.17425	0.951	0.968	1
Cyth2	0.077659	0.100606	0.646	0.534	1
Eif3f	0.078099	-0.13128	0.963	0.952	1
Capg	0.078377	-0.12673	0.854	0.881	1

Ptp4a2	0.078457	0.137543	1	0.949	1
Kdelc2	0.078823	-0.13309	0.354	0.426	1
Oxct1	0.079041	-0.18262	0.805	0.872	1
Atp6v1h	0.079099	0.150307	0.707	0.693	1
Uqcr10	0.079274	-0.13955	0.817	0.825	1
Ebp	0.079288	-0.10964	0.573	0.604	1
Bcorl1	0.07975	0.184632	0.366	0.287	1
2310039H(	0.079783	-0.15	0.402	0.444	1
Ddx47	0.079852	0.105253	0.366	0.272	1
B3gnt9	0.079959	-0.15731	0.561	0.629	1
Prrc2b	0.08022	0.191978	0.817	0.744	1
Mrps34	0.080241	-0.10173	0.341	0.419	1
Sparcl1	0.080423	0.137492	0.366	0.288	1
Kdelr3	0.080833	-0.14027	0.732	0.817	1
Nr3c1	0.080874	0.308342	0.866	0.817	1
Polr2j	0.08091	-0.11518	0.561	0.617	1
Ndufb5	0.081136	-0.16329	0.756	0.793	1
Snw1	0.081381	0.120442	0.573	0.485	1
Yap1	0.081423	0.110507	0.707	0.655	1
Dab2ip	0.081469	0.138977	0.451	0.345	1
Tkt	0.081567	0.12941	0.683	0.562	1
Taldo1	0.081651	-0.11329	0.561	0.629	1
Mndal	0.081934	-0.19471	0.5	0.579	1
Aff3	0.081964	-0.10757	0.72	0.779	1
Tle3	0.082	0.146637	0.573	0.486	1
Dmxl1	0.082447	0.141431	0.573	0.486	1
Nol12	0.082572	-0.11217	0.232	0.307	1
Scand1	0.082653	-0.1742	0.817	0.836	1
Hdac5	0.08291	0.116021	0.561	0.476	1
Aida	0.082997	0.113693	0.744	0.668	1
Hbp1	0.083258	0.129006	0.817	0.764	1
Pip5k1a	0.083506	0.109026	0.732	0.658	1
Ino80	0.083706	0.103317	0.5	0.405	1
Pgm2l1	0.083802	0.206317	0.305	0.231	1
Dcn	0.083881	-0.20023	1	0.999	1
Plec	0.084079	0.280973	0.854	0.833	1
Fut11	0.084227	-0.14728	0.451	0.502	1
S100a11	0.0844	-0.20256	1	0.981	1
Isyna1	0.084835	0.104551	0.232	0.159	1
Ltbp1	0.084901	-0.1224	0.927	0.951	1
Daam1	0.084914	0.123242	0.39	0.316	1
Rlim	0.085498	0.150075	0.524	0.419	1
Fxr2	0.085499	0.111708	0.549	0.459	1

Ndufa8	0.085852	-0.10945	0.634	0.713	1
Gle1	0.086193	-0.10788	0.171	0.25	1
Atcayos	0.086216	0.154188	0.159	0.099	1
Farp1	0.086514	-0.12942	0.329	0.408	1
Plpbp	0.086526	0.133904	0.61	0.495	1
Erp29	0.086536	-0.18405	0.78	0.814	1
Thbs1	0.086669	-0.36536	0.622	0.691	1
Tm9sf3	0.086749	-0.1509	0.878	0.895	1
Igf2bp2	0.08685	0.171119	0.366	0.284	1
Hectd1	0.086989	0.162708	0.878	0.833	1
Iqgap1	0.087176	0.137157	0.927	0.898	1
Ufd1	0.087446	0.110279	0.512	0.419	1
Gps1	0.087607	-0.1153	0.524	0.569	1
Gpr180	0.0879	-0.15241	0.451	0.517	1
Anxa4	0.087945	-0.11935	0.671	0.732	1
Ctcf	0.088027	0.107367	0.695	0.579	1
Plpp3	0.0886	-0.21441	0.951	0.988	1
Atxn7	0.088706	0.13299	0.622	0.517	1
Mkrn1	0.088771	-0.118	0.512	0.579	1
Mfsd11	0.088856	-0.11175	0.585	0.635	1
Phtf1	0.088926	-0.1115	0.22	0.3	1
Tprgl	0.089157	-0.14531	0.854	0.856	1
Dram1	0.089311	-0.15358	0.366	0.428	1
Ywhaq	0.08933	0.14641	0.915	0.898	1
Glo1	0.089571	-0.1366	0.634	0.691	1
Trip11	0.089621	0.11292	0.78	0.674	1
Sumf1	0.089705	-0.12438	0.415	0.479	1
Ccdc28b	0.08986	-0.11468	0.232	0.297	1
Slc15a2	0.090152	0.100089	0.146	0.09	1
Hnrnpf	0.090157	0.136974	0.976	0.914	1
Bach1	0.090232	0.101809	0.768	0.747	1
Clptm1	0.090284	0.102854	0.622	0.552	1
Nfe2l1	0.090334	0.112873	0.793	0.699	1
Gabbr1	0.090407	0.190898	0.39	0.32	1
Tspan4	0.090443	-0.21198	0.72	0.751	1
Hes1	0.090788	0.167965	0.841	0.764	1
Stxbp6	0.090921	-0.10375	0.22	0.298	1
Taok1	0.091337	0.131167	0.89	0.803	1
Abhd14b	0.091468	0.100167	0.439	0.341	1
Mrps21	0.091505	-0.13125	0.768	0.787	1
Dnajc1	0.091739	-0.15279	0.78	0.838	1
Idh1	0.091808	-0.14697	0.744	0.849	1
Ash2l	0.091925	0.102839	0.354	0.272	1

Ctsf	0.091962	-0.10565	0.695	0.761	1
Sbf2	0.091994	0.12064	0.744	0.585	1
Atp13a1	0.092031	-0.10044	0.232	0.311	1
Aldh3a1	0.092143	-0.34182	0.549	0.594	1
Mfap4	0.092292	-0.74371	0.756	0.803	1
Wdr61	0.092436	-0.12124	0.39	0.472	1
Gapvd1	0.092613	0.174443	0.646	0.575	1
Lamp1	0.093223	-0.15136	0.976	0.983	1
Il11ra1	0.093275	-0.13118	0.866	0.895	1
Prr13	0.093292	-0.16094	0.732	0.811	1
Dlst	0.093313	0.118542	0.561	0.454	1
Ly6c1	0.09345	-0.14914	0.866	0.952	1
Dennd1b	0.093738	0.105895	0.366	0.277	1
Fstl1	0.093738	-0.16024	0.951	0.999	1
Psmc3	0.093999	-0.12262	0.683	0.753	1
Ddx6	0.094365	0.144848	0.878	0.895	1
Dtd1	0.094415	-0.10806	0.171	0.245	1
Usmg5	0.094554	0.125609	0.878	0.754	1
Nrep	0.094886	-0.27485	0.39	0.447	1
Timm13	0.095067	-0.11927	0.829	0.831	1
Tnks2	0.095074	-0.11737	0.634	0.702	1
Atp2c1	0.095111	0.147345	0.549	0.472	1
Anxa5	0.095242	-0.13691	0.988	0.996	1
Man2a1	0.095253	-0.16576	0.61	0.723	1
Phf3	0.095285	0.175856	0.793	0.636	1
Uqcrh	0.095289	-0.11609	0.976	0.969	1
Egflam	0.095466	0.101792	0.183	0.121	1
Emc2	0.095568	0.113085	0.488	0.402	1
Comt	0.095609	-0.13642	0.841	0.881	1
Ift27	0.096088	-0.12387	0.549	0.591	1
Prdx5	0.096093	-0.22624	0.817	0.894	1
Ubp1	0.096424	0.120851	0.573	0.475	1
Akap9	0.09653	0.127191	0.78	0.713	1
Smim27	0.096764	-0.12584	0.402	0.473	1
Ik	0.096772	-0.13633	0.488	0.539	1
Med1	0.096793	0.137037	0.61	0.482	1
Ddx3x	0.097238	0.234929	0.963	0.977	1
Rbm42	0.097707	0.140605	0.707	0.624	1
Sec22b	0.097817	-0.13948	0.72	0.774	1
Dnajc21	0.098054	0.153727	0.61	0.512	1
Deptor	0.098111	-0.23773	0.646	0.675	1
Eif4g2	0.098413	0.216926	0.988	0.981	1
Tm2d2	0.098775	-0.12688	0.634	0.713	1

Baiap2	0.098776	0.156146	0.683	0.613	1
Dido1	0.098845	0.17775	0.488	0.435	1
Jtb	0.098901	-0.1291	0.683	0.723	1
Cnih4	0.099125	-0.13992	0.476	0.541	1
Pura	0.099288	0.148232	0.805	0.801	1
Arf1	0.099302	-0.13176	0.939	0.948	1
Ssr3	0.099399	-0.15637	0.878	0.948	1
Arhgap6	0.099559	-0.10494	0.171	0.243	1
2010109A1	0.099901	0.119572	0.134	0.083	1
Cop1	0.100007	0.145824	0.866	0.803	1
Ier2	0.100391	0.343473	0.976	0.948	1
Adk	0.100749	-0.10183	0.768	0.836	1
R3hdm4	0.10076	-0.11527	0.756	0.76	1
Nrbf2	0.100884	-0.12798	0.28	0.339	1
Ugp2	0.100931	0.155302	0.805	0.748	1
Galnt15	0.101359	0.113101	0.256	0.182	1
Ddx39b	0.101431	-0.13808	0.598	0.674	1
Taf10	0.101477	-0.1277	0.744	0.761	1
Gatad2a	0.102028	0.111023	0.61	0.508	1
Prorsd1	0.102435	-0.11176	0.378	0.447	1
Rbm8a	0.102656	0.110159	0.829	0.697	1
Prelid1	0.10292	-0.13678	0.72	0.747	1
March7	0.103008	0.114003	0.646	0.622	1
Ppfibp1	0.103171	-0.13728	0.841	0.881	1
Sept11	0.103607	0.32538	0.463	0.406	1
Fam3c	0.103767	-0.15464	0.634	0.675	1
Ctr9	0.103863	0.124782	0.378	0.287	1
Fzd2	0.104286	-0.11909	0.829	0.787	1
Ggt5	0.104499	0.122086	0.415	0.326	1
Chpt1	0.104524	-0.14064	0.671	0.683	1
Map4	0.104551	0.106562	0.768	0.643	1
Gstm2	0.104671	-0.15483	0.695	0.741	1
Ufc1	0.104758	-0.1431	0.817	0.822	1
Fnbp1	0.104759	0.116081	0.695	0.59	1
Sec61b	0.104919	-0.18019	0.939	0.942	1
Gse1	0.10503	0.164998	0.329	0.255	1
Rheb	0.105082	-0.12709	0.878	0.84	1
Maged2	0.105202	-0.12626	0.537	0.627	1
Svbp	0.105294	-0.12755	0.573	0.626	1
Cyp2f2	0.105473	-0.21404	0.756	0.828	1
Col5a3	0.105549	-0.11636	0.78	0.9	1
Rabac1	0.106077	-0.10337	0.963	0.962	1
Msrb3	0.106223	-0.10758	0.402	0.475	1

B230217C:	0.106571	0.138025	0.329	0.259	1
Rnase1	0.106979	-0.13851	0.085	0.146	1
Psmb10	0.107011	-0.14881	0.341	0.402	1
Ahnak2	0.107046	-0.11389	0.646	0.719	1
Anks1	0.107282	0.114455	0.402	0.314	1
Cd81	0.107859	-0.14027	0.988	0.983	1
Ablim1	0.108054	-0.12888	0.744	0.809	1
Erg28	0.108326	-0.12772	0.268	0.322	1
Ran	0.108534	0.139107	0.854	0.774	1
Cpe	0.108639	-0.27786	0.768	0.798	1
Ppp1ca	0.108674	-0.14686	0.817	0.875	1
Prelp	0.108851	-0.1096	0.683	0.747	1
Lmo4	0.109022	0.478746	0.72	0.655	1
Arfip2	0.10923	-0.10026	0.439	0.504	1
Mpnd	0.109254	-0.12962	0.598	0.601	1
Cttn	0.109255	-0.16667	0.707	0.722	1
Fubp1	0.1095	0.129812	0.744	0.703	1
Ube2n	0.109882	0.103063	0.817	0.751	1
Taf12	0.110007	-0.10129	0.232	0.295	1
Dnaja2	0.110008	0.187962	0.829	0.783	1
Clip1	0.110082	-0.14659	0.744	0.767	1
Kat2b	0.110588	0.11248	0.634	0.549	1
Smim1	0.110968	-0.1283	0.317	0.386	1
Zcchc17	0.1111	-0.10084	0.39	0.488	1
Akirin2	0.111192	0.155294	0.671	0.581	1
Tmem159	0.111908	0.138696	0.72	0.639	1
Srsf6	0.112195	0.140002	0.89	0.841	1
Usp47	0.112423	0.125129	0.659	0.584	1
Ccl11	0.112603	-0.17166	0.354	0.438	1
Cblb	0.112696	0.1131	0.585	0.467	1
Derl2	0.112815	-0.10031	0.427	0.498	1
Elob	0.112958	-0.11969	0.951	0.968	1
Ms4a4d	0.113171	-0.15957	0.634	0.665	1
Ak1	0.11355	-0.1557	0.39	0.448	1
Smoc2	0.113963	-0.19207	0.829	0.934	1
Lsamp	0.114329	-0.15019	0.427	0.501	1
Cdv3	0.115036	0.232697	0.793	0.799	1
Psma1	0.1151	0.1274	0.695	0.581	1
Tnpo3	0.115339	0.105634	0.524	0.467	1
Fth1	0.11574	-0.16755	1	1	1
Ccar1	0.115897	0.210115	0.768	0.68	1
Cgref1	0.115971	-0.1573	0.463	0.511	1
Sh3pxd2a	0.116072	-0.13753	0.805	0.917	1

Cox6b1	0.116113	-0.14342	0.89	0.902	1
Wapl	0.116508	0.135242	0.646	0.616	1
Ykt6	0.116588	-0.13381	0.622	0.642	1
Plpp1	0.116663	-0.15769	0.756	0.78	1
Mrps28	0.116755	-0.11052	0.451	0.517	1
Ncstn	0.116908	-0.10899	0.61	0.649	1
Osr2	0.117749	-0.11512	0.756	0.814	1
Klf3	0.117924	0.154983	0.841	0.793	1
Sbno2	0.118246	0.107605	0.768	0.713	1
Gdi2	0.118307	-0.12861	0.854	0.934	1
Atp1a1	0.118517	0.109415	0.854	0.754	1
Ankrd26	0.118554	0.218159	0.195	0.141	1
Wdfy3	0.118574	0.165805	0.585	0.534	1
Psmb4	0.118582	-0.14404	0.866	0.862	1
Klf7	0.119053	0.146383	0.573	0.493	1
Spg20	0.119842	-0.11841	0.354	0.419	1
Senp6	0.120108	0.210578	0.817	0.799	1
Ufsp2	0.120845	-0.12327	0.451	0.504	1
Insig1	0.121088	0.204422	0.72	0.652	1
Meaf6	0.121338	-0.11293	0.207	0.281	1
Tril	0.121574	-0.16438	0.268	0.33	1
Il1r2	0.122191	-0.15365	0.817	0.918	1
Prdx6	0.122756	-0.14452	0.866	0.956	1
Gosr2	0.122852	-0.11229	0.476	0.555	1
Rad50	0.123162	-0.13048	0.22	0.277	1
Rtf1	0.123367	0.15967	0.805	0.672	1
Itm2c	0.123404	-0.11701	0.939	0.955	1
Atp6v1e1	0.123426	0.113097	0.829	0.755	1
Pnisr	0.123565	0.188202	0.561	0.444	1
Cnih1	0.125389	-0.1405	0.805	0.824	1
Cpxm1	0.125676	-0.19338	0.829	0.863	1
Cisd2	0.125846	-0.16061	0.707	0.697	1
Rps21	0.126071	0.163291	1	0.999	1
Dse	0.126611	-0.13842	0.646	0.658	1
Fam171b	0.126636	-0.12051	0.329	0.389	1
Tomm5	0.126678	-0.14517	0.659	0.656	1
Prpf38a	0.12672	0.117775	0.329	0.271	1
Dact3	0.127242	0.114759	0.354	0.277	1
Kctd17	0.127442	-0.12465	0.427	0.479	1
Lamtor4	0.128	-0.11959	0.695	0.734	1
Nfia	0.128031	-0.1059	0.976	0.968	1
Txndc17	0.128196	-0.10042	0.841	0.865	1
Adamts5	0.128441	-0.19818	0.634	0.675	1

AC149090.	0.128863	0.112152	0.805	0.735	1
Slc39a6	0.129865	0.130326	0.683	0.578	1
Steap3	0.129912	-0.11372	0.695	0.755	1
Igfbp7	0.130025	-0.13572	1	0.991	1
Ezh2	0.130381	0.128106	0.305	0.227	1
Nme1	0.130593	-0.15157	0.671	0.7	1
Mrpl33	0.131156	0.173169	0.89	0.849	1
Emilin1	0.13122	-0.1418	0.585	0.632	1
Tanc2	0.13174	0.107335	0.415	0.341	1
Fkbp11	0.131822	-0.15025	0.268	0.338	1
Aspg	0.132704	-0.1268	0.232	0.301	1
Gtf2h5	0.132729	-0.13817	0.707	0.799	1
Tuba1b	0.132773	0.139933	0.902	0.836	1
Ncor2	0.133291	0.126347	0.707	0.643	1
Ramp1	0.133731	-0.12724	0.256	0.316	1
Fam180a	0.133738	-0.2039	0.366	0.445	1
Cks1b	0.133897	0.169262	0.354	0.279	1
Rsrc2	0.134076	0.140313	0.817	0.713	1
Bace2	0.134703	-0.11223	0.671	0.716	1
Foxn2	0.135225	0.100518	0.61	0.469	1
Tcaf1	0.135952	-0.117	0.476	0.527	1
S100a10	0.136137	0.104821	1	0.996	1
Mrps14	0.136853	-0.11076	0.598	0.617	1
Ywhag	0.137409	0.102331	0.671	0.582	1
Serpinb6a	0.137455	-0.15628	0.963	0.993	1
Rcn2	0.137538	0.135775	0.829	0.739	1
Fuca1	0.138033	-0.11404	0.841	0.856	1
Slco3a1	0.138957	-0.10676	0.439	0.515	1
Ism1	0.139418	-0.24831	0.512	0.527	1
Amd2	0.140071	0.119823	0.341	0.279	1
Pros1	0.140182	-0.14665	0.817	0.885	1
Cr1l	0.140323	-0.14892	0.78	0.771	1
Limd1	0.140396	0.185981	0.805	0.75	1
Cd9	0.140416	0.214622	0.939	0.933	1
Ddx50	0.140859	0.142909	0.744	0.686	1
Glud1	0.14099	0.121443	0.939	0.936	1
Smarca5	0.141024	0.182839	0.793	0.738	1
Hdac3	0.141171	-0.12389	0.39	0.459	1
Cldn10	0.141798	-0.17656	0.78	0.799	1
Naa38	0.141893	-0.10596	0.427	0.505	1
Snapin	0.14194	-0.13682	0.598	0.6	1
Entpd1	0.142631	-0.15077	0.402	0.445	1
Abca8b	0.142676	0.103502	0.122	0.077	1

Decr1	0.14289	-0.10354	0.354	0.393	1
Nucb2	0.143217	-0.13095	0.622	0.649	1
Fastk	0.143238	-0.12534	0.378	0.429	1
Gpc3	0.143679	-0.21144	0.232	0.159	1
Dmxl2	0.143697	0.103273	0.195	0.14	1
Pigx	0.143958	-0.12988	0.402	0.428	1
Lrp5	0.144162	0.119205	0.28	0.217	1
Sec13	0.144482	-0.1278	0.634	0.718	1
Syne1	0.144544	0.149156	0.61	0.533	1
Lysmd2	0.144555	-0.11457	0.402	0.463	1
Vcl	0.144776	0.124737	0.439	0.374	1
Cops9	0.145091	-0.12139	0.707	0.824	1
Capn2	0.145194	-0.12447	0.671	0.744	1
Mga	0.145681	0.133643	0.72	0.694	1
Msl3	0.146251	-0.1204	0.341	0.392	1
Sgms2	0.146342	-0.10311	0.427	0.469	1
Yrdc	0.146422	0.137839	0.476	0.399	1
Rhoc	0.146569	-0.16269	0.793	0.811	1
Pgrmc2	0.146584	-0.11152	0.622	0.643	1
Lrrtm3	0.147004	-0.21012	0.549	0.588	1
Lta4h	0.147011	-0.11786	0.598	0.642	1
H2afx	0.14718	0.108559	0.305	0.24	1
Pcm1	0.147362	0.12328	0.61	0.54	1
Eef1b2	0.147619	-0.10695	0.976	0.988	1
Cdc16	0.148235	-0.10046	0.463	0.537	1
Slfn5	0.148262	-0.11364	0.305	0.368	1
Rsbn1	0.14898	0.174526	0.488	0.443	1
Rps6ka4	0.149116	-0.10669	0.415	0.456	1
Gabarapl2	0.149281	0.127566	0.89	0.833	1
Lgals1	0.149318	-0.12275	1	0.999	1
Ddt	0.149499	-0.11498	0.646	0.683	1
Ptpn1	0.149582	0.106366	0.5	0.41	1
Oaf	0.149677	0.151319	0.89	0.846	1
Ndufb9	0.149917	-0.12905	0.793	0.808	1
Efemp2	0.150027	-0.1126	0.659	0.731	1
Timm17b	0.150146	-0.15567	0.439	0.475	1
Cdip1	0.15017	0.122171	0.451	0.38	1
Scaf1	0.150499	0.110353	0.5	0.448	1
Ric8a	0.150817	-0.10064	0.305	0.38	1
Fam71a	0.151217	-0.14362	0.146	0.201	1
Syf2	0.151235	0.110564	0.817	0.712	1
Sfxn1	0.151417	-0.1012	0.366	0.44	1
Lamtor5	0.151516	-0.12724	0.659	0.668	1

Fgfr4	0.151771	-0.11117	0.451	0.517	1
Irx2	0.151861	0.13791	0.829	0.747	1
Capza2	0.151907	0.101451	0.939	0.852	1
Uqcrq	0.151941	-0.11241	0.793	0.847	1
Herc2	0.15212	0.119604	0.72	0.624	1
Yipf3	0.152278	-0.11843	0.817	0.822	1
Klf6	0.15274	0.19712	0.988	0.949	1
Mrps16	0.153132	-0.11454	0.537	0.574	1
P2rx4	0.153252	-0.14841	0.646	0.646	1
Tef	0.153722	0.13134	0.5	0.435	1
Nf1	0.153728	0.10664	0.573	0.509	1
Scd1	0.154046	-0.14888	0.744	0.776	1
Gsn	0.154734	-0.1829	1	1	1
Xiap	0.155109	0.1421	0.659	0.595	1
Pink1	0.155183	-0.12695	0.683	0.67	1
Id1	0.155612	0.203658	0.305	0.236	1
Lpar1	0.156835	-0.10313	0.841	0.876	1
Cspg5	0.156909	0.128675	0.159	0.111	1
Ntn1	0.157063	-0.1382	0.72	0.79	1
Snx7	0.157325	-0.13042	0.5	0.553	1
Mafg	0.157646	0.151373	0.659	0.627	1
Dyrk1a	0.15795	0.106046	0.61	0.544	1
Dmtf1	0.158548	0.147949	0.378	0.31	1
Baz2b	0.158576	0.118678	0.732	0.691	1
Kif1b	0.159351	0.106657	0.866	0.801	1
Ntrk2	0.159622	-0.14107	0.244	0.301	1
St13	0.159789	0.142568	0.866	0.834	1
Ube2k	0.160146	0.131424	0.841	0.777	1
Rif1	0.16031	0.148847	0.476	0.403	1
Msl1	0.16042	0.130407	0.72	0.603	1
Kcmf1	0.160551	0.163538	0.732	0.681	1
Cmtm3	0.160845	-0.15253	0.866	0.914	1
Myadm	0.161018	0.195531	0.866	0.809	1
Per1	0.161191	0.129391	0.537	0.464	1
Aff1	0.161513	0.136138	0.732	0.63	1
Casp4	0.161726	-0.13629	0.402	0.499	1
Esyt1	0.161791	0.155932	0.512	0.498	1
Abca9	0.162403	0.13928	0.305	0.239	1
Scpep1	0.162479	-0.10691	0.866	0.91	1
Atp5f1	0.162554	-0.13444	0.841	0.878	1
Cnot6l	0.162577	0.135352	0.634	0.537	1
Dlg4	0.162779	0.185305	0.598	0.544	1
Enpp2	0.163175	0.416333	0.78	0.722	1

Agt	0.163422	0.16728	0.402	0.329	1
Cfdp1	0.164407	-0.11578	0.695	0.747	1
Api5	0.164507	0.148532	0.561	0.492	1
Bola3	0.165144	-0.10641	0.427	0.505	1
Numa1	0.16537	0.140776	0.61	0.543	1
Hdgf	0.165406	0.166921	0.951	0.872	1
Nadk	0.166078	-0.10885	0.476	0.518	1
a	0.168757	0.418711	0.305	0.266	1
Zfp148	0.169054	0.110057	0.683	0.632	1
Marcks	0.169163	-0.14925	0.988	0.969	1
Sfxn3	0.169249	-0.13418	0.634	0.665	1
Tax1bp3	0.169409	-0.10242	0.598	0.626	1
Ndrg3	0.169667	-0.1117	0.427	0.464	1
Thbs3	0.170027	-0.11466	0.524	0.563	1
Kansl1l	0.170108	0.126239	0.5	0.45	1
Abhd2	0.170141	0.138592	0.549	0.512	1
Wbp1l	0.170238	-0.14002	0.622	0.629	1
Pianp	0.170498	-0.13222	0.329	0.384	1
Adam12	0.170837	-0.1447	0.451	0.489	1
Uqcrb	0.17275	-0.12194	0.841	0.836	1
Rpl35	0.173031	0.123766	0.988	0.977	1
Phax	0.17315	-0.11457	0.39	0.434	1
Col4a3	0.173599	0.306698	0.244	0.189	1
Igfbp4	0.173927	0.133508	0.28	0.207	1
Slc48a1	0.174164	-0.14215	0.72	0.709	1
Gm1673	0.176028	0.15193	0.305	0.246	1
Gdi1	0.177091	0.103148	0.427	0.367	1
Creb3l3	0.177393	-0.10949	0.463	0.505	1
Gm42418	0.177809	-0.30176	1	1	1
Abcf1	0.177825	-0.13819	0.512	0.54	1
Naga	0.177847	-0.11089	0.573	0.642	1
Tpp2	0.178356	0.122669	0.671	0.613	1
Prpf39	0.179018	0.103163	0.512	0.419	1
Ufm1	0.179125	-0.11422	0.561	0.587	1
Cox4i1	0.179252	0.184859	1	0.985	1
Myl12a	0.180099	-0.16498	0.939	0.974	1
Mrpl30	0.180256	-0.12848	0.671	0.677	1
Tcf7l1	0.180598	0.105598	0.598	0.524	1
C1qtnf2	0.181137	-0.11684	0.354	0.41	1
Epc2	0.18173	0.14542	0.561	0.511	1
Anp32e	0.181852	0.130916	0.622	0.543	1
Trove2	0.182289	-0.11529	0.488	0.527	1
Gda	0.183115	-0.12844	0.72	0.79	1

D8Ertd738	0.183612	-0.10406	0.866	0.865	1
Pcna	0.183883	0.221949	0.573	0.523	1
Cox6a1	0.184492	-0.10482	0.89	0.841	1
Hoxc6	0.184768	-0.11317	0.427	0.492	1
Crispld2	0.185706	-0.29283	0.976	0.955	1
Loxl1	0.186055	-0.14505	0.878	0.872	1
Gpc4	0.186135	-0.13655	0.841	0.84	1
Pld3	0.186497	-0.11424	0.695	0.707	1
Mphosph8	0.18776	0.146324	0.39	0.326	1
Tvp23b	0.188367	-0.13274	0.561	0.598	1
Set	0.188594	0.401914	0.89	0.792	1
Selenof	0.188711	-0.10416	0.963	0.981	1
Unc50	0.189013	-0.11118	0.5	0.524	1
U2surp	0.189126	0.101624	0.573	0.525	1
Scnn1b	0.189205	-0.20909	0.134	0.183	1
Map4k4	0.189882	0.164179	0.927	0.86	1
Atp2b4	0.191373	0.181491	0.683	0.632	1
Commd3	0.191648	-0.1522	0.695	0.686	1
Esr1	0.192521	-0.13122	0.61	0.674	1
mt-Nd2	0.192904	-0.14859	1	0.999	1
Gnl1	0.193081	0.129626	0.488	0.422	1
Mrps24	0.193214	-0.10015	0.61	0.668	1
Omd	0.193409	-0.11527	0.293	0.358	1
St3gal4	0.193578	-0.12424	0.512	0.555	1
Smim14	0.193899	-0.10943	0.89	0.932	1
Acot13	0.193899	-0.10359	0.329	0.39	1
Ctdsp2	0.19395	0.113001	0.915	0.9	1
Jak2	0.194036	0.153778	0.451	0.378	1
Rpl36a	0.194705	0.110114	0.963	0.977	1
Psenen	0.195827	-0.11736	0.902	0.841	1
Nudcd2	0.19736	-0.12974	0.476	0.491	1
Hspa1b	0.198124	0.787277	0.793	0.777	1
Pbx1	0.198431	-0.10615	0.963	0.959	1
Gfpt1	0.198463	-0.10966	0.683	0.757	1
Mtpn	0.200957	-0.11599	0.756	0.777	1
Aes	0.201421	-0.10642	0.89	0.913	1
Casp1	0.201505	-0.10272	0.268	0.322	1
Eif2s3y	0.202095	0.105721	0.537	0.475	1
Myo10	0.202952	0.193543	0.256	0.204	1
Rybp	0.203893	0.140389	0.622	0.568	1
Rpl10a	0.204008	-0.12868	1	0.991	1
Tmem100	0.204433	0.102797	0.488	0.406	1
Pltp	0.204504	-0.23526	0.549	0.603	1

St7	0.204653	0.10601	0.293	0.236	1
Tfg	0.205208	-0.11804	0.659	0.659	1
Tnmd	0.205343	0.234224	0.39	0.311	1
Rbpj	0.205526	0.120167	0.756	0.694	1
Sh3glb1	0.20565	-0.11845	0.939	0.94	1
Gm26518	0.205841	0.20519	0.402	0.345	1
Ccdc12	0.205942	-0.11685	0.756	0.769	1
Aspn	0.206112	0.241374	0.951	0.934	1
Sdhb	0.206248	-0.10269	0.646	0.642	1
Pbrm1	0.206445	0.103262	0.902	0.825	1
P3h1	0.206508	-0.1205	0.598	0.598	1
Lgals3bp	0.206556	-0.17005	0.354	0.408	1
Sh3kbp1	0.207258	-0.10706	0.524	0.601	1
Ctsk	0.208444	-0.13337	1	0.996	1
Fam162a	0.208777	-0.10094	0.451	0.509	1
Rnf187	0.20916	-0.1041	0.659	0.738	1
Usf2	0.21097	0.108536	0.72	0.661	1
Fosb	0.212275	0.141706	1	0.99	1
Erlec1	0.212412	-0.1259	0.732	0.716	1
Ralb	0.213638	-0.10612	0.451	0.493	1
Kmt2a	0.2138	0.157968	0.866	0.741	1
Cdk5rap3	0.214388	-0.10239	0.573	0.606	1
Lrp10	0.21476	-0.12524	0.707	0.713	1
Ndn	0.214804	-0.13548	0.793	0.77	1
Stag2	0.215267	0.109753	0.732	0.651	1
Rpl31	0.215841	-0.10139	0.939	0.956	1
Smarcd2	0.216718	-0.1016	0.524	0.557	1
Col18a1	0.218015	-0.10779	0.695	0.758	1
Fbxw11	0.219021	0.123789	0.598	0.54	1
Lamb2	0.220285	-0.10489	0.927	0.924	1
Il3ra	0.22105	-0.10659	0.476	0.521	1
Hmgb1	0.221077	0.101875	1	0.983	1
Adamtsl5	0.221744	-0.10782	0.634	0.638	1
Pfn1	0.222061	-0.17647	0.963	0.964	1
Eif3a	0.222091	0.121383	0.89	0.87	1
Mapk14	0.223691	-0.11366	0.415	0.461	1
Zfp503	0.224071	-0.11837	0.549	0.603	1
Scaf8	0.225586	0.10067	0.451	0.409	1
Iqsec1	0.225968	0.166257	0.476	0.419	1
Myof	0.226664	-0.12981	0.707	0.721	1
Col14a1	0.226825	-0.26516	0.354	0.412	1
Rab14	0.22843	0.106392	0.902	0.786	1
Reep5	0.228701	-0.10897	0.939	0.965	1

Ndufb10	0.228736	-0.10758	0.78	0.801	1
Chpf2	0.228815	-0.14106	0.524	0.546	1
Srf	0.228955	0.117396	0.585	0.517	1
Dusp7	0.232124	0.18776	0.427	0.355	1
Smim11	0.23225	-0.11127	0.622	0.619	1
Atrx	0.232935	0.182475	0.915	0.828	1
Evi2a	0.233953	-0.11379	0.354	0.397	1
Irak3	0.235113	0.156901	0.634	0.601	1
Cdk9	0.235374	0.163509	0.573	0.518	1
Maml3	0.23565	0.175344	0.329	0.266	1
Chd6	0.236476	0.13921	0.524	0.498	1
Spp1	0.236633	-0.30534	0.122	0.08	1
Tagln	0.238326	-0.13983	0.159	0.112	1
Tbck	0.238366	0.105017	0.305	0.249	1
Ptpru	0.238971	0.100923	0.171	0.125	1
Zdhhc18	0.239316	-0.11073	0.427	0.469	1
Psmd8	0.239472	-0.11071	0.902	0.905	1
Gatd1	0.239934	-0.10068	0.512	0.556	1
Zcrb1	0.241245	-0.10618	0.634	0.643	1
Use1	0.242034	-0.10054	0.768	0.766	1
Ndufb8	0.242316	-0.12569	0.671	0.689	1
Flot2	0.243295	-0.10653	0.451	0.501	1
Tubb2a	0.244038	0.199305	0.805	0.78	1
Bfar	0.244519	-0.11409	0.537	0.525	1
Luc7l2	0.244928	0.156694	0.939	0.892	1
Lemd2	0.245144	0.100355	0.805	0.755	1
Swi5	0.246793	-0.14763	0.951	0.898	1
Inf2	0.247232	-0.10111	0.634	0.699	1
Ssb	0.247683	0.188007	0.902	0.828	1
Gfpt2	0.251199	0.127676	0.695	0.654	1
Gramd1a	0.251393	0.156248	0.366	0.335	1
Mob1a	0.251516	-0.10711	0.695	0.725	1
Ddb1	0.253999	-0.10391	0.841	0.888	1
Dcaf8	0.254091	-0.12333	0.622	0.619	1
Zfp638	0.254849	0.10076	0.598	0.534	1
Mettl9	0.255486	-0.11949	0.622	0.646	1
Slc29a1	0.25566	-0.15178	0.817	0.817	1
Fam46c	0.255666	0.149845	0.622	0.552	1
Aldh1a1	0.256009	-0.15975	0.72	0.764	1
Ddah1	0.256063	-0.22324	0.305	0.36	1
Klhl5	0.256358	-0.12003	0.28	0.309	1
Serp1	0.256481	-0.10845	0.854	0.87	1
Nktr	0.256512	0.100316	0.902	0.868	1

Nrp1	0.256632	-0.10775	0.585	0.654	1
Psma7	0.256835	-0.12511	0.78	0.83	1
Raph1	0.257137	0.155012	0.439	0.419	1
Ubr2	0.257298	0.120514	0.659	0.601	1
Ppp1r14b	0.258877	-0.16108	0.963	0.952	1
Rdx	0.259059	-0.12753	0.854	0.873	1
Csf1r	0.259681	-0.11151	0.329	0.37	1
Ube2l3	0.259853	-0.11187	0.72	0.757	1
Ube2g2	0.260042	-0.11958	0.549	0.584	1
mt-Atp8	0.26006	-0.13975	0.707	0.739	1
Gna11	0.260222	-0.10355	0.72	0.712	1
Lgals4	0.261249	0.129213	0.159	0.122	1
Bzw1	0.26156	0.117719	0.854	0.843	1
Rora	0.261739	0.126517	0.951	0.936	1
Tcerg1	0.262361	0.119276	0.463	0.383	1
Shc1	0.26303	0.114957	0.683	0.654	1
Zfp950	0.264136	-0.10037	0.183	0.214	1
Fkbp1a	0.264416	-0.13906	0.915	0.934	1
Srp14	0.264541	-0.10482	0.805	0.821	1
Meis2	0.264595	0.103175	0.598	0.531	1
Ripk1	0.265648	0.104758	0.671	0.613	1
Tmem9b	0.26615	-0.11463	0.463	0.488	1
Pgr	0.26618	-0.17487	0.598	0.633	1
Pla1a	0.267841	0.283373	0.354	0.309	1
0610012G(	0.269541	-0.10306	0.585	0.592	1
Cspp1	0.270078	0.121034	0.366	0.323	1
Rps19	0.27016	0.110004	1	0.993	1
Rplp2	0.270274	0.109092	0.988	0.985	1
Odc1	0.270522	0.125101	0.598	0.6	1
Psmb1	0.270747	-0.11547	0.866	0.908	1
Srprb	0.272034	0.104657	0.463	0.402	1
Pde8a	0.272079	-0.10602	0.707	0.7	1
P3h4	0.272805	-0.12884	0.695	0.694	1
Itgb5	0.273097	-0.1293	0.963	0.94	1
Ythdf2	0.273934	0.138701	0.488	0.435	1
Zeb1	0.274085	-0.10089	0.598	0.6	1
F2r	0.274333	0.100461	0.293	0.245	1
Clec2d	0.274349	-0.15079	0.39	0.421	1
Rasl11a	0.275138	0.10323	0.159	0.118	1
Pdrg1	0.275145	-0.15975	0.476	0.507	1
Slc27a1	0.276323	-0.10297	0.537	0.549	1
Sacm1l	0.277177	0.104237	0.439	0.412	1
Hnrnpm	0.278522	0.121487	0.829	0.792	1

Ppia	0.280108	0.134422	1	0.997	1
Rras	0.280269	-0.10815	0.732	0.725	1
Ctsd	0.280456	-0.10478	0.988	0.974	1
Ebf1	0.2815	0.127289	0.976	0.958	1
Coro1b	0.284881	-0.1172	0.561	0.56	1
Hmg20b	0.28733	-0.11424	0.585	0.62	1
Mrpl58	0.288114	-0.10627	0.366	0.406	1
Snap23	0.288135	-0.11619	0.537	0.559	1
Glipr2	0.288452	-0.12313	0.22	0.258	1
Vmp1	0.28921	-0.11182	0.695	0.71	1
Fxyd6	0.292288	-0.2615	0.183	0.223	1
P4ha1	0.292995	-0.1195	0.707	0.735	1
Aldoa	0.293102	-0.11582	0.902	0.932	1
Aplp2	0.294371	0.129257	0.988	0.946	1
Ackr3	0.294845	0.233391	0.817	0.857	1
Mrps12	0.295312	-0.10171	0.463	0.498	1
Aimp1	0.295964	0.119205	0.634	0.636	1
Tab2	0.296107	0.112886	0.854	0.785	1
Hnrnpk	0.298648	0.12673	0.976	0.964	1
H2afv	0.299446	-0.12693	0.756	0.76	1
Rnf6	0.299552	0.122001	0.573	0.493	1
Slc30a5	0.300951	-0.1112	0.451	0.469	1
Pcnp	0.301797	0.116869	0.793	0.766	1
Prss23	0.305616	-0.18858	0.512	0.55	1
Myl9	0.308618	-0.12549	0.207	0.157	1
Cd248	0.308931	-0.15191	0.841	0.833	1
Lamtor1	0.309734	-0.14807	0.744	0.689	1
Sept8	0.313116	-0.10087	0.817	0.812	1
Rbms1	0.314936	-0.11256	0.951	0.91	1
Igfbp5	0.315832	-0.10392	0.793	0.869	1
Itga1	0.316027	0.12386	0.22	0.186	1
Scd2	0.316886	-0.17809	0.72	0.731	1
Akap13	0.317758	0.160106	0.963	0.914	1
Pla2r1	0.320172	0.137653	0.439	0.403	1
Mrgprf	0.323589	-0.10395	0.39	0.421	1
Gpm6b	0.324271	-0.12612	0.561	0.606	1
Golga3	0.324577	-0.12896	0.488	0.493	1
Nid1	0.327133	-0.22942	0.841	0.822	1
H13	0.330097	-0.12249	0.768	0.787	1
D1Ertd622	0.331282	0.154773	0.122	0.09	1
Polr2l	0.332334	0.278153	0.512	0.434	1
Twist1	0.332343	0.116147	0.939	0.956	1
Grina	0.334122	-0.12085	0.854	0.817	1

Tcf7	0.33711	0.109591	0.159	0.125	1
Slc25a1	0.337634	-0.10096	0.402	0.434	1
Ccng2	0.337667	-0.10299	0.573	0.56	1
Nrp2	0.33928	0.124474	0.634	0.606	1
Nagk	0.341703	-0.13068	0.585	0.571	1
Ago2	0.341712	0.121411	0.744	0.681	1
Tmed5	0.34417	0.325503	0.585	0.584	1
Ccl7	0.344233	-0.17859	0.756	0.748	1
Galnt17	0.344609	-0.12944	0.256	0.281	1
Slc9a1	0.345301	-0.11163	0.439	0.445	1
Psma4	0.345545	-0.13302	0.829	0.771	1
Arntl	0.350629	0.103074	0.634	0.559	1
Gspt1	0.350648	0.151009	0.671	0.672	1
Slc25a5	0.351221	0.167051	0.878	0.87	1
Ampd3	0.351289	0.123342	0.5	0.44	1
Dda1	0.351832	0.103316	0.476	0.457	1
N4bp2	0.352876	0.131804	0.39	0.357	1
Spry1	0.35296	0.112002	0.402	0.358	1
Cst3	0.353642	0.117841	1	0.983	1
Pdgfrb	0.353675	-0.14267	0.866	0.833	1
Myo6	0.354318	-0.11037	0.683	0.63	1
Ifi205	0.354951	0.199896	0.439	0.381	1
Pdzk1ip1	0.356851	-0.11101	0.256	0.288	1
Lsp1	0.357256	-0.13684	0.659	0.635	1
Rexo2	0.357752	-0.13513	0.805	0.805	1
Tgfb1	0.360734	0.161048	0.585	0.552	1
Raly	0.360916	0.111224	0.866	0.815	1
Rpl22l1	0.363378	-0.1313	0.963	0.943	1
C1qtnf3	0.366142	-0.10304	0.598	0.689	1
Anxa2	0.366333	-0.10909	0.963	0.971	1
Bmp7	0.366502	0.184344	0.524	0.476	1
Ehmt1	0.368455	0.106826	0.463	0.37	1
Svep1	0.370335	-0.10735	0.841	0.865	1
Engase	0.370791	0.127141	0.146	0.115	1
Pi15	0.371512	0.152822	0.244	0.214	1
Gbp2	0.373042	-0.15348	0.317	0.344	1
Ormdl3	0.374138	-0.1039	0.732	0.767	1
Fgfr1op2	0.37448	-0.11205	0.707	0.678	1
Gclc	0.375251	0.110647	0.585	0.528	1
Wisp2	0.376265	-0.23298	0.244	0.288	1
Cdr2	0.37684	-0.13316	0.732	0.721	1
Penk	0.376958	-0.16798	0.317	0.345	1
Papss1	0.37937	-0.11429	0.476	0.495	1

Ndrg4	0.381862	-0.12529	0.598	0.591	1
Tcf7l2	0.382667	0.141525	0.695	0.718	1
Ube2h	0.384688	0.113509	0.829	0.785	1
Grem1	0.385172	-0.28467	0.293	0.326	1
Gng11	0.386742	-0.22789	0.585	0.61	1
Itm2a	0.388248	-0.14025	0.268	0.323	1
Bcl11b	0.392186	-0.11065	0.537	0.572	1
Pamr1	0.394094	-0.11421	0.329	0.37	1
Phldb2	0.394125	-0.22054	0.622	0.627	1
Park7	0.394652	-0.10492	0.878	0.854	1
Rbm5	0.394966	0.172738	0.634	0.613	1
Selenot	0.396399	-0.10196	0.646	0.635	1
Rgl1	0.397171	-0.10031	0.622	0.623	1
Mgat2	0.398416	0.10966	0.695	0.624	1
Enpp3	0.398525	-0.10817	0.22	0.24	1
Abca8a	0.398526	0.310236	0.268	0.25	1
Ptx3	0.401179	0.295948	0.183	0.236	1
Dynlt3	0.402104	-0.1041	0.61	0.608	1
Smg1	0.403065	0.110194	0.671	0.658	1
Ppp2r2c	0.405209	-0.12672	0.634	0.649	1
Bin1	0.406395	0.127895	0.598	0.55	1
Fam102b	0.407313	-0.11363	0.878	0.878	1
Cd44	0.408849	0.128407	0.671	0.63	1
Mtch1	0.409378	-0.11107	0.976	0.975	1
Ddit4	0.411114	0.267413	0.293	0.275	1
Trim47	0.41199	0.134565	0.549	0.486	1
Mfap5	0.413649	-0.11362	0.512	0.543	1
Dhrs7	0.417038	-0.11312	0.805	0.785	1
Megf6	0.420399	-0.17949	0.085	0.112	1
Tmem140	0.421057	-0.10831	0.659	0.645	1
Adamtsl1	0.424447	-0.12415	0.427	0.448	1
Josd2	0.42876	-0.11127	0.427	0.434	1
Fgfr2	0.430175	0.109647	0.561	0.505	1
Cggbp1	0.431069	0.118637	0.744	0.757	1
Vdac3	0.431516	-0.10242	0.768	0.731	1
Apod	0.434062	-1.29883	0.878	0.77	1
Slc6a8	0.436638	-0.13041	0.598	0.63	1
Vapb	0.439282	-0.10576	0.61	0.584	1
Stc1	0.444977	-0.39701	0.22	0.242	1
Lsm10	0.44859	-0.10115	0.256	0.269	1
Cxcl2	0.450766	-0.61455	0.195	0.23	1
Higd2a	0.450945	-0.11745	0.707	0.674	1
Sgk1	0.454138	0.213056	0.939	0.94	1

Pigk	0.45632	-0.1012	0.671	0.654	1
Tmem26	0.461086	-0.11244	0.28	0.31	1
Pde4b	0.461539	-0.10932	0.256	0.284	1
Ehbp1	0.462983	0.129102	0.476	0.469	1
Jun	0.467777	0.133627	1	0.993	1
Ramp3	0.471429	-0.37612	0.402	0.405	1
Efna2	0.471468	0.204168	0.268	0.245	1
Eny2	0.472134	-0.10877	0.756	0.755	1
Cbx1	0.475666	0.10081	0.659	0.616	1
Fst	0.477213	-0.14946	0.146	0.17	1
Mamdc2	0.480386	-0.10332	0.183	0.205	1
Id3	0.491353	0.152032	0.878	0.796	1
Elf2	0.496364	0.128766	0.78	0.732	1
Rtl8a	0.498293	0.116648	0.524	0.501	1
Gpatch8	0.503026	0.127791	0.476	0.459	1
Ints6	0.508281	0.129316	0.744	0.715	1
Pja2	0.514336	-0.11003	0.646	0.626	1
Tmeff2	0.516627	-0.21422	0.341	0.341	1
Ptpre	0.517816	-0.1009	0.402	0.416	1
Ddx24	0.525428	0.135878	0.683	0.624	1
Ccl21a	0.528838	-0.23258	0.683	0.623	1
Cxcl10	0.535984	-0.25542	0.268	0.288	1
Ccl2	0.54096	-0.13618	0.817	0.761	1
Serpine1	0.5473	0.143557	0.817	0.796	1
Prpf38b	0.549115	0.132544	0.744	0.718	1
Tusc3	0.558657	-0.11594	0.573	0.572	1
Map1lc3a	0.559496	-0.11682	0.963	0.953	1
Sspn	0.561472	-0.10141	0.549	0.563	1
Tmem119	0.56185	-0.14087	0.293	0.307	1
Ifi203	0.567026	-0.12245	0.366	0.384	1
Ranbp2	0.567058	0.134732	0.683	0.651	1
Creb5	0.567383	0.105214	0.78	0.875	1
Dhx40	0.568414	-0.10133	0.463	0.451	1
Dut	0.568512	-0.10537	0.378	0.38	1
Postn	0.569714	0.613457	0.427	0.39	1
Cct2	0.570186	0.130438	0.829	0.764	1
Fgl2	0.573406	-0.19379	0.707	0.689	1
Pmepa1	0.574279	-0.29407	0.744	0.687	1
Cp	0.576359	-0.15304	0.378	0.332	1
Stub1	0.576435	-0.10264	0.78	0.75	1
Ttc28	0.579049	0.142045	0.732	0.709	1
Adm	0.58668	-0.22125	0.293	0.291	1
Itga5	0.596352	0.207102	0.561	0.54	1

Qpct	0.598025	-0.11875	0.573	0.571	1
Has1	0.604977	0.116541	0.622	0.595	1
Mme	0.616027	-0.10283	0.146	0.164	1
Igfbp2	0.620042	-0.60571	0.146	0.162	1
Dnajb14	0.620446	-0.10189	0.512	0.467	1
Ramp2	0.629663	-0.12469	0.585	0.54	1
Gadd45g	0.630513	-0.23863	0.841	0.836	1
Dnm3os	0.633428	0.21166	0.512	0.476	1
Mgp	0.635792	-0.60603	0.159	0.131	1
Cpsf6	0.640529	0.115105	0.524	0.525	1
Rbp1	0.644424	-0.15556	0.232	0.24	1
Klf2	0.65695	-0.11102	0.817	0.753	1
Cdkn1c	0.657258	0.176519	0.5	0.496	1
Klk1	0.658666	0.132156	0.659	0.662	1
B830012L1	0.658991	-0.13033	0.512	0.501	1
Ccnd1	0.675967	-0.1148	0.207	0.22	1
Atp5b	0.685656	0.1158	0.951	0.951	1
Acta2	0.699118	-0.15632	0.146	0.163	1
Cxcl1	0.706774	-0.48208	0.5	0.477	1
Fmr1	0.715158	0.149333	0.622	0.592	1
Fabp4	0.722547	0.353505	0.354	0.338	1
Col7a1	0.7297	-0.1117	0.207	0.221	1
Lbp	0.737901	-0.25003	0.5	0.45	1
Rgcc	0.776847	-0.1531	0.488	0.493	1
Ptgs2	0.777809	-0.21812	0.122	0.108	1
Pdia6	0.783066	0.109903	0.866	0.881	1
Lgals3	0.794791	-0.10909	0.976	0.971	1
Spry2	0.798234	-0.1286	0.732	0.667	1
Sfn	0.801235	0.104292	0.293	0.269	1
Csrp2	0.819715	-0.21187	0.476	0.454	1
Cilp	0.836953	-0.4986	0.573	0.53	1
Mt2	0.840441	-0.11355	0.939	0.881	1
Hacd4	0.848447	-0.10583	0.524	0.476	1
Ctla2a	0.850044	-0.17577	0.512	0.517	1
Tpm4	0.855063	-0.11426	0.902	0.879	1
Adh5	0.858991	-0.11361	0.439	0.426	1
C1s1	0.86057	-0.14801	0.793	0.795	1
Btbd3	0.863644	0.104903	0.171	0.167	1
Prpf4b	0.867228	0.243849	0.61	0.646	1
Pxdn	0.8706	0.115723	0.524	0.514	1
Klk1b27	0.873401	-0.15809	0.622	0.611	1
Gch1	0.87776	-0.11961	0.378	0.336	1
Cxcl12	0.896924	-0.15295	0.366	0.346	1

Phlda1	0.933471	-0.16963	0.622	0.582	1
Pla2g16	0.950731	-0.12445	0.195	0.183	1
Fbn2	0.955401	-0.2726	0.195	0.173	1
Akr1b3	0.967196	-0.11487	0.78	0.735	1
Col6a3	0.996428	-0.24364	0.841	0.866	1
Tspan6	0.996747	0.132244	0.207	0.208	1
