# Supplementary material for: Transcriptomic characterization of Lonrf1 at the single-cell level under pathophysiological conditions
Source: J Biochem. 2023 Mar 8;173(6):459–69. doi: 10.1093/jb/mvad021 (PMC10226518; doi:10.1093/jb/mvad021)
Supplement: Web_Material_mvad021 [file web_material_mvad021.zip › Supplementary Table S7.pdf]

Supplementary Table S7

DEG LonFR1+vsLonRF1- in Tomlow Fibro from wound

	p_val	avg_log2F(	pct.1	pct.2	p_val_adj
Lonrf1	0	0.820127	1	0	0
Lepr	2.46E-17	0.471613	0.333	0.144	5.12E-13
Prlr	3.43E-17	1.054182	0.231	0.081	7.14E-13
Dio3	4.82E-17	0.375796	0.122	0.026	1.00E-12
Npm1	6.00E-17	0.491784	0.983	0.964	1.25E-12
Hhip	6.49E-17	0.17015	0.126	0.027	1.35E-12
Pappa2	3.65E-16	0.571645	0.221	0.077	7.58E-12
Atp1b1	3.40E-15	0.136307	0.102	0.02	7.07E-11
Dcn	3.47E-15	-0.40122	1	1	7.21E-11
Gsn	4.64E-15	-0.54151	1	0.999	9.64E-11
Cd24a	1.23E-14	0.729828	0.235	0.093	2.55E-10
Bcl2	2.34E-14	0.536889	0.354	0.177	4.86E-10
Igfbp3	7.82E-14	2.064733	0.442	0.266	1.63E-09
Tfap2c	8.96E-14	0.241183	0.119	0.03	1.86E-09
Plpp3	1.38E-13	-0.39128	0.959	0.986	2.87E-09
Ubash3b	1.85E-13	0.259319	0.231	0.094	3.84E-09
Slc26a7	2.88E-13	0.70705	0.136	0.039	5.99E-09
Eva1a	3.48E-13	0.174168	0.143	0.042	7.23E-09
Hspa8	3.80E-13	0.349611	1	0.998	7.90E-09
Hsp90ab1	2.52E-12	0.329932	1	1	5.23E-08
Lef1	3.22E-12	0.331679	0.136	0.042	6.69E-08
Scara5	4.40E-12	-0.42684	0.728	0.881	9.14E-08
Cfl1	1.44E-11	0.415509	0.993	0.975	3.00E-07
Rarres2	1.47E-11	-0.37449	0.854	0.96	3.05E-07
Fscn1	1.60E-11	0.316141	0.551	0.366	3.32E-07
Cpne5	2.28E-11	0.201341	0.16	0.056	4.74E-07
Wif1	3.80E-11	0.320941	0.252	0.116	7.91E-07
Serping1	4.15E-11	-0.30653	0.946	0.991	8.62E-07
Rnase4	4.70E-11	-0.36851	0.969	0.99	9.77E-07
H3f3b	5.13E-11	0.341286	1	1	1.07E-06
Tnxb	5.50E-11	-0.4217	0.793	0.894	1.14E-06
Dusp2	5.69E-11	0.361316	0.276	0.136	1.18E-06
Mex3a	6.45E-11	0.272252	0.31	0.165	1.34E-06
Tob2	7.45E-11	0.41914	0.891	0.824	1.55E-06
Psap	7.97E-11	-0.24738	0.997	0.995	1.66E-06
Tgif2	8.60E-11	0.175533	0.259	0.123	1.79E-06
Ly6a	9.04E-11	-0.34599	0.905	0.969	1.88E-06
Notch1	9.27E-11	0.294469	0.313	0.165	1.93E-06
Efemp1	9.82E-11	-0.44598	0.718	0.838	2.04E-06
Il1r2	1.05E-10	-0.39025	0.748	0.863	2.19E-06

Gadd45b	1.39E-10	0.613701	0.952	0.902	2.89E-06
Igfbp6	1.46E-10	-0.37336	0.847	0.943	3.04E-06
Tmem176l	1.47E-10	0.271167	0.378	0.216	3.05E-06
Lrp1	1.47E-10	-0.30914	0.969	0.991	3.05E-06
Tuba1c	1.76E-10	0.471052	0.571	0.407	3.65E-06
Bhlhe40	2.52E-10	0.442548	0.793	0.665	5.24E-06
Ablim1	3.15E-10	-0.29994	0.626	0.778	6.55E-06
Gli1	3.48E-10	0.184325	0.228	0.102	7.24E-06
Cebpb	4.48E-10	0.55514	0.993	0.992	9.31E-06
Ncl	4.65E-10	0.397246	0.973	0.936	9.66E-06
Adh7	4.83E-10	-0.39365	0.772	0.858	1.01E-05
Slc38a2	5.59E-10	0.340514	0.993	0.984	1.16E-05
Ctdspl	5.99E-10	0.409409	0.514	0.363	1.24E-05
Clec3b	6.19E-10	-0.43146	0.752	0.878	1.29E-05
Lmo4	6.86E-10	0.453484	0.793	0.685	1.43E-05
Pcolce2	8.86E-10	-0.38877	0.765	0.867	1.84E-05
Tfap2a	1.02E-09	0.192209	0.102	0.03	2.13E-05
Bambi	1.20E-09	0.295072	0.327	0.186	2.48E-05
Sobp	1.22E-09	0.396114	0.391	0.241	2.54E-05
Mmp2	1.26E-09	-0.32382	0.969	0.99	2.61E-05
Rps24	1.31E-09	0.255526	1	0.998	2.72E-05
Runx1	1.36E-09	0.48374	0.616	0.464	2.83E-05
Htra3	1.52E-09	-0.30945	0.864	0.953	3.15E-05
Sulf2	1.83E-09	-0.35589	0.643	0.756	3.80E-05
Sertad2	1.88E-09	0.419199	0.745	0.622	3.92E-05
Fbn1	1.92E-09	-0.3847	0.949	0.973	3.99E-05
Ndnf	1.95E-09	0.486377	0.218	0.104	4.05E-05
Rab20	2.07E-09	0.137818	0.163	0.066	4.30E-05
Mgst1	2.10E-09	-0.28793	0.857	0.934	4.36E-05
Purb	2.16E-09	0.351304	0.867	0.784	4.49E-05
Aebp1	2.20E-09	-0.37335	0.912	0.978	4.58E-05
Tgfbr3	2.23E-09	-0.28165	0.888	0.946	4.63E-05
Rps19	2.32E-09	0.311229	0.997	0.994	4.82E-05
Adamts2	2.58E-09	-0.32343	0.932	0.978	5.36E-05
Adgrl1	4.13E-09	0.180792	0.32	0.183	8.58E-05
Ubc	4.30E-09	0.370653	1	0.997	8.94E-05
Cspg4	4.39E-09	0.232562	0.194	0.087	9.14E-05
Wnt6	5.29E-09	0.147904	0.116	0.04	0.00011
Cd34	5.42E-09	-0.30876	0.891	0.965	0.000113
Ncam1	5.65E-09	0.300797	0.476	0.318	0.000118
Ctsh	6.54E-09	-0.32649	0.813	0.899	0.000136
Sqle	6.95E-09	0.184333	0.289	0.157	0.000144
H2afz	7.23E-09	0.429344	0.98	0.96	0.00015

Rps2	7.26E-09	0.257168	1	0.999	0.000151
Ube2s	7.34E-09	0.388372	0.946	0.912	0.000153
Timp2	7.43E-09	-0.26568	0.983	0.997	0.000155
Hsp90aa1	7.84E-09	0.357856	0.99	0.986	0.000163
Myo1b	8.62E-09	0.22808	0.398	0.254	0.000179
Hspa2	8.92E-09	0.314624	0.51	0.36	0.000185
Dnajb1	1.05E-08	0.332529	0.847	0.73	0.000218
Thbs2	1.25E-08	-0.3768	0.895	0.957	0.000261
Cdh4	1.26E-08	-0.34555	0.52	0.673	0.000261
Eif2s2	1.26E-08	0.269896	0.949	0.894	0.000262
Hnrnpu	1.38E-08	0.330223	0.969	0.941	0.000286
Nckap5l	1.40E-08	0.208013	0.459	0.303	0.000291
Eif4a1	1.42E-08	0.39444	0.993	0.985	0.000296
Rps16	1.45E-08	0.268841	0.997	0.997	0.000301
Col3a1	1.46E-08	-0.32451	1	1	0.000304
Junb	1.66E-08	0.573183	0.993	0.989	0.000346
Chchd2	1.72E-08	0.262533	0.993	0.992	0.000357
Grn	1.79E-08	-0.22123	0.847	0.942	0.000372
Ccnl1	1.83E-08	0.283688	0.993	0.971	0.00038
Plod2	1.84E-08	0.125225	0.184	0.082	0.000382
Ptma	1.93E-08	0.391492	1	0.999	0.000402
Calm1	1.94E-08	0.326461	0.986	0.965	0.000403
Ppp2r2c	1.94E-08	-0.24805	0.378	0.544	0.000404
Hspb1	1.99E-08	0.680446	0.503	0.371	0.000413
Chst8	2.27E-08	0.105515	0.112	0.039	0.000472
Naca	2.31E-08	0.213294	0.997	0.979	0.000479
Sept11	2.35E-08	0.317797	0.653	0.507	0.000489
Runx3	2.37E-08	0.280399	0.272	0.149	0.000492
Ctsk	2.50E-08	-0.29587	0.99	0.998	0.00052
Gns	2.79E-08	-0.24114	0.837	0.915	0.00058
Cdkn1a	3.23E-08	0.503323	0.932	0.894	0.000672
Rps9	3.37E-08	0.195013	1	0.999	0.0007
Kdm6b	3.49E-08	0.266229	0.983	0.931	0.000726
Rpl6	3.56E-08	0.195231	1	0.996	0.00074
Gem	3.61E-08	0.424228	0.925	0.864	0.00075
Cyp2f2	3.61E-08	-0.5411	0.578	0.721	0.000751
Pla2g2e	3.84E-08	-0.33546	0.412	0.561	0.000798
Taf1d	3.97E-08	0.363136	0.759	0.668	0.000826
Top1	4.10E-08	0.350322	0.949	0.943	0.000852
Marcksl1	4.12E-08	0.302683	0.701	0.545	0.000857
Sod3	4.17E-08	-0.37384	0.558	0.701	0.000867
Col1a1	4.50E-08	-0.31135	1	1	0.000936
Gjc1	4.77E-08	0.15229	0.276	0.154	0.000993

Nav2	4.83E-08	0.192491	0.238	0.125	0.001005
Tenm3	4.88E-08	0.158368	0.248	0.132	0.001014
Tgfbi	5.02E-08	-0.39102	0.752	0.879	0.001044
Pam	5.44E-08	-0.30851	0.827	0.89	0.001131
Igfbp4	6.02E-08	0.670955	0.466	0.322	0.001252
Inhba	6.03E-08	0.639057	0.306	0.184	0.001254
Lmna	6.20E-08	0.294043	1	0.996	0.001289
Hnrnpl	6.24E-08	0.365101	0.881	0.816	0.001298
Tnfrsf12a	6.35E-08	0.416762	0.844	0.773	0.001319
Vim	6.50E-08	0.285725	1	1	0.001351
Id4	6.56E-08	0.153456	0.361	0.218	0.001365
Mdk	6.98E-08	0.377465	0.473	0.331	0.001451
Prrc2c	6.99E-08	0.256831	0.922	0.873	0.001453
H3f3a	8.27E-08	0.225656	1	0.999	0.001718
Nasp	8.51E-08	0.394432	0.752	0.638	0.001769
a	8.86E-08	0.538128	0.367	0.237	0.001841
Actg1	9.33E-08	0.388166	1	0.997	0.00194
Rps8	9.44E-08	0.192547	1	0.999	0.001962
Ier5	9.45E-08	0.43586	0.942	0.909	0.001965
S100a13	9.59E-08	-0.2172	0.925	0.956	0.001995
Tiparp	9.99E-08	0.245144	0.803	0.678	0.002076
Dnaja1	1.04E-07	0.39583	0.983	0.959	0.002157
Gpx1	1.12E-07	0.342938	0.973	0.955	0.002336
Stmn1	1.23E-07	0.294368	0.361	0.229	0.002565
Srrt	1.26E-07	0.227768	0.578	0.433	0.002629
Dnajb9	1.28E-07	0.357557	0.918	0.831	0.002655
Eif1	1.31E-07	0.234388	1	0.999	0.002721
Nr4a1	1.37E-07	0.456314	0.956	0.904	0.002843
Rtn4	1.43E-07	0.305917	0.99	0.971	0.002981
Ubb	1.45E-07	0.314889	1	1	0.00302
Lrrc15	1.45E-07	0.418764	0.272	0.152	0.003025
Celf2	1.46E-07	-0.24242	0.881	0.936	0.003031
Nfkbia	1.55E-07	0.278656	0.993	0.985	0.003215
Shisa5	1.57E-07	-0.19688	0.748	0.835	0.003261
Ctsb	1.58E-07	-0.19679	0.969	0.993	0.003283
Cd47	1.60E-07	-0.22315	0.983	0.99	0.003319
Cks2	1.67E-07	0.291887	0.439	0.301	0.003481
Slc6a6	1.68E-07	0.632562	0.548	0.419	0.003491
Lamb2	1.73E-07	-0.23996	0.878	0.933	0.003602
Creb5	1.73E-07	-0.37056	0.646	0.806	0.003606
Tsc22d2	1.75E-07	0.211072	0.847	0.712	0.003632
Eif5a	1.83E-07	0.255688	0.976	0.957	0.003801
Wsb1	1.83E-07	0.273186	0.983	0.951	0.003814

Hnrnpab	1.87E-07	0.259855	0.939	0.894	0.003878
Pabpc1	1.96E-07	0.225163	0.993	0.979	0.004065
Cyp26b1	2.13E-07	-0.58088	0.789	0.838	0.004432
Tns3	2.27E-07	0.287533	0.35	0.229	0.004727
Egr3	2.38E-07	0.287763	0.401	0.263	0.004955
Klf10	2.41E-07	0.411404	0.565	0.434	0.005017
Rhoj	2.44E-07	-0.241	0.799	0.92	0.005067
Dapk1	2.48E-07	-0.25112	0.677	0.802	0.005156
Btf3	2.48E-07	0.236067	0.997	0.981	0.005159
Cbx4	2.67E-07	0.265566	0.48	0.343	0.005543
Htra1	2.67E-07	-0.2085	0.956	0.983	0.005549
Ifrd1	2.74E-07	0.242975	0.98	0.933	0.005687
F2r	2.89E-07	0.263778	0.493	0.342	0.006005
Sparc	2.89E-07	-0.28566	1	1	0.006009
Ddx5	2.91E-07	0.326192	0.997	0.999	0.006056
Mettl7a1	3.00E-07	-0.31218	0.626	0.746	0.006235
Ezr	3.23E-07	0.147311	0.228	0.121	0.006715
Rbbp6	3.46E-07	0.358487	0.908	0.872	0.0072
Snrnp70	3.52E-07	0.260882	0.915	0.869	0.007319
Rpl24	3.59E-07	0.181026	0.993	0.994	0.007469
Itpr1	3.67E-07	0.110127	0.265	0.15	0.007625
Ms4a4d	4.26E-07	-0.21608	0.503	0.644	0.008857
Col1a2	4.38E-07	-0.26966	1	1	0.009115
Klf13	4.52E-07	0.388559	0.864	0.75	0.009404
Rps3	4.58E-07	0.220181	0.997	0.996	0.009513
Sfpq	4.94E-07	0.290017	0.969	0.948	0.010263
Lgals9	4.94E-07	-0.22192	0.65	0.75	0.010263
Ptger3	4.98E-07	0.100948	0.143	0.062	0.010355
Adh1	5.09E-07	-0.28557	0.609	0.786	0.010587
Ppp1r14a	5.36E-07	-0.33631	0.466	0.595	0.011151
Gas1	5.37E-07	-0.31593	0.922	0.954	0.011166
Ltbp1	5.37E-07	-0.30231	0.833	0.899	0.011167
Wwtr1	5.57E-07	0.222069	0.901	0.854	0.011577
Zfp131	5.84E-07	0.355992	0.738	0.641	0.012149
Map3k8	6.02E-07	0.160199	0.548	0.401	0.012521
Medag	6.13E-07	-0.22634	0.65	0.824	0.012738
Smarca5	6.21E-07	0.233865	0.871	0.781	0.012907
Tiam2	6.40E-07	0.12923	0.187	0.094	0.013306
Maff	6.40E-07	0.295759	0.728	0.601	0.013309
Stat3	6.40E-07	0.311205	0.956	0.888	0.013314
Pde3a	6.44E-07	0.162011	0.49	0.34	0.013391
Egflam	6.52E-07	0.196912	0.327	0.202	0.013553
Hspa1b	6.66E-07	0.43147	0.915	0.809	0.01385

Hnrnpa1	7.09E-07	0.288942	0.949	0.908	0.014735
Ezh2	7.20E-07	0.226011	0.371	0.249	0.01496
Nr4a3	7.35E-07	0.283161	0.524	0.385	0.015287
Rps6kb1	7.61E-07	0.217316	0.772	0.648	0.01582
Gas6	7.65E-07	-0.35059	0.857	0.926	0.015899
Btg1	7.71E-07	0.227135	0.993	0.983	0.016035
Zbtb2	8.19E-07	0.186423	0.483	0.349	0.017025
St6galnac5	8.36E-07	0.158518	0.112	0.046	0.017386
Auts2	8.53E-07	0.304458	0.412	0.29	0.017733
Col5a3	8.70E-07	-0.30203	0.748	0.866	0.018094
Isyna1	9.06E-07	0.15407	0.262	0.153	0.018826
Abi3bp	9.09E-07	-0.36627	0.816	0.887	0.018905
Hnrnpc	1.01E-06	0.224403	0.884	0.838	0.020999
Gpc3	1.02E-06	0.148851	0.412	0.273	0.021107
Alyref	1.07E-06	0.266983	0.718	0.608	0.022346
Tmem176a	1.08E-06	0.137522	0.235	0.134	0.022501
Oaz1	1.11E-06	0.242191	0.993	0.994	0.023038
Col5a1	1.12E-06	-0.25364	0.922	0.979	0.023244
Cux1	1.14E-06	0.126813	0.35	0.222	0.023608
Csrnp1	1.14E-06	0.274926	0.884	0.815	0.02378
Csf1	1.26E-06	-0.28093	0.697	0.801	0.026174
Rpl36a	1.31E-06	0.223644	0.99	0.983	0.027162
Tuba4a	1.31E-06	0.159556	0.293	0.178	0.027253
Timp3	1.36E-06	-0.38175	0.81	0.879	0.028214
Ier3	1.36E-06	0.378405	0.969	0.963	0.02831
Twsg1	1.40E-06	-0.22809	0.765	0.84	0.02911
Rab7	1.40E-06	-0.19707	0.864	0.907	0.029133
Fau	1.45E-06	0.145804	1	1	0.030222
Srsf6	1.50E-06	0.232709	0.905	0.81	0.031279
Smad7	1.54E-06	0.31309	0.837	0.753	0.031932
Tshz3	1.59E-06	0.136923	0.483	0.333	0.033067
Epha4	1.64E-06	0.169655	0.231	0.13	0.034182
Aff3	1.66E-06	-0.22481	0.599	0.706	0.034439
Rsf1	1.68E-06	0.245174	0.697	0.582	0.034856
Rpl15	1.71E-06	0.221395	1	0.996	0.03546
Ifitm1	1.86E-06	0.398232	0.388	0.27	0.038743
Vcl	1.88E-06	0.212057	0.531	0.409	0.039115
Pnrc1	1.88E-06	0.21615	0.98	0.973	0.039174
Lum	1.91E-06	-0.29133	0.946	0.98	0.039654
Rpl27a	1.98E-06	0.154561	1	1	0.041194
Itgbl1	1.99E-06	-0.20695	0.741	0.892	0.041292
Rcc2	2.09E-06	0.191212	0.51	0.393	0.043422
Kctd1	2.14E-06	0.103793	0.122	0.053	0.044522

Nbl1	2.18E-06	-0.30681	0.915	0.968	0.045365
Idi1	2.20E-06	0.201106	0.34	0.221	0.045655
Cxcr4	2.24E-06	0.149912	0.16	0.078	0.046473
Crabp2	2.26E-06	0.28793	0.235	0.135	0.047084
Eloc	2.37E-06	0.220089	0.861	0.784	0.049193
Rpl18	2.37E-06	0.184264	1	0.997	0.04924
Raly	2.40E-06	0.205804	0.871	0.816	0.049928
Neat1	2.47E-06	0.327065	0.983	0.952	0.051297
Ywhaz	2.49E-06	0.211122	0.98	0.937	0.05186
Ecm1	2.54E-06	-0.29127	0.786	0.88	0.052795
Gda	2.55E-06	-0.24871	0.582	0.702	0.052928
Rlim	2.59E-06	0.185341	0.585	0.436	0.053768
Sf3b1	2.68E-06	0.258022	0.956	0.923	0.055702
Stk17b	2.72E-06	0.121806	0.337	0.214	0.056466
Arl4a	2.76E-06	0.31081	0.793	0.683	0.057416
Slc2a1	2.77E-06	0.175288	0.333	0.216	0.057617
Ywhae	2.83E-06	0.158922	0.969	0.953	0.058792
Ly6c1	2.90E-06	-0.2814	0.857	0.936	0.060258
Rpl8	3.08E-06	0.189416	1	0.999	0.063997
Rhbdf2	3.09E-06	0.115554	0.241	0.14	0.064261
Pcf11	3.12E-06	0.245801	0.823	0.734	0.064771
Zfand5	3.13E-06	0.528536	0.976	0.969	0.065148
Ppia	3.17E-06	0.275561	1	0.993	0.065986
Rpl9	3.20E-06	0.191719	1	0.997	0.066541
Nsd3	3.23E-06	0.235062	0.922	0.88	0.067201
Icam1	3.28E-06	0.404597	0.922	0.875	0.068181
Tmem140	3.30E-06	-0.21052	0.507	0.623	0.068582
Taf4b	3.31E-06	0.212692	0.303	0.198	0.068898
Midn	3.38E-06	0.361344	0.915	0.858	0.070231
Trp53	3.49E-06	0.308523	0.793	0.683	0.072479
Pim1	3.59E-06	0.351143	0.918	0.889	0.074686
Serpine2	3.66E-06	0.82242	0.391	0.287	0.076063
Ang	3.74E-06	-0.21968	0.633	0.737	0.077709
Ctsl	3.82E-06	-0.18748	0.993	0.997	0.079451
Gng12	3.86E-06	-0.2276	0.83	0.907	0.080157
Pim3	3.99E-06	0.311893	0.714	0.613	0.082946
Add3	4.02E-06	-0.19124	0.823	0.886	0.083511
Thoc6	4.03E-06	0.187477	0.599	0.47	0.083782
Ankrd10	4.09E-06	0.28894	0.544	0.441	0.085086
Gnl3	4.15E-06	0.186547	0.68	0.572	0.086265
Tpt1	4.23E-06	0.187374	1	1	0.08785
Dynll1	4.32E-06	0.22375	0.993	0.984	0.089881
Nr4a2	4.35E-06	0.382372	0.561	0.457	0.090458

Oxtr	4.40E-06	-0.31774	0.388	0.521	0.091503
Comt	4.42E-06	-0.19066	0.786	0.845	0.091911
Rpl13a	4.42E-06	0.221864	0.976	0.939	0.091958
Gpnmb	4.68E-06	-0.29999	0.718	0.811	0.097282
Phf3	4.72E-06	0.255806	0.714	0.631	0.098034
Eif3j1	4.75E-06	0.205703	0.776	0.644	0.098764
Hnrnpa2b1	4.76E-06	0.301349	0.986	0.969	0.098873
Svep1	4.76E-06	-0.25827	0.796	0.854	0.098886
Col5a2	4.82E-06	-0.23484	0.966	0.992	0.100137
Epop	4.84E-06	0.119671	0.112	0.049	0.100545
Col13a1	4.85E-06	0.219532	0.156	0.079	0.100794
Apoe	4.90E-06	0.459832	0.983	0.955	0.101894
Rps28	4.93E-06	0.168712	0.997	0.997	0.102425
Gm26615	5.11E-06	0.275756	0.442	0.329	0.106164
Fnbp1l	5.19E-06	0.145933	0.269	0.165	0.107877
Fam13a	5.23E-06	0.162589	0.122	0.056	0.108642
Tsc22d1	5.45E-06	0.349746	0.81	0.727	0.113326
Rorb	5.48E-06	0.165649	0.31	0.195	0.11385
Adamts9	5.49E-06	0.189547	0.238	0.139	0.114046
Vegfa	5.53E-06	0.238742	0.776	0.66	0.115001
Srgn	5.69E-06	0.182907	0.224	0.131	0.118287
Il1rl2	5.74E-06	-0.18537	0.585	0.676	0.119422
Tob1	5.75E-06	0.349014	0.888	0.838	0.119441
Sorbs1	5.84E-06	0.192883	0.241	0.149	0.12151
Npc2	5.86E-06	-0.18742	0.966	0.987	0.121791
Sltm	5.96E-06	0.238966	0.69	0.576	0.123814
Grasp	6.13E-06	0.297358	0.711	0.613	0.127495
Pmepa1	6.45E-06	0.320855	0.827	0.758	0.134194
Ahdc1	6.55E-06	0.273994	0.687	0.57	0.136155
Set	6.58E-06	0.304177	0.908	0.843	0.136888
Nop58	6.59E-06	0.280274	0.697	0.585	0.137101
Rpl18a	6.62E-06	0.192397	1	0.996	0.137721
Phf13	6.92E-06	0.181374	0.527	0.411	0.143844
Gstm1	6.99E-06	-0.2033	0.776	0.836	0.145396
Yrdc	7.21E-06	0.192023	0.507	0.387	0.149849
Cdc42ep4	7.24E-06	0.227714	0.323	0.212	0.150492
Rps26	7.40E-06	0.190169	1	0.997	0.15394
Fbxl7	7.53E-06	0.16735	0.32	0.208	0.156446
Prdx1	7.54E-06	0.221127	0.99	0.985	0.156737
Ppp1r15a	7.60E-06	0.279891	0.976	0.944	0.157917
Chd3os	7.97E-06	0.10477	0.177	0.094	0.165643
Clp1	8.30E-06	0.122809	0.282	0.173	0.172595
B230369F2	8.36E-06	0.197603	0.33	0.22	0.173721

Tnn	8.40E-06	0.273986	0.119	0.053	0.174734
Lpar1	8.45E-06	-0.19335	0.789	0.865	0.175681
Ddx3x	8.62E-06	0.188648	0.98	0.969	0.179178
Man1a	8.65E-06	-0.23131	0.878	0.93	0.179784
Kansl1l	8.81E-06	0.201977	0.616	0.499	0.183219
Col27a1	9.07E-06	0.127911	0.238	0.141	0.18859
Scarb1	9.15E-06	0.160359	0.248	0.148	0.190137
Fstl1	9.20E-06	-0.24215	0.973	0.996	0.191168
Wdr26	9.28E-06	0.188294	0.898	0.841	0.192904
Ppm1d	9.31E-06	0.189246	0.354	0.24	0.193588
Sparcl1	9.38E-06	0.31442	0.503	0.376	0.19493
Ptgir	9.49E-06	0.14178	0.15	0.075	0.197342
Rbm39	9.53E-06	0.222691	0.986	0.979	0.198213
Eif4a2	9.54E-06	0.258515	0.956	0.91	0.198325
Ghr	9.72E-06	-0.1993	0.735	0.832	0.202098
Cmtm3	9.94E-06	-0.19536	0.765	0.869	0.206688
Rbm4b	9.98E-06	0.320782	0.544	0.427	0.207529
Rspo3	9.98E-06	0.133315	0.163	0.085	0.20753
Calm2	9.98E-06	0.249058	0.993	0.987	0.207565
Zfp36	1.01E-05	0.270415	0.99	0.966	0.210762
Crispld2	1.04E-05	-0.35584	0.881	0.924	0.216081
Rel	1.10E-05	0.214961	0.565	0.447	0.228527
Steap3	1.16E-05	-0.20754	0.612	0.739	0.241679
Sipa1l1	1.16E-05	0.140723	0.289	0.187	0.241853
Myo10	1.17E-05	0.192798	0.378	0.262	0.243624
Prdm1	1.26E-05	0.131036	0.156	0.08	0.261375
Dkk2	1.30E-05	0.36229	0.442	0.324	0.269735
Prdx6	1.31E-05	-0.21949	0.881	0.937	0.271681
Sema3b	1.35E-05	-0.15816	0.527	0.657	0.280659
Rasl11a	1.36E-05	0.110983	0.252	0.149	0.28172
Hexim1	1.36E-05	0.362028	0.81	0.764	0.282096
Cd63	1.49E-05	-0.15649	0.997	0.999	0.309176
Rpl27	1.49E-05	0.174551	0.983	0.975	0.309247
Irs2	1.50E-05	0.311073	0.752	0.694	0.310926
Sfrp2	1.51E-05	0.116819	0.139	0.069	0.314807
Hexb	1.57E-05	-0.14758	0.636	0.718	0.327381
Adgrd1	1.58E-05	-0.20787	0.592	0.712	0.328925
Actn1	1.59E-05	0.158311	0.31	0.199	0.331245
Podn	1.62E-05	-0.18924	0.619	0.717	0.336058
Map2k3	1.62E-05	0.220109	0.735	0.636	0.336336
Hnrnpk	1.63E-05	0.204124	0.997	0.971	0.33816
Sbno1	1.69E-05	0.262593	0.789	0.758	0.351369
Sde2	1.71E-05	0.225513	0.738	0.653	0.356052

Coch	1.78E-05	0.19029	0.439	0.318	0.370439
Trps1	1.82E-05	0.43946	0.745	0.681	0.379015
Hspa1a	1.83E-05	0.454537	0.932	0.872	0.379831
Lamp1	1.83E-05	-0.17262	0.952	0.986	0.380661
Zbtb7a	1.86E-05	0.317899	0.884	0.849	0.387323
Eif3h	1.89E-05	0.193527	0.939	0.911	0.393186
Tln2	1.92E-05	0.210508	0.282	0.189	0.398964
Hspa4	1.96E-05	0.175129	0.677	0.554	0.408076
Gdf10	1.98E-05	0.171845	0.112	0.05	0.412082
Phf23	2.00E-05	0.231877	0.677	0.562	0.415388
Jmjd1c	2.00E-05	0.208925	0.925	0.864	0.415567
Aqp1	2.01E-05	0.261093	0.262	0.167	0.416972
Csde1	2.06E-05	0.150887	0.956	0.938	0.428761
Cnn3	2.08E-05	0.185526	0.84	0.763	0.432291
Xdh	2.09E-05	-0.18388	0.667	0.782	0.433537
Sub1	2.10E-05	0.24085	0.898	0.839	0.435855
Elf1	2.14E-05	0.258722	0.857	0.784	0.444724
Gorasp2	2.21E-05	-0.15605	0.667	0.72	0.458923
Angptl2	2.26E-05	-0.26857	0.687	0.777	0.470171
Mcl1	2.28E-05	0.223307	0.986	0.976	0.474197
Flt3l	2.39E-05	-0.20529	0.395	0.513	0.496654
Cox6b2	2.40E-05	0.202617	0.221	0.136	0.4981
Loxl2	2.40E-05	-0.15179	0.799	0.908	0.499898
Ptges3	2.47E-05	0.203141	0.85	0.801	0.512955
Rexo1	2.52E-05	0.228611	0.497	0.383	0.523673
Slc3a2	2.56E-05	0.289698	0.84	0.799	0.532425
Rpl3	2.60E-05	0.201418	0.997	0.991	0.540394
Gja1	2.61E-05	-0.26173	0.901	0.926	0.542535
Ssbp3	2.61E-05	0.212869	0.755	0.687	0.542603
H2afy	2.83E-05	0.17129	0.687	0.61	0.589184
Eif1a	2.86E-05	0.25399	0.755	0.663	0.594246
Cpq	2.86E-05	-0.14743	0.793	0.876	0.5946
Aldh1a1	2.93E-05	-0.20647	0.575	0.693	0.609414
Tra2b	2.93E-05	0.234641	0.949	0.92	0.610017
Slbp	2.96E-05	0.227878	0.687	0.585	0.615148
Ppic	3.05E-05	-0.18407	0.942	0.981	0.634889
Slc35f5	3.06E-05	-0.14276	0.619	0.713	0.637191
Uqcrh	3.12E-05	0.165436	0.966	0.968	0.648183
Hspa5	3.13E-05	0.19242	1	0.996	0.651357
Ier2	3.15E-05	0.339493	0.973	0.956	0.653887
Arid4b	3.16E-05	0.206671	0.908	0.839	0.656215
Mrps6	3.16E-05	0.241224	0.48	0.376	0.656874
Rps13	3.38E-05	0.188239	0.997	0.995	0.70176

Hbegf	3.39E-05	0.197727	0.364	0.252	0.704171
Sox11	3.46E-05	0.248198	0.19	0.112	0.720361
Ankrd11	3.49E-05	0.226204	0.881	0.828	0.725983
Cdkn1c	3.52E-05	0.236869	0.622	0.52	0.730994
Fosl1	3.57E-05	0.227983	0.422	0.314	0.741987
Prnp	3.66E-05	-0.19418	0.922	0.962	0.760216
Anp32b	3.68E-05	0.173747	0.816	0.758	0.766048
Ly6e	3.72E-05	-0.15789	0.799	0.889	0.77357
Pkm	3.87E-05	0.223028	0.922	0.873	0.804929
Dek	3.96E-05	0.307537	0.786	0.714	0.824024
Hnrnpf	3.98E-05	0.197394	0.925	0.907	0.828136
Khdrbs1	4.23E-05	0.186978	0.769	0.699	0.878889
Pard6g	4.23E-05	0.113558	0.248	0.155	0.880323
Serpinh1	4.36E-05	-0.16531	0.993	0.995	0.907291
Bzw1	4.46E-05	0.164967	0.901	0.842	0.928132
Zcchc24	4.47E-05	-0.19891	0.779	0.856	0.929636
Nupr1	4.48E-05	-0.1981	0.949	0.985	0.931362
Plod1	4.50E-05	-0.18669	0.796	0.875	0.935014
Egr2	4.55E-05	0.219266	0.374	0.267	0.945901
Frat2	4.60E-05	0.230963	0.299	0.206	0.956227
Ahnak2	4.60E-05	-0.18528	0.616	0.726	0.957229
Cdv3	4.65E-05	0.175711	0.85	0.783	0.967152
Dnm3os	4.69E-05	0.273086	0.612	0.485	0.975618
Slc38a10	4.70E-05	-0.17108	0.748	0.828	0.976114
Prex1	4.86E-05	-0.25169	0.514	0.61	1
Rai14	4.88E-05	0.129869	0.289	0.189	1
Zfp703	4.92E-05	0.169223	0.626	0.506	1
Ndufa4l2	4.98E-05	-0.32483	0.714	0.78	1
Slc24a5	5.24E-05	0.128172	0.15	0.081	1
Fgfr1	5.27E-05	0.371891	0.864	0.824	1
Ttc19	5.32E-05	0.142626	0.537	0.415	1
Mpped2	5.33E-05	0.155078	0.136	0.072	1
Tra2a	5.35E-05	0.301076	0.837	0.74	1
Mmp23	5.36E-05	-0.17755	0.724	0.844	1
Wnk1	5.40E-05	0.237094	0.939	0.928	1
Klf16	5.65E-05	0.163774	0.371	0.272	1
Mrpl23	5.71E-05	-0.13038	0.697	0.765	1
Rpain	5.77E-05	0.105345	0.395	0.273	1
Nfia	5.77E-05	-0.1794	0.912	0.959	1
Maf	5.79E-05	-0.22927	0.765	0.842	1
Csnk1a1	5.90E-05	0.209528	0.969	0.96	1
Flvcr1	5.94E-05	0.158521	0.269	0.18	1
Mmp27	5.97E-05	-0.18079	0.52	0.647	1

Skil	6.01E-05	0.301005	0.67	0.609	1
Crybg3	6.08E-05	0.190789	0.439	0.33	1
Rpl35a	6.10E-05	0.155977	1	0.997	1
Impdh2	6.48E-05	0.209547	0.762	0.678	1
Wnt11	6.49E-05	0.161957	0.282	0.186	1
Ddr2	6.53E-05	-0.18309	0.85	0.907	1
Rpl23	6.54E-05	0.141167	1	1	1
Tmed3	6.57E-05	-0.16577	0.857	0.936	1
Smoc2	6.66E-05	-0.36108	0.83	0.872	1
Tial1	6.66E-05	0.145756	0.663	0.541	1
Slc5a3	6.68E-05	0.448141	0.524	0.427	1
Hnrnph1	6.82E-05	0.233071	0.993	0.967	1
Snrpf	6.91E-05	0.204319	0.85	0.802	1
Unc5b	6.98E-05	0.169345	0.238	0.154	1
Arhgef17	7.15E-05	0.123493	0.316	0.222	1
Rplp0	7.19E-05	0.200992	0.997	0.997	1
Ntrk3	7.19E-05	0.105419	0.133	0.068	1
Marveld1	7.25E-05	-0.18117	0.769	0.815	1
Itm2b	7.32E-05	-0.13555	0.997	1	1
Rpl13	7.35E-05	0.172461	1	1	1
Naa50	7.52E-05	0.142384	0.707	0.606	1
Bicc1	7.53E-05	-0.20355	0.786	0.857	1
Ddx21	7.56E-05	0.192424	0.646	0.537	1
Fcgrt	7.67E-05	-0.14193	0.946	0.972	1
Cd200	8.10E-05	0.248169	0.221	0.14	1
Pcolce	8.12E-05	-0.16731	0.929	0.977	1
Rc3h1	8.13E-05	0.19418	0.823	0.75	1
Atf4	8.21E-05	0.235924	0.98	0.973	1
Tmem251	8.24E-05	0.106265	0.306	0.204	1
Ube2k	8.25E-05	0.16959	0.847	0.774	1
Rsl1d1	8.39E-05	0.203685	0.592	0.514	1
Klf11	8.47E-05	0.145911	0.296	0.202	1
Klf4	8.68E-05	0.18961	0.997	0.994	1
Rasd1	8.72E-05	0.48447	0.412	0.319	1
Rps15a	8.94E-05	0.158582	0.993	0.996	1
Rnf19b	9.00E-05	0.159123	0.694	0.598	1
Kmt2a	9.16E-05	0.17105	0.779	0.711	1
Etf1	9.16E-05	0.216758	0.867	0.804	1
Rpl14	9.23E-05	0.152018	0.983	0.985	1
Map3k6	9.24E-05	0.166127	0.306	0.214	1
Cavin3	9.54E-05	-0.17364	0.922	0.947	1
Rbm38	9.59E-05	0.10575	0.19	0.114	1
Topors	9.70E-05	0.265776	0.663	0.574	1

Larp4	0.000101	0.141602	0.721	0.636	1
Rps14	0.000102	0.144167	0.997	0.999	1
Hnrnpll	0.000104	0.131388	0.667	0.557	1
Rpl19	0.000104	0.140391	1	1	1
Lox	0.000104	-0.25097	0.762	0.856	1
Jund	0.000104	0.186655	1	1	1
Furin	0.000107	0.171799	0.745	0.624	1
Ifngr1	0.000108	-0.14707	0.701	0.784	1
Rpl32	0.000108	0.163436	0.997	0.993	1
Baiap2	0.000109	0.196495	0.728	0.629	1
Mef2c	0.00011	0.220897	0.667	0.569	1
Pla2g5	0.000111	-0.21683	0.361	0.457	1
Fkbp9	0.000111	-0.1594	0.891	0.958	1
Pdgfra	0.000112	-0.21522	0.922	0.962	1
Rps21	0.000115	0.147141	1	0.999	1
Fbl	0.000116	0.19944	0.646	0.561	1
Col11a1	0.000119	0.265823	0.204	0.123	1
E330013P(	0.000119	-0.12469	0.136	0.23	1
Arid4a	0.00012	0.175705	0.629	0.51	1
Ptp4a1	0.00012	0.229925	0.908	0.882	1
Mex3d	0.000121	0.119122	0.534	0.413	1
Pmp22	0.000122	-0.13666	0.942	0.974	1
Acin1	0.000124	0.236374	0.81	0.743	1
Rbm8a	0.000124	0.193321	0.786	0.723	1
Lgals7	0.000124	0.16423	0.415	0.305	1
Tox4	0.000127	0.160445	0.534	0.432	1
Tagln2	0.000128	0.264399	0.861	0.812	1
Rpl41	0.000128	0.158167	1	1	1
Nagk	0.00013	-0.148	0.473	0.551	1
Ikzf4	0.000132	0.118834	0.16	0.09	1
Epas1	0.000132	0.107911	0.245	0.16	1
Irx5	0.000132	-0.13408	0.408	0.51	1
Arf6	0.000133	0.194667	0.759	0.694	1
Prkce	0.000134	-0.23291	0.626	0.711	1
Cdk9	0.000139	0.142094	0.578	0.485	1
Foxd1	0.000143	0.143013	0.146	0.08	1
Ptch1	0.000144	0.179869	0.241	0.158	1
Ube2d3	0.000145	0.155282	0.983	0.986	1
Borcs6	0.000148	0.148054	0.371	0.274	1
Eef1a1	0.000148	0.141119	1	1	1
Adk	0.00015	-0.15405	0.714	0.83	1
Snu13	0.00015	0.20161	0.85	0.805	1
Styx	0.000151	0.110646	0.337	0.239	1

Ccnd3	0.000152	0.356213	0.544	0.458	1
Pamr1	0.000158	-0.27224	0.296	0.388	1
Fnbp4	0.00016	0.125729	0.459	0.353	1
Rps27	0.000163	0.152309	1	0.996	1
Nufip2	0.000163	0.198838	0.918	0.87	1
Rsrc2	0.000165	0.184113	0.776	0.714	1
Ntn1	0.000166	-0.11216	0.687	0.777	1
Chka	0.000167	0.316002	0.738	0.69	1
C1ra	0.000168	-0.17611	0.776	0.867	1
Sox9	0.000168	0.189145	0.16	0.092	1
Arih1	0.000168	0.1542	0.874	0.8	1
Rpl39	0.000169	0.178366	1	0.999	1
Snrnp48	0.000171	0.141992	0.432	0.327	1
Gpr1	0.000175	-0.10895	0.156	0.258	1
Rara	0.000176	0.133312	0.456	0.341	1
Tgif1	0.000177	0.204883	0.714	0.635	1
Rgl1	0.000181	-0.11686	0.534	0.625	1
Ube2d2a	0.000182	0.17758	0.874	0.83	1
Mrgprf	0.000183	-0.13507	0.276	0.371	1
Trp53inp2	0.000187	-0.13406	0.429	0.522	1
1810058I2	0.000188	-0.18032	0.861	0.915	1
Azin1	0.00019	0.177928	0.85	0.763	1
Mbnl2	0.000192	0.156264	0.946	0.92	1
Calu	0.000195	-0.14834	0.935	0.981	1
Sh3d19	0.000196	-0.14894	0.677	0.747	1
Hnrnpm	0.000198	0.15234	0.827	0.779	1
Id3	0.000199	0.416216	0.881	0.844	1
Srsf11	0.000201	0.13041	0.908	0.863	1
Eif3e	0.000204	0.146293	0.925	0.884	1
Dnajc21	0.000206	0.138969	0.626	0.515	1
Tcf3	0.000208	0.133583	0.558	0.461	1
Fyn	0.000208	-0.1672	0.762	0.809	1
Slco2b1	0.000209	-0.18829	0.5	0.589	1
Rps5	0.000209	0.18289	0.997	0.994	1
Tpp2	0.000212	0.151912	0.677	0.588	1
Cnksr3	0.000214	0.105946	0.245	0.159	1
Slco3a1	0.000216	-0.10772	0.435	0.533	1
Jak2	0.000218	0.116867	0.483	0.381	1
Peg3	0.000219	0.305272	0.493	0.394	1
Capg	0.000222	-0.13958	0.827	0.86	1
Atxn2l	0.000224	0.166199	0.707	0.648	1
Mmp14	0.000225	-0.20511	0.952	0.987	1
Apbb1ip	0.000225	-0.14096	0.544	0.647	1

Cebpa	0.00023	0.244716	0.432	0.325	1
Ddx50	0.000231	0.164601	0.765	0.702	1
Shc2	0.000231	-0.13686	0.415	0.506	1
Meg3	0.000232	-0.26174	0.857	0.906	1
Trim44	0.000232	0.122873	0.551	0.442	1
S100a4	0.000233	-0.36203	0.939	0.953	1
Erf	0.000237	0.143215	0.636	0.532	1
Ccbe1	0.000237	-0.23571	0.384	0.474	1
Rhob	0.000241	0.201921	0.912	0.859	1
Cdkn2aip	0.000241	0.162243	0.466	0.368	1
Nlgn2	0.000242	0.111198	0.439	0.339	1
Tanc1	0.000244	0.127368	0.469	0.361	1
Rps20	0.000246	0.149533	1	0.999	1
Laptm4a	0.000247	-0.10573	1	0.999	1
Tmem45a	0.000248	-0.14968	0.622	0.698	1
Angptl1	0.00025	-0.24215	0.517	0.636	1
Antxr1	0.00025	-0.16899	0.895	0.942	1
Sppl2a	0.000252	-0.16875	0.701	0.763	1
Ybx1	0.000255	0.143333	0.997	0.977	1
Sh3d21	0.000256	0.12837	0.184	0.114	1
Gm8797	0.000257	0.10352	0.279	0.19	1
Gpr153	0.000261	-0.18175	0.789	0.84	1
Plcb1	0.000263	0.102878	0.105	0.053	1
Rasgrp2	0.000264	0.172035	0.207	0.135	1
Igsf10	0.000265	-0.17519	0.884	0.926	1
Amd1	0.000266	0.138741	0.432	0.33	1
Ism1	0.000267	-0.1848	0.384	0.488	1
Hnrnpdl	0.000268	0.232352	0.864	0.819	1
Ldb1	0.000268	0.152541	0.714	0.609	1
Dll1	0.00027	0.203071	0.15	0.088	1
Lamc1	0.000272	-0.19535	0.861	0.912	1
Ankrd17	0.000272	0.183863	0.864	0.825	1
Bdh2	0.000276	-0.17482	0.493	0.567	1
Plekhh2	0.000279	0.157318	0.282	0.201	1
Arhgap35	0.00028	0.137759	0.463	0.357	1
Tmcc1	0.00028	0.110152	0.554	0.435	1
Fbln2	0.000281	-0.15086	0.929	0.982	1
Trim28	0.000289	0.139476	0.724	0.625	1
Tomm20	0.000294	0.193043	0.871	0.84	1
Atrnl1	0.000299	-0.12224	0.51	0.607	1
Tpm2	0.000299	0.18341	0.344	0.255	1
Coq10b	0.000303	0.18222	0.759	0.701	1
Snrpa	0.000306	0.110119	0.463	0.349	1

Hsph1	0.000307	0.123432	0.483	0.375	1
Txlna	0.000308	0.131101	0.541	0.434	1
Gm20342	0.000309	0.132686	0.289	0.2	1
Ccl7	0.000309	-0.30261	0.65	0.737	1
Nsd2	0.000311	0.100259	0.293	0.202	1
Vgll3	0.000313	-0.19056	0.571	0.65	1
Rpl30	0.000314	0.155477	1	0.999	1
Brd2	0.000319	0.254551	0.976	0.956	1
2810403A(	0.000323	0.151586	0.534	0.442	1
Rpl7	0.000325	0.165728	0.997	0.997	1
Pigk	0.000336	-0.14772	0.551	0.643	1
Nfkbiz	0.00034	0.21665	0.861	0.8	1
Tapbp	0.000342	-0.157	0.806	0.857	1
Dact1	0.000345	0.103604	0.32	0.221	1
Uqcc2	0.000346	-0.14783	0.799	0.873	1
Rps3a1	0.000346	0.143643	1	1	1
Txnrd1	0.000347	0.157505	0.667	0.578	1
Bbc3	0.000354	0.147664	0.303	0.216	1
Wee1	0.00036	0.112761	0.388	0.282	1
Taf1	0.000361	0.145274	0.554	0.455	1
Cnot1	0.000373	0.153071	0.626	0.536	1
Id1	0.00038	0.178363	0.418	0.327	1
Ccl11	0.000388	-0.3474	0.391	0.484	1
Rps18	0.000393	0.210542	0.997	0.992	1
Gpc4	0.0004	-0.1962	0.694	0.74	1
Akr1cl	0.000417	-0.11586	0.323	0.425	1
Csrp1	0.000417	0.109438	0.548	0.449	1
Tubb4b	0.000422	0.17133	0.912	0.863	1
Sp3	0.000424	-0.12754	0.65	0.707	1
Atp5g2	0.000433	0.150493	0.952	0.928	1
Exoc6b	0.000439	0.207658	0.514	0.414	1
Slc16a2	0.000439	0.113705	0.265	0.183	1
Csad	0.00044	0.183817	0.459	0.367	1
Bex3	0.000447	0.166798	0.374	0.291	1
Inhbb	0.00045	-0.11411	0.514	0.589	1
Msl1	0.000455	0.134157	0.653	0.567	1
Snai1	0.000461	0.2988	0.5	0.415	1
Rplp1	0.00047	0.169163	1	0.998	1
Aldh3a1	0.000473	-0.33351	0.408	0.497	1
Ankrd28	0.000482	0.194419	0.622	0.543	1
Larp1b	0.000483	0.198896	0.398	0.313	1
Btg3	0.000499	0.142831	0.398	0.308	1
Cnbp	0.0005	0.190576	0.942	0.91	1

Ppp2r2a	0.000502	0.169196	0.728	0.676	1
Klf5	0.000502	0.239718	0.361	0.277	1
Cpe	0.000513	-0.19383	0.748	0.816	1
Ripk1	0.000519	0.127054	0.721	0.627	1
AC160336.	0.000521	0.261963	0.687	0.601	1
Itpkc	0.000522	0.131608	0.418	0.322	1
Birc6	0.000525	0.129643	0.833	0.743	1
Sumo2	0.000526	0.151373	0.956	0.947	1
Snhg12	0.000526	0.168129	0.667	0.569	1
Esr1	0.000532	-0.13223	0.493	0.606	1
Raf1	0.000537	0.143046	0.704	0.645	1
Ctdspl2	0.000537	0.100469	0.449	0.344	1
Rflnb	0.000537	0.163746	0.303	0.222	1
Ctbp2	0.000539	0.155314	0.507	0.421	1
Uqcc3	0.00054	-0.10031	0.422	0.519	1
Zmym5	0.00054	0.19185	0.704	0.605	1
Hmgcs1	0.000541	0.185752	0.633	0.561	1
Kdm5b	0.000543	0.144838	0.786	0.733	1
Fxr1	0.000552	0.140406	0.718	0.647	1
Nab2	0.000559	0.245854	0.507	0.413	1
Srebf2	0.000562	0.166136	0.588	0.49	1
Hmox1	0.000562	0.254317	0.81	0.743	1
Prrc2b	0.000569	0.172513	0.786	0.743	1
Lhfp	0.000573	-0.1749	0.816	0.891	1
Huwe1	0.000574	0.139889	0.833	0.758	1
Setbp1	0.00059	0.27726	0.619	0.506	1
Nars	0.000594	0.115232	0.769	0.672	1
Baz2a	0.000598	0.154169	0.507	0.421	1
Dennd4a	0.000604	0.135852	0.534	0.423	1
Cyb5a	0.000607	-0.13718	0.942	0.973	1
Peak1	0.000612	-0.1194	0.486	0.574	1
Nop56	0.000619	0.164874	0.616	0.54	1
Ptpn2	0.00062	0.107061	0.432	0.334	1
Siah2	0.000636	0.132139	0.486	0.387	1
Ptpn12	0.000648	0.15648	0.626	0.534	1
Cspp1	0.00065	0.126755	0.439	0.342	1
Psmb8	0.000652	-0.13873	0.439	0.522	1
Kmt2e	0.000661	0.19375	0.912	0.895	1
Ntn4	0.000662	-0.17905	0.418	0.489	1
Sms	0.000669	0.163091	0.228	0.158	1
Grem1	0.000672	-0.48846	0.194	0.276	1
Sel1l	0.000678	-0.11735	0.544	0.61	1
1810013L2	0.000681	0.16053	0.646	0.551	1

Fbxo30	0.000688	0.196093	0.578	0.483	1
Nfil3	0.00069	0.180998	0.653	0.562	1
Itgb5	0.000691	-0.14235	0.81	0.912	1
Sntb2	0.000697	-0.16233	0.864	0.902	1
Rpl12	0.000699	0.168401	1	0.993	1
Ccnt1	0.000705	0.138478	0.616	0.525	1
Sspn	0.000729	-0.15424	0.52	0.582	1
Ypel3	0.000732	-0.1516	0.864	0.886	1
C1qtnf3	0.000735	-0.28554	0.486	0.565	1
Srrm2	0.000735	0.239432	0.976	0.932	1
Clk4	0.000735	0.145049	0.561	0.487	1
Ranbp2	0.000751	0.175702	0.735	0.662	1
Pcna	0.000756	0.131132	0.599	0.495	1
Mxra7	0.000768	-0.12149	0.772	0.824	1
Zbtb10	0.000782	0.128287	0.629	0.529	1
Dnajc3	0.000785	-0.12019	0.854	0.888	1
Ptbp3	0.000796	0.162973	0.776	0.714	1
Ifi207	0.000799	-0.22067	0.483	0.556	1
Vcpip1	0.0008	0.134154	0.476	0.37	1
Eef1g	0.000807	0.123635	0.959	0.945	1
Prpsap1	0.000808	0.102248	0.497	0.392	1
Qpct	0.000809	-0.20588	0.52	0.597	1
Cdk2ap1	0.000813	0.152768	0.728	0.646	1
Lamp2	0.00082	-0.12521	0.969	0.99	1
Rpl28	0.000826	0.139593	0.997	0.999	1
Rbmxl1	0.000842	0.124024	0.398	0.307	1
Fbxo33	0.000851	0.183603	0.605	0.527	1
Atp6ap1	0.000854	-0.12581	0.823	0.863	1
Ran	0.000866	0.169093	0.847	0.797	1
Kat6b	0.000873	0.139751	0.49	0.395	1
Mafk	0.000881	0.189003	0.602	0.52	1
Hexa	0.000886	-0.13834	0.867	0.919	1
Wapl	0.000903	0.172605	0.697	0.618	1
Cct2	0.000915	0.203112	0.772	0.735	1
Hmga1	0.000916	0.111806	0.156	0.098	1
Hnrnpa0	0.000924	0.219026	0.959	0.926	1
Herc4	0.000929	0.116646	0.405	0.308	1
Cul1	0.000945	0.194616	0.714	0.642	1
Vkorc1	0.000966	-0.11978	0.898	0.918	1
Strn3	0.000969	0.141747	0.847	0.802	1
Map2k2	0.000979	0.135645	0.789	0.734	1
Spen	0.000983	0.136425	0.537	0.46	1
Mob3c	0.000989	0.104179	0.398	0.31	1

Dido1	0.000993	0.168957	0.52	0.427	1
Derl2	0.000994	-0.10655	0.435	0.531	1
Klf9	0.001013	0.259049	0.963	0.976	1
Prelp	0.001013	-0.2025	0.701	0.782	1
Qk	0.001028	0.159077	0.942	0.895	1
Cadm3	0.001043	-0.19588	0.537	0.618	1
Ier5l	0.001046	0.244133	0.422	0.337	1
Tcerg1	0.001048	0.108882	0.466	0.366	1
Atp5g1	0.001058	0.163689	0.861	0.834	1
Foxp2	0.00107	0.108123	0.18	0.117	1
Capns1	0.001071	-0.12798	0.915	0.947	1
Efcab14	0.001073	0.131183	0.565	0.476	1
Clip1	0.001076	-0.12364	0.687	0.762	1
Mylk	0.00108	0.120084	0.303	0.221	1
H2afx	0.001084	0.165808	0.35	0.262	1
Tspan4	0.001085	-0.15873	0.65	0.713	1
Scarb2	0.001089	0.193177	0.544	0.467	1
Yif1b	0.00111	-0.1152	0.582	0.635	1
Rgs2	0.00111	0.260139	0.248	0.182	1
mt-Nd3	0.001124	-0.14178	0.942	0.989	1
C4b	0.001125	-0.32472	0.68	0.759	1
Adcy1	0.001126	-0.16727	0.259	0.348	1
Gpbp1	0.001135	0.156238	0.741	0.692	1
Srsf5	0.001139	0.203608	0.935	0.891	1
Bri3	0.001146	-0.11687	0.915	0.931	1
Pole4	0.001151	0.142346	0.588	0.493	1
Rin2	0.001153	-0.19234	0.711	0.763	1
Cct3	0.001154	0.123719	0.616	0.513	1
Etv1	0.001155	0.119809	0.204	0.134	1
Rnf19a	0.00116	0.126795	0.67	0.56	1
Rrad	0.001164	0.12207	0.33	0.237	1
Rps7	0.001168	0.139585	1	0.992	1
1600020E(	0.001179	0.10297	0.286	0.208	1
Mtdh	0.001184	0.130996	0.932	0.893	1
Zfp445	0.001194	0.114066	0.391	0.304	1
Plk2	0.001198	0.141253	0.418	0.324	1
Lhfpl2	0.001203	-0.14389	0.493	0.569	1
Plekha1	0.001204	-0.12499	0.476	0.542	1
Vapa	0.001205	0.146623	0.925	0.881	1
Osr2	0.001214	-0.10531	0.612	0.723	1
Spag9	0.001226	0.215547	0.959	0.946	1
Postn	0.001238	0.109881	0.66	0.561	1
Sfr1	0.001246	0.153777	0.969	0.942	1

Ep300	0.001248	0.198337	0.697	0.616	1
Capza1	0.001255	0.107106	0.673	0.569	1
Brd8	0.001257	0.142242	0.507	0.414	1
Tcp1	0.001262	0.1146	0.721	0.661	1
Med13l	0.001271	0.172279	0.833	0.785	1
Gspt1	0.00128	0.190024	0.755	0.681	1
Fam102b	0.00129	-0.14371	0.823	0.888	1
Gsto1	0.0013	0.104701	0.48	0.39	1
Smarcc2	0.001306	0.129746	0.629	0.545	1
Rbm18	0.001339	0.142732	0.561	0.481	1
Spty2d1	0.00134	0.149261	0.772	0.712	1
Pcbp2	0.001346	0.130237	0.976	0.97	1
Hip1	0.00135	0.121052	0.265	0.189	1
Daam1	0.001355	0.144014	0.439	0.35	1
Sp4	0.001356	0.101745	0.221	0.152	1
Rps23	0.001357	0.141593	1	0.997	1
Ldha	0.00136	0.206449	0.796	0.765	1
Zbtb11	0.00137	0.186443	0.524	0.452	1
Rbm3	0.001371	0.140442	0.99	0.979	1
Bace2	0.001373	-0.17775	0.493	0.571	1
Ergic3	0.001374	-0.1135	0.908	0.918	1
Kif5b	0.001376	0.138192	0.915	0.861	1
Frem1	0.001377	0.126839	0.167	0.106	1
Anp32e	0.001383	0.153506	0.639	0.558	1
Sema3a	0.001399	-0.21129	0.255	0.345	1
Tardbp	0.001416	0.130671	0.704	0.603	1
Anxa5	0.001418	-0.14612	0.976	0.989	1
Ist1	0.001438	0.123708	0.721	0.63	1
Elavl1	0.001457	0.132972	0.816	0.739	1
Serpinb6a	0.001463	-0.10542	0.942	0.974	1
Ppfibp1	0.001466	-0.11662	0.741	0.854	1
Nkd1	0.001467	0.194172	0.323	0.253	1
Dync1li1	0.00147	0.116162	0.602	0.523	1
Cstb	0.001479	0.148248	0.912	0.875	1
Kcnq1ot1	0.001489	0.35141	0.697	0.643	1
2010111I0	0.001506	0.157012	0.446	0.348	1
Gmfb	0.001514	-0.10331	0.582	0.664	1
Jarid2	0.001545	0.107671	0.483	0.386	1
Plk3	0.001549	0.123522	0.456	0.372	1
Cyp27a1	0.001554	-0.10016	0.238	0.319	1
Ostc	0.001564	-0.11484	0.857	0.906	1
Pgpep1	0.001567	-0.12497	0.432	0.535	1
Ednra	0.001577	0.125367	0.235	0.167	1

Fkbp10	0.001582	-0.10888	0.772	0.836	1
Stk40	0.001593	0.207834	0.653	0.581	1
Lama4	0.001598	-0.16556	0.639	0.722	1
Nup153	0.001599	0.106899	0.561	0.46	1
Sik1	0.00161	0.226509	0.639	0.563	1
Chd6	0.001633	0.128478	0.595	0.502	1
Pou3f1	0.001639	0.105883	0.252	0.178	1
Ino80	0.001642	0.118204	0.449	0.362	1
Syncrip	0.001642	0.16581	0.861	0.793	1
Dynlrb1	0.001659	-0.11079	0.929	0.933	1
Rpl10a	0.001681	0.15559	1	0.995	1
Mfsd11	0.001685	-0.12095	0.514	0.597	1
Glmp	0.00169	-0.12473	0.701	0.767	1
Rpl23a	0.001691	0.148203	0.986	0.981	1
Chd1	0.001695	0.15518	0.629	0.54	1
Rps27a	0.001698	0.146818	1	0.994	1
Rpl22l1	0.0017	0.200145	0.973	0.957	1
Creb3l1	0.001705	-0.12696	0.816	0.901	1
Cnot6	0.001716	0.12128	0.738	0.66	1
Frmd6	0.001719	-0.17581	0.782	0.817	1
Cul3	0.001758	0.110617	0.82	0.742	1
Gxylt2	0.001758	-0.14386	0.714	0.795	1
Entpd2	0.001763	-0.16005	0.776	0.848	1
Setd2	0.00177	0.127217	0.701	0.609	1
Atp5b	0.001789	0.155	0.973	0.945	1
Pgr	0.001818	-0.22551	0.466	0.544	1
Dazap1	0.001871	0.123399	0.707	0.598	1
Igf1r	0.001892	0.216855	0.884	0.791	1
Ifitm3	0.001902	-0.11243	0.983	0.993	1
Ikzf5	0.001904	0.143751	0.49	0.397	1
Rnf145	0.001924	-0.10075	0.531	0.62	1
Spon1	0.001926	0.329025	0.629	0.574	1
Sf1	0.001952	0.122154	0.765	0.703	1
Hnrnpa3	0.001973	0.14306	0.949	0.929	1
Fdps	0.001974	0.147543	0.381	0.311	1
Ptov1	0.001993	0.116275	0.864	0.816	1
Serf2	0.00201	-0.11495	0.986	0.997	1
Med13	0.002018	0.155182	0.878	0.841	1
Pnn	0.002056	0.196107	0.616	0.522	1
Sat1	0.002056	-0.18949	0.922	0.95	1
Kcnk2	0.002063	-0.14458	0.119	0.189	1
Hmgb2	0.002066	0.237073	0.582	0.499	1
Itgav	0.002072	-0.12266	0.707	0.777	1

Fosl2	0.00208	0.262343	0.83	0.809	1
Rap1b	0.002089	0.123672	0.969	0.935	1
Chst12	0.00209	-0.10577	0.646	0.708	1
Yy1	0.002096	0.191514	0.867	0.803	1
Dpysl2	0.002108	-0.13735	0.844	0.869	1
Atg101	0.002117	0.105546	0.517	0.414	1
Srpk1	0.002124	0.168474	0.551	0.467	1
Mepce	0.002136	0.220362	0.575	0.528	1
Lsamp	0.002141	-0.14957	0.354	0.434	1
Kdelr2	0.002154	-0.14557	0.912	0.96	1
Cbx1	0.002163	0.149556	0.704	0.626	1
mt-Co1	0.002195	-0.1531	1	1	1
Naa15	0.002229	0.159737	0.687	0.617	1
Clk1	0.002264	0.249109	0.942	0.904	1
Iqsec1	0.002265	0.143579	0.514	0.433	1
Fzd1	0.002287	0.109964	0.673	0.588	1
Rpl26	0.002288	0.116608	1	0.992	1
Ssb	0.002303	0.146551	0.871	0.843	1
Gt(ROSA)2	0.002324	0.107896	0.296	0.22	1
Trib1	0.002325	0.127185	0.81	0.716	1
Mapre1	0.002327	0.167192	0.912	0.902	1
Ppp1r10	0.002362	0.3035	0.833	0.801	1
Ppp1r18	0.002363	0.140097	0.667	0.57	1
Nt5e	0.002375	-0.16144	0.347	0.428	1
Luc7l	0.002388	0.110238	0.524	0.422	1
Casp12	0.002422	-0.10729	0.391	0.46	1
Cox4i1	0.00244	0.169745	0.986	0.985	1
Tubb5	0.002446	0.181315	0.939	0.922	1
Tle4	0.00245	0.148521	0.493	0.418	1
Gnai1	0.002455	0.10779	0.262	0.19	1
March7	0.002466	0.142779	0.65	0.585	1
Immp1l	0.002472	0.110548	0.616	0.533	1
Lman1	0.002473	-0.13872	0.833	0.88	1
Son	0.0025	0.125343	0.99	0.962	1
Pgk1	0.002533	0.127485	0.66	0.585	1
Pnisr	0.002555	0.168237	0.568	0.49	1
C1qtnf6	0.002555	-0.13253	0.755	0.842	1
Cldn10	0.002567	-0.25281	0.65	0.691	1
Pttg1ip	0.002572	-0.13079	0.823	0.862	1
2310022B(	0.002595	-0.15087	0.731	0.773	1
S100a16	0.002615	-0.12566	0.765	0.798	1
Lrrc17	0.002622	0.104499	0.265	0.195	1
Arid2	0.002635	0.143745	0.643	0.558	1

Cd248	0.002689	-0.15601	0.711	0.809	1
Jag1	0.0027	0.100396	0.238	0.169	1
H2-T23	0.002721	-0.15494	0.724	0.78	1
Srsf7	0.002723	0.149522	0.731	0.656	1
Sos2	0.002766	0.108868	0.524	0.418	1
Rbms3	0.002769	-0.15574	0.884	0.893	1
Kdm7a	0.002783	0.162555	0.83	0.78	1
Traf4	0.002812	0.102429	0.235	0.167	1
G3bp2	0.002822	0.116985	0.704	0.644	1
Senp6	0.002836	0.134838	0.844	0.782	1
Elf2	0.002845	0.171447	0.799	0.743	1
Scarf2	0.002856	-0.18586	0.844	0.906	1
Gstm2	0.002857	-0.13831	0.639	0.681	1
Peli1	0.002861	0.108724	0.861	0.774	1
Copz2	0.002868	-0.14331	0.806	0.839	1
Id2	0.002882	0.222764	0.673	0.613	1
Dnajc10	0.002901	-0.12083	0.534	0.606	1
Prrc1	0.002909	-0.10794	0.588	0.668	1
Rpl37	0.002917	0.149511	1	0.998	1
Pik3r3	0.002936	0.109805	0.17	0.113	1
Idh1	0.002937	-0.12326	0.769	0.8	1
Rps11	0.002941	0.130051	0.997	0.995	1
Phldb2	0.002952	-0.21903	0.541	0.614	1
Wac	0.002961	0.120062	0.837	0.785	1
Ahsa1	0.002965	0.123823	0.582	0.513	1
Epb41	0.002982	0.101106	0.31	0.235	1
Ier3ip1	0.002995	-0.12702	0.799	0.838	1
Camsap2	0.003044	0.148002	0.473	0.391	1
Prr13	0.003045	-0.10962	0.735	0.798	1
Srsf3	0.003061	0.222967	0.939	0.92	1
Sesn2	0.003063	0.10898	0.289	0.219	1
Cygb	0.003077	0.105272	0.418	0.321	1
Ube2b	0.003104	0.13867	0.949	0.912	1
Erc1	0.00312	0.142238	0.425	0.36	1
Ggnbp2	0.003166	0.167149	0.813	0.74	1
Cycs	0.003169	0.166461	0.633	0.582	1
Sod2	0.003174	0.23932	0.653	0.597	1
Tspan5	0.00318	-0.11134	0.517	0.566	1
Ankrd12	0.003215	0.18979	0.837	0.808	1
Nup62	0.00328	0.116313	0.361	0.285	1
Mat2a	0.003301	0.195386	0.925	0.928	1
Zc3h13	0.003309	0.127448	0.5	0.423	1
Slc29a1	0.003332	-0.15694	0.707	0.786	1

Rpl5	0.003332	0.113754	0.993	0.992	1
Rsrp1	0.003335	0.167503	0.99	0.976	1
Mfap5	0.003335	-0.18449	0.599	0.68	1
mt-Nd5	0.003339	-0.1739	0.952	0.981	1
Srsf2	0.00334	0.16019	0.969	0.959	1
Jmjd6	0.003343	0.108542	0.565	0.5	1
Polr2a	0.003352	0.161215	0.776	0.716	1
Ckb	0.003355	0.186184	0.697	0.638	1
Kpna4	0.00336	0.110067	0.721	0.648	1
Deptor	0.003366	-0.16809	0.622	0.662	1
Entpd1	0.003381	-0.13782	0.395	0.464	1
Adamts5	0.003392	-0.24233	0.68	0.757	1
Mafb	0.003415	0.112388	0.707	0.631	1
Epdr1	0.003421	-0.1023	0.364	0.435	1
Hmgn1	0.003456	0.118845	0.918	0.911	1
Bag3	0.003459	0.156386	0.864	0.819	1
Ywhaq	0.003513	0.182798	0.946	0.908	1
Rest	0.003532	0.180514	0.67	0.588	1
Zcchc6	0.003551	0.130689	0.762	0.705	1
Rabep1	0.003574	-0.11584	0.517	0.581	1
Safb2	0.003587	0.18236	0.595	0.521	1
Tmem183a	0.003619	0.125928	0.415	0.339	1
Klhl21	0.003638	0.138733	0.554	0.481	1
Egfr	0.003673	-0.18988	0.653	0.704	1
Akirin1	0.003677	0.124403	0.517	0.444	1
Dhrs7	0.003688	-0.10453	0.711	0.763	1
Rps10	0.003691	0.110576	1	0.997	1
Rplp2	0.003705	0.141414	0.997	0.994	1
Cyb5r3	0.003736	-0.13874	0.816	0.854	1
Mmp11	0.003746	-0.14904	0.374	0.451	1
Gatad2a	0.003758	0.109423	0.571	0.513	1
P4ha1	0.003761	-0.11048	0.616	0.701	1
Tor1b	0.003823	-0.10254	0.544	0.598	1
Wwc2	0.003847	0.174435	0.714	0.686	1
Axl	0.003848	-0.16871	0.878	0.933	1
Gnl2	0.00386	0.14271	0.405	0.324	1
Bbx	0.003861	0.103963	0.418	0.332	1
Mef2d	0.003862	0.184653	0.68	0.623	1
Scpep1	0.003862	-0.14445	0.833	0.91	1
mt-Nd1	0.003885	-0.15234	1	1	1
Pds5a	0.003906	0.123429	0.619	0.536	1
Isg15	0.003915	0.189173	0.224	0.158	1
Ecm2	0.003938	-0.14242	0.816	0.896	1

Ube2o	0.004044	0.169169	0.446	0.379	1
Stam2	0.004063	0.100624	0.452	0.378	1
Katna1	0.004121	0.116102	0.497	0.405	1
Ctsd	0.004163	-0.12814	0.949	0.975	1
Pbx1	0.004171	-0.13664	0.922	0.948	1
4921524J1	0.004189	0.127383	0.432	0.367	1
Hspg2	0.004217	-0.15504	0.867	0.917	1
B2m	0.004246	-0.16771	0.983	0.994	1
Fmr1	0.004266	0.110736	0.653	0.575	1
Cic	0.004274	0.17046	0.418	0.354	1
Dap3	0.004359	0.107435	0.585	0.499	1
Zfp36l1	0.004366	0.169439	0.993	0.991	1
Tor1aip2	0.004403	0.111595	0.755	0.679	1
Chd4	0.00442	0.160992	0.932	0.905	1
Snx18	0.004427	-0.10193	0.626	0.696	1
Ube2e3	0.004466	0.111614	0.728	0.648	1
Pcbp1	0.004467	0.118141	0.918	0.904	1
Atn1	0.004476	0.120943	0.524	0.435	1
Plpp1	0.004487	-0.16493	0.677	0.726	1
Cpsf7	0.004498	0.11582	0.469	0.385	1
Uvrag	0.004529	0.102041	0.541	0.455	1
Sdc1	0.004649	0.200258	0.51	0.435	1
Notum	0.00466	0.388853	0.112	0.069	1
Stip1	0.004675	0.115993	0.405	0.336	1
Fez1	0.004717	-0.12787	0.344	0.41	1
Nectin2	0.00474	0.130288	0.218	0.157	1
Ubxn1	0.004744	0.102237	0.796	0.766	1
Mtss1l	0.004753	0.161625	0.418	0.346	1
Cct7	0.004784	0.129069	0.724	0.684	1
Fgfr2	0.004831	0.18226	0.524	0.46	1
Rpl4	0.004866	0.120071	0.997	0.98	1
Arid1a	0.004875	0.152436	0.793	0.734	1
Parp3	0.004909	-0.10181	0.469	0.527	1
Tcf7	0.004925	0.179393	0.173	0.12	1
Plec	0.00493	0.210869	0.895	0.817	1
Cdk5r1	0.004998	0.100142	0.194	0.136	1
Zcrb1	0.005	0.154771	0.731	0.682	1
Ncoa7	0.005034	-0.14812	0.616	0.683	1
Ubn1	0.005046	0.105391	0.745	0.701	1
Rangap1	0.005067	0.124335	0.282	0.22	1
Bmp4	0.005069	0.554214	0.531	0.47	1
Fam129b	0.005195	0.117678	0.554	0.488	1
Dusp7	0.005201	0.129071	0.442	0.368	1

Gm26532	0.005212	0.178423	0.486	0.42	1
Fndc1	0.005216	-0.17221	0.527	0.59	1
Fam180a	0.005219	-0.2463	0.412	0.473	1
Atrx	0.00523	0.160008	0.871	0.85	1
Srp9	0.005238	0.12019	0.888	0.878	1
Cdk17	0.005271	0.110508	0.262	0.199	1
Rps4x	0.005279	0.115568	1	0.998	1
Rcor1	0.005327	0.121606	0.537	0.466	1
Cpsf6	0.005343	0.13042	0.585	0.516	1
Galnt10	0.005357	-0.13048	0.374	0.427	1
Rpl7a	0.005399	0.125964	0.993	0.986	1
Camk2d	0.005522	-0.12881	0.806	0.83	1
Cox7a2l	0.005524	-0.11533	0.884	0.928	1
1700017B(	0.005559	0.118413	0.347	0.273	1
Nrp2	0.005586	0.249499	0.646	0.57	1
Klk1b24	0.005665	-0.43616	0.531	0.589	1
Rb1	0.005682	0.138537	0.255	0.188	1
Rsbn1l	0.005742	0.11356	0.524	0.436	1
Selenop	0.005766	-0.12027	0.925	0.975	1
Arhgap15	0.005779	0.102328	0.33	0.256	1
Rcn2	0.005788	0.150261	0.789	0.762	1
Evl	0.005806	0.114384	0.371	0.288	1
Abca1	0.005814	-0.11049	0.799	0.821	1
Gsk3b	0.005849	0.11384	0.912	0.863	1
Trrap	0.005931	0.148014	0.554	0.494	1
mt-Nd2	0.005958	-0.1647	1	0.999	1
Tpm3	0.005976	0.143573	0.881	0.886	1
Cnpy2	0.006017	-0.10551	0.84	0.896	1
Brwd1	0.006102	0.113424	0.626	0.547	1
Tcp11l2	0.006102	0.149948	0.68	0.606	1
Anxa1	0.006104	-0.12326	0.949	0.976	1
AW54987	0.006111	-0.10887	0.446	0.514	1
Smc3	0.006189	0.157869	0.66	0.614	1
H13	0.006204	-0.1033	0.697	0.754	1
Eif3a	0.006217	0.128607	0.905	0.884	1
Ackr3	0.006222	-0.20386	0.724	0.803	1
Eif4g2	0.006266	0.130239	0.986	0.983	1
Sin3b	0.006291	0.126582	0.731	0.67	1
Sf3b2	0.0063	0.105977	0.85	0.788	1
Zrsr2	0.006302	0.101008	0.534	0.461	1
Ephx1	0.006322	-0.11425	0.565	0.629	1
March5	0.006328	0.131406	0.619	0.565	1
Cfap36	0.006393	0.112868	0.497	0.421	1

Ackr1	0.00641	-0.12069	0.388	0.458	1
Ddit4	0.006547	0.137493	0.354	0.281	1
Pdlim4	0.006617	0.117532	0.514	0.439	1
Plin3	0.006625	-0.10759	0.588	0.638	1
Vat1	0.006626	-0.1016	0.878	0.901	1
Ehd2	0.006695	-0.11299	0.779	0.846	1
Igf1	0.006701	-0.14712	0.881	0.896	1
Itgb3	0.006899	-0.1058	0.31	0.378	1
Mcrip1	0.006924	-0.11286	0.776	0.854	1
Ahcyl2	0.006933	0.129473	0.469	0.389	1
Grb10	0.006981	0.113252	0.759	0.715	1
Snrpg	0.007003	0.10317	0.915	0.889	1
Fam133b	0.007106	0.118398	0.588	0.507	1
Pabpn1	0.007107	0.114454	0.439	0.363	1
Adam9	0.007112	-0.11645	0.534	0.581	1
Myc	0.007136	0.135106	0.599	0.535	1
Fermt2	0.007189	0.108727	0.871	0.785	1
Pcsk6	0.007218	-0.24457	0.384	0.444	1
Tmod3	0.007267	0.130979	0.782	0.746	1
Rasa1	0.007311	0.139581	0.663	0.596	1
Usp7	0.007317	0.120576	0.718	0.67	1
Nolc1	0.00733	0.122613	0.446	0.378	1
Rpl17	0.00736	0.105916	1	0.992	1
Selenos	0.007422	-0.11687	0.861	0.939	1
Gnas	0.007451	0.134765	0.997	0.998	1
Txnip	0.007489	-0.17445	0.687	0.733	1
Rnd3	0.007525	0.126539	0.738	0.654	1
Rsbn1	0.007592	0.110166	0.517	0.431	1
Rprd2	0.007702	0.105507	0.446	0.376	1
Rpl21	0.007768	0.115238	0.993	0.997	1
Serbp1	0.007799	0.139924	0.963	0.937	1
Sun2	0.00785	0.117344	0.643	0.562	1
Tgfbr2	0.00792	-0.12506	0.912	0.956	1
Rpsa	0.008028	0.137192	1	0.999	1
Dpep1	0.008041	-0.14079	0.741	0.809	1
Eln	0.008067	-0.20186	0.857	0.894	1
Lars2	0.008078	-0.35756	0.748	0.803	1
Poldip3	0.008126	0.101866	0.609	0.519	1
Inmt	0.008133	-0.268	0.265	0.335	1
Lrrtm3	0.00814	-0.19268	0.303	0.369	1
Ldlr	0.00816	0.167511	0.401	0.338	1
Rpl22	0.008173	0.101605	0.993	0.986	1
Cdon	0.008209	-0.16607	0.388	0.45	1

Ago2	0.00822	0.124223	0.701	0.62	1
Tnc	0.008228	0.35012	0.235	0.177	1
Rpl10	0.008231	0.105043	1	0.999	1
Mrc2	0.008241	-0.12446	0.759	0.809	1
Axin2	0.008311	-0.15192	0.306	0.371	1
Olfml2b	0.008374	-0.1171	0.755	0.83	1
Ash1l	0.008525	0.138509	0.935	0.92	1
Cldnd1	0.008613	0.105404	0.663	0.585	1
Camsap1	0.008651	0.106055	0.35	0.279	1
Eef1b2	0.008669	0.103963	0.983	0.981	1
Des	0.008671	-0.20285	0.429	0.496	1
Smc4	0.00869	0.117411	0.316	0.257	1
Col15a1	0.00877	-0.10642	0.803	0.897	1
Brd4	0.008806	0.133152	0.81	0.754	1
Cdkn2d	0.008892	0.112397	0.313	0.247	1
Tpm1	0.008978	0.268101	0.759	0.723	1
Dst	0.009061	0.175934	0.823	0.77	1
Rtraf	0.0091	0.100186	0.874	0.862	1
Ftl1	0.009138	0.103714	1	1	1
Smchd1	0.00916	0.196966	0.592	0.553	1
Eif5	0.009184	0.171204	0.99	0.971	1
Mier3	0.009217	0.114361	0.49	0.411	1
Slc25a25	0.009262	0.107231	0.534	0.447	1
Trim16	0.009289	0.101158	0.371	0.304	1
Sema4a	0.009305	0.13196	0.405	0.332	1
Heg1	0.009587	-0.11242	0.473	0.553	1
Selenom	0.00961	-0.1013	0.939	0.962	1
Prpf38b	0.009615	0.121381	0.779	0.725	1
Fn1	0.009634	-0.12632	0.748	0.796	1
Vgll4	0.009671	0.118296	0.422	0.344	1
Irx2	0.009718	-0.1089	0.616	0.68	1
Vit	0.009806	-0.12857	0.224	0.298	1
Enpp2	0.009812	0.241416	0.714	0.687	1
D16Ertd47	0.009819	0.100945	0.279	0.216	1
Selenow	0.010014	-0.10093	0.956	0.974	1
Rell1	0.010018	0.167474	0.527	0.47	1
Cpeb2	0.010051	0.156808	0.51	0.447	1
Dhrs3	0.010075	-0.12192	0.493	0.537	1
Jpt1	0.01022	0.107403	0.861	0.84	1
Sar1a	0.01023	-0.10629	0.901	0.937	1
Birc2	0.010297	0.161327	0.466	0.403	1
Gm2a	0.01034	-0.11847	0.575	0.622	1
Ogt	0.010341	0.123202	0.626	0.546	1

Fam105a	0.010387	-0.16699	0.459	0.51	1
Ilf2	0.01039	0.176426	0.612	0.547	1
Brpf1	0.01047	0.108229	0.259	0.196	1
Il1r1	0.01052	-0.14188	0.571	0.635	1
Fbxo32	0.01063	0.208931	0.476	0.418	1
Zfp281	0.010657	0.155083	0.34	0.286	1
Metrnl	0.010685	-0.13615	0.759	0.822	1
Rps6	0.010695	0.10918	0.993	0.987	1
Tecr	0.010822	0.101969	0.796	0.755	1
Ugp2	0.010839	-0.13656	0.724	0.769	1
Mkx	0.01088	-0.12628	0.405	0.481	1
Wipi2	0.010896	0.156065	0.629	0.541	1
Nr3c1	0.010904	0.300048	0.84	0.83	1
Ptprd	0.010987	0.108391	0.364	0.298	1
Pthlh	0.011114	-0.16698	0.367	0.441	1
Pde4d	0.011187	0.177264	0.466	0.418	1
Tgfb1	0.011381	0.118918	0.612	0.528	1
Rabac1	0.011415	-0.1233	0.939	0.954	1
Mafg	0.011537	0.120913	0.714	0.636	1
Ddx24	0.011622	0.121295	0.667	0.616	1
Rgmb	0.011669	-0.11449	0.554	0.618	1
Paf1	0.01175	0.111795	0.476	0.405	1
Magoh	0.011781	0.178752	0.68	0.662	1
Ythdc1	0.011786	0.195562	0.704	0.631	1
Rrm2b	0.011822	0.101402	0.507	0.439	1
Hectd1	0.01184	0.144592	0.837	0.832	1
Prkar1a	0.011982	0.10814	0.895	0.883	1
Mxra8	0.011997	-0.10768	0.884	0.937	1
Por	0.01207	-0.13359	0.643	0.668	1
Reep3	0.012091	0.152314	0.949	0.925	1
Csrp2	0.012174	0.125936	0.582	0.52	1
Plagl1	0.012408	0.103246	0.378	0.309	1
Dpt	0.012531	-0.1205	0.895	0.931	1
Zfp148	0.012569	0.136372	0.68	0.619	1
Lrrn4cl	0.0126	-0.1271	0.544	0.594	1
Arhgdib	0.012646	0.112162	0.357	0.291	1
Psmd4	0.012715	0.103873	0.745	0.712	1
Abhd2	0.01282	0.195141	0.541	0.478	1
Cyp51	0.012848	0.160593	0.354	0.293	1
Odc1	0.012859	0.171066	0.605	0.575	1
Atox1	0.01288	-0.12086	0.918	0.941	1
Syf2	0.012983	0.113708	0.741	0.714	1
Tax1bp1	0.013012	0.10796	0.935	0.903	1

Klk1b21	0.013049	-0.28156	0.595	0.654	1
Esyt1	0.013058	0.176214	0.582	0.504	1
Plac8	0.013064	0.192071	0.139	0.094	1
Srsf1	0.013067	0.116564	0.541	0.495	1
Tagln	0.01307	0.272932	0.245	0.184	1
Adam17	0.013135	-0.10488	0.646	0.668	1
Usp19	0.013176	0.100731	0.531	0.469	1
Eny2	0.01321	0.105016	0.813	0.779	1
Taf15	0.013259	0.102157	0.378	0.309	1
Cope	0.013262	-0.11133	0.799	0.827	1
Matn2	0.0133	0.11143	0.578	0.502	1
Gatad2b	0.013544	0.13132	0.762	0.728	1
Oser1	0.013937	0.168967	0.578	0.516	1
Atp13a3	0.014214	0.123625	0.653	0.573	1
Tram1	0.01448	-0.11645	0.789	0.816	1
Zdhhc20	0.014512	0.10368	0.735	0.673	1
Antxr2	0.014558	-0.14294	0.735	0.782	1
Ep400	0.014636	0.124824	0.728	0.664	1
Rpl35	0.014763	0.128204	1	0.988	1
C1s1	0.014916	-0.1089	0.741	0.807	1
9530068E(	0.014949	-0.11161	0.823	0.882	1
Tpbg	0.014977	0.161676	0.609	0.532	1
Nop53	0.015006	0.121753	0.816	0.785	1
Eapp	0.01504	-0.10074	0.439	0.484	1
Mkln1	0.015115	0.153738	0.762	0.703	1
Klk1b1	0.015165	-0.24766	0.259	0.324	1
Naaa	0.015317	-0.11414	0.52	0.573	1
Rbfox2	0.015335	0.100429	0.738	0.688	1
Gsk3a	0.015375	0.123112	0.728	0.672	1
Glrx5	0.015526	0.126277	0.622	0.526	1
Pa2g4	0.015552	0.123868	0.588	0.532	1
Alkbh5	0.015581	0.191767	0.796	0.782	1
Ifi27l2a	0.015726	0.377808	0.272	0.213	1
Loxl1	0.015806	-0.14539	0.823	0.878	1
Eif4e	0.015807	0.117308	0.806	0.758	1
Tmed5	0.015874	0.18124	0.633	0.593	1
Anapc13	0.016195	-0.10027	0.67	0.715	1
Hdgf	0.016232	0.162869	0.905	0.861	1
Reck	0.016338	-0.14827	0.721	0.719	1
Ubn2	0.016549	0.176045	0.752	0.705	1
Lsp1	0.016648	-0.13042	0.588	0.646	1
Sox5	0.016814	-0.11814	0.211	0.258	1
Puf60	0.016964	0.109879	0.65	0.592	1

Bptf	0.017019	0.119653	0.895	0.863	1
9930021J0	0.017021	0.1335	0.639	0.569	1
Smc1a	0.017243	0.151318	0.639	0.586	1
Rcn1	0.017328	-0.10346	0.776	0.86	1
Cask	0.017457	0.107059	0.432	0.362	1
Pdcd4	0.017717	0.129385	0.769	0.713	1
Nova1	0.017834	-0.10106	0.425	0.47	1
Nbea	0.017842	0.101253	0.517	0.47	1
Eif1b	0.017996	0.100827	0.769	0.722	1
Adamtsl5	0.018046	-0.10737	0.551	0.581	1
Fbxw7	0.018132	0.101897	0.33	0.27	1
Slc27a1	0.018269	-0.11246	0.456	0.505	1
Rad23b	0.018393	0.102147	0.765	0.735	1
Igsf3	0.018855	0.120957	0.415	0.353	1
Pura	0.019296	0.13082	0.881	0.848	1
Iws1	0.019415	0.138685	0.459	0.396	1
Rbp1	0.019424	0.20372	0.296	0.236	1
Gm32219	0.019445	-0.11317	0.252	0.296	1
Arid3a	0.019459	0.111737	0.228	0.177	1
Arhgef1	0.019482	0.116369	0.537	0.467	1
Serpina3n	0.019551	-0.14434	0.527	0.587	1
Luc7l3	0.019565	0.173063	0.755	0.712	1
Eif4h	0.019805	0.131199	0.81	0.774	1
Rbm27	0.019826	0.112544	0.544	0.484	1
Gclc	0.019977	0.142485	0.585	0.506	1
Tfe3	0.020089	0.111017	0.578	0.492	1
Noct	0.020091	0.20697	0.32	0.274	1
Col18a1	0.020159	-0.10495	0.65	0.728	1
Gpatch8	0.020582	0.134421	0.52	0.468	1
Usp37	0.020746	0.104328	0.282	0.226	1
Rbm25	0.020897	0.109235	0.901	0.872	1
Cpz	0.021111	-0.10193	0.673	0.741	1
Vdac2	0.021466	0.107998	0.765	0.705	1
Tmem158	0.021532	0.1253	0.493	0.408	1
Kcmf1	0.021676	0.123946	0.755	0.699	1
Atxn7l3	0.021709	0.105995	0.449	0.383	1
Ctnnb1	0.021877	0.13535	0.932	0.922	1
Slc25a4	0.021897	0.111162	0.956	0.932	1
Dact3	0.021939	0.10333	0.408	0.345	1
Mpc1	0.021999	0.109591	0.786	0.751	1
Saraf	0.022607	0.119451	0.724	0.693	1
Rpn2	0.022663	-0.10587	0.847	0.901	1
Rock1	0.022824	0.121987	0.895	0.832	1

Sptssa	0.022994	0.101661	0.738	0.705	1
Hpgd	0.023016	-0.11814	0.391	0.456	1
Gm1673	0.023019	0.123426	0.272	0.221	1
Spred1	0.023139	0.136036	0.694	0.648	1
Brd1	0.023301	0.147486	0.694	0.64	1
Ampd3	0.023339	-0.10088	0.374	0.427	1
Asxl1	0.023518	0.116298	0.391	0.33	1
Col16a1	0.023531	-0.16743	0.915	0.925	1
Ubr5	0.023625	0.151376	0.718	0.691	1
Shox2	0.023785	0.123884	0.599	0.549	1
Fndc3b	0.023996	-0.10576	0.912	0.95	1
Myh9	0.024098	0.175791	0.721	0.671	1
Rora	0.024217	-0.10025	0.912	0.933	1
Senp2	0.024316	0.173972	0.599	0.546	1
Cbfa2t3	0.02434	0.118856	0.204	0.154	1
Cd55	0.024616	-0.18325	0.741	0.798	1
Rela	0.024617	0.106748	0.67	0.625	1
Dusp5	0.024883	0.124322	0.422	0.367	1
Has2	0.024965	-0.10591	0.497	0.559	1
Tmem47	0.025156	-0.12266	0.619	0.661	1
Grem2	0.025374	-0.17872	0.31	0.361	1
Ccdc80	0.025433	-0.11723	0.918	0.957	1
Pros1	0.025438	-0.11134	0.844	0.905	1
Mndal	0.025511	-0.10003	0.463	0.526	1
Ube2n	0.025598	0.111687	0.769	0.745	1
Nfkb1	0.025673	0.124278	0.901	0.894	1
Dhx9	0.025733	0.126181	0.619	0.538	1
Myl9	0.025801	0.145878	0.306	0.25	1
Lgals3	0.025848	-0.26156	0.952	0.949	1
Luzp1	0.02591	0.157853	0.667	0.608	1
Vasp	0.025994	0.102136	0.497	0.433	1
Gabbr1	0.026164	0.108013	0.367	0.31	1
Atp9a	0.026256	-0.1341	0.66	0.654	1
Ppp1r2	0.026324	0.118677	0.918	0.888	1
St6galnac6	0.027023	-0.10154	0.514	0.552	1
Cacybp	0.027067	0.111979	0.531	0.46	1
Dnajb4	0.027171	0.12217	0.796	0.748	1
Haus8	0.027195	0.100231	0.296	0.245	1
Cdk2ap2	0.027324	0.170787	0.69	0.66	1
Ppp2r2d	0.027397	0.125817	0.701	0.637	1
St3gal1	0.027637	-0.11332	0.585	0.634	1
Rack1	0.027725	0.103028	1	0.995	1
Baz1b	0.027813	0.127191	0.629	0.574	1

Col23a1	0.027869	0.266608	0.439	0.389	1
Sqstm1	0.028615	0.139986	0.929	0.898	1
Rps12	0.028766	0.138252	1	0.998	1
Ets2	0.028959	0.117529	0.759	0.694	1
Uqcrc2	0.029279	0.104111	0.622	0.555	1
Zcchc14	0.029586	0.117563	0.711	0.661	1
Arid5b	0.030212	0.14507	0.847	0.811	1
Gls	0.030256	0.186037	0.901	0.897	1
Irf1	0.030409	0.107692	0.81	0.779	1
Paxbp1	0.03044	0.120189	0.35	0.299	1
Egr1	0.030441	0.112596	0.997	0.995	1
Stc1	0.030998	-0.22609	0.17	0.221	1
Ube2h	0.031616	0.1075	0.82	0.754	1
Luc7l2	0.031619	0.126581	0.925	0.919	1
Klf2	0.031636	0.266233	0.793	0.76	1
Psma5	0.031696	0.114264	0.738	0.683	1
Gm13889	0.03214	0.126051	0.265	0.213	1
Mta1	0.032571	0.103746	0.493	0.434	1
Ebf1	0.033646	-0.20406	0.915	0.932	1
Dnaja2	0.03418	0.137709	0.799	0.775	1
Gnai2	0.034538	0.100777	0.98	0.97	1
Brd3	0.034617	0.108551	0.554	0.507	1
Dpysl3	0.034653	-0.1058	0.595	0.651	1
Ramp3	0.035131	-0.27971	0.248	0.292	1
Lrp6	0.035197	0.126129	0.69	0.661	1
Pdlim5	0.035274	-0.1056	0.619	0.667	1
Kansl1	0.035533	0.101675	0.667	0.611	1
Inf2	0.035642	-0.26364	0.527	0.551	1
Spaca6	0.036498	0.172866	0.401	0.358	1
Sema3c	0.036711	-0.13094	0.452	0.504	1
Rab11fip2	0.036979	-0.11088	0.548	0.59	1
Gnaq	0.037252	0.116459	0.721	0.703	1
mt-Co2	0.037848	-0.11039	1	1	1
Ccnd2	0.037994	-0.12504	0.595	0.637	1
Cyth3	0.03846	0.15266	0.759	0.729	1
Cdc5l	0.038897	0.104062	0.561	0.497	1
Tek	0.038911	-0.13694	0.238	0.292	1
Pdlim7	0.039011	0.116353	0.561	0.522	1
Hk2	0.039177	0.123761	0.561	0.504	1
Safb	0.039316	0.108098	0.667	0.621	1
Pcm1	0.039774	0.141901	0.619	0.572	1
Cdk12	0.040632	0.116107	0.694	0.645	1
Col4a4	0.041518	-0.126	0.34	0.394	1

Ptges	0.041647	0.126321	0.639	0.594	1
Serpinf1	0.041655	-0.10222	0.959	0.971	1
Bdp1	0.042301	0.106039	0.49	0.431	1
Sgk1	0.042436	0.130414	0.952	0.951	1
Kmt2d	0.042451	0.104675	0.517	0.445	1
mt-Co3	0.043127	-0.11004	1	1	1
Hnrnpd	0.043507	0.109334	0.68	0.652	1
Cited2	0.043572	0.142005	0.578	0.521	1
Prmt1	0.044146	0.110257	0.558	0.512	1
Atp2b4	0.044238	0.165585	0.687	0.637	1
Dab2ip	0.044289	0.145567	0.391	0.345	1
mt-Nd4	0.044379	-0.11883	1	1	1
Kmt5a	0.044665	0.102819	0.378	0.33	1
Clec2d	0.044887	-0.13193	0.333	0.383	1
Gapvd1	0.045352	0.108271	0.633	0.582	1
Slc25a5	0.045571	0.131035	0.898	0.869	1
Pcdh7	0.045745	0.117758	0.395	0.344	1
Ankrd26	0.04696	0.114125	0.173	0.136	1
Bcl9l	0.047424	0.109983	0.701	0.669	1
Cct5	0.047822	0.105579	0.755	0.738	1
Adam33	0.048648	-0.10372	0.452	0.491	1
Rbbp4	0.048733	0.106192	0.793	0.732	1
Gm12840	0.049391	0.144101	0.221	0.175	1
Tle3	0.050116	0.11077	0.527	0.47	1
Nrn1	0.050349	0.236855	0.578	0.533	1
Vezf1	0.050804	0.118531	0.84	0.794	1
Slk	0.051964	0.148058	0.741	0.718	1
Lamc3	0.052107	0.140277	0.473	0.415	1
Insig1	0.052695	0.123578	0.684	0.619	1
Dlg4	0.05281	0.154374	0.622	0.566	1
Ggt5	0.053171	-0.19595	0.364	0.402	1
Atp2b1	0.054261	0.195966	0.85	0.812	1
Kdm1a	0.054391	0.100687	0.687	0.635	1
Crebbp	0.055241	0.10969	0.864	0.843	1
Anp32a	0.057004	0.156776	0.646	0.603	1
Ssr3	0.057407	-0.1011	0.895	0.934	1
Tln1	0.057448	0.124474	0.718	0.679	1
Rab5a	0.057567	0.107233	0.776	0.74	1
Ifi204	0.057986	-0.12697	0.558	0.608	1
Ing2	0.059131	0.139863	0.381	0.346	1
Rtf1	0.060012	0.1145	0.707	0.678	1
Fam198b	0.060105	-0.13725	0.395	0.429	1
Sdc2	0.060562	-0.14388	0.837	0.857	1

Tnfsf9	0.060626	0.185087	0.439	0.4	1
Rbm5	0.060901	0.100009	0.67	0.615	1
Hes1	0.061027	0.189931	0.806	0.777	1
Ktn1	0.061033	0.105081	0.595	0.545	1
Angptl4	0.062489	0.102421	0.33	0.274	1
Hoxc8	0.064212	-0.1091	0.503	0.545	1
Bclaf1	0.064824	0.133069	0.779	0.748	1
Ddit4l	0.06532	-0.16068	0.241	0.282	1
Pum1	0.06585	0.114389	0.81	0.761	1
Anxa3	0.066028	-0.26546	0.578	0.609	1
Socs3	0.067596	0.360613	0.806	0.79	1
Hacd4	0.068866	-0.10165	0.412	0.438	1
Smad5	0.07018	-0.11077	0.571	0.604	1
Usp15	0.070696	0.101239	0.476	0.428	1
Ugdh	0.071061	-0.16995	0.861	0.861	1
Gramd1a	0.072836	0.10658	0.371	0.341	1
Tubb6	0.072957	0.138013	0.534	0.484	1
Rps25	0.073467	0.100635	1	0.981	1
Gadd45g	0.073729	-0.14314	0.827	0.853	1
Cpxm1	0.074611	-0.10569	0.833	0.89	1
Jun	0.076071	0.153093	0.993	0.994	1
Dpp4	0.078331	-0.14445	0.568	0.588	1
Nrcam	0.080294	-0.10267	0.269	0.3	1
Itih5	0.083563	0.185294	0.231	0.19	1
Ncor1	0.083687	0.10176	0.871	0.832	1
Txnl1	0.083728	0.10297	0.752	0.711	1
Rassf1	0.084261	0.121739	0.684	0.674	1
Gdf15	0.084325	-0.1138	0.616	0.638	1
Apod	0.086418	-0.65896	0.871	0.839	1
Pdzk1ip1	0.086605	-0.11707	0.16	0.19	1
Ccl8	0.086613	-0.10139	0.456	0.511	1
Ccl2	0.08786	-0.31192	0.759	0.792	1
Myef2	0.090021	0.124388	0.282	0.245	1
Daam2	0.090079	0.151669	0.201	0.164	1
Serpina3c	0.090336	-0.11297	0.364	0.397	1
Ccl21a	0.092413	0.7589	0.354	0.298	1
Robo2	0.095164	0.198092	0.626	0.608	1
Zwint	0.095458	0.11874	0.469	0.42	1
Cdh11	0.09556	0.127078	0.762	0.754	1
Hmgn2	0.09598	0.110092	0.643	0.617	1
Nfatc4	0.101488	0.125254	0.605	0.57	1
Baz1a	0.103174	0.105897	0.667	0.621	1
Sirt1	0.107025	0.122475	0.633	0.595	1

Mmp3	0.107193	-0.28922	0.616	0.65	1
Ltbp4	0.107371	0.189506	0.616	0.59	1
Rrp1	0.108053	0.103548	0.592	0.574	1
Pianp	0.108856	-0.11708	0.371	0.4	1
Atp1a1	0.11228	0.100518	0.772	0.764	1
Pdpn	0.112968	-0.11175	0.755	0.832	1
Ets1	0.115337	0.133723	0.483	0.46	1
Rnf149	0.115569	0.100596	0.558	0.509	1
Ogn	0.116246	-0.12337	0.633	0.685	1
Eif5b	0.11972	0.101906	0.779	0.767	1
Tspan11	0.121797	0.101121	0.452	0.42	1
Crem	0.123053	0.114695	0.388	0.344	1
Prss23	0.127022	-0.23674	0.629	0.66	1
Emb	0.127594	0.137301	0.231	0.199	1
Fos	0.130007	0.217725	0.99	0.996	1
Sesn3	0.131271	0.100954	0.503	0.461	1
Uhrf2	0.131808	0.11678	0.622	0.601	1
Robo1	0.141992	0.110956	0.497	0.465	1
Gadd45a	0.14474	0.105583	0.483	0.441	1
Cyr61	0.146312	0.146317	0.963	0.943	1
Mirg	0.146749	-0.10806	0.412	0.461	1
Aspn	0.146814	0.147813	0.918	0.937	1
H2-K1	0.147037	-0.11209	0.983	0.994	1
Ubr2	0.147629	0.108716	0.602	0.574	1
Tnmd	0.147823	0.123103	0.483	0.436	1
Srsf10	0.148194	0.124562	0.721	0.681	1
Stau1	0.148589	0.100908	0.51	0.471	1
Cp	0.151174	-0.17736	0.316	0.342	1
Pi16	0.155546	-0.24232	0.51	0.542	1
Uba52	0.157523	0.111902	0.963	0.954	1
Lgalsl	0.157929	0.147772	0.408	0.38	1
Twist1	0.160781	0.124167	0.956	0.959	1
Mnt	0.163667	0.105912	0.289	0.258	1
Matr3	0.167876	0.102043	0.844	0.83	1
Gm26802	0.170366	-0.1263	0.395	0.419	1
Irgm1	0.170738	0.117209	0.245	0.218	1
Cd44	0.177174	0.108108	0.677	0.655	1
Fgl2	0.183645	-0.15188	0.67	0.66	1
Socs1	0.183773	0.100611	0.643	0.596	1
Mapk8	0.185669	0.102587	0.639	0.598	1
Irf2bpl	0.197793	0.107145	0.595	0.578	1
Ralbp1	0.198444	0.154552	0.861	0.869	1
Tcf4	0.20333	0.132007	0.993	0.99	1

Tctn1	0.215611	0.122826	0.167	0.142	1
Msl2	0.217995	0.102557	0.585	0.534	1
Klk1	0.223085	-0.4079	0.599	0.606	1
Mphosph8	0.22668	0.106084	0.381	0.338	1
Rps27rt	0.237735	0.100998	0.793	0.747	1
Fam107b	0.238582	0.110927	0.248	0.224	1
Mgp	0.250342	0.314237	0.269	0.239	1
Bmp7	0.256686	0.168614	0.446	0.419	1
Camk2n1	0.277256	0.112975	0.616	0.597	1
Acta2	0.279584	0.292765	0.361	0.323	1
Pdgfrb	0.286452	-0.10646	0.816	0.824	1
Il11ra1	0.306232	0.121187	0.874	0.899	1
Thbs1	0.310775	-0.19773	0.738	0.737	1
D10Wsu1C	0.324524	0.150671	0.367	0.338	1
Col4a1	0.32964	0.165798	0.895	0.895	1
Spp1	0.337066	-0.15827	0.102	0.084	1
Cxcl10	0.344547	0.101435	0.293	0.314	1
Masp1	0.349831	-0.12588	0.667	0.688	1
Gm42418	0.392342	-0.16466	0.997	0.999	1
Rspo1	0.41661	0.140855	0.469	0.438	1
Plat	0.416929	-0.14086	0.425	0.439	1
Klk1b27	0.445799	-0.4921	0.5	0.503	1
Col4a2	0.447507	0.190029	0.806	0.795	1
Cxcl2	0.45154	-0.21932	0.293	0.269	1
Sdc4	0.454198	0.231035	0.895	0.888	1
Klf7	0.45492	0.12039	0.537	0.532	1
Twist2	0.509291	-0.10789	0.728	0.757	1
Igfbp2	0.526854	-0.50305	0.194	0.206	1
Dclk1	0.541667	0.105276	0.707	0.708	1
Nov	0.542494	-0.15487	0.156	0.166	1
Cacna1g	0.549711	0.155009	0.524	0.513	1
Lpl	0.595368	-0.12198	0.687	0.679	1
Trf	0.702923	-0.1837	0.109	0.115	1
Cxcl12	0.718781	-0.13904	0.507	0.522	1
Cxcl1	0.82547	-0.14309	0.612	0.622	1
Adm	0.833371	-0.11049	0.378	0.355	1
F13a1	0.858168	-0.20169	0.133	0.133	1
Gm47283	0.909695	0.114924	0.265	0.276	1
Gpx3	0.909914	-0.11064	0.816	0.802	1
C2	0.925847	-0.11546	0.34	0.327	1
Ptx3	0.92674	-0.13108	0.33	0.316	1
Ctla2a	0.946409	-0.14393	0.524	0.529	1
Ptgs2	0.98618	0.101895	0.153	0.154	1
